# A Dynamic Silver(I) Nanocluster Holds Together a 3 × 3 Self-Assembled Grid

**DOI:** 10.1021/jacs.5c07271

**Published:** 2025-08-18

**Authors:** Andrew W. Heard, Luca Pesce, Peter T. Gierth, Simone Adorinni, Tanya K. Ronson, Barbara Rossi, John D. Thoburn, Tomas Deingruber, Martin Welch, David R. Spring, Silvia Marchesan, Giovanni M. Pavan, Jonathan R. Nitschke

**Affiliations:** † Yusuf Hamied Department of Chemistry, 2152 University of Cambridge, Lensfield Road, Cambridge CB2 1EW, United Kingdom; ‡ School of Chemistry, University of Birmingham, Birmingham B15 2TT, United Kingdom; § Astex Pharmaceuticals, 436 Cambridge Science Park, Milton Road, Cambridge CB4 0QA, United Kingdom; ∥ Department of Applied Science and Technology, 19032 Politecnico di Torino, Torino 10129, Italy; ⊥ Nantes Université, CNRS, CEISAM − UMR 6230, Nantes 44000, France; # Elettra Sincrotrone Trieste, Basovizza, Trieste 34149, Italy; ¶ Department of Chemistry, 1342 Randolph-Macon College, Ashland, Virginia 23005, United States; ∇ Department of Biochemistry, University of Cambridge, Cambridge CB2 1QW, United Kingdom; ○ Department of Chemical and Pharmaceutical Science, University of Trieste, Trieste 34127, Italy; ⧫ Unit of Trieste, INSTM, Trieste 34127, Italy; †† Department of Innovative Technologies, University of Applied Sciences and Arts of Southern Switzerland, Lugano-Viganello CH-6962, Switzerland

## Abstract

Metal ions with well-defined coordination geometries can serve as fixed joints within self-assembled architectures, defining the relative orientations of ligands within higher-order superstructures. The exchange of ligands and metal ions between different positions is slow, involving disruption or distortion. Here we report a series of Ag^I^_12_X_4_L_6_ 3 × 3 metal–organic grid-like structures, where the core Ag^I^_12_X_4_ nanocluster is in dynamic motion, with Ag^I^ ions moving between different binding sites, with concomitant conformational changes of the organic ligands, which continue to occupy well-defined positions nevertheless. The identity of the incorporated halide anion governs the activation barrier for silver ion exchange, thus enabling rate control in response to two distinct stimuli: by changing the temperature, and by exchanging one halide for another. The dynamic nanocluster within these grids thus provides a new mode of using metal ions in coordination-driven self-assembly, establishing that the mobile Ag^I^ ions behave in similar ways to Ag^0^ atoms in surface-bound clusters and in silver nanoparticles. The kinetic parameters determined in this work, and the techniques developed to measure them, could serve the scientific community to provide additional insight into dynamic metal nanoclusters.

## Graphical abstract

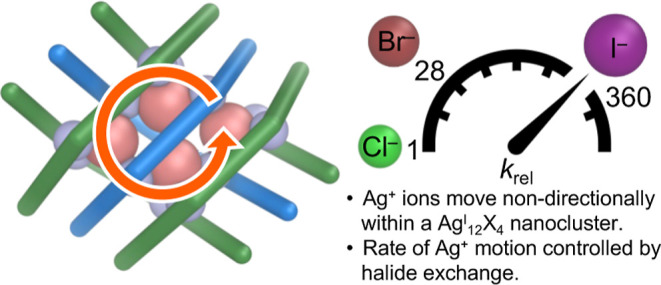

## Introduction

Metal–organic grids (Figure 1)^1–3^ assemble from rigid ligands and metal ions that serve as crossing points between these ligands. They have been used as the frameworks of interlocked molecules,^4,5^ two-dimensional molecularly woven fabrics,^6^ and single molecule magnets.^7^ Noteworthily, such grids typically consist of coordinatively saturated metal ions that each define a junction point between two ligands,^2^ although solvophobic and stacking effects may outweigh coordinative saturation.^8^

**Figure 1:**
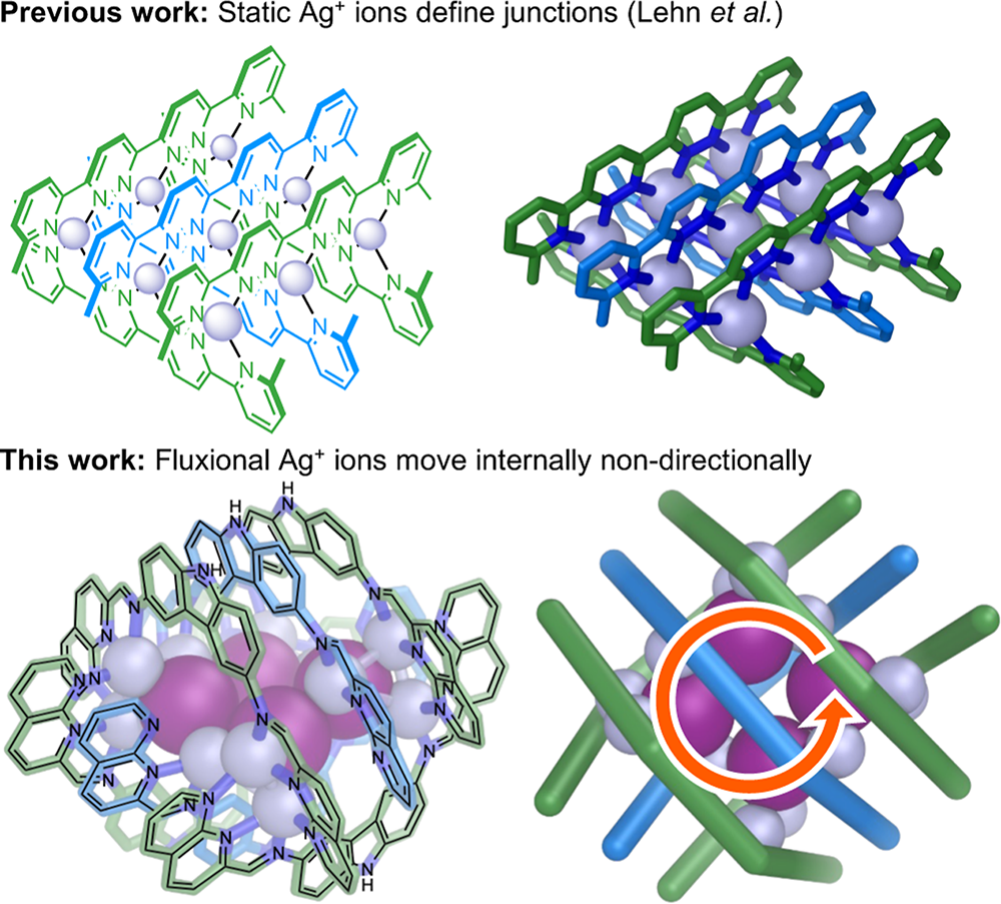
Comparison between the original Ag^I^_9_L_6_ 3 × 3 metal–organic grids of Lehn et al.,^3^ where each Ag^I^ ion defines a fixed junction between ligands, and the present work describing Ag^I^_12_X_4_L_6_ 3 × 3 metal–organic grids where the Ag^I^ ions can move through the structure in either direction.

Metal-ion nanoclusters are finding uses in nanotechnology^9^ and supramolecular chemistry,^10–12^ thanks to their catalytic abilities^13^ and photoluminescent properties.^14^ Metal ions may be placed precisely within clusters through bridging ligands^15^ and anion templation.^16^ Macrocycles^17^ and metal–organic cages^18–21^ have been shown to stabilize atomically precise silver clusters. Trigonal prisms^18^ and octahedra^19^ are known with Ag^I^_2_ vertices, along with six-stranded helicates containing Ag^I^_4_X (where X = halide) and Ag^I^_6_(SO_4_)_2_ cluster vertices,^20^ double-walled tetrahedra with Ag^I^_4_X cluster vertices,^21^ and tetrahedra with Ag^I^_3_X vertices.^22^ Small metallocene-type silver clusters have been shown to display dynamic coordination.^11^ However, transformations in metal nanoclusters are challenging to characterize.^9,23,24^ Understanding the complex dynamics and mechanisms of atom movement in nanoparticles,^24,25^ on surfaces,^26^ and in clusters^27–30^ is important to understand the ongoing transformative processes underlying heterogeneous catalysis.^31,32^ However, experimental determination of rate constants and energy barriers to atom exchange in clusters remains difficult.^25,33^

Here we report a family of 3 × 3 grid-like structures held together by a fluxional Ag^I^_12_X_4_ nanocluster core, wherein all 12 of the Ag^I^ ions are observed to exchange with each other. The grid-like structures reported in this work, herein referred to as grids, contain additional peripheral metal ions, thus exceeding the nine metal ions present in a traditional 3 × 3 grid.^1^ Using nuclear magnetic resonance (NMR) spectroscopy, we elucidate the rate constants and activation barriers for Ag^I^ exchange within the dynamic silver­(I)-halide nanoclusters. Utilization of these bespoke NMR pulse programs may allow determination of energy barriers in dynamic nanoparticle systems with NMR-active metal ions. Importantly, the dynamic silver­(I)-halide nanocluster that binds the ligands represents a conceptual departure from the typical use of metals as fixed structural elements in self-assembled structures.^34^ Such dynamic-metal junctions may enable the preparation of further new classes of self-assembled dynamic and functional structures. Ultimately, the use of NMR spectroscopy, as a tool for the quantification of key parameters in dynamic silver nanoclusters,^9,24,26,28^ can be useful to the wider community of scientists interested in dynamic nanoclusters.

## Results and Discussion

### Synthesis and Characterization of **1-I**

The reaction between 3,6-diamino-9*H*-carbazole **A** (3 equiv), 2-formyl-1,8-naphthyridine **B** (6 equiv), silver­(I) bis­(trifluoromethanesulfonyl)­imide (triflimide, Tf_2_N^–^, 6 equiv) and tetra-*n*-butylammonium iodide (TBAI, 2 equiv) in acetonitrile formed **1-I** as the uniquely observed product (Figure 2a). Single crystal X-ray diffraction revealed **1-I** to be a *D*_2_-symmetric Ag^I^_12_I_4_L_6_ 3 × 3 grid-like structure, with ditopic ligands surrounding a small central cavity that contains four iodide anions, each forming part of an Ag^I^_3_I subcluster within a larger Ag^I^_12_I_4_ nanocluster (Figures 2b, Supporting Information Section 5.1). **1-I** is reminiscent of a 3 × 3 grid, but contains 12 silver­(I) ions, thus exceeding the nine metal ions that define the junctions of a 3 × 3 grid as traditionally defined.^1^

**Figure 2:**
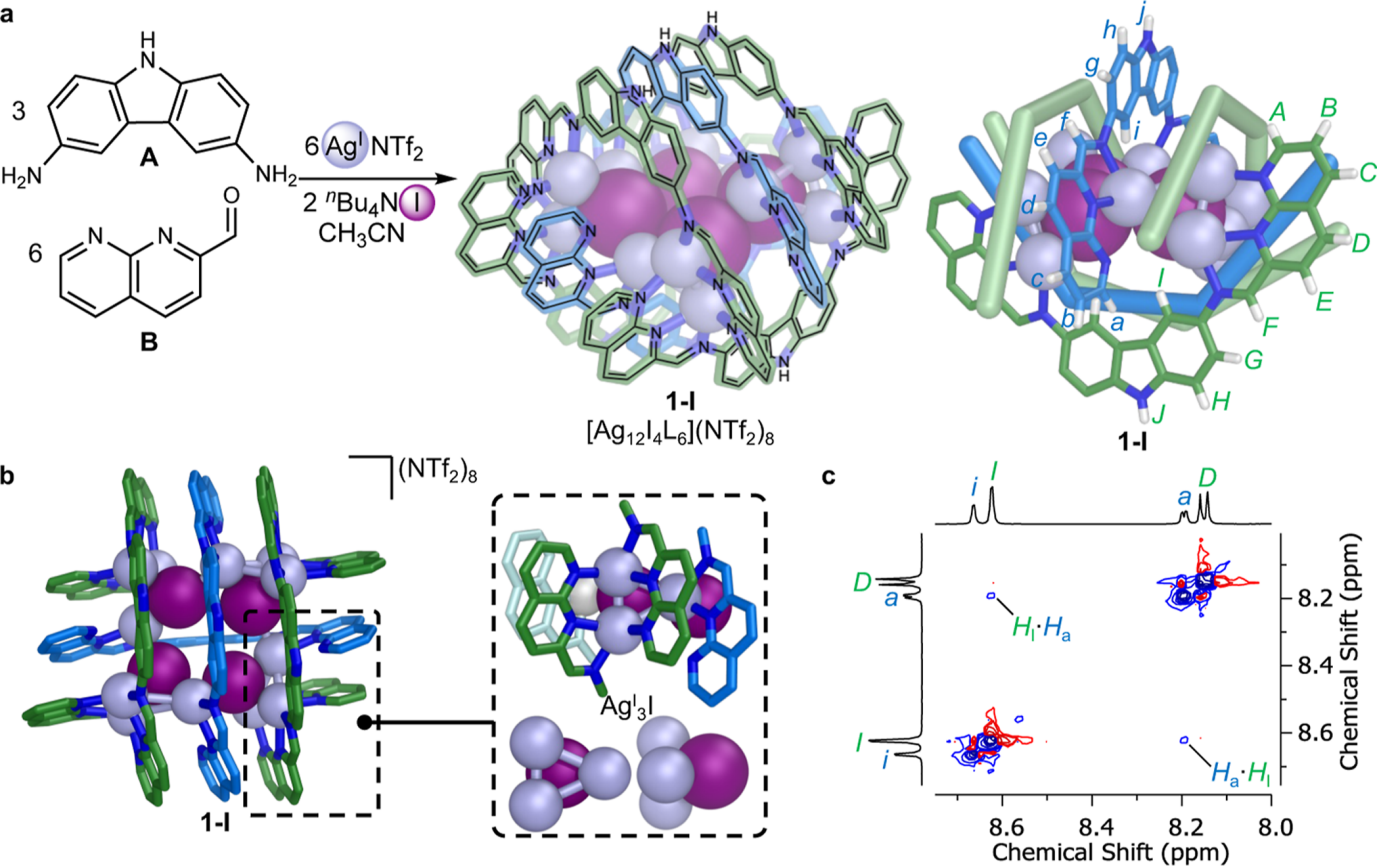
Synthesis and characterization of **1-I**, [Ag^I^_12_I_4_L_6_]­(NTf_2_)_8_. (a) Self-assembly of **1-I** from subcomponents **A** and **B**, silver­(I) triflimide and tetra-*n*-butylammonium iodide. (b) X-ray crystal structure of **1-I** with inset showing the Ag^I^_3_I subcluster structure within the Ag^I^_12_I_4_ nanocluster core. (c) Partial ^1^H–^1^H NOESY NMR spectrum showing H_I_···H_a_ correlations (500 MHz, CD_3_CN, 298 K).

NMR spectra (Figures S1–S12), high-resolution electrospray ionization mass spectrometry (ESI-MS) (Figures S13–S15), and UV resonance Raman spectra of **1-I** are consistent with the persistence of this [Ag^I^_12_I_4_L_6_]­(NTf_2_)_8_ grid structure in solution (Supporting Information Section 7.2). The ^1^H NMR spectrum shows two ligand environments in a 2:1 ratio at all temperatures measured (Figure S4), implying a thermally averaged *D*_2d_-symmetric solution conformation for **1-I**, with a low barrier between the two enantiomeric *D*_2_-symmetric conformations observed in the solid state (Figure 2b). Nuclear Overhauser enhancement spectroscopy (NOESY) NMR displayed correlations between the two sets of signals (Figures 2c, S9), confirming the presence of parallel ligands in **1-I**.

The X-ray structure of **1-I** (Figures 2b, Supporting Information Section 5.1) elucidates the roles played by various noncovalent interactions in holding the structure together, and suggests how the silver­(I) ions may exchange between positions. The four iodides play an essential bridging role forming the core Ag^I^_12_I_4_ nanocluster. Each iodide forms a vertex of a distorted tetrahedron whose other vertices are silver­(I) ions, with Ag^I^–I^–^ bonds 2.58–2.79 Å in length. Each iodide also bridges to a silver­(I) vertex of an adjacent Ag^I^_3_I tetrahedron, with longer Ag^I^–I^–^ bonds ranging from 2.96 to 3.02 Å. These distances are all within the 3.70 Å sum of van der Waals radii for Ag^I^ and I^–^. In the absence of halide, no discrete assembly was observed to form, and instead the solution was observed to gel.

We infer that argentophilic interactions between the three Ag^I^ ions (Figure 2b) also stabilize **1-I**, with an average Ag^I^···Ag^I^ separation of 3.14 Å within each Ag^I^_3_I unit, again within the 3.44 Å sum of van der Waals radii. Aromatic stacking between ligands further stabilizes the structure, with an average separation of 4.01 Å. No silver­(I) ion is close enough to coordinate to the terminal naphthyridine nitrogen atoms of the central crossing ligands (blue, Figure 2a), suggesting that these donors may play a role in enabling the silver ions to circulate between the Ag^I^_3_I units, as detailed below. Stacking interactions may help bind these coordinatively unsaturated ligands in place, in a manner reminiscent of the 3 × 3 grid reported by Siegel et al., where the central ligands were bound by stacking interactions alone.^8^

### Characterization of Fluxional Ag^I^-Clusters

The ^1^H–^109^Ag heteronuclear multiple bond correlation (HMBC) NMR spectrum of **1-I** at 232 K (Supporting Information Section 3.2) showed two ^109^Ag signals at 706 and 805 ppm in a 1:2 integral ratio. These signals were assigned to the single silver atoms at the edges of the grid (Ag_*B*_, Figure 3a), and the pairs of silver ions defining the grid corners (Ag_*A*_, Figure 3a), respectively. Our assignments were supported by the observation of ^1^H–^109^Ag correlations between the corner Ag_*A*_ nuclei and the outer ligands (green), and between the edge Ag_*B*_ ions and the central ligands (blue). Selective ^109^Ag-decoupled ^1^H NMR spectra indicated that each ^109^Ag environment is correlated to only one set of ligand signals at 232 K (Figure S20). Above 252 K, the ^109^Ag signals broadened and both ^109^Ag environments showed HMBC correlations to both sets of ligand ^1^H signals (Figures S21–S24), consistent with exchange between the two environments (Figure 3a). Furthermore, at 232 K each imine signal (from H_F_ and H_f_) was observed as a doublet, resulting from ^1^H–^109^Ag *J*-coupling; however above 252 K these *J*-couplings disappeared, and the signals appeared as broadened singlets.

**Figure 3:**
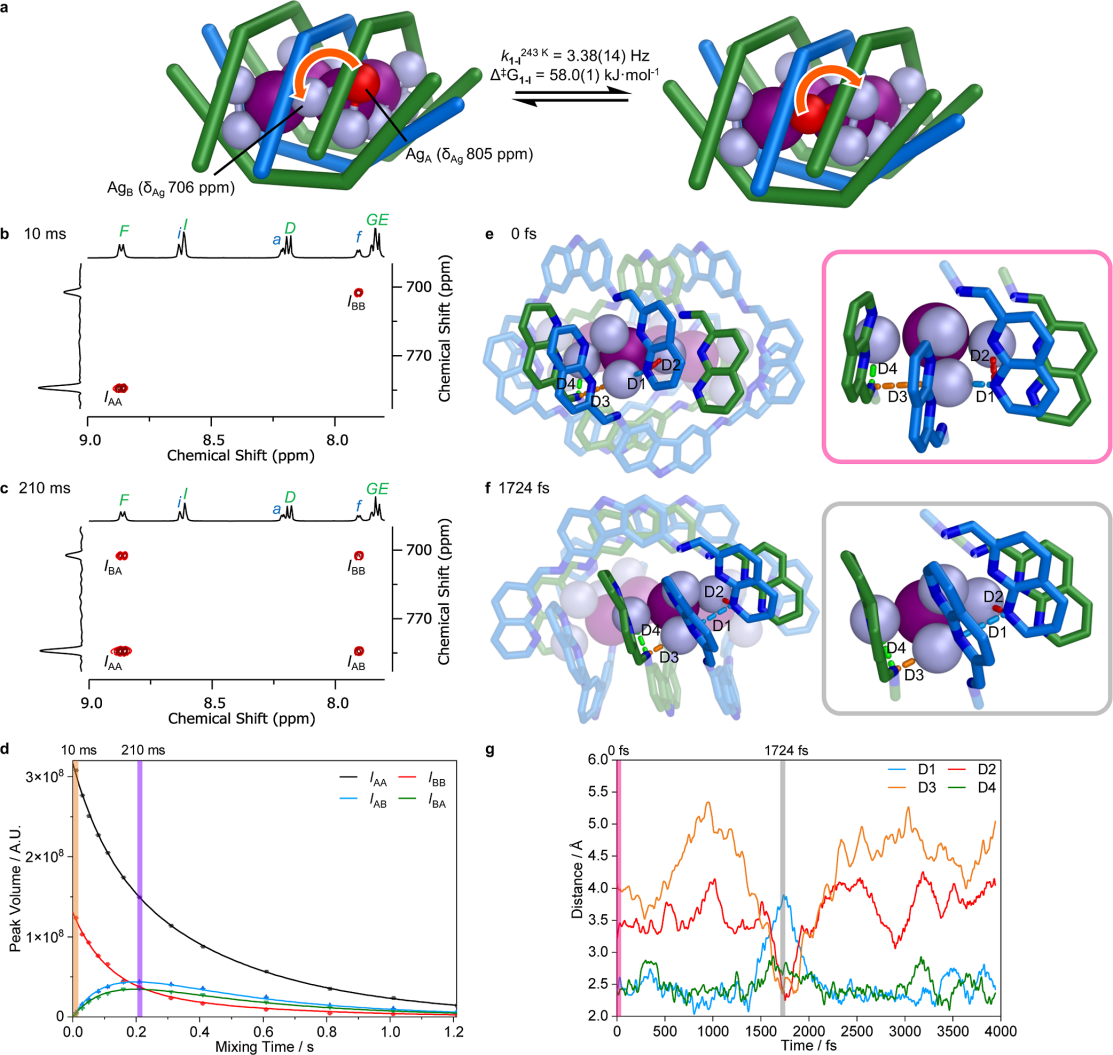
NMR characterization and molecular dynamics simulation of silver ion movement within **1-I**. (a) Cartoon depicting rotation of silver ions within one Ag^I^_3_I cluster within the Ag^I^_12_I_4_ nanocluster core of **1-I**. Silver NMR chemical shifts were determined at 232 K. (b) Partial ^1^H–^109^Ag HSQC-EX NMR spectrum at 243 K with 10 ms mixing time, showing no ^109^Ag exchange. (c) Partial ^1^H–^109^Ag HSQC-EX NMR spectrum at 243 K with 210 ms mixing time, showing maximal ^109^Ag exchange between environments. (d) Plot of the imine-silver cross-peak signal volumes from HSQC-EX spectra at 243 K, as the mixing time increased from 10 to 1210 ms. Diagonal peaks *I*_AA_ and *I*_BB_ decreased in volume with increasing mixing time due to relaxation and exchange. Exchange peaks *I*_AB_ and *I*_BA_ initially increased in volume due to exchange, finding maxima at approximately 210 ms, then decreasing due to relaxation. Orange and violet lines correspond to the 10 and 210 ms HSQC-EX spectra shown in Figure 3b,c, respectively. (e) The starting configuration of a QM/MM MD simulation of **1-I** at 243 K with explicit solvent. (f) The configuration at 1724 fs of this QM/MM MD simulation, showing the furthest displacement of the migrating Ag^I^ ion. (g) Plot of four key N···Ag distances over the course of a 4000 fs QM/MM MD simulation. Pink and gray lines indicate the 0 and 1724 fs configurations shown in Figure 3e,f, respectively.

Variable temperature ^1^H–^109^Ag heteronuclear single quantum coherence exchange NMR spectroscopy of **1-I** (HSQC-EX, Figures 3b,c, Supporting Information Section 4), adapted from a ^1^H–^15^N exchange NMR pulse sequence,^35^ was undertaken to probe silver exchange within the nanocluster (Figure 3b). The concentration of **1-I** was determined to be 16 mM relative to an external ethylbenzene standard (Supporting Information Section 4.4). The ^1^H–^109^Ag HSQC-EX spectra showed correlations only between each imine environment (H_F_ and H_f_) and the Ag^I^ ion it was most closely associated with (Figure 3b, *I*_AA_, *I*_BB_) at 243 K with a mixing time of 10 ms. Increasing the mixing time (*T*_m_) in increments from 10 to 1210 ms resulted in the appearance of exchange correlation peaks (Supporting Information Section 4.6). The volume of the exchange peaks (Figure 3c, *I*_AB_, *I*_BA_) reached a maximum at a mixing time of 210 ms (Figure 3c) before decreasing at longer mixing times as the signals relax (Figure 3d). These HSQC-EX spectra thus confirmed the exchange of ^109^Ag between the edge and corner environments.

Corresponding 2D ^1^H–^1^H exchange NMR spectroscopy (EXSY) measurements showed there was no exchange between the two ligand ^1^H environments on the same time scale (Supporting Information Section 4.10). Taken together, the ^1^H–^109^Ag HSQC-EX and ^1^H–^1^H EXSY NMR results indicated that ^109^Ag exchange does not occur via a disassembly reassembly process, but that Ag^I^ ions continuously move around the inside of the Ag^I^_12_I_4_ nanocluster in **1-I** due to an intramolecular ion-migration process (Supporting Movie 1). As dissociation of at least two ligands would be required to exchange the two Ag environments, we infer that complete or partial dissociation of **1-I** cannot be responsible for the Ag exchange.

The volumes of the four correlation peaks of the HSQC-EX spectra were fitted (Supporting Information Section 4.5), providing a total magnetization exchange rate constant, *k*_**1‑I**_^243 K^, of 3.38(14) Hz (Supporting Information Section 4.6). Due to the double occupancy of site Ag_*A*_ and single occupancy of site Ag_*B*_, the observed magnetization exchange rate constant is double the exchange rate between each site (*k*_A→B(**1–I**)_^243 K^ = 1.69(07) Hz), corresponding to a Gibbs free energy of activation (Δ*G* ^‡^_**1‑I**_) of 58.0(1) kJ·mol^–1^ (Supporting Information Section 4.11).

### Mechanism of Silver Exchange

The dynamic motion of silver ions observed (Figure 3b–d) within **1-I** was further investigated through a combination of computational approaches (Supporting Information Section 8). Quantum mechanics/molecular mechanics (QM/MM) molecular dynamics (MD) simulations (Figures 3e–g, Supporting Information Section 8.1, S179) investigated the flexibility of the grid structure, regarding the torsional dynamics of the naphthyridine moieties. These simulations showed that the naphthyridine moieties of **1-I** can rotate, imparting flexibility into the coordination grid and its cavity. These dynamics, observed in the QM/MM MD simulations (Supporting Movie 2), are associated with the motion of the Ag^I^ ions in **1-I.** Following the dynamic rearrangements of the grid, the Ag^I^ ions in **1-I** can temporarily unbind from the outer coordinating naphthyridine nitrogen atoms, which triggers a series of dynamical rearrangements in the Ag^I^_3_I cluster (Figure 3g).

QM/MM MD simulations (Figure 3e–g) showed how the initial starting configuration (Figure 3e) evolved over 4000 fs. Figure 3g charts the four key N···Ag distances within one Ag^I^_3_I cluster, showing how at 1724 fs (Figure 3f) the N···Ag distances D2 and D3 fall within N–Ag bonding distance (2.19–2.46 Å, as determined from the crystal structure of **1-I**). The simulation also showed that at 1724 fs, D1 is elongated beyond the sum of van der Waals radii of N and Ag (3.27 Å). Altogether, these measurements suggest the transition of an Ag^I^ ion between two N donor sites at 1724 fs, before returning to its initial position.

Classical MD simulations (Supporting Information Section 8.2) are useful to assess the extent to which the grid is dynamic under experimental conditions of temperature and solvent.^36^ Our simulations highlight that dynamic ligand motions are possible in **1-I** in experimental regimes, which could facilitate the exchange of silver ions within the central cluster. The ligands oscillate around an equilibrium configuration, leading to a continuous dynamic motion of the grid and of its cavity, which can trigger the ions to exchange between positions within the cluster (Supporting Movies S3, S4 and S5). The rapid, high-amplitude ligand motion observed in the simulation supports the hypothesis that such structural dynamics are associated with the silver exchange mechanism. We infer that this motion may mediate a change in the coordination mode of the Ag^I^ ion attached to the central ligand, from bidentate coordination to the imine and innermost naphthyridine nitrogen atom, to coordination at the outer naphthyridine nitrogen atom. This motion of the central ligand could create defects in the internal coordination environment, which may in turn facilitate the exchange of Ag^I^ ions inside **1-I**.

This proposed mechanism, based on observations from the simulations, represents a simplified view of a more complex process, with contributions from other minor pathways. Structure dynamics, global and local rearrangements can influence the mechanism. The millisecond resolution of the NMR evidence provided is significantly longer than the picosecond simulation time scale, limiting the mechanistic conclusions that can be confidently asserted from the data. The NMR evidence provides an averaged overview of the process and allows extraction of kinetic information for the global process. The high activation barrier observed experimentally suggests that there is rotation of each Ag^I^_3_I cluster, rather than simultaneous concerted movement of all 12 Ag^I^ ions, and that such dynamics follow (and are likely permitted by) the dynamics of the grid, revealed by the picosecond-scale simulations.

The hypothesized change in coordination mode of Ag^I^, from innermost to outer naphthyridine nitrogen atom, is reminiscent of the molecular ball-bearing of Shionoya et al.^37^ where nearby vacant coordination sites facilitate the independent rotation of coaxial ligands relative to one another.

### Halide-Controlled Exchange Rates

When tetra-*n*-butylammonium chloride or bromide was used in place of the iodide, 3 × 3 grids **1-Cl** and **1-Br** were prepared in analogous fashion to the reaction shown in Figure 2a (Supporting Information Sections 3.3–3.6). Both were characterized by NMR and Raman spectroscopies, ESI-MS, and single-crystal X-ray diffraction (Supporting Information Section 5.2–5.3). The X-ray structures revealed a greater degree of rhombic distortion in **1-Cl** and **1-Br** than in **1-I**, although low temperature (232 K) ^1^H NMR did not freeze out the two environments expected for a rhombic *D*_2_-symmetric grid (Figures S48 and S71), implying a similarly low barrier to the rocking motion that resulted in thermally averaged *D*_2d_ symmetry seen by NMR for **1-I**.

These rhombic distortions caused subtle variations in the structures of the central Ag^I^_12_X_4_ nanoclusters. Complex **1-Cl** had two distinct types of vertices within the Ag^I^_12_Cl_4_ core (Figure 4b): a Ag^I^_4_Cl cluster in each obtuse vertex, and Ag^I^_2_Cl clusters in the acute vertices. **1-Br** had mixed clusters, with Ag^I^_2_Br, Ag^I^_3_Br and Ag^I^_4_Br clusters (Figure 4c). The thermally averaged *D*_2d_-symmetry observed in solution indicates that these differences in cluster structure may be an artifact of crystallization, simplifying to two Ag-environments in solution. The larger, softer halides were observed to favor the Ag^I^_3_X cluster in the solid state, which mediates faster Ag exchange (see below). With the smaller halide anions, the distances between parallel ligands were reduced from 4.01 Å in **1-I** to 3.78 Å and 3.68 Å for **1-Cl** and **1-Br** respectively, implying an increased contribution from aromatic stacking to stabilize the structures.

**Figure 4:**
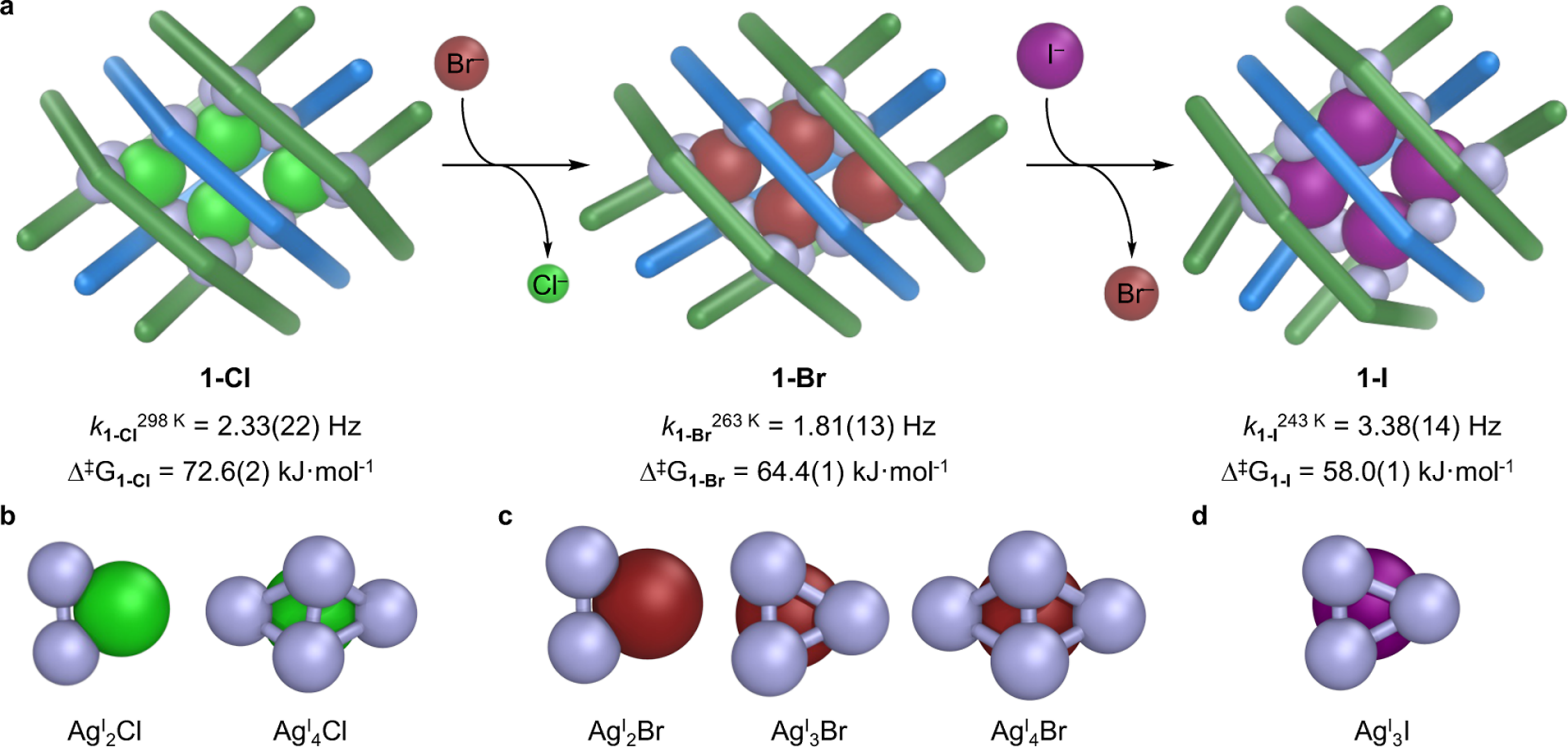
Halide exchange enabled the transformation between grids, where halide identity determined silver cluster nuclearity and geometry. (a) Halide exchange transformed **1-Cl** to **1-Br** and finally **1-I**. Total silver magnetization exchange-rates (*k*_1‑X_^T^) increase as the halide component increases in softness, decreasing the Gibbs free activation energy (Δ*G* ^‡^_1‑X_). (b) Ag^I^_2_Cl and Ag^I^_4_Cl clusters observed in the crystal structure of **1-Cl**. (c) Ag^I^_2_Br, Ag^I^_3_Br and Ag^I^_4_Br clusters observed in the crystal structure of **1-Br**. (d) Ag^I^_3_I cluster observed in **1-I**.

Halide exchange (Figures 4a, Supporting Information Section 6) enabled conversion from **1-Cl** to **1-Br** and **1-I**, and conversion of **1-Br** to **1-I**, following the hierarchy of binding affinity of the halides to silver,^21^ I^–^ > Br^–^ > Cl^–^. Variable temperature ^1^H–^109^Ag HMBC and HSQC-EX experiments (Supporting Information Sections 3.4, 3.6, 4.7 and 4.8) showed that **1-Br** and **1-Cl** also displayed ^109^Ag exchange with different energy barriers. As with **1-I**, the ^1^H–^1^H EXSY control experiments for **1-Cl** (Figure S158) and **1-Br** (Figures S160, Supporting Information Section 4.10) showed no exchange between ligand signals, indicating that any Ag exchange occurs intramolecularly, and not by complete or partial disassembly of the grid.

To obtain HSQC-EX data for **1-Cl** and **1-Br**, the samples had to be cooled to a temperature where the exchange occurred at a measurable rate, below the frequency of the ^1^H–^109^Ag *J*-coupling constant. The total magnetization exchange rate constants (*k*_**1‑X**_^T^) for silver exchange were extracted from the fitted data (Supporting Information Section 4.5). For **1-Br**, at 15 mM concentration, exchange was observed at 263 K, with a total magnetization rate constant of *k*_**1‑Br**_^263 K^ = 1.81(13) Hz, and corresponding Δ*G* ^‡^_**1‑Br**_ of 64.4(1) kJ·mol^–1^ (Supporting Information Section 4.7 and 4.11). The ^109^Ag exchange in **1-Cl** had the highest activation barrier, requiring the sample to be warmed to 298 K to observe exchange (Supporting Information Section 4.8). The exchange in **1-Cl**, at 20 mM concentration, occurred with a *k*_**1‑Cl**_^298 K^ = 2.33(22) Hz, corresponding to Δ*G* ^‡^_**1‑Cl**_ = 72.6(2) kJ·mol^–1^ (Supporting Information Sections 4.8 and 4.11).

To further clarify that the silver exchange mechanism is intramolecular, the ^1^H–^109^Ag HSQC-EX NMR experiments were repeated for **1-Cl** following 6-fold dilution (3 mM) (Supporting Information Section 4.9). Below this concentration, the signal-to-noise ratio decreased. The total magnetization rate constant determined under these conditions was 2.89(30) Hz, giving a corresponding activation barrier of Δ*G* ^‡^_**1‑Cl**_ = 72.1(3) kJ·mol^–1^ (Supporting Information Section 4.9 and 4.11). The rate constant and activation barrier obtained for **1-Cl** at 20 mM is within the 95% confidence window of the 3 mM measurement. The good agreement between these two values implies that concentration has a minimal effect on the silver-exchange rate.

These experiments determined the hierarchy of exchange rates, with the larger and softer halides having a lower barrier to Ag^I^ exchange Δ*G* ^‡^_**1‑I**_ **<** Δ*G* ^‡^_**1‑Br**_ **<** Δ*G* ^‡^_**1‑Cl**_ (Figures 4a, Supporting Information Section 4.11). As expected, the exchange rate also depended on temperature. The rate for **1-I** at 243 K was faster than in **1-Cl** at 298 K, *k*_**1‑I**_^243K^ > *k*_**1‑Cl**_^298 K^, despite the 55 K temperature difference. These HSQC-EX experiments reveal that exchange rate depends on both temperature and halide identity. The calculated room temperature total magnetization exchange rate constants for **1-I** (*k*_**1‑I**_^298 K^) and **1-Br** (*k*_**1‑Br**_^298 K^) were 832 and 65.0 Hz respectively, showing 360-fold and 28-fold rate acceleration upon exchange of chloride for iodide or bromide, respectively (*k*_**1‑I**_^298K^ > *k*_**1‑Br**_^298K^ > *k*_**1‑Cl**_^298 K^).

As ligand vibration is likely to apply force to the cluster in such a way as to result in silver movement (Supporting Information Section 8), the shorter interligand separation observed in **1-Cl** and **1-Br** likely dampens ligand motion so as to impede ion motion. The observation that Ag^I^ moves most rapidly in **1-I** is likely due to both the increased separation between parallel ligands, resulting from the larger size of the iodide anions, and the shorter Ag···Ag separations in **1-I** (Supporting Information Section 5.1), resulting from the stronger Ag–I coordination bonds. The ligands in **1-Cl** and **1-Br** are spaced closer together, damping ligand vibrations and increasing the barrier to silver exchange. The size of the halides incorporated into these 3 × 3 grids governs the vibrational freedom of the central ligand, lowering the barrier to motion by 14.6(3) kJ·mol^–1^ in going from **1-Cl** to **1-I**, resulting in a 360-fold rate acceleration.

The rate of Ag^I^ ion movement within metal–organic grids **1-Cl**, **1-Br** and **1-I** is comparable to the rate of movement within Ag^0^ clusters observed by scanning transmission electron microscopy on surfaces.^9,33,38,39^ This 3 × 3 grid model cluster system allows rates of cluster flux and energy barriers to be probed by NMR spectroscopy, providing insight into cluster,^27^ nanoparticle,^25^ and surface^9,26^ dynamics, which are challenging to characterize^25,33^ and where transient metastable states have significant impacts on heterogeneous catalysis.^31,32^ Our NMR method adds to the range of analytical methods available to study dynamic nanoclusters.^10,11,26,27,30,33,40^ This ^1^H–^109^Ag HSQC-EX NMR technique enabled the characterization of dynamic silver nanoclusters; however, mM concentrations were required. The fast relaxation of quadrupolar ^63^Cu nuclei, the broad line-widths of ^195^Pt, and the low frequencies of ^197^Au nuclei may limit the applicability of this technique to nanoclusters of these other metals. A further discussion of the ideal nuclei properties is included in the Supporting Information (Section 4.3).

## Conclusions

In conclusion, we report a series of metal–organic grid-like structures held together by a core Ag^I^_12_X_4_ nanocluster, where the silver­(I) ions exchange freely among themselves. Studies of this exchange elucidated the factors that govern it, where rates can be governed by halide exchange, as well as by changing the temperature. Bespoke silver-exchange NMR spectroscopy techniques enabled quantification of the barriers to silver motion, which range from 58.0 to 72.6 kJ·mol^–1^. Our measurements and computational studies revealed the transition state for ion migration, and the key role of a longitudinal rocking motion of the central ligand, which can trigger movement of the individual ions. These studies further explain why the more compact structures with smaller halides have a larger barrier to motion than the larger halide analogues. Use of these NMR characterization techniques to study motion in fluxional metal clusters could prove useful in the fields of ion-conductive materials,^41^ and in heterogeneous catalysis,^9,32^ where the dynamics between metastable isomers of catalytically active cluster species is important to understanding and optimizing a catalytic process.^28,31,32,39^ The quantification of ion motion rates in nanoclusters by NMR spectroscopy, advances the understanding of structural dynamics in materials, and provides a new characterization technique for research on fluxional nanostructures, although the reported spectroscopic methodology may have limited applicability to ^63^Cu, ^195^Pt and ^197^Au.

## Supplementary Material















## Data Availability

Details on the molecular models and on the molecular simulations, and additional MD data are provided in the Supporting Information. Complete details on the molecular models and simulations (simulation parameters, input files, trajectories, etc.) are available at https://zenodo.org/records/14887862.

## Notes

Synthetic experimental procedures and characterization data: NMR spectroscopy, mass spectrometry, Raman spectroscopy, X-ray diffraction. Details of computational studies. Supporting Information (Supporting Information.pdf) (PDF)
^1^H–^109^Ag HSQC-EX NMR Pulse Sequence (hsqcetexgpnd.3.ptg) (TXT)
Supporting Movie 1 (Supporting Movie 1.avi) (AVI)
Supporting Movie 2 (Supporting Movie 2.mp4) (MOV)
Supporting Movie 3 (Supporting Movie 3.mp4) (MOV)
Supporting Movie 4 (Supporting Movie 4.mp4) (MOV)
Supporting Movie 5 (Supporting Movie 5.mp4) (MOV)

## Abbreviations

Å: Angstrom
ESI-MS: electrospray ionization mass spectrometry
EXSY: exchange spectroscopy
fs: femtosecond
Δ*G* ^‡^_1‑X_: Gibbs free activation energy
HMBC: heteronuclear multiple bond correlation
HSQC-EX: heteronuclear single quantum coherence exchange
Hz: Hertz
K: Kelvin
*k*_**1‑X**_^T^: total magnetization exchange rate constants
kJ·mol^–1^: kilojoule per mole
MD: molecular dynamics
ms: millisecond
NMR: nuclear magnetic resonance
NOESY: nuclear Overhauser effect spectroscopy
ps: picosecond
QM/MM: quantum mechanics/molecular mechanics
TBAI: tetra-*n*-butylammonium iodide
*T*_m_: mixing time
UVRR: UV Resonance Raman
X: halide anion
