# Supplementary material for: A Dynamic Silver(I) Nanocluster Holds Together a 3 × 3 Self-Assembled Grid
Source: J Am Chem Soc. 2025 Aug 18;147(34):30842–50. doi: 10.1021/jacs.5c07271 (PMC12395403; doi:10.1021/jacs.5c07271)
Supplement: Supplementary file 1 [file ja5c07271_si_001.pdf]

Supporting Information

A Dynamic Silver(I) Nanocluster Holds Together a 3×3 Self-Assembled Grid

Andrew W. Heard,[1,2,3] Luca Pesce,[4,5] Peter T. Gierth,[1] Simone Adorinni,[1] Tanya K. Ronson,[1] Barbara Rossi,[6] John D. Thoburn,[7] Tomas Deingruber,[1] Martin Welch,[8] David Spring,[1] Silvia Marchesan,*[9,10] Giovanni Pavan,*[4,11] and Jonathan R. Nitschke*[1]

[1] Yusuf Hamied Department of Chemistry, University of Cambridge, Lensfield Road, Cambridge, CB2 1EW (U.K.)

[2] School of Chemistry, University of Birmingham, Birmingham, B15 2TT (U.K.)

[3] Astex Pharmaceuticals, 436 Cambridge Science Park, Milton Road, Cambridge, CB4 0QA (U.K.)

[4] Department of Applied Science and Technology, Politecnico di Torino, 10129 Torino, IT-8471 (Italy)

[5] Nantes Université, CNRS, CEISAM – UMR 6230, 44000 Nantes (France)

[6] Elettra Sincrotrone Trieste, Basovizza, Trieste, IT-34149 (Italy)

[7] Randolph-Macon College, Department of Chemistry, Ashland, VA, USA

[8] Department of Biochemistry, University of Cambridge, Cambridge CB2 1QW (U.K.)

[9] Department of Chemical and Pharmaceutical Science, University of Trieste, Trieste, IT-34127 (Italy)

[10] Unit of Trieste, INSTM, 34127 Trieste (Italy)

[11] Department of Applied Science and Technology, Politecnico di Torino, Corso Duca degli Abruzzi 24, Torino 10129 (Italy)

Email: Silvia Marchesan (smarchesan@units.it); Giovanni Pavan (giovanni.pavan@polito.it); Jonathan R. Nitschke (jrn34@cam.ac.uk)

Table of Contents

1. General Experimental Information S3
2. Synthesis of Subcomponents S4
2.1 Synthesis of **S1** S4
2.2 Synthesis of **A** S4
2.3 Synthesis of **B** S4
3. Synthesis and characterization of $[Ag_{12}X_4L_6](NTf_2)_8$ grids S5
3.1 Synthesis and characterization of **1-I** S5
3.2 ^{109}Ag NMR characterization of **1-I** S13
3.3 Synthesis and characterization of **1-Cl** S18
3.4 ^{109}Ag NMR characterization of **1-Cl** S27
3.5 Synthesis and characterization of **1-Br** S32
3.6 ^{109}Ag NMR characterization of **1-Br** S40
4. ^{1}H-^{109}Ag Heteronuclear Single Quantum Coherence-Exchange Spectroscopy S44
4.1 General Experimental Considerations S44
4.2 Details of the HSQC-Based Exchange Experiments S46
4.3 Optimisation of the Experimental Conditions S47
4.4 NMR Determination of Sample Concentration S48
4.5 Mathematical Description of Signal Decay S51
4.6 Exchange Measurements on **1-I** at 243 K S53
4.7 Exchange Measurements on **1-Br** at 263 K S62
4.8 Exchange Measurements on **1-Cl** at 298 K S75
4.9 Dilution Control Experiment for **1-Cl** at 298 K S87
4.10 EXSY Control Experiments S97
4.11 Summary and Analysis of ^{1}H-^{109}Ag HSQC-EX Experiments S101
5. X-ray crystallography of 1 .... S103
5.1 X-ray crystallography of grid **1-I** S103
5.2 X-ray crystallography of grid **1-Cl** S105
5.3 X-ray crystallography of grid **1-Br** S107
5.4 Analysis of crystal structures **1-Cl**, **1-Br** and **1-I** S109
6. Halide Exchange Experiments of 1 .... S110
7. Raman analysis of grids 1-Cl, 1-Br, and 1-I S114
7.1 Thin-flim Raman Spectroscopy S114
7.2 UV Resonance Raman Spectroscopy S117
8. Computational dynamics S118
8.1 Quantum Mechanics/Molecular Mechanics Molecular Dynamics Simulations S118
8.2 Classical Molecular Dynamics Simulations S119
9. References S121

1. General Experimental Information

Unless otherwise specified, all starting materials, solvents and reagents were used as supplied and without further purification (Acros Organics, Alfa Aesar, Fisher Scientific, FluoroChem, Sigma Aldrich and VWR). All reactions were stirred with magnetic followers. Centrifugation of samples was carried out with a 5804/5804R Benchtop Centrifuge.

Low-resolution mass spectra were obtained on a Waters Xevo G2-S bench top QTOF with electrospray ionization (ESI) through direct injection. High-resolution ESI mass spectra were obtained on a Waters Synapt G2-Si mass spectrometer (cone voltage 10-30 eV; desolvation temp. 373 K; ionization temp. 333 K) infused from a Harvard syringe pump at a rate of 4-10 µL min^{-1}.

NMR spectra were recorded on a Bruker DRX-400, Bruker Avance 500 Cryo, Bruker 500 TCI-ATM Cryo and Bruker 700 TCI-ATM Cryo. Chemical shifts (δ) for NMR spectra are reported in parts per million (ppm) and are reported relative to the solvent residual peak. Coupling constants (*J*) are reported in hertz (Hz). The following abbreviations are used to describe signal multiplicity in ^{1}H, ^{13}C and ^{19}F NMR spectra: s: singlet, d: doublet, t: triplet, q: quartet, dd: doublet of doublets; dt: doublet of triplets; m: multiplet, br: broad. ^{109}Ag NMR are reported in ppm, measured at 23 MHz. DOSY NMR experiments were performed on a 400 MHz Avance III HD Smart Probe NMR spectrometer. Maximum gradient strength was 6.57 G/cm A. The standard Bruker pulse program, ledbpgp2s, employing a stimulated echo and longitudinal eddy-current delay (LED) using bipolar gradient pulses for diffusion using 2 spoil gradients was utilized for samples in CD_3CN. Rectangular gradients were used with a total duration of 1.5 ms. Gradient pulse durations were 850-1400 µs. ^{1}H-^{109}Ag heteronuclear single quantum coherence-exchange (HSQC-EX) NMR spectroscopy was adapted from ^{1}H-^{15}N chemical exchange pulse programme published by Farrow *et al.*[1]

X-ray crystallography data were collected at Beamline I19 of Diamond Light Source employing silicon double crystal monochromated synchrotron radiation (0.6889 Å) with ω and ψ scans at 100(2) K.[2] Data integration and reduction were undertaken with Xia2.[3] Subsequent computations were carried out using the Olex2 graphical user interface. Multi-scan empirical absorption corrections were applied to the data using the AIMLESS[4] tool in the CCP4 suite.[5] The structures were solved by direct methods using SHELXT[6] then refined and extended with SHELXL.[7] In general, non-hydrogen atoms with occupancies greater than 0.5 were refined anisotropically. Carbon-bound hydrogen atoms were included in idealized positions and refined using a riding model. Disorder was modeled using standard crystallographic methods including constraints, restraints, and rigid bodies where necessary. Crystallographic data along with specific details pertaining to the refinement follow. Crystallographic data have been deposited with the CCDC (**1-I** 2377078, **1-Cl** 2378429, **1-Br** 2377075).

Visible-light Raman spectra were recorded using an Invia Renishaw spectrometer (50) equipped with 785 nm laser. Ultraviolet (UV) Resonance Raman spectra were measured at the BL 10.2-IUVS beamline of the Elettra Trieste (Italy) using an excitation wavelength at 266 nm, grating 1008, and resolution 1.82 cm^{-1}•pixel provided by a passively Q-switched laser system. The Raman signal was collected in back-scattering geometry and analysed through a 75 cm spectrograph (Trivista 557, Princeton Instruments) equipped with holographic gratings of 1800 and 3600 grooves/mm and a UV/enhanced CCD camera for detection.

2. Synthesis of Subcomponents

2.1 Synthesis of **S1**

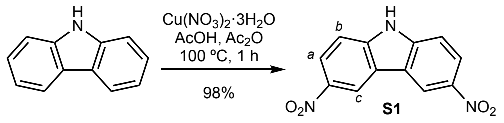

S1 was synthesized through a modified literature procedure.[8]

Copper(II) nitrate-trihydrate (7.98 g, 33 mmol, 1.2 eq) was dissolved in acetic acid (15 mL) and acetic anhydride (38 mL) and stirred at room temperature for 20 min. Carbazole (4.50 g, 27 mmol, 1.0 eq.) was added portion-wise over 5 min, followed by acetic acid (15 mL). The reaction was heated at 100 ºC for 1 h. The reaction was cooled room temperature, and poured into deionized water (300 mL). The yellow precipitate was collected and washed with deionized water (3 × 150 mL). The yellow precipitate was added to a solution of potassium hydroxide (37.5 g) in deionized water (375 mL) and EtOH (375 mL), and the resulting red solution stirred at room temperature for 30 min. The red solution was filtered, and the filtrate acidified with concentrated HCl (20 mL) causing a yellow precipitate to form. The yellow precipitate collected by vacuum filtration, and washed with deionized water (500 mL) and EtOH (100 mL). The yellow precipitate was then suspended in acetone (500 mL), and the solvent was removed *in vacuo* yielding 3,6-dinitro-9*H*-carbazole, **S1**, as a yellow solid (6.32 g, 24.6 mmol, 91%). Characterization by ^{1}H NMR and MS match previously reported data.[8]

δ$_H$ (d_6-DMSO, 500 MHz) 12.78 (1H, br. s, N***H***), 9.51 (2H, d, *J* = 2.3, ***H***$_c$), 8.40 (2H, dd, *J* = 9.0, 2.3, ***H***$_a$), 7.78 (2H, d, *J* = 9.0, ***H***$_b$). LR-ESI-MS [NEG] *m/z* 256.1 [M-H]$^-$.

2.2 Synthesis of **A**

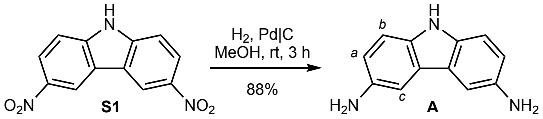

A flask was charged with 10 wt.% Palladium on carbon (310 mg, 0.292 mmol, 0.05 eq.) and washed down the walls of the flask with MeOH (10 mL). 3,6-dinitrocarbazole (1.50 g, 5.83 mmol, 1.0 eq.) was added in MeOH (100 mL). A balloon of N_2 was bubbled through the solution, followed by a balloon of H_2. The reaction was stirred at room temperature for 3 h under H_2 and conversion monitored by LCMS. The reaction was filtered through Celite® and concentrated *in vacuo*, yielding 3,6-diamino-9*H*-carbazole, **A**, a black solid (1.01 g, 5.12 mmol, 88%). Characterization by ^{1}H NMR and MS match previously reported data.[9]

δ$_H$ (d_6-DMSO, 500 MHz) 10.15 (1H, br. s, N***H***), 7.08-7.05 (4H, m, ***H***$_b$, ***H***$_c$), 6.68 (2H, dd, *J* = 8.5, 2.1, ***H***$_a$), 4.74 (4H, br. s, N***H***$_2$). LR-ESI-MS [POS] *m/z* 198.2 [M+H]$^+$.

2.3 Synthesis of **B**

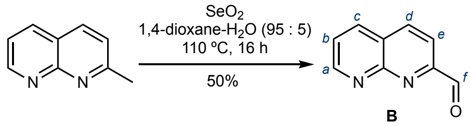

2-methyl-1,8-naphthyridine (2.00 g, 13.87 mmol, 1.0 eq.) and SeO_2 (1.69 g, 15.26 mmol, 1.1 eq.) were heated in 1,4-dioxane (330 mL) and water (20 mL) at 110 ºC for 16 h. The reaction was cooled to room temperature, concentrated *in vacuo*, filtered through a silica flashpad (SiO_2, CH_2Cl_2-MeOH 95 : 5). The residue was purified by column chromatography (SiO_2, petrol-EtOAc 50→100%) then concentrated *in vacuo* again yielding 2-formyl-1,8-naphthyridine, **B**, as a pale yellow solid (1.10 g, 6.95 mmol, 50%).

δ$_H$ ($CDCl_3$, 700 MHz) 10.33 (1H, d, *J* = 0.8, ***H***$_f$), 9.28 (1H, dd, *J* = 4.1, 1.9, ***H***$_a$), 8.40 (1H, d, *J* = 8.2, ***H***$_d$), 8.31 (1H, dd, *J* = 8.2, 1.9, ***H***$_c$), 8.15 (1H, d, *J* = 8.2, ***H***$_e$), 7.64 (1H, dd, *J* = 8.2, 4.1, ***H***$_b$). **δ**$_C$ ($CDCl_3$, 176 MHz) 193.8 (***C***$_f$), 155.9, 155.2 (***C***$_a$), 155.1, 139.0 (***C***$_d$), 137.2 (***C***$_c$), 125.4, 124.3 (***C***$_b$), 118.5 (***C***$_e$). LR-ESI-MS [POS] *m/z* 159.1 [M+H]$^+$ Characterization by ^{1}H and ^{13}C NMR match previously reported literature.[10]

3. Synthesis and characterization of $[Ag_{12}X_4L_6](NTf_2)_8$ grids

3.1 Synthesis and characterization of **1-I**

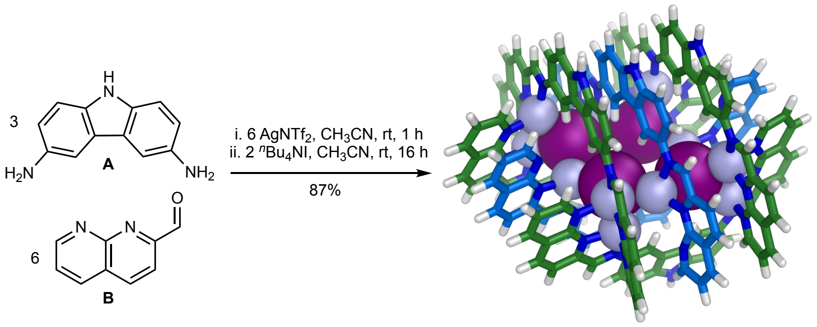

$[Ag^{I}_{12}I_4L_6](NTf_2)_8$

3,6-diamino-9*H*-carbazole **A** (1.97 mg, 10.0 µmol, 3.0 eq.), 2-formyl-1,8-naphthyridine **B** (3.16 mg, 20.0 µmol, 6.0 eq.) and $AgNTf_2$ (7.75 mg, 20.0 µmol, 6.0 eq.) were stirred in CH_3CN (0.5 mL) at rt for 1 h. $^nBu_4NI$ (2.46 mg, 6.67 µmol, 2.0 eq.) in CH_3CN (0.5 mL) was added and the reaction stirred at rt for 16 h. The reaction was concentrated under N_2, washed with Et_2O (3 × 10 mL), dissolved in CH_3CN, filtered and concentrated *in vacuo* yielding a dark red solid **1-I** (9.5 mg, 1.45 µmol, 87%, 6892.8 $gmol^{-1}$).

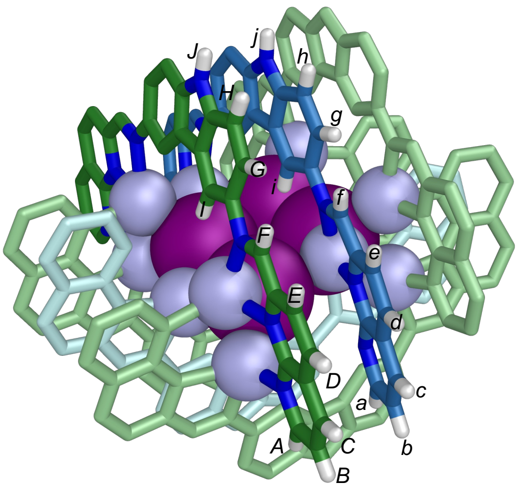

δ_H (CD_3CN, 700 MHz) 10.12 (2H, s, ***H***$_J$), 9.45 (1H, s, ***H***$_j$), 8.83 (4H, s, ***H***$_F$), 8.65 (2H, d, *J* = 1.5, ***H***$_i$), 8.61 (4H, dd, *J* = 1.7, ***H***$_I$), 8.18 (2H, dd, *J* = 4.9, 1.7, ***H***$_a$), 8.14 (4H, d, *J* = 8.1, ***H***$_D$), 7.95 (2H, s, ***H***$_f$), 7.82 (4H, br. dd, *J* = 8.7, 1.7, ***H***$_G$), 7.79 (4H, d, *J* = 8.0, ***H***$_E$), 7.65 (4H, d, *J* = 8.6, ***H***$_H$), 7.63 (4H, br. d, *J* = 7.4, ***H***$_C$), 7.54 (4H, dd, *J* = 5.0, 1.6, ***H***$_A$), 7.33 (2H, d, *J* = 8.0, ***H***$_d$), 7.19-7.16 (4H, m, ***H***$_e$, ***H***$_h$), 7.09 (2H, br. d, *J* = 8.6, ***H***$_g$), 6.91 (2H, d, *J* = 7.5, ***H***$_c$), 6.46 (4H, dd, *J* = 7.5, 5.1, ***H***$_B$), 6.43 (2H, dd, *J* = 7.6, 4.8, ***H***$_b$).

δ_C (CD_3CN, 176 MHz) 157.5 (***C***$_A$), 156.5 (***C***$_a$), 154.2, 153.6 (***C***$_F$), 151.6, 151.2, 151.0, 149.4 (***C***$_f$), 142.4, 141.9 (***C***$_D$), 141.3, 140.9 (***C***$_d$), 140.1 (***C***$_C$), 139.0, 138.9, 137.1 (***C***$_c$), 129.9, 127.1 (***C***$_E$), 125.5, 124.9 (***C***$_e$), 124.2 (***C***$_I$), 124.0 (***C***$_b$), 123.9, 123.8 (***C***$_B$), 123.8(***C***$_i$), 120.9 (q, $^1J_{CF}$ = 320.5, ***C***F_3), 116.6 (***C***$_G$), 115.5 (***C***$_g$), 114.2 (***C***$_H$), 114.0, 113.8 (***C***$_h$).

δ_F (CD_3CN, 376 MHz) -80.1 ($N(SO_2C$***F***$_3)_2$).

δ_{Ag} (CD_3CN, 23 MHz) 788 ($8Ag_{vertex}$), 696 ($4Ag_{edge}$).

DOSY D = 5.06 × 10^{-10} m^2s^{-1}, log(D) = -9.30, r = 12.9 Å.

LR-ESI-MS [POS] $[Ag_{12}I_4L_6](NTf_2)_8$ *m*/*z* 3174.0 $[M\text{-}2NTf_2]^{2+}$, 2022.6 $[M\text{-}3NTf_2]^{3+}$, 1446.8 $[M\text{-}4NTf_2]^{4+}$. Fragmentation into $[Ag_6I_2L_3](NTf_2)_4$ contributes to the peaks at *m*/*z* 3174.0 $[M\text{-}NTf_2]^{1+}$, and 1446.8 $[M\text{-}2NTf_2]^{2+}$.

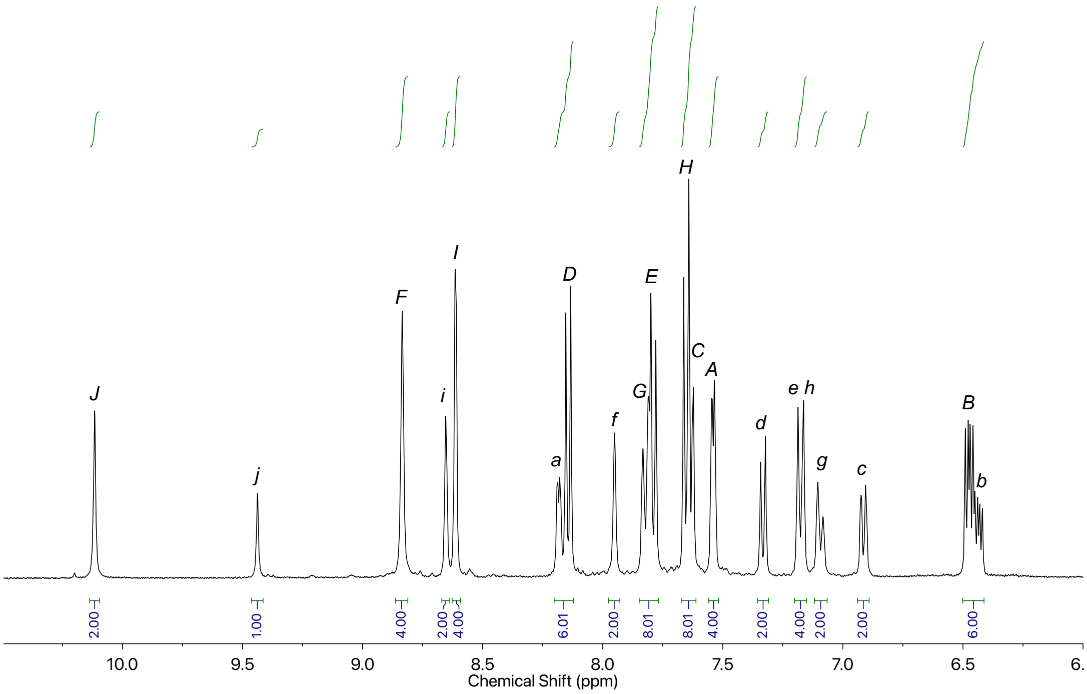

Figure S1. ^{1}H NMR of **1-I** (CD_3CN, 700 MHz, 298 K).

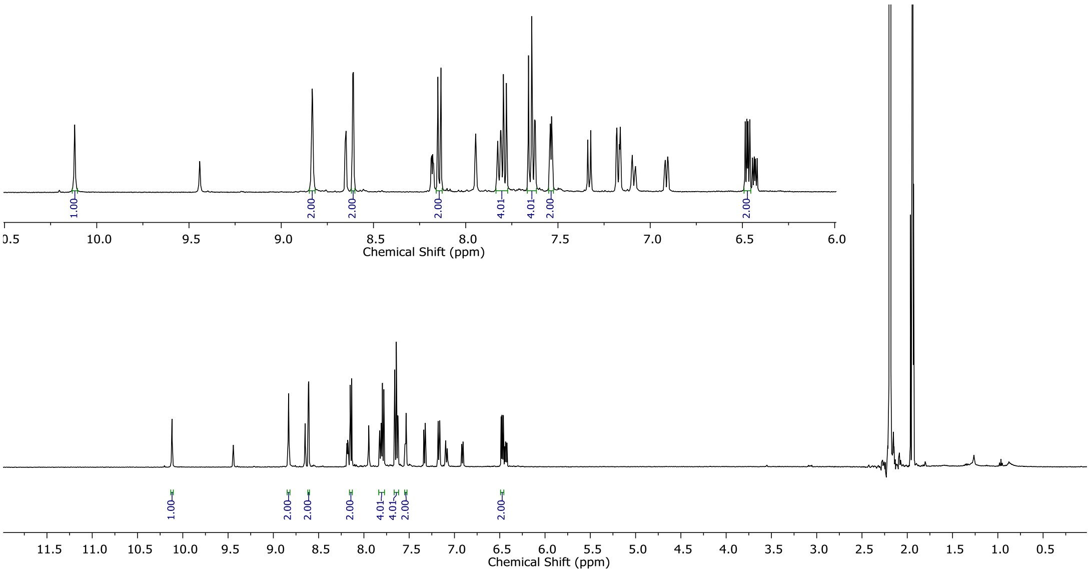

Figure S2. ^{1}H NMR showing the outer (green) arm of **1-I** (CD_3CN, 700 MHz, 298 K).

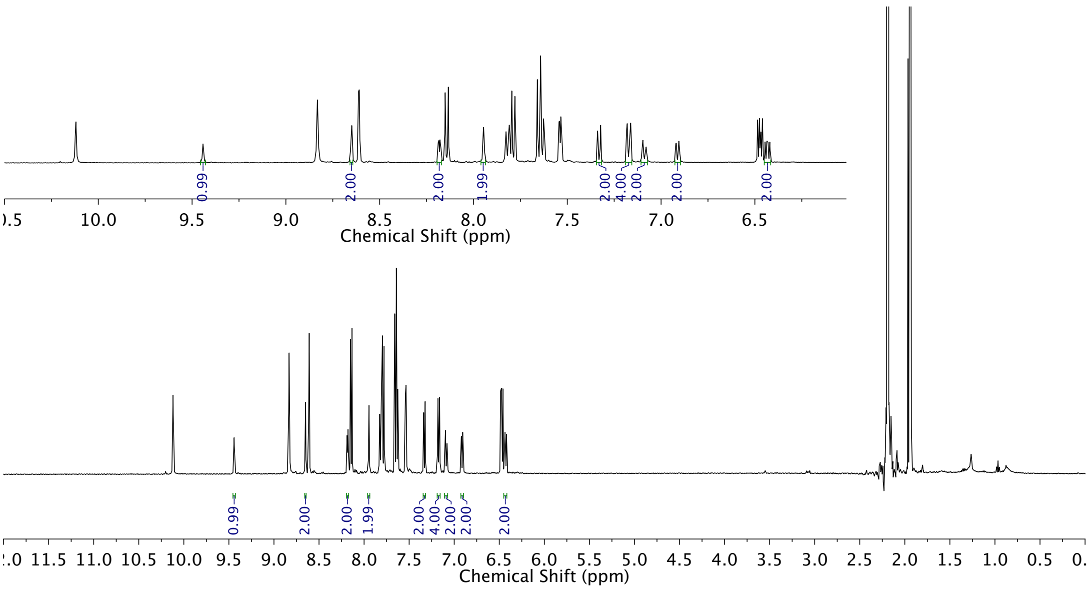

Figure S3. ¹H NMR showing the inner (blue) arm of **1-I** (CD_3CN, 700 MHz, 298 K).

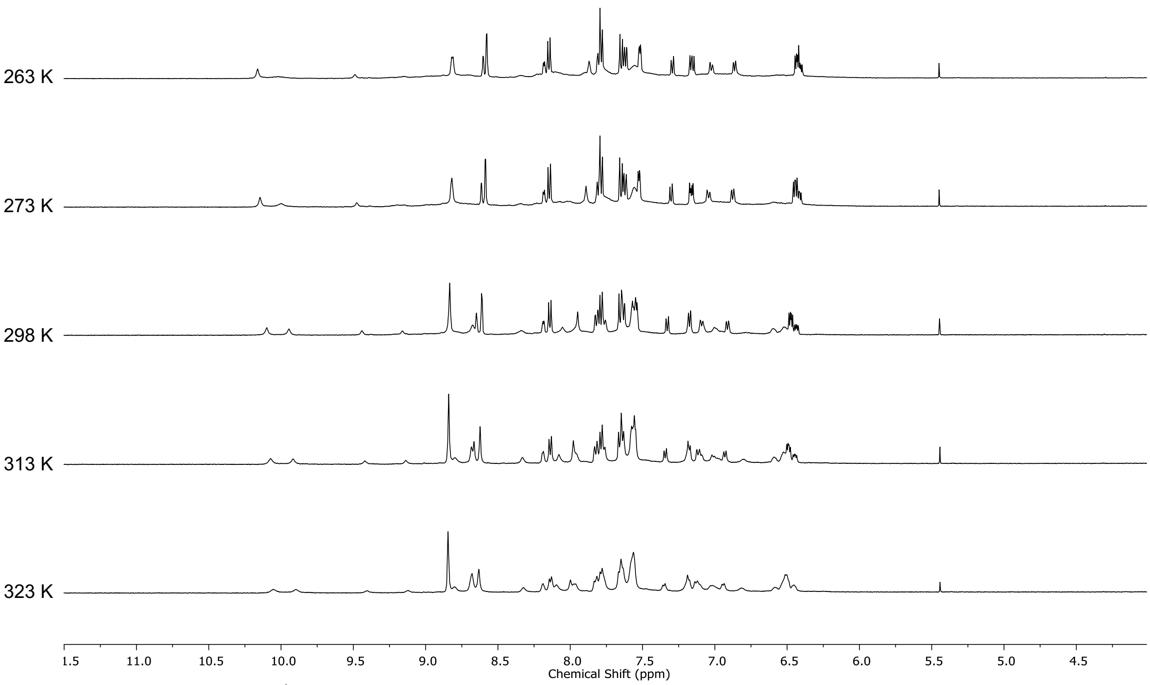

Figure S4. Variable temperature ¹H NMR of **1-I** (CD_3CN, 500 MHz).

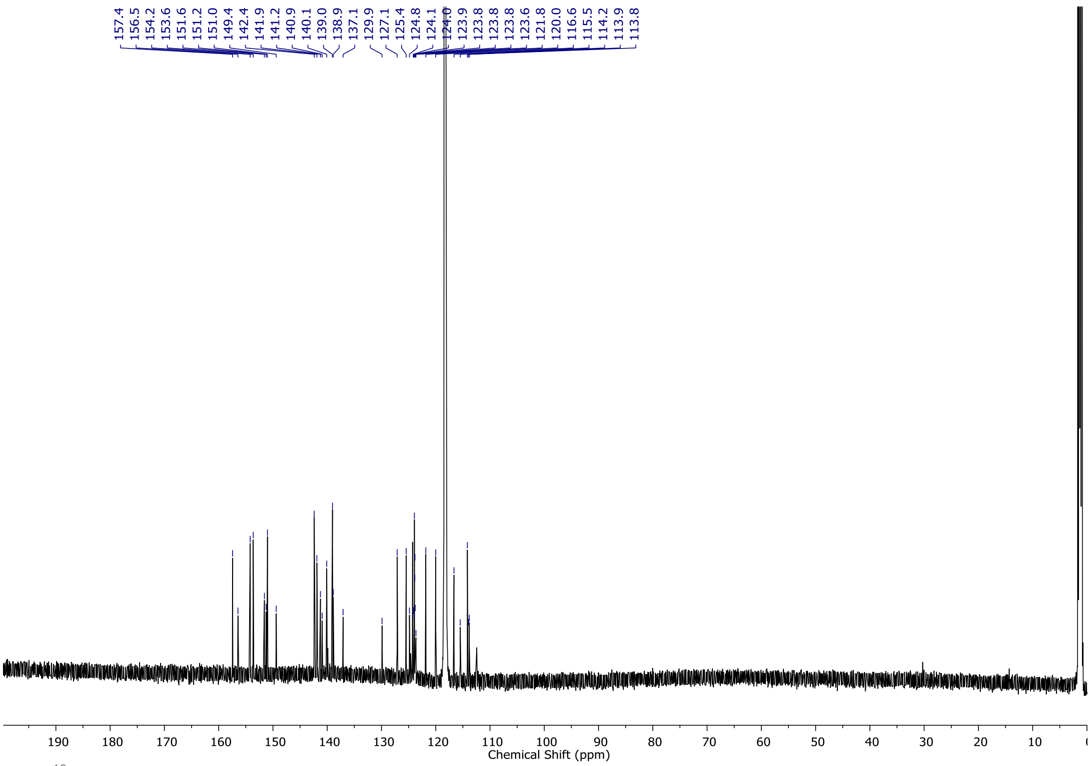

Figure S5. ^{13}C NMR of **1-I** (CD_3CN, 176 MHz, 298 K).

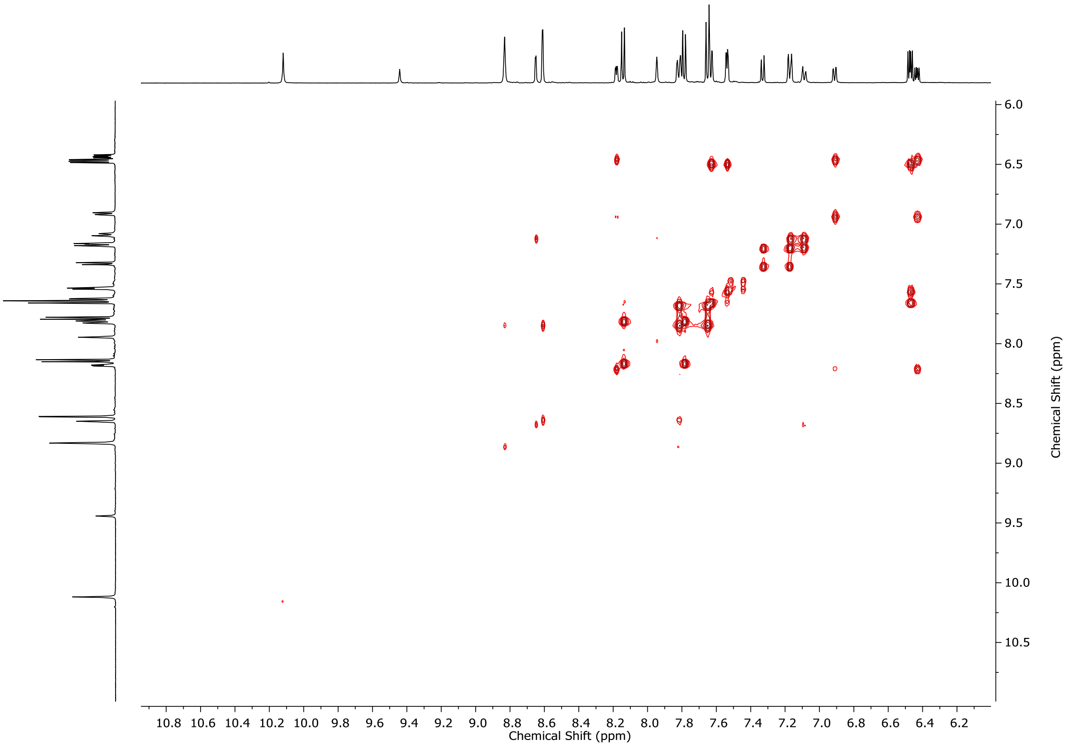

Figure S6. ^{1}H-^{1}H COSY NMR of **1-I** (CD_3CN, 298 K).

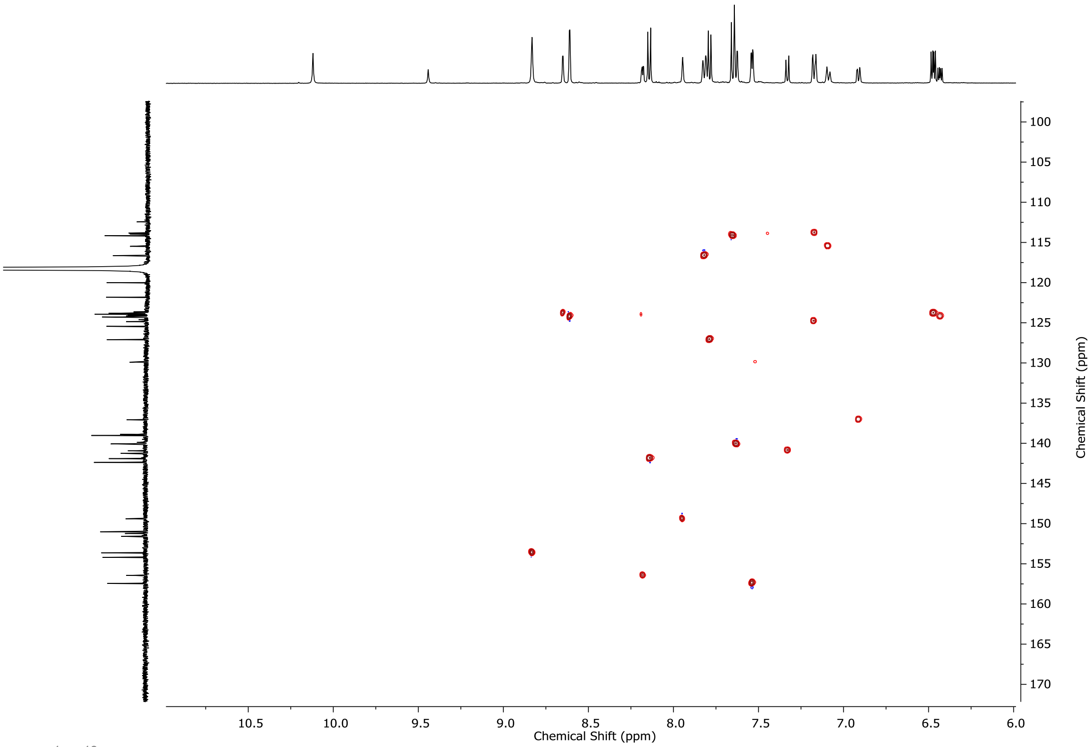

Figure S7. ^{1}H-^{13}C HSQC NMR of **1-I** (CD_3CN, 298 K).

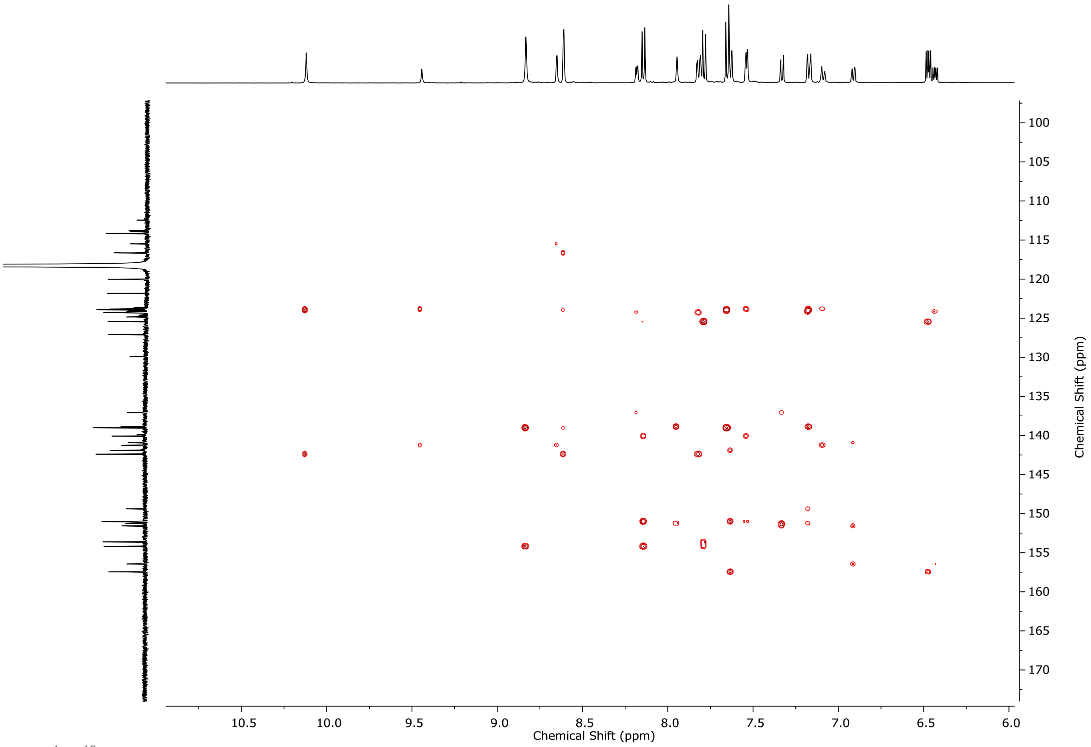

Figure S8. ^{1}H-^{13}C HMBC NMR of **1-I** (CD_3CN, 298 K).

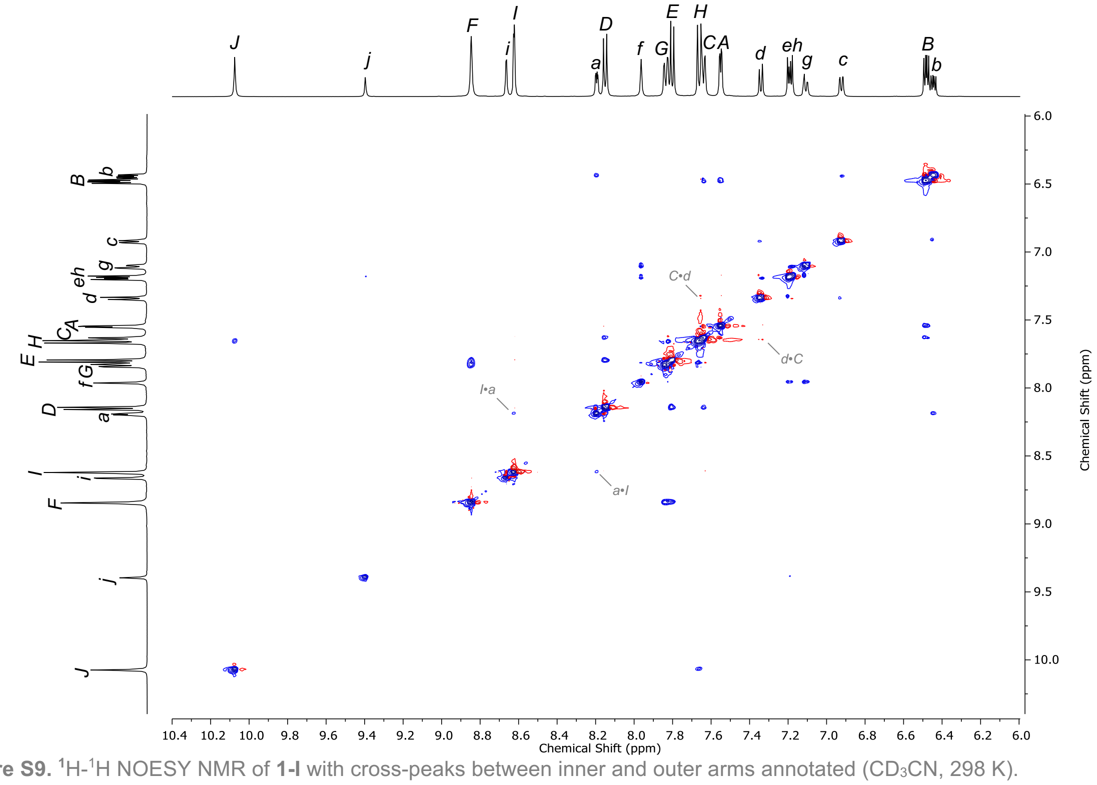

Figure S9. ^1H-^1H NOESY NMR of **1-I** with cross-peaks between inner and outer arms annotated (CD_3CN, 298 K).

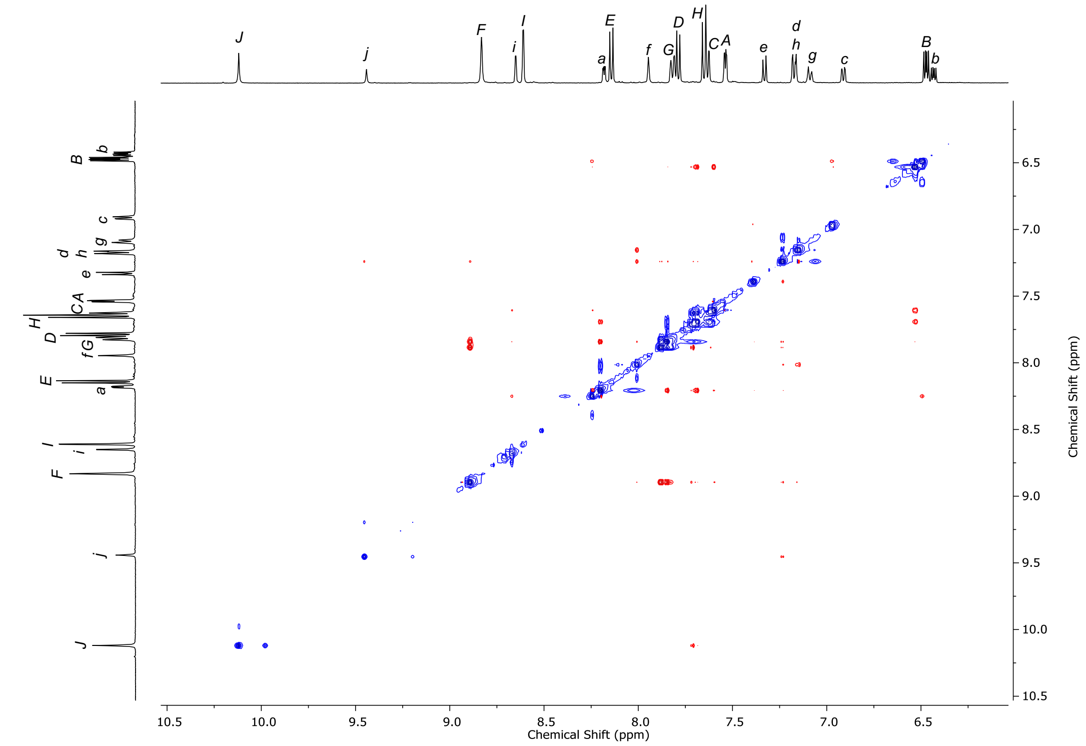

Figure S10. ^1H-^1H ROESY NMR of **1-I** with cross-peaks between inner and outer arms annotated (CD_3CN, 298 K).

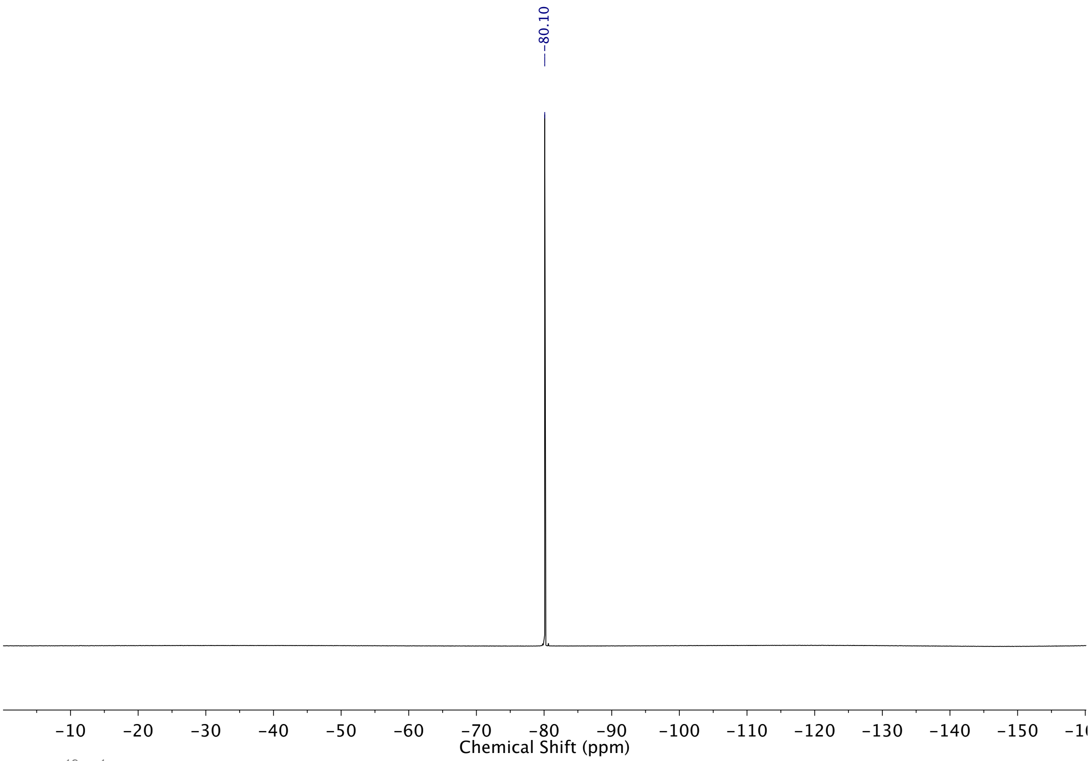

Figure S11. $^{19}F\{^{1}H\}$ NMR of **1-I** (CD_3CN, 376 MHz, 298 K).

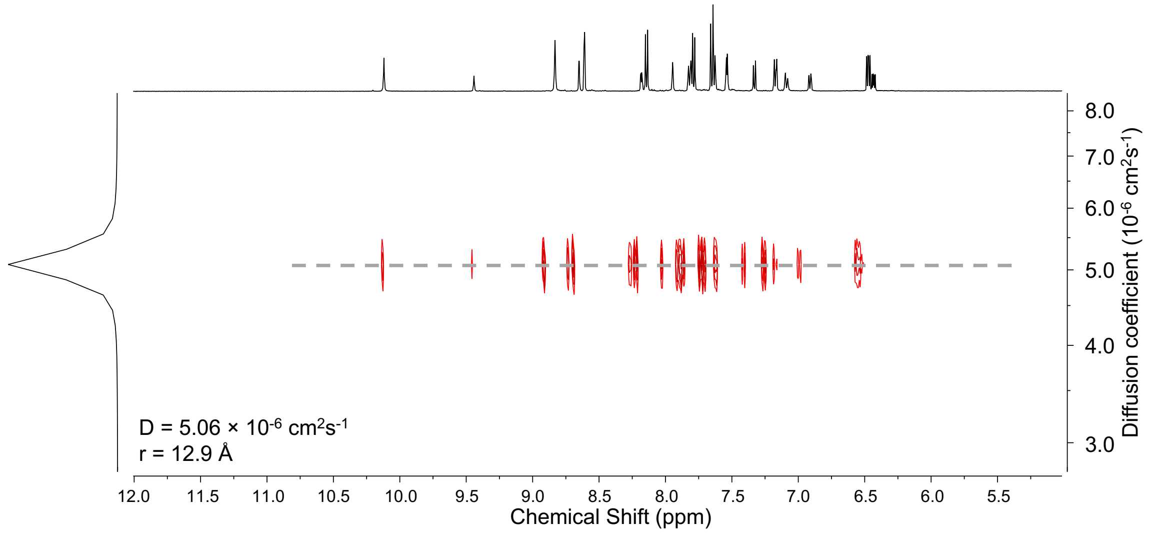

Figure S12. ^{1}H DOSY NMR of **1-I** (CD_3CN, 400 MHz, 298 K).

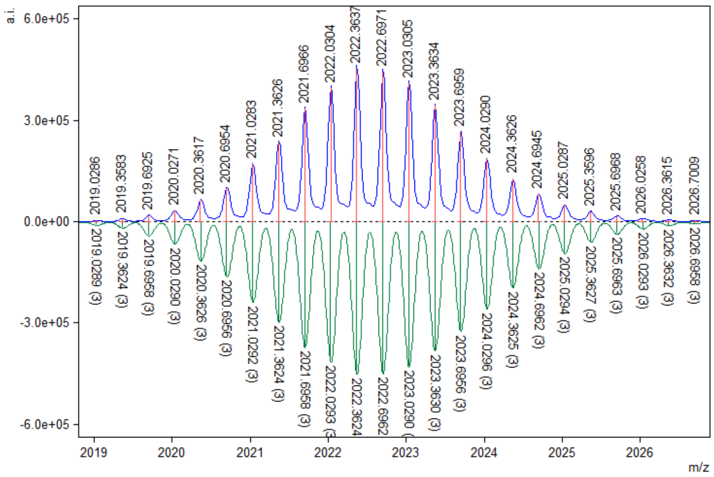

Figure S13. HR-ESI-MS [POS] **1-I** $[Ag_{12}I_4L_6](NTf_2)_8$ $[M-3NTf_2]^{3+}$ ($\delta_{expt-calc}$ 0.64 ppm).

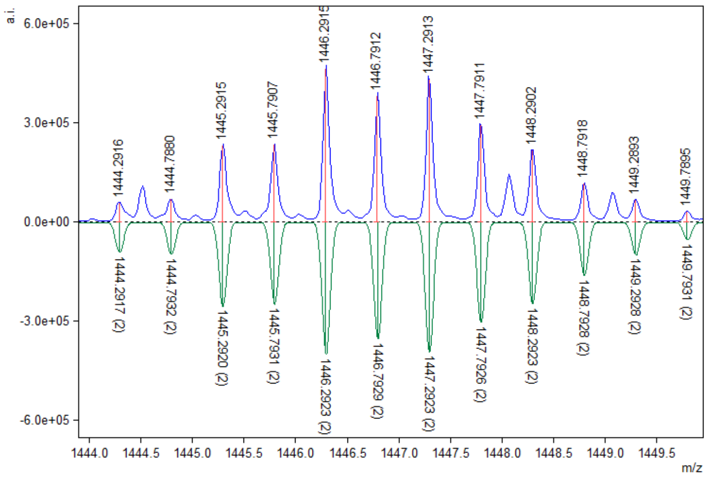

Figure S14. HR-ESI-MS [POS] **1-I** $[Ag_6I_2L_3](NTf_2)_4$ $[M-2NTf_2]^{2+}$ ($\delta_{expt-calc}$ 0.55 ppm).

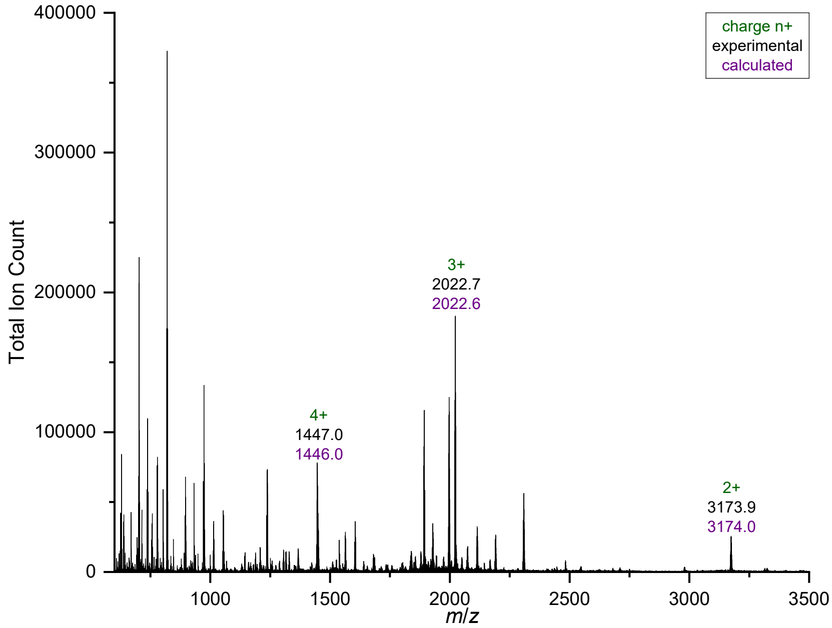

Figure S15. LR-ESI-MS [POS] **1-I** $[Ag_{12}I_4L_6](NTf_2)_8$ *m/z* 3174.0 $[M-2NTf_2]^{2+}$, 2017.7 $[M-3NTf_2]^{3+}$, 1446.9 $[M-4NTf_2]^{4+}$. Peaks at 1447.0 and 3173.9 contain contributions from $[Ag_6I_2L_3](NTf_2)_4$ fragment.

3.2 ^{109}Ag NMR characterization of **1-I**

Initial 1D ^{109}Ag (Figure S16) and ^{1}H-^{109}Ag HMBC spectra (Figure S17) of **1-I** at 298 K showed one low intensity environment at 788 ppm (with low signal-to-noise ratio), with correlations to both sets of ^{1}H signals. Indeed the ^{109}Ag decoupled 1H NMR spectra (Figure S18-Figure S19) irradiated at 788 ppm removed ^{109}Ag couplings to imine signals corresponding to both ligand environments.

Cooling **1-I** to 232 K revealed two sharp ^{109}Ag environments at 805 and 706 ppm (Figure S20) which showed distinct correlations to the two sets of ligand signals. The environment at 805 ppm corresponded to the 8 Ag atoms in the vertex environment with correlations to the outer ligand ^{1}H environment; the environment at 706 ppm corresponded to the 4 Ag atoms at the edge environment, with correlations to the inner ligand ^{1}H environment.

Variable temperature ^{1}H-^{109}Ag HMBC spectra taken at 232, 252, 272, 285 and 298 K (Figure S20-Figure S24) revealed at at 232 K the two ^{109}Ag environments correlate to separate ^{1}H environments however, above 252 K both environments correlate to both ^{1}H environments, with a decreasing population of the lower shift environment. The mixing of correlation environments observed at 252 K implies exchange of ^{109}Ag environments on a faster timescale than the ^{1}H environments. The ^{109}Ag NMR spectroscopy shows the movement of the Ag cluster inside the organic host.

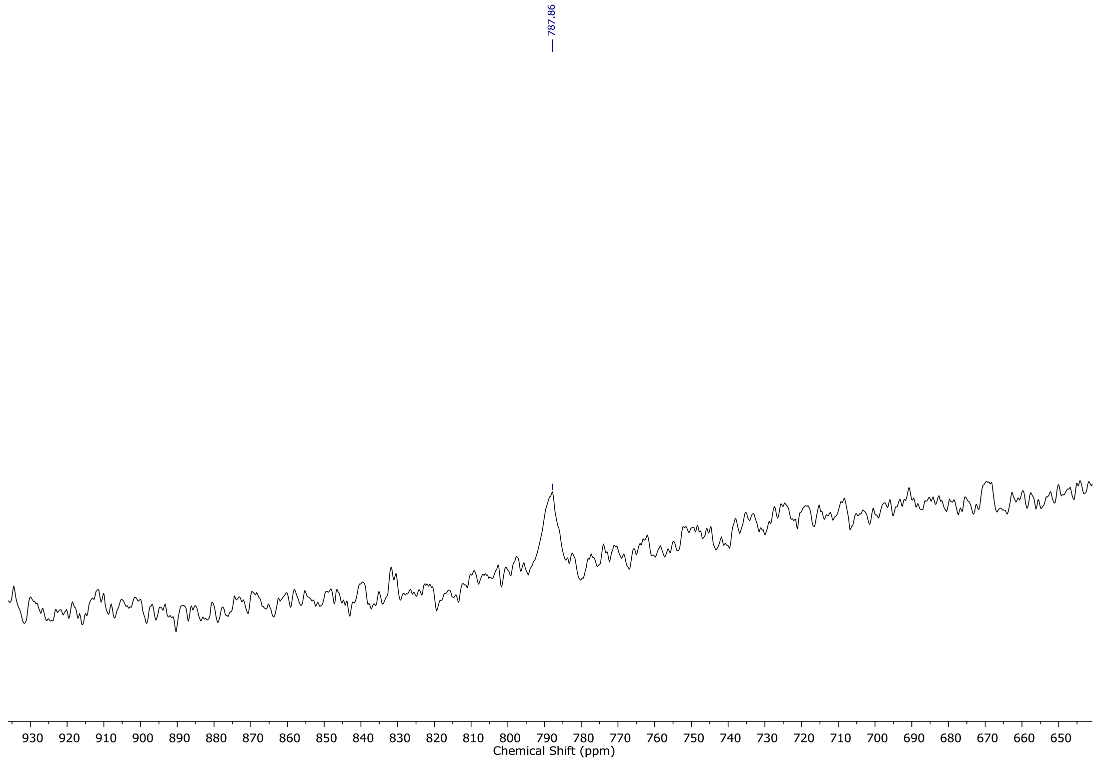

Figure S16. ^{109}Ag NMR of **1-I** (CD_3CN, 23 MHz, 298 K).

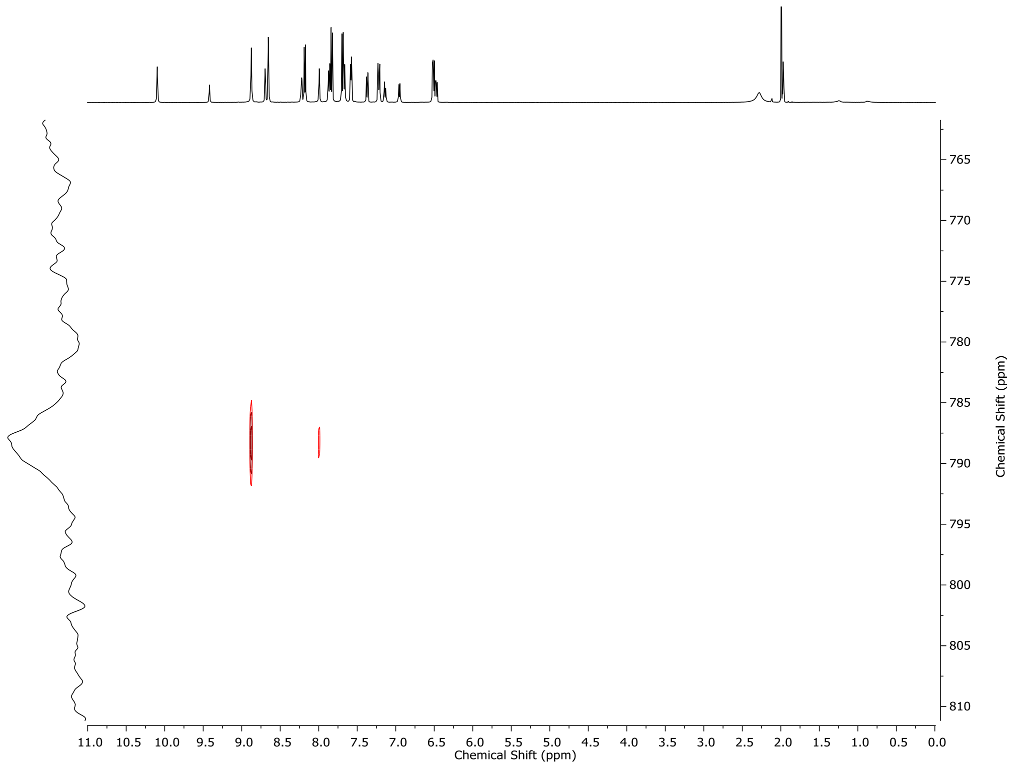

Figure S17. ^{1}H-^{109}Ag HMBC NMR of **1-I** (CD_3CN, 298 K).

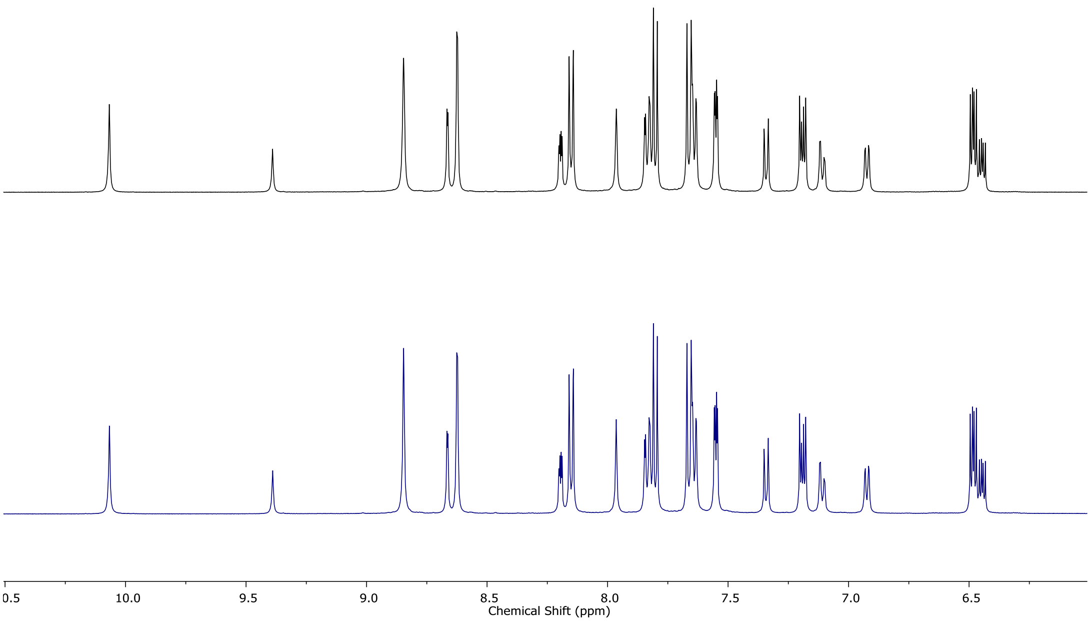

Figure S18. Stackplot of standard ^{1}H NMR (top, black) and ^{1}H NMR with ^{109}Ag decoupling at 788 ppm (bottom, blue) of **1-I** (CD_3CN, 500 MHz, 298 K).

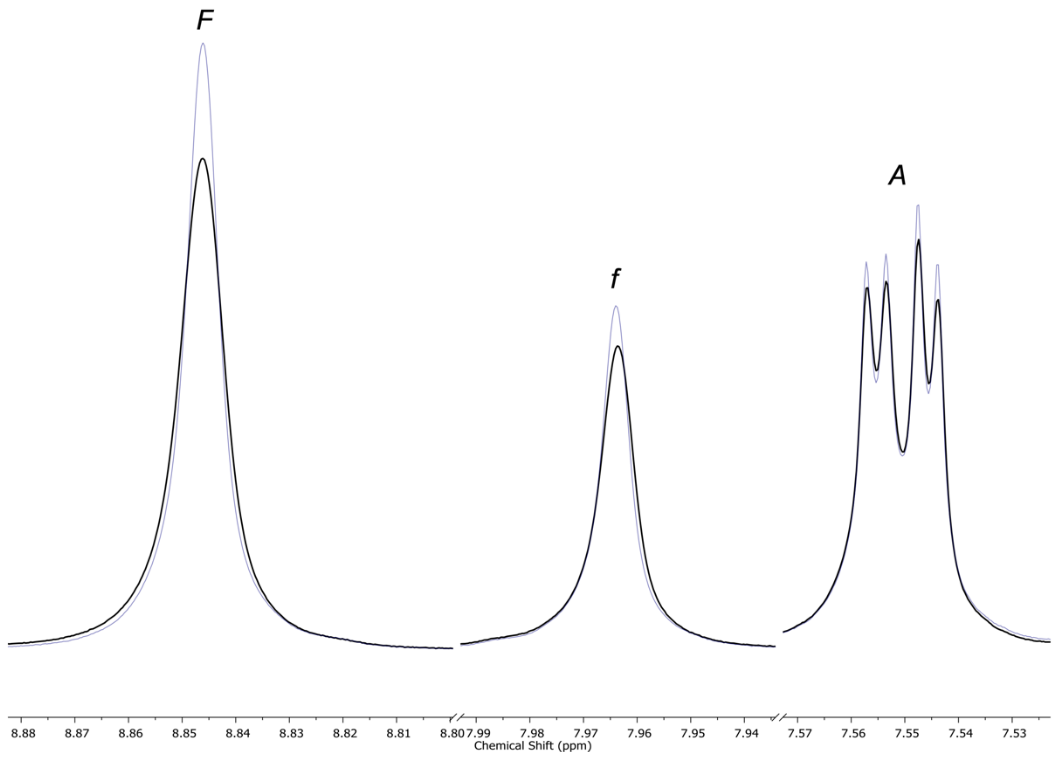

Figure S19. Superposition of standard ^1H NMR (black) and ^1H NMR with ^{109}Ag decoupling at 788 ppm (blue) of **1-I** (CD_3CN, 500 MHz, 298 K).

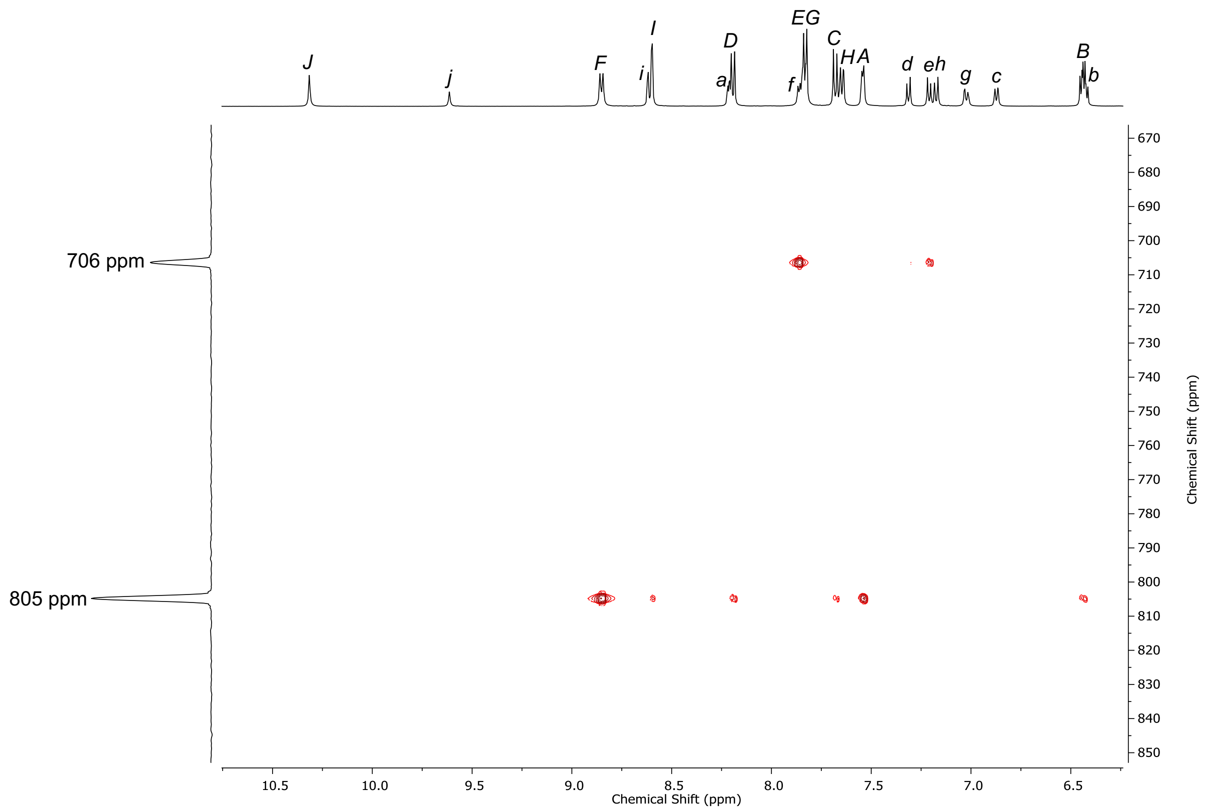

Figure S20. Enlarged ^1H-^{109}Ag HMBC NMR of **1-I** (CD_3CN, 232 K).

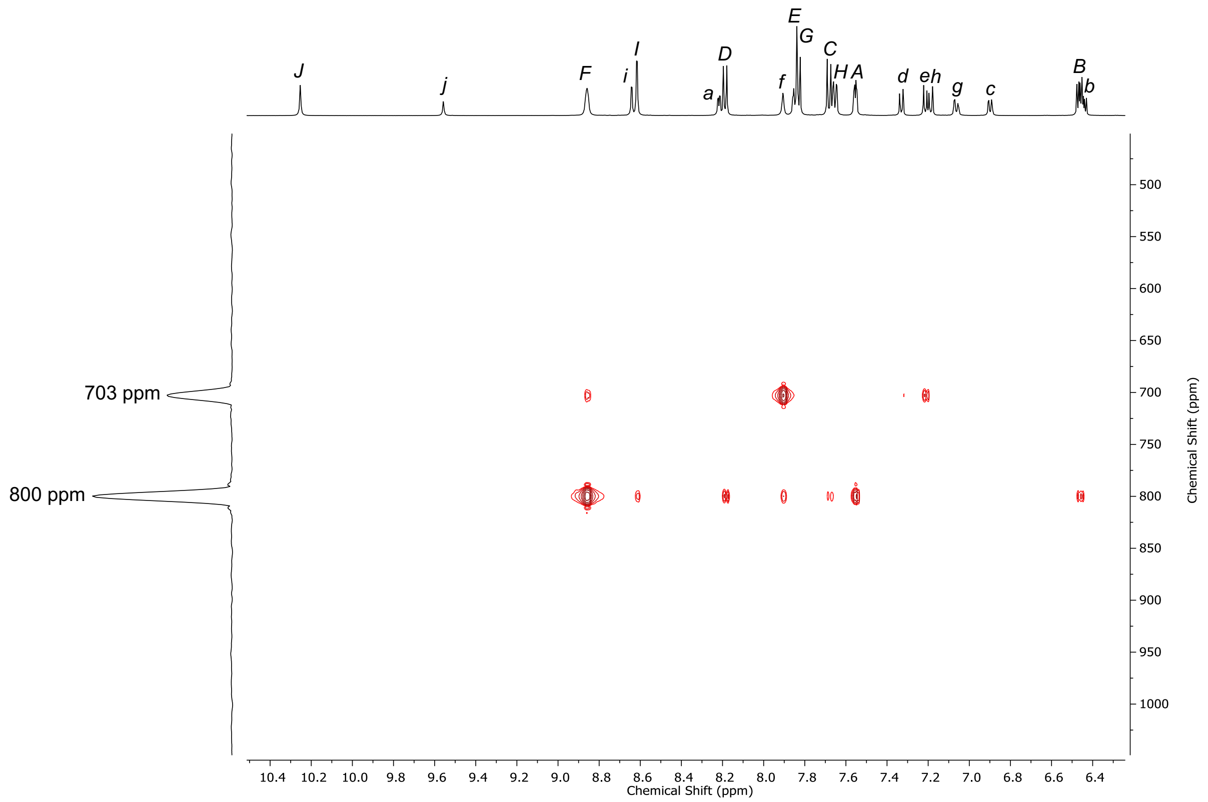

Figure S21. Enlarged ^{1}H-^{109}Ag HMBC NMR of **1-I** (CD_3CN, 252 K).

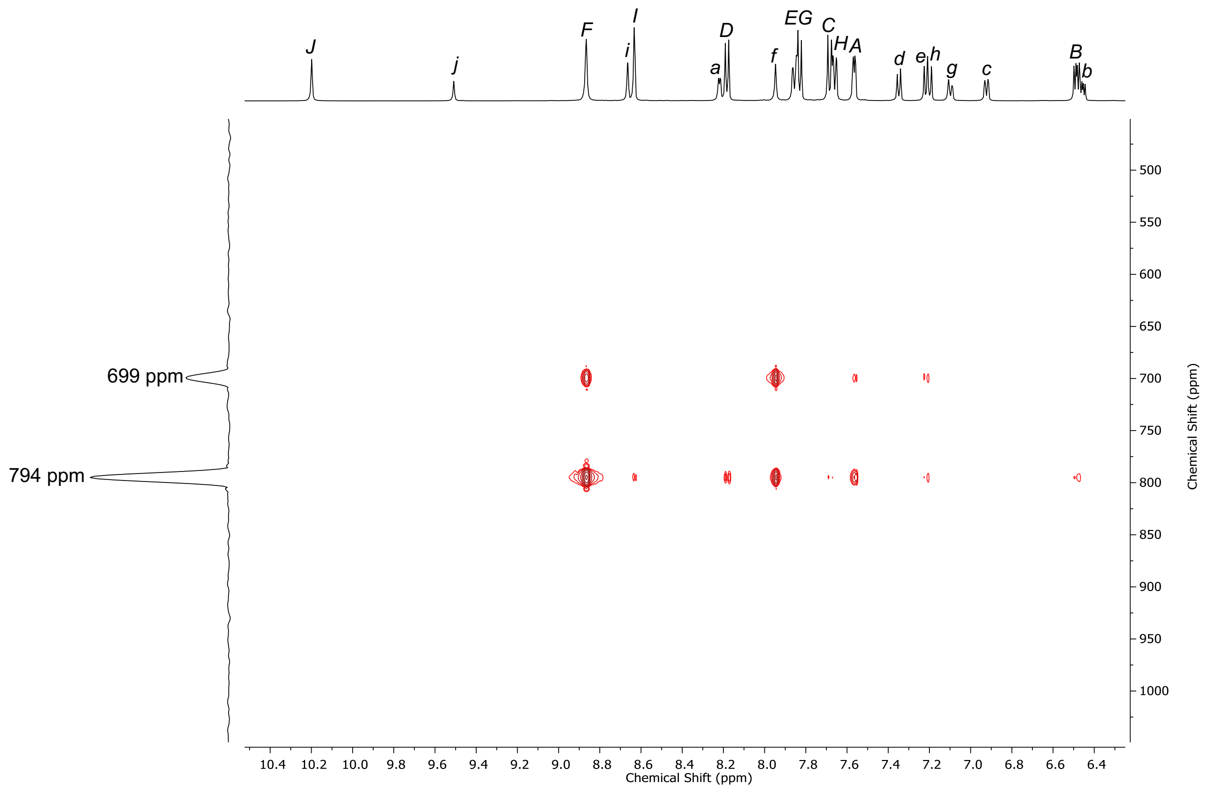

Figure S22. Enlarged ^{1}H-^{109}Ag HMBC NMR of **1-I** (CD_3CN, 272 K).

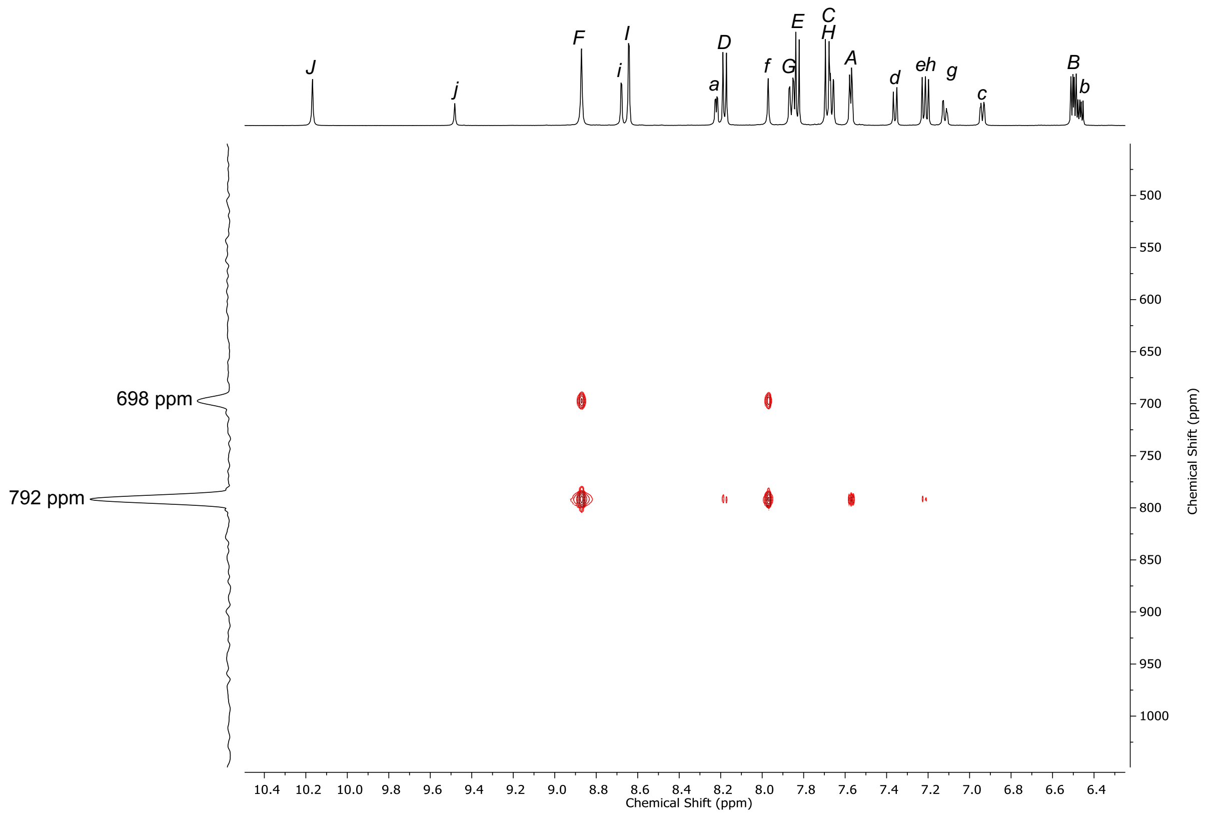

Figure S23. Enlarged ^{1}H-^{109}Ag HMBC NMR of **1-I** (CD_3CN, 285 K).

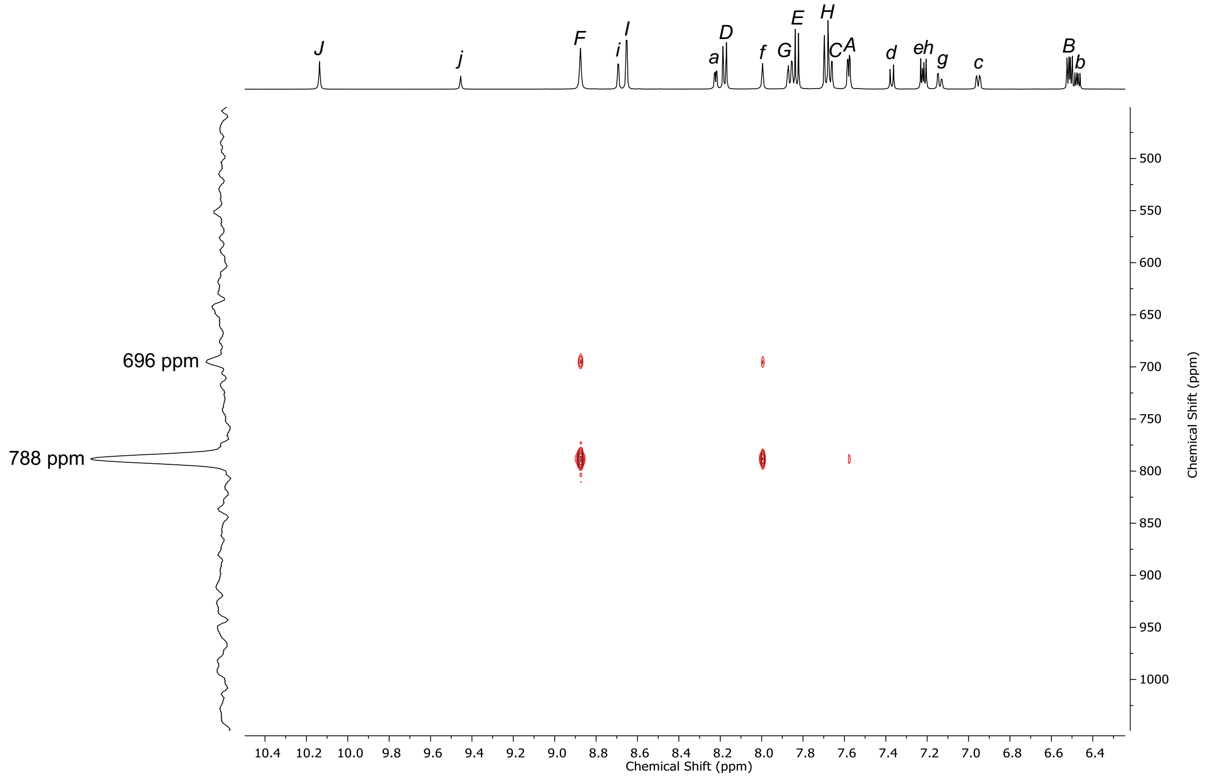

Figure S24. Enlarged ^{1}H-^{109}Ag HMBC NMR of **1-I** (CD_3CN, 298 K).

3.3 Synthesis and characterization of **1-Cl**

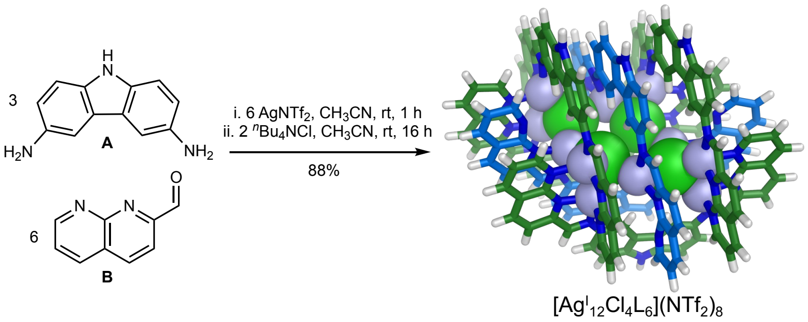

3,6-diamino-9*H*-carbazole **A** (3.94 mg, 20.0 µmol, 3.0 eq.), 2-formyl-1,8-naphthyridine **B** (6.32 mg, 40.0 µmol, 6.0 eq.) and $AgNTf_2$ (15.52 mg, 40.0 µmol, 6.0 eq.) were stirred in CH_3CN (1.5 mL) at rt for 1 h. $^nBu_4NCl$ (3.71 mg, 13.3 µmol, 2.0 eq.) in CH_3CN (0.5 mL) was added and the reaction stirred at rt for 16 h. The reaction was concentrated under N_2, washed with Et_2O (3 × 10 mL), dissolved in CH_3CN, filtered and concentrated *in vacuo* yielding a dark red solid **1-Cl** (19.1 mg, 2.92 µmol, 88%, 6542.5 $gmol^{-1}$).

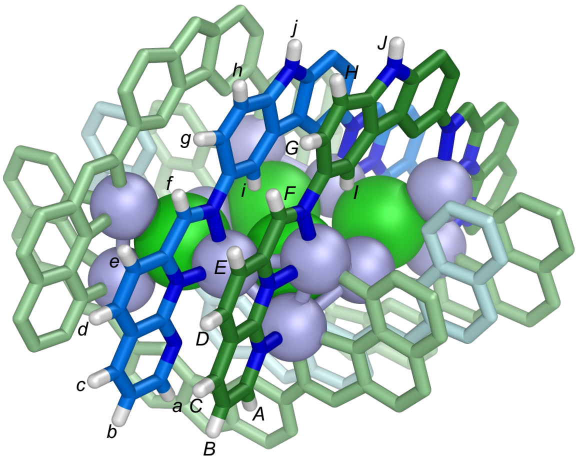

δ_H (CD_3CN, 500 MHz) 10.19 (2H, s, $\boldsymbol{H}_J$), 9.01 (1H, s, $\boldsymbol{H}_j$), 8.99 (2H, d, J = 2.0, $\boldsymbol{H}_i$), 8.94 (4H, d, J_{HAg} = 8.4, $\boldsymbol{H}_F$), 8.50 (4H, d, J = 2.1, $\boldsymbol{H}_I$), 8.13 (2H, dd, J = 4.6, 1.6, $\boldsymbol{H}_a$), 8.11 (4H, d, J = 8.2, $\boldsymbol{H}_D$), 8.06 (2H, d, J_{HAg} = 5.3, $\boldsymbol{H}_f$), 7.96 (4H, dd, J = 9.1, 2.1, $\boldsymbol{H}_G$), 7.81 (4H, d, J = 8.2, $\boldsymbol{H}_E$), 7.74 (4H, d, J = 9.0, $\boldsymbol{H}_H$), 7.57 (4H, dd, J = 8.0, 1.7, $\boldsymbol{H}_C$), 7.23 (4H, dd, J = 4.9, 1.7, $\boldsymbol{H}_A$), 7.01 (2H, dd, J = 9.0, 2.0, $\boldsymbol{H}_g$), 7.00 (1H, d, J = 8.1, $\boldsymbol{H}_d$), 6.95 (2H, dd, J = 7.7, 8.1, 1.6, $\boldsymbol{H}_c$), 6.85 (2H, d, J = 8.9, $\boldsymbol{H}_h$), 6.82 (2H, d, J = 8.0, $\boldsymbol{H}_e$), 6.60 (2H, dd, J = 7.7, 4.6, $\boldsymbol{H}_b$), 6.34 (4H, dd, J = 8.0, 4.9, $\boldsymbol{H}_B$).

δ_C (CD_3CN, 176 MHz) 156.2 ($\boldsymbol{C}_A$), 155.8 ($\boldsymbol{C}_a$), 153.1, 151.8 ($\boldsymbol{C}_F$), 150.8, 150.7, 149.6, 147.4 ($\boldsymbol{C}_f$), 141.7, 141.1 ($\boldsymbol{C}_D$), 139.9, 139.2 ($\boldsymbol{C}_d$), 139.0 ($\boldsymbol{C}_C$), 137.9, 137.4, 136.8 ($\boldsymbol{C}_c$), 126.0 ($\boldsymbol{C}_E$), 124.4, 124.2 ($\boldsymbol{C}_I$), 123.9 ($\boldsymbol{C}_e$), 123.9 ($\boldsymbol{C}_b$), 123.5, 123.4 ($\boldsymbol{C}_i$), 123.1, 122.9, 122.7 ($\boldsymbol{C}_B$), 120.0 (q, $^1J_{CF}$ = 321.0, $\boldsymbol{C}F_3$), 115.9 ($\boldsymbol{C}_G$), 114.0 ($\boldsymbol{C}_g$), 113.0 ($\boldsymbol{C}_H$), 112.8 ($\boldsymbol{C}_h$).

δ_F (CD_3CN, 376 MHz) -80.0 ($N(SO_2C\boldsymbol{F}_3)_2$).

δ_{Ag} (CD_3CN, 23 MHz) 632 ($8Ag_{vertex}$), 554 ($4Ag_{edge}$).

DOSY D = 4.71 × 10^{-10} m^2s^{-1}, log(D) = -9.33, r = 13.9 Å.

LR-ESI-MS [POS] $[Ag_{12}Cl_4L_6](NTf_2)_8$ *m/z* 2991.0 $[M\text{-}2NTf_2]^{2+}$, 1900.7 $[M\text{-}3NTf_2]^{3+}$, 1355.1 $[M\text{-}4NTf_2]^{4+}$. Fragmentation into $[Ag_6Cl_2L_3](NTf_2)_4$ contributes to the peaks at *m/z* 2991.0 $[M\text{-}NTf_2]^{1+}$, and 1355.1 $[M\text{-}2NTf_2]^{2+}$.

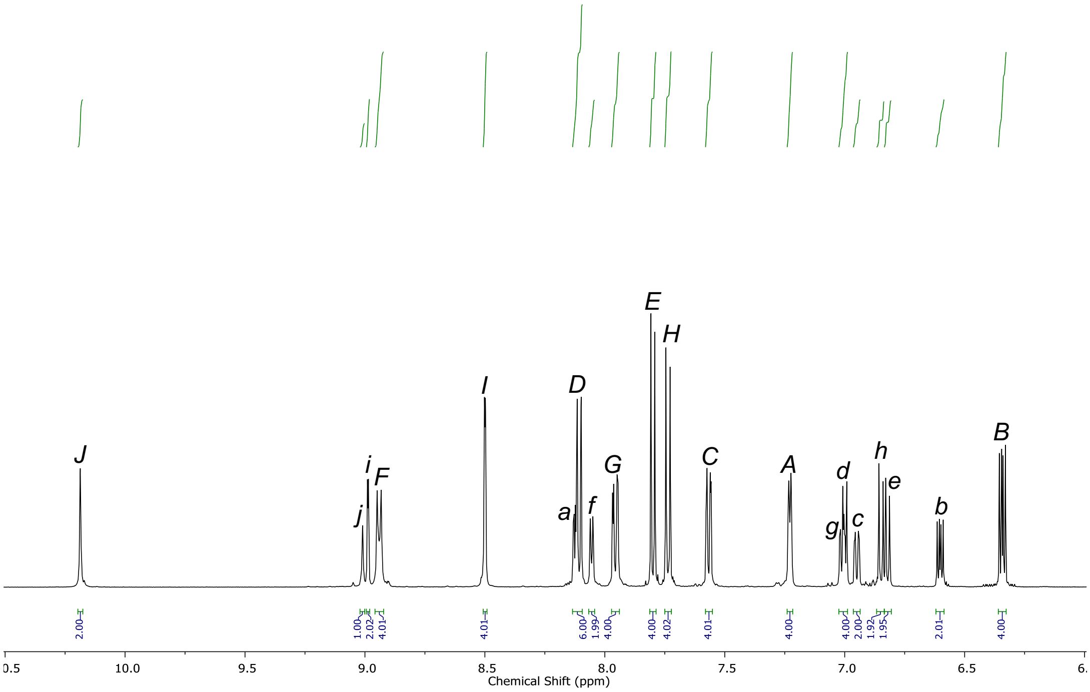

Figure S25. ^{1}H NMR of **1-Cl** (CD_3CN, 500 MHz, 298 K).

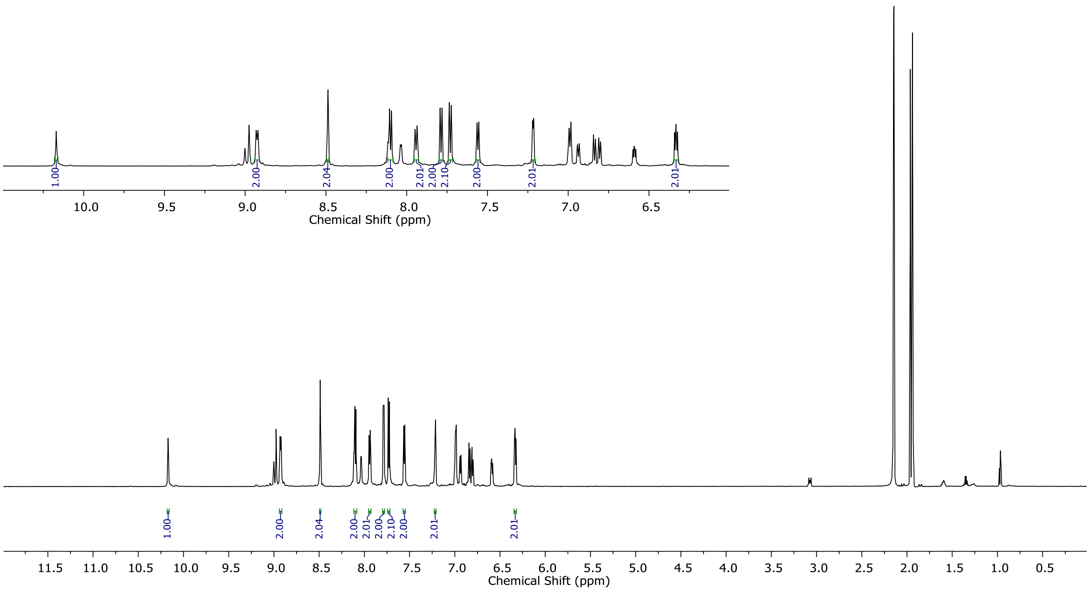

Figure S26. ^{1}H NMR showing the outer arm of **1-Cl** (CD_3CN, 500 MHz, 298 K).

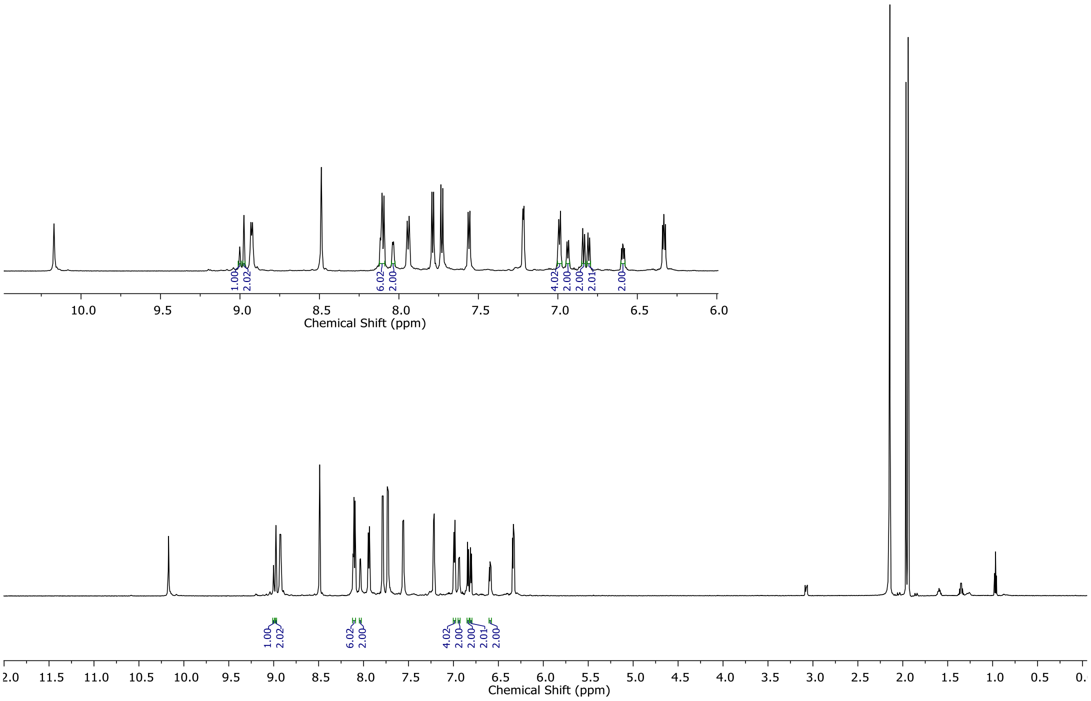

Figure S27. ^{1}H NMR showing the inner arm of **1-Cl** (CD_3CN, 500 MHz, 298 K).

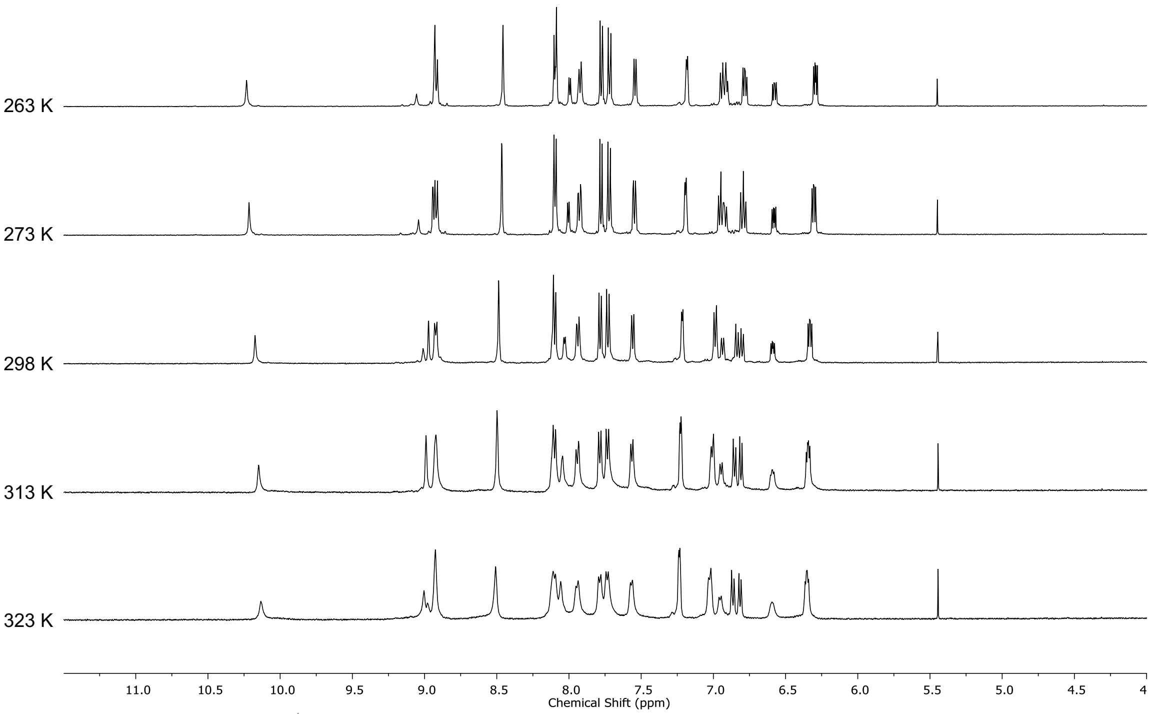

Figure S28. Variable temperature ^{1}H NMR of **1-Cl** (CD_3CN, 500 MHz).

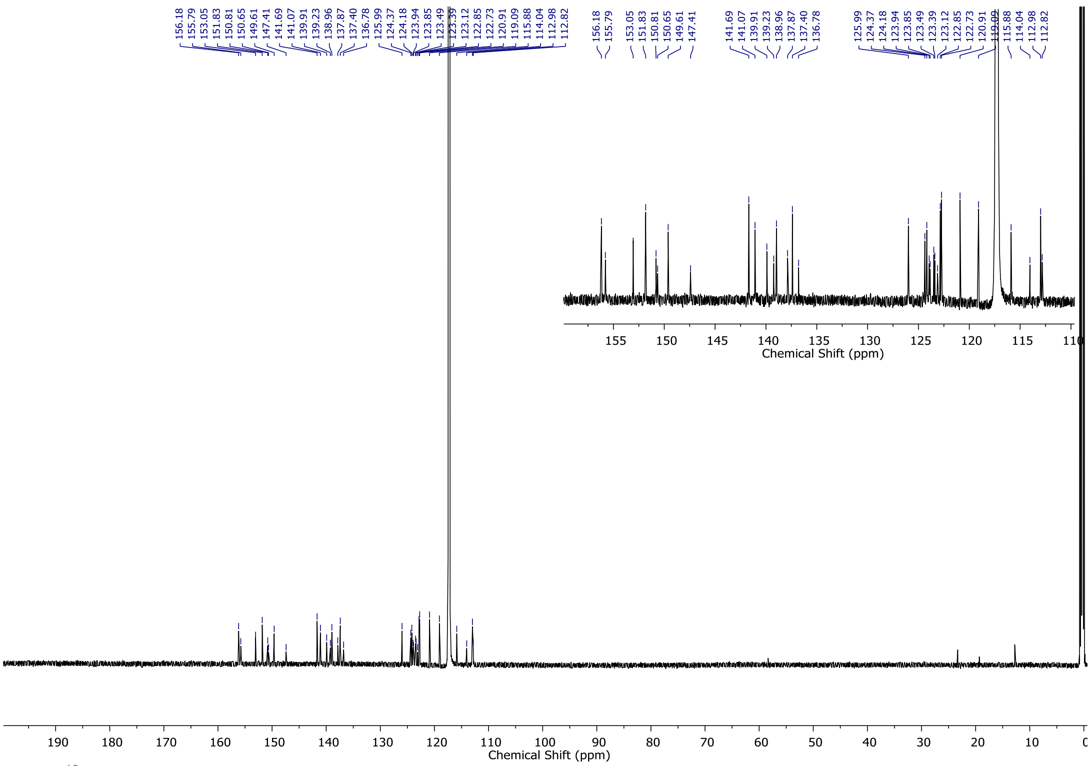

Figure S29. ^{13}C NMR of **1-Cl** (CD_3CN, 176 MHz, 298 K).

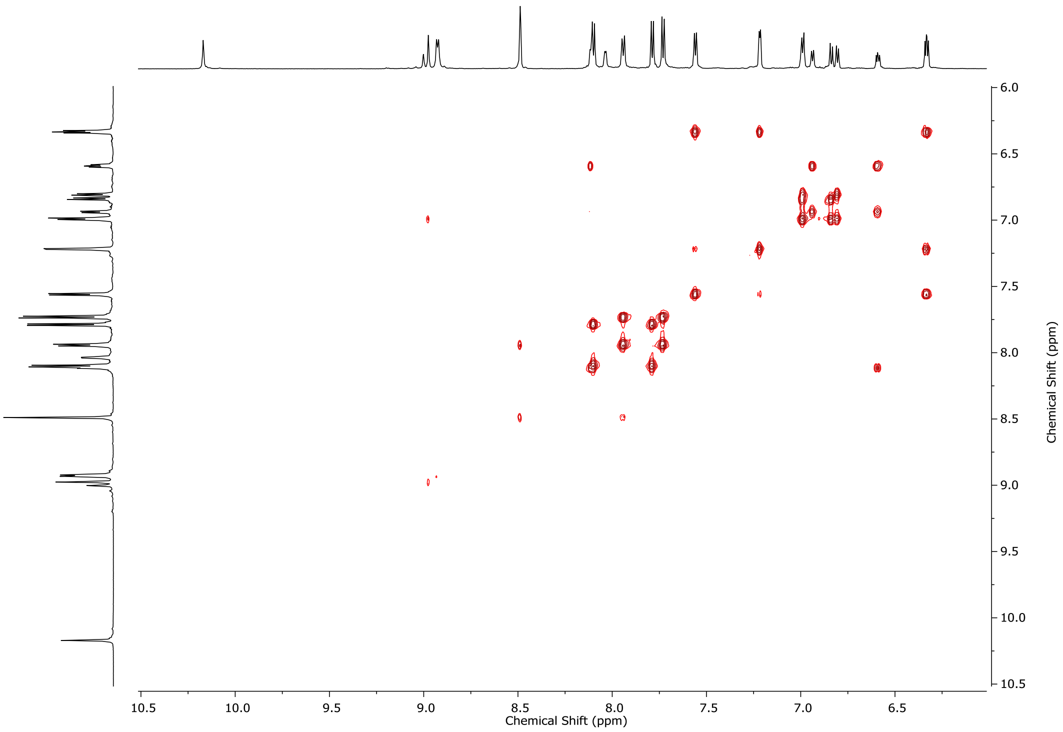

Figure S30. ^{1}H-^{1}H COSY NMR of **1-Cl** (CD_3CN, 298 K).

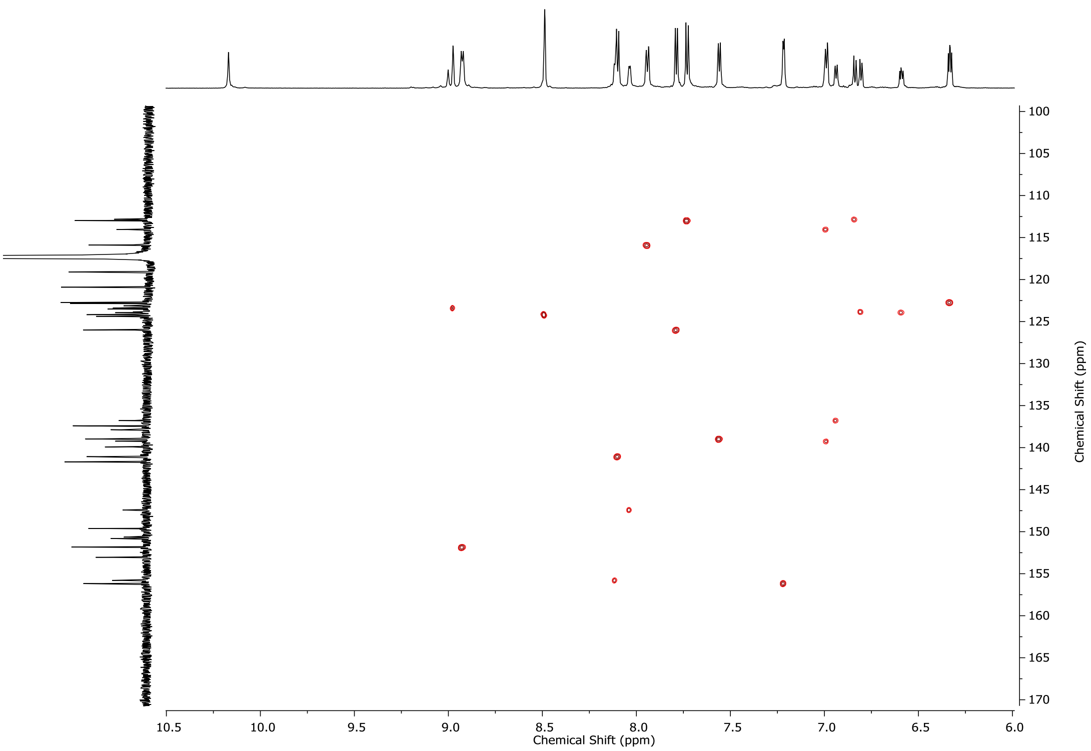

Figure S31. ^{1}H-^{13}C HSQC NMR of **1-Cl** (CD_3CN, 298 K).

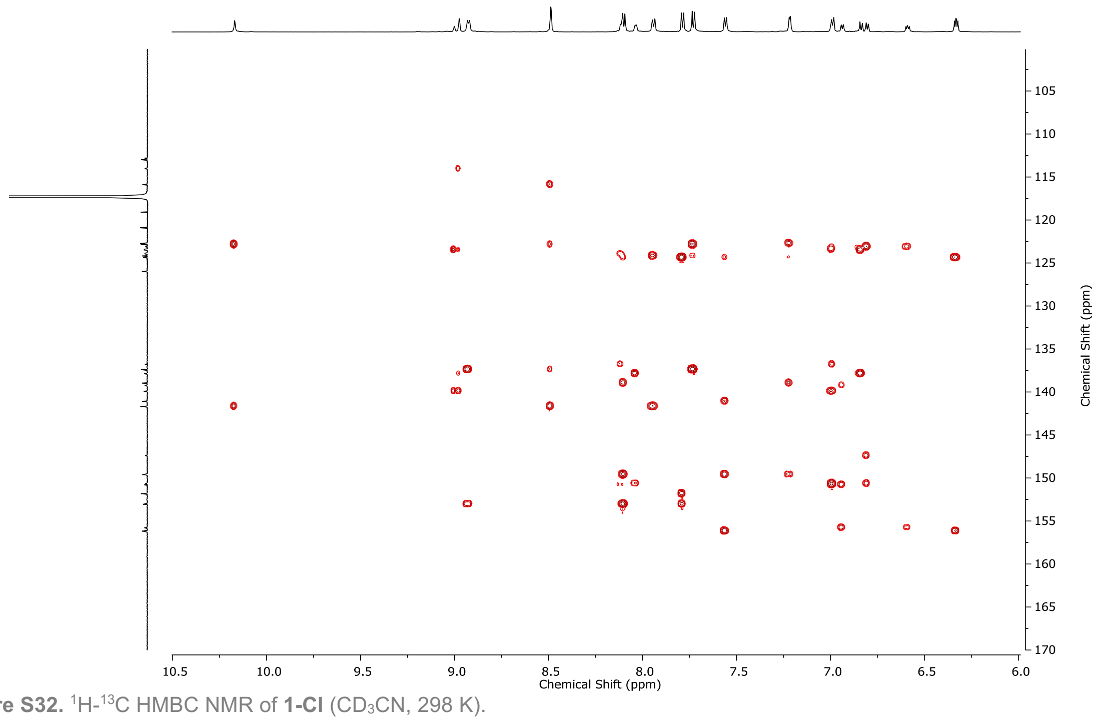

Figure S32. ^{1}H-^{13}C HMBC NMR of **1-Cl** (CD_3CN, 298 K).

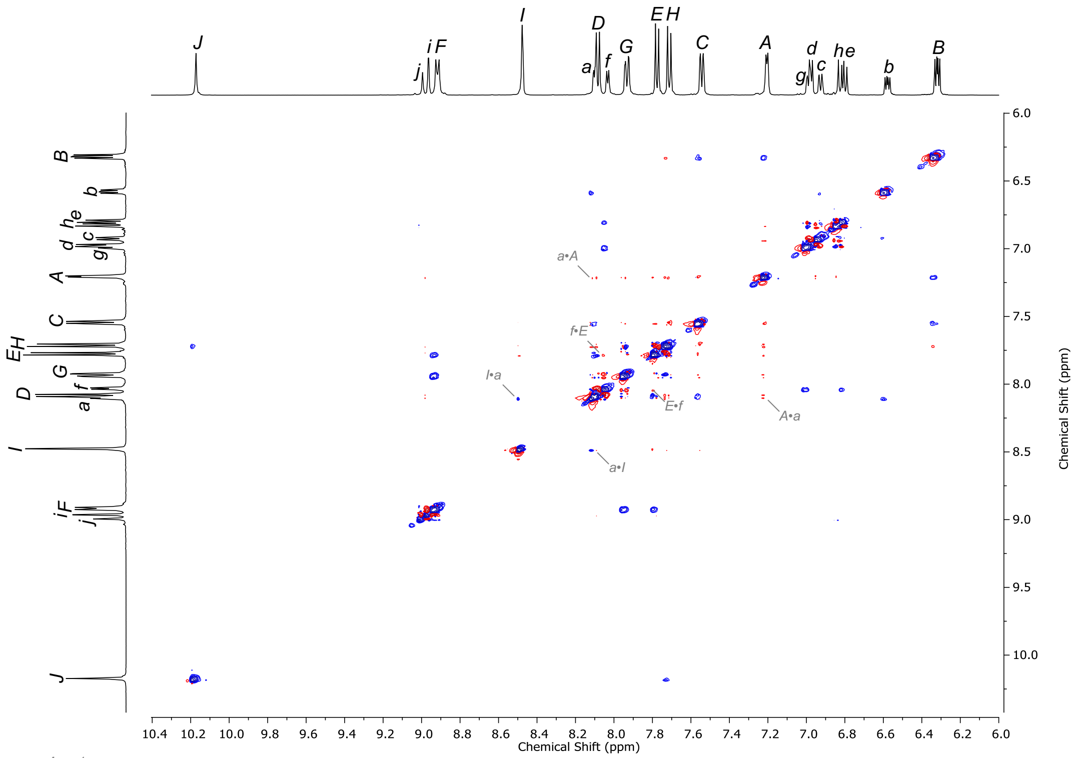

Figure S33. ^{1}H-^{1}H NOESY NMR of **1-Cl** with cross-peaks between inner and outer arms annotated (CD_3CN, 298 K).

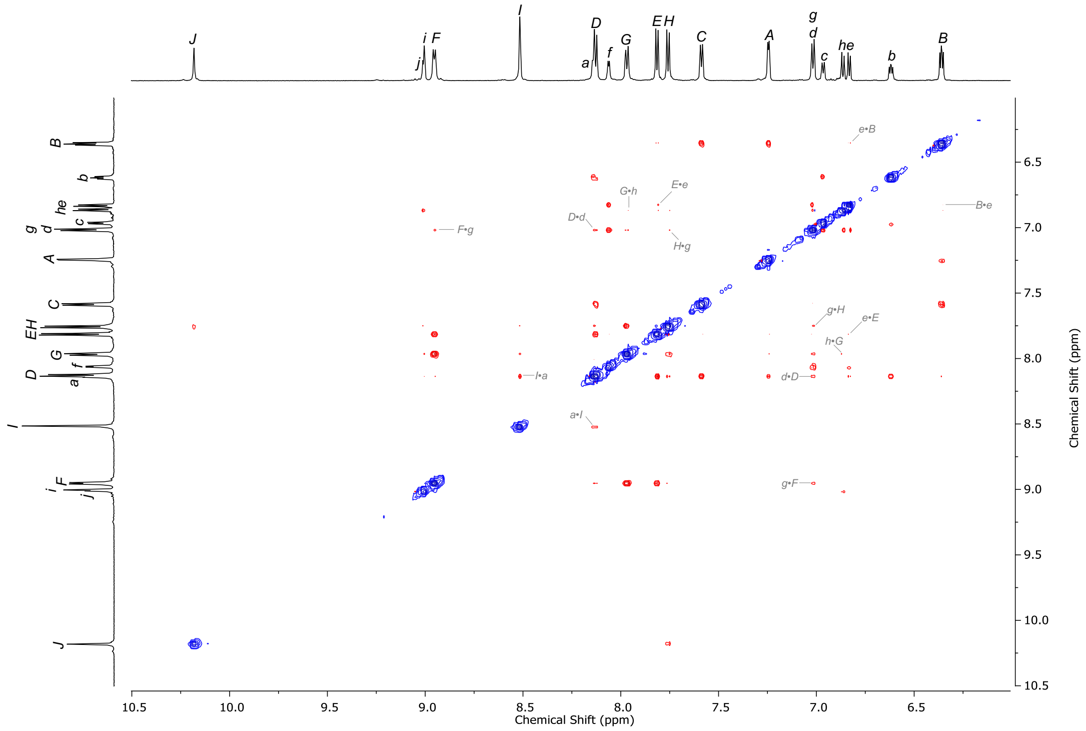

Figure S34. ^{1}H-^{1}H ROESY NMR of **1-Cl** with cross-peaks between inner and outer arms annotated (CD_3CN, 298 K).

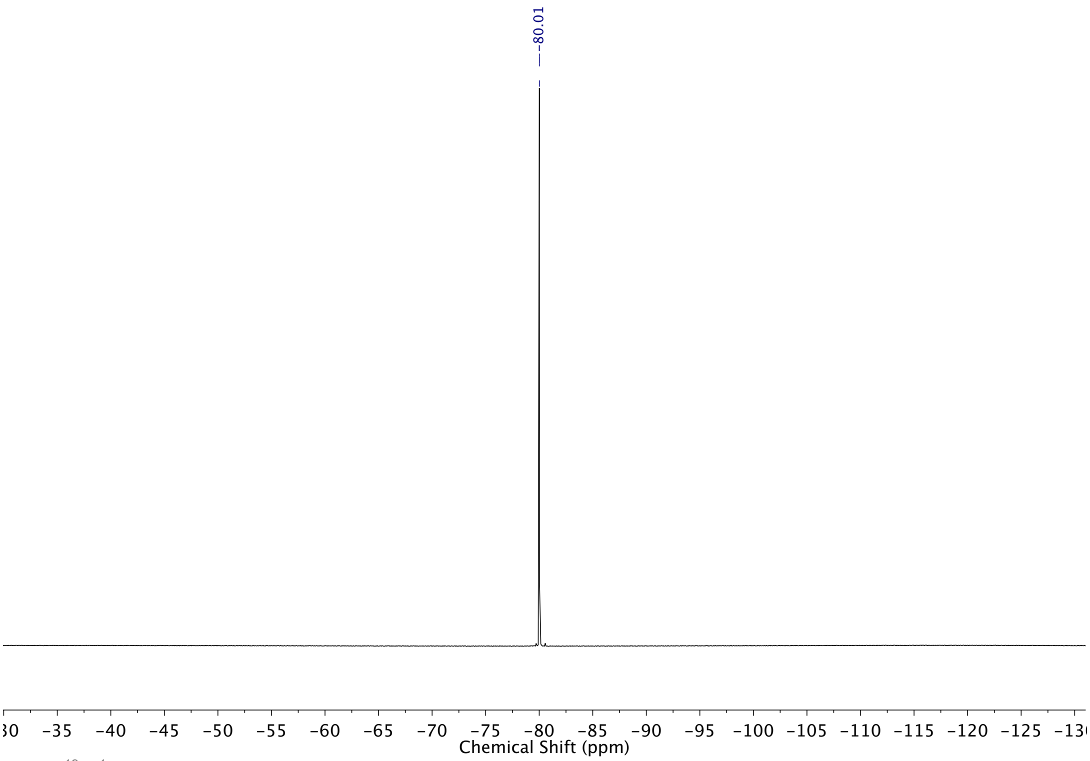

Figure S35. $^{19}F\{^1H\}$ NMR of **1-Cl** (CD_3CN, 376 MHz, 298 K).

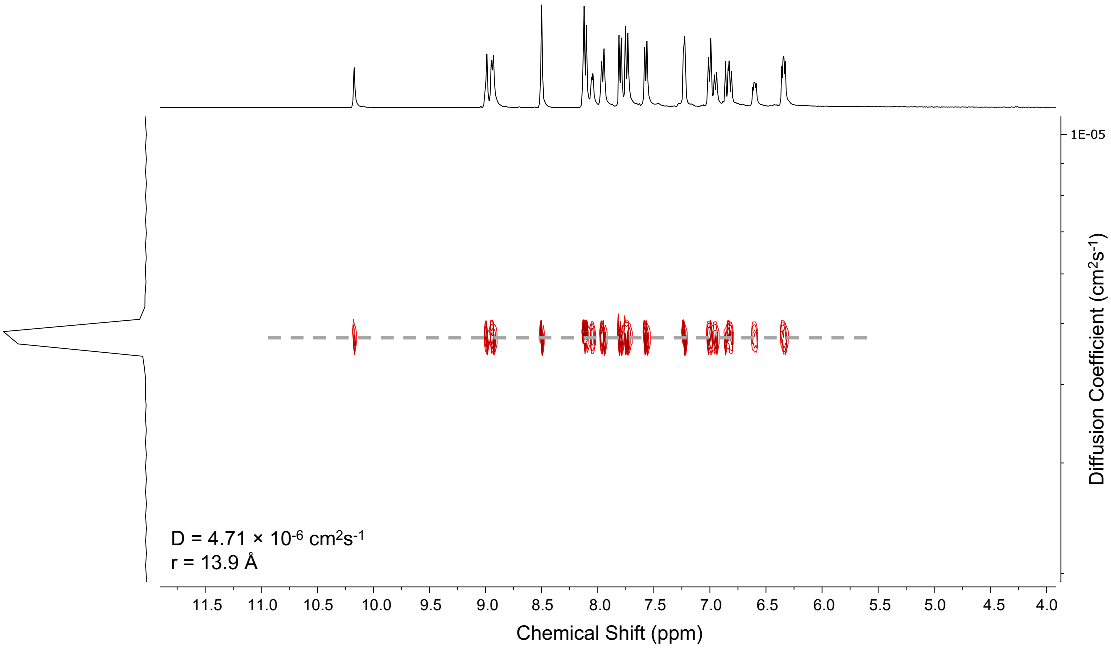

Figure S36. ^1H DOSY NMR of **1-Cl** (CD_3CN, 400 MHz, 298 K).

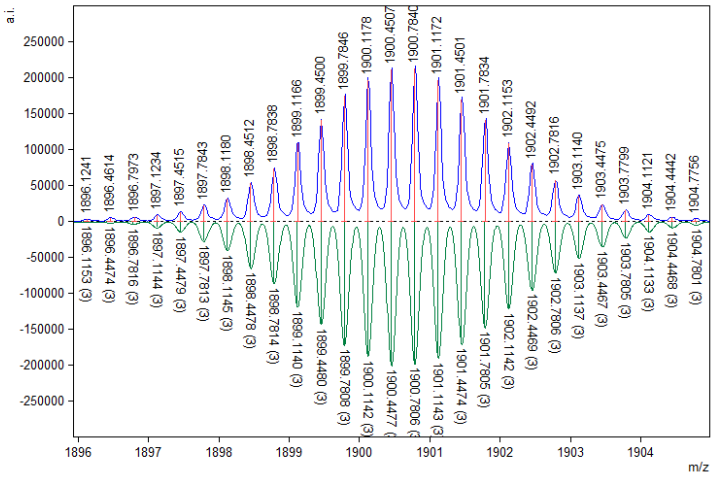

Figure S37. HR-ESI-MS [POS] **1-Cl** $[Ag_{12}Cl_4L_6](NTf_2)_8$ $[M-3NTf_2]^{3+}$ ($\delta_{expt-calc}$ 1.79 ppm).

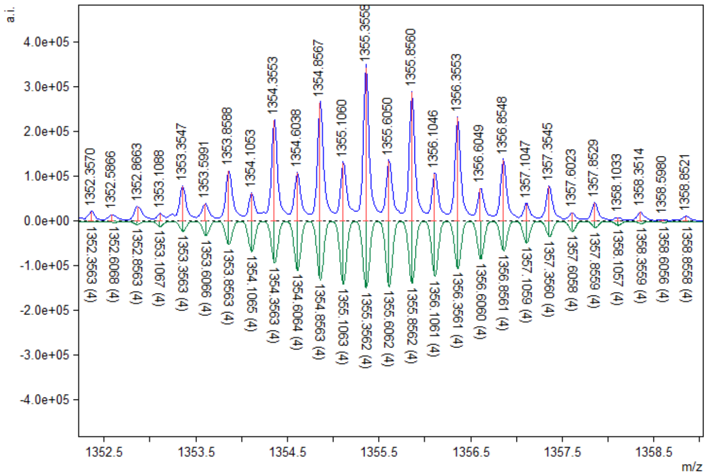

Figure S38. HR-ESI-MS [POS] **1-Cl** $[Ag_{12}Cl_4L_6](NTf_2)_8$ $[M-4NTf_2]^{4+}$ ($\delta_{expt-calc}$ 1.77 ppm), overlapping with $[Ag_6Cl_2L_3](NTf_2)_4$ $[M-2NTf_2]^{2+}$ fragment.

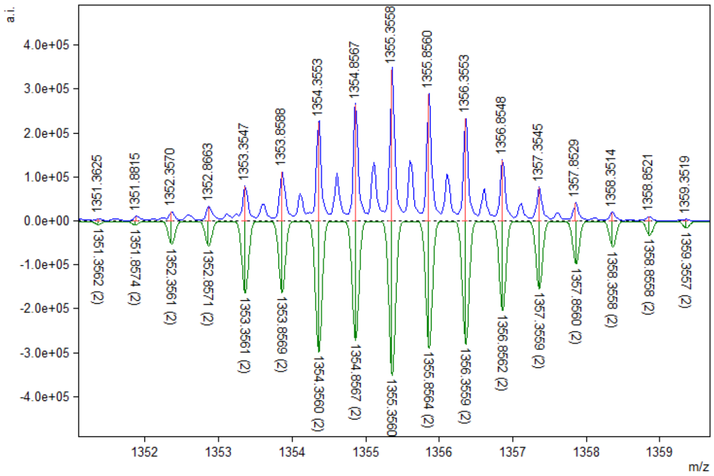

Figure S39. HR-ESI-MS [POS] **1-Cl** $[Ag_6Cl_2L_3](NTf_2)_4$ $[M-2NTf_2]^{2+}$ ($\delta_{expt-calc}$ 0.15 ppm).

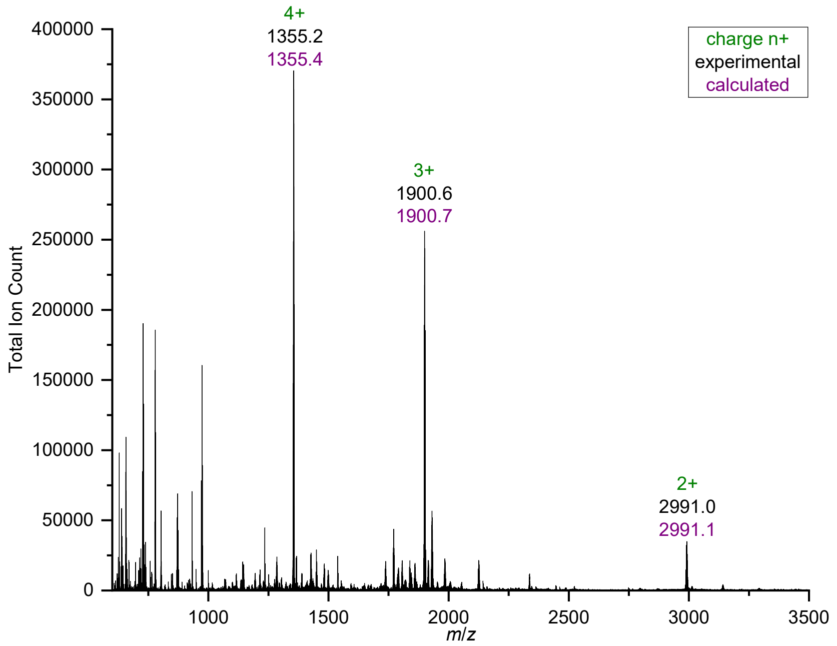

Figure S40. LR-ESI-MS [POS] **1-Cl** $[Ag_{12}Cl_4L_6](NTf_2)_8$ *m/z* 2991.0 $[M-2NTf_2]^{2+}$, 1900.7 $[M-3NTf_2]^{3+}$, 1355.1 $[M-2NTf_2]^{2+}$. Signals at 2991.1 and 1355.1 have contributions from the $[Ag_6Cl_2L_3](NTf_2)_4$ fragment.

3.4 ^{109}Ag NMR characterization of **1-Cl**

At 298 K, 1D ^{109}Ag NMR spectroscopy showed two Ag environments (Figure S41) corresponding to the 8 Ag atoms at the vertex environment (632 ppm) and the 4 Ag atoms in the environment along the edge (554 ppm). A ^{109}Ag INEPT spectrum (Figure S42) showed these same environments with improved signal-to-noise ratio. At 298 K, the ^{1}H-^{109}Ag HMBC spectrum (Figure S43) confirmed the assignment through correlations to key ^{1}H environments. Selective ^{109}Ag decoupling of the ^{1}H NMR spectra (Figure S44- Figure S47) further confirm the ^{109}Ag assignment. Cooling the sample to 232 K (Figure S48) and 272 K (Figure S49) showed the same behavior.

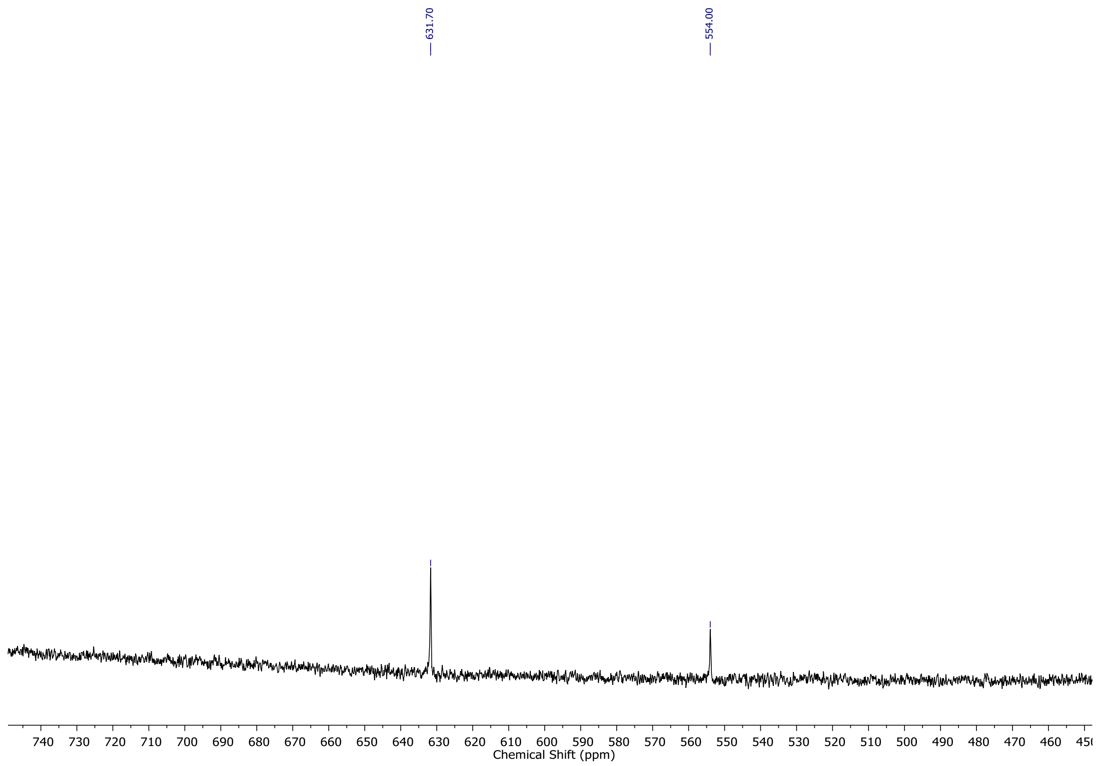

Figure S41. Baseline corrected ^{109}Ag NMR of **1-Cl** (CD_3CN, 23 MHz, 298 K).

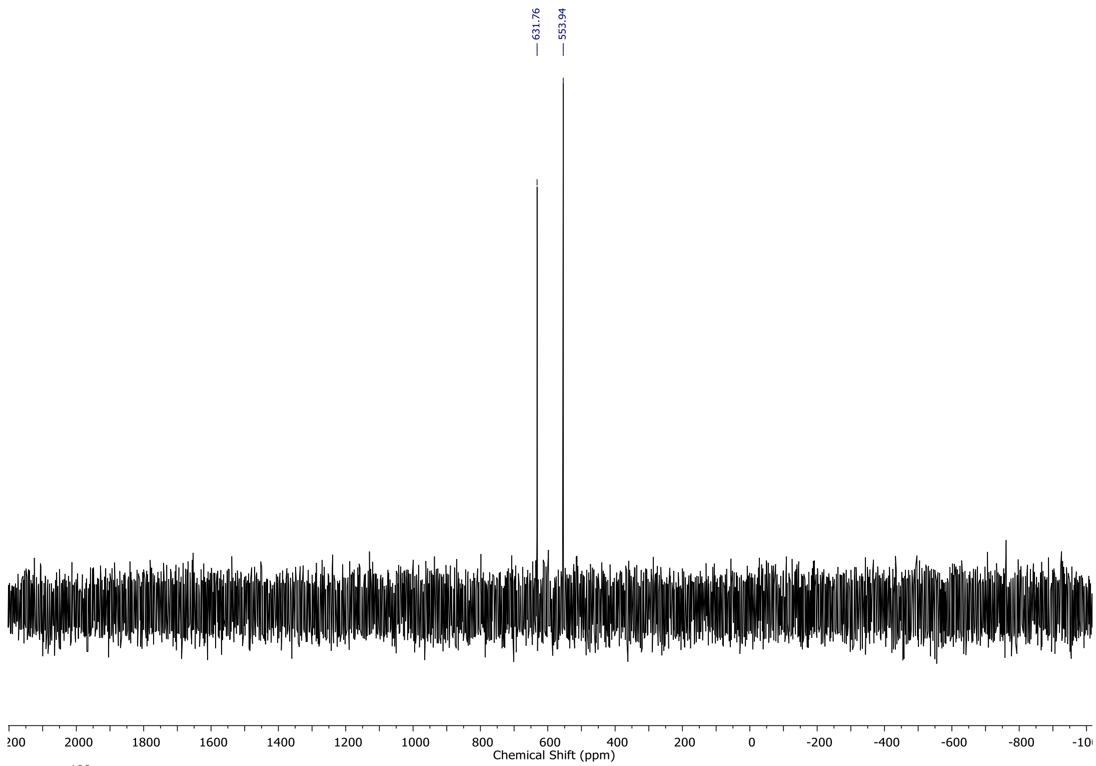

Figure S42. ^{109}Ag INEPT NMR of **1-Cl** (CD_3CN, 23 MHz, 298 K).

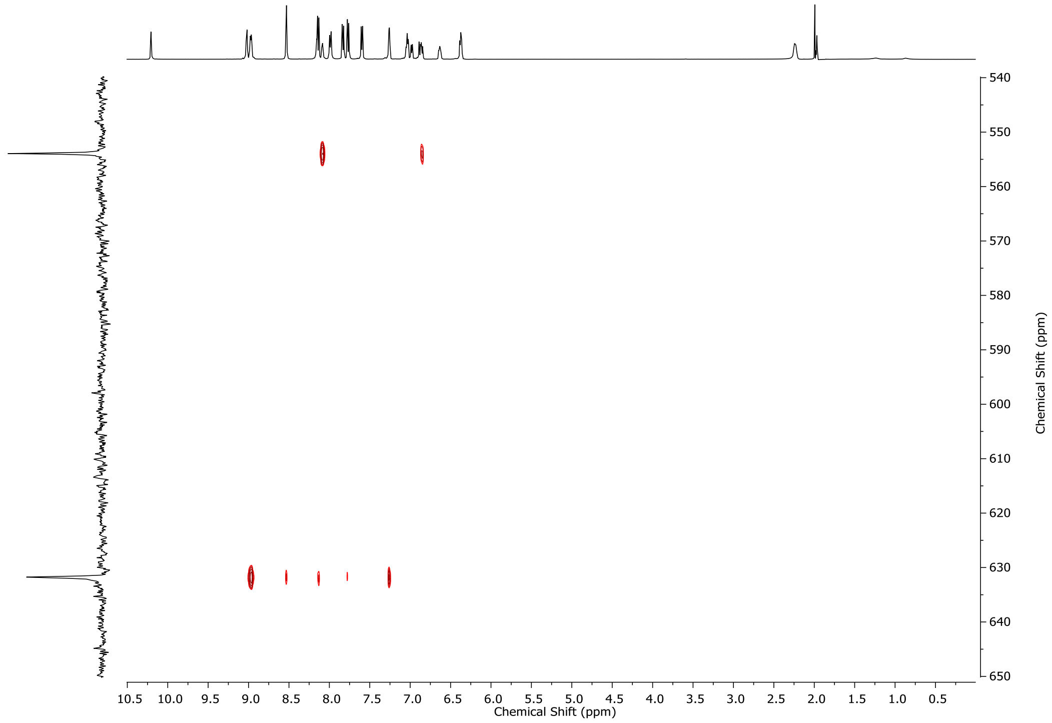

Figure S43. ^{1}H-^{109}Ag HMBC NMR of **1-Cl** (CD_3CN, 298 K).

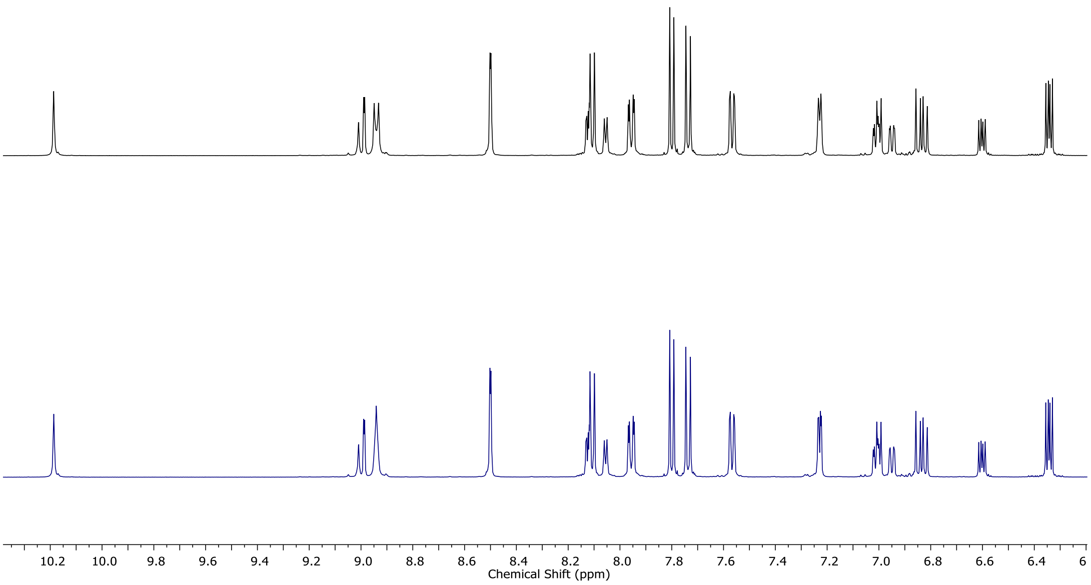

Figure S44. Stackplot of standard ^{1}H NMR (top, black) and ^{1}H NMR with ^{109}Ag decoupling at 632 ppm (bottom, blue) of **1-Cl** (CD_3CN, 500 MHz, 298 K).

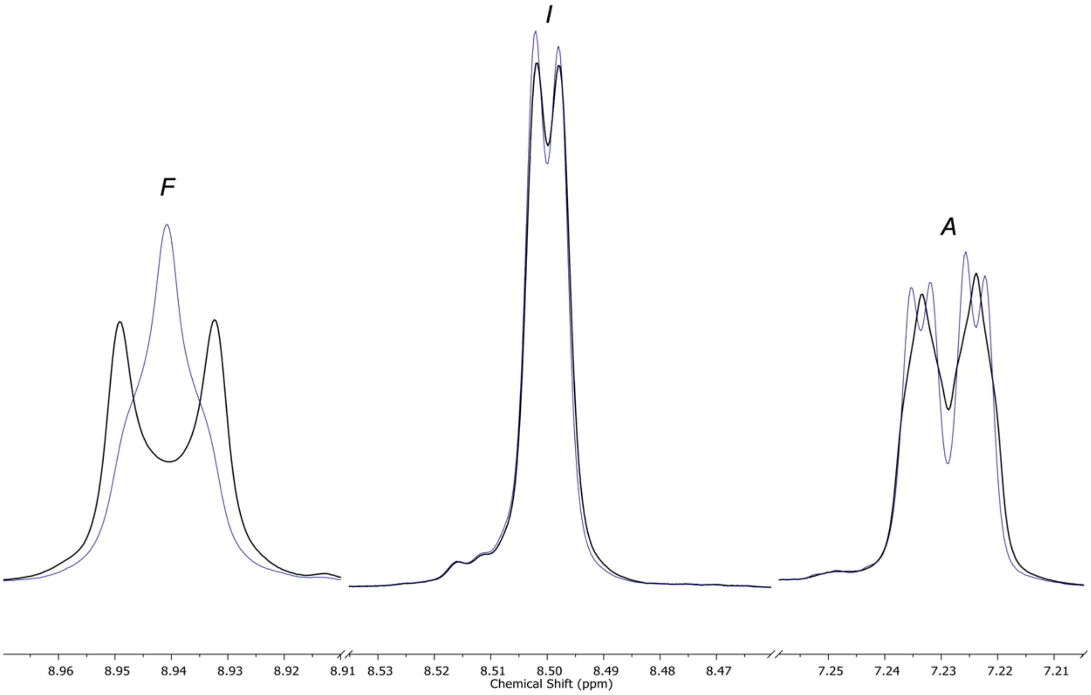

Figure S45. Superposition of standard ^{1}H NMR (black) and ^{1}H NMR with ^{109}Ag decoupling at 632 ppm (blue) of **1-Cl** (CD_3CN, 500 MHz, 298 K).

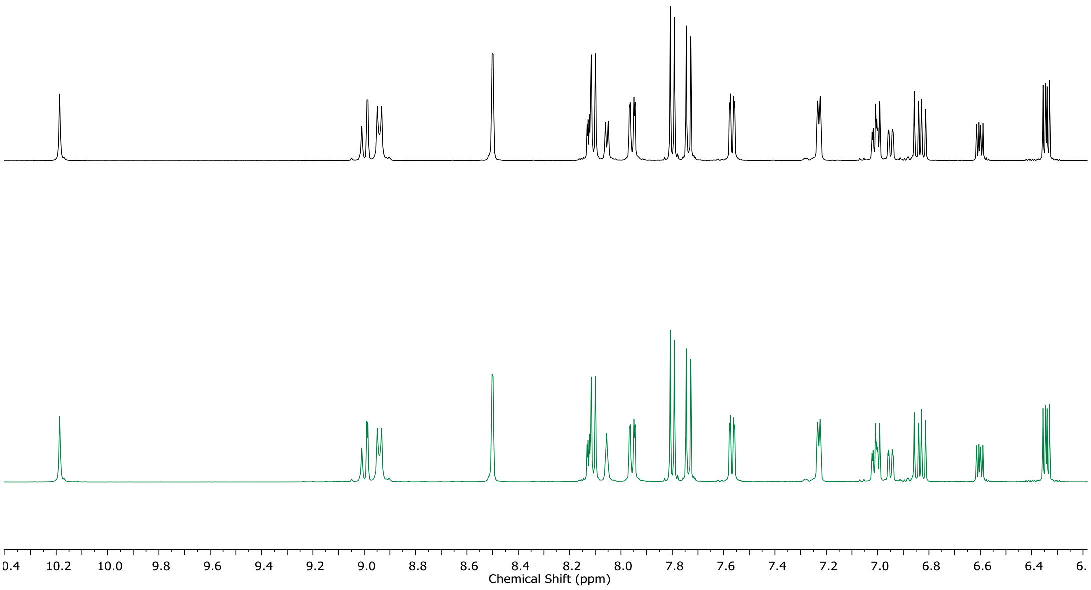

Figure S46. Stackplot of standard ^{1}H NMR (top, black) and ^{1}H NMR with ^{109}Ag decoupling at 554 ppm (bottom, green) of **1-Cl** (CD_3CN, 500 MHz, 298 K).

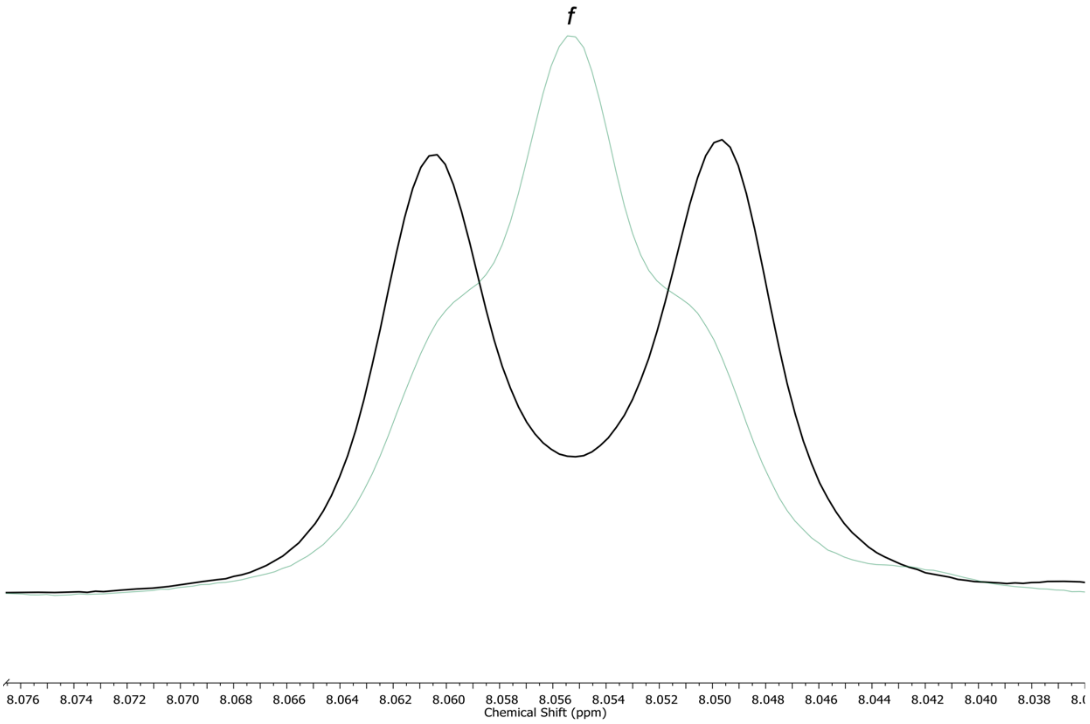

Figure S47. Superposition of standard ^{1}H NMR (black) and ^{1}H NMR with ^{109}Ag decoupling at 554 ppm (green) of **1-Cl** (CD_3CN, 500 MHz, 298 K).

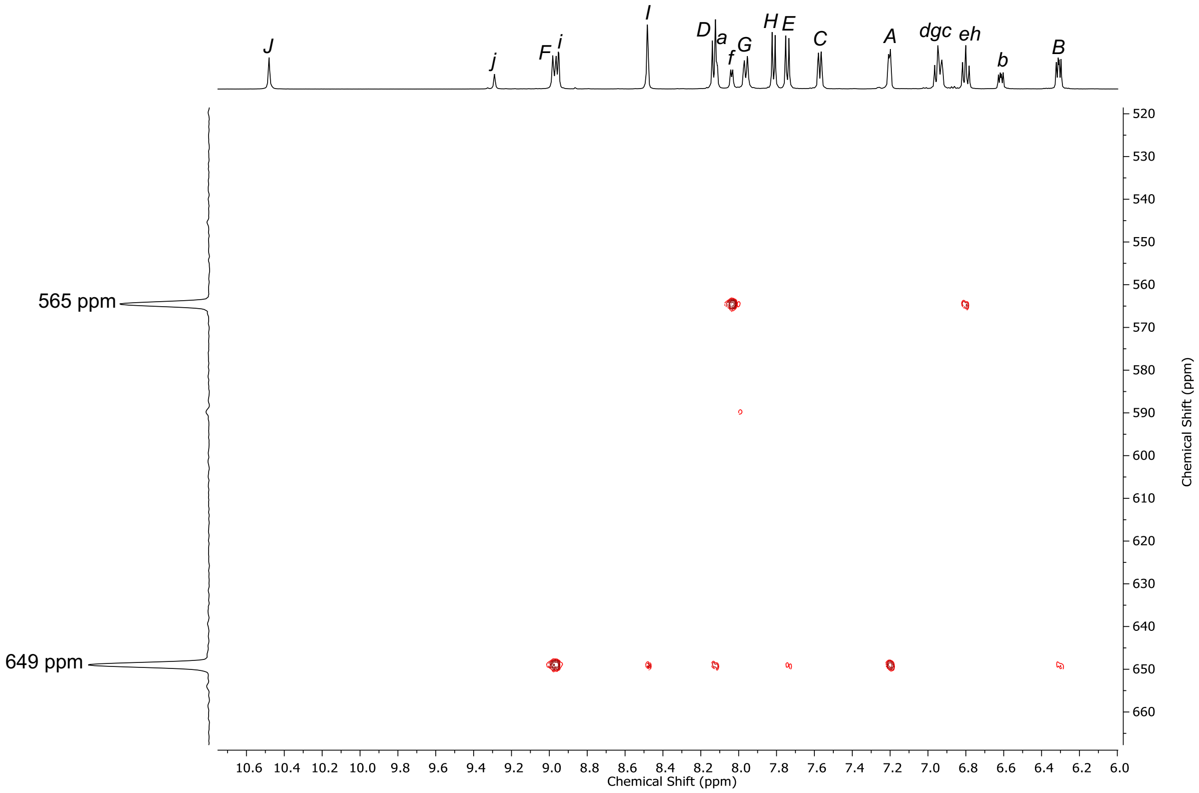

Figure S48. Enlarged ^{1}H-^{109}Ag HMBC NMR of **1-Cl** (CD_3CN, 232 K).

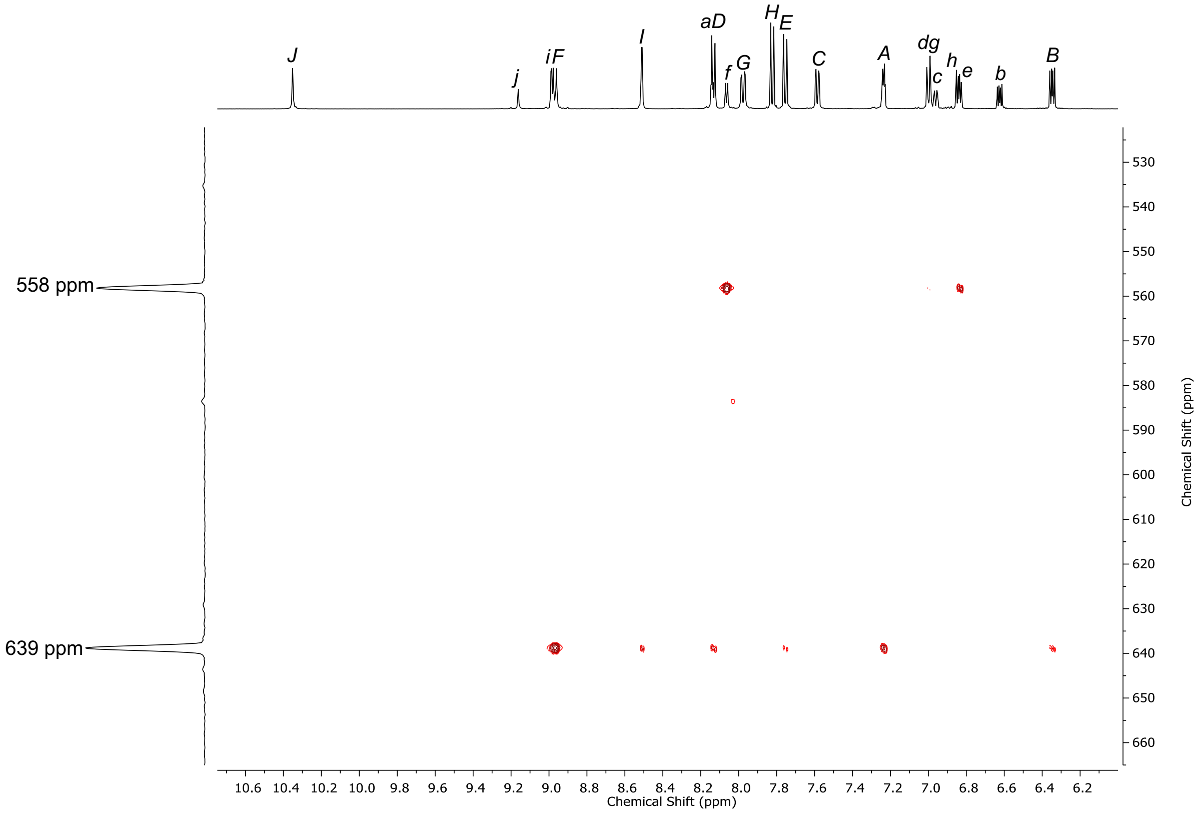

Figure S49. Enlarged ^{1}H-^{109}Ag HMBC NMR of **1-Cl** (CD_3CN, 272 K).

3.5 Synthesis and characterization of **1-Br**

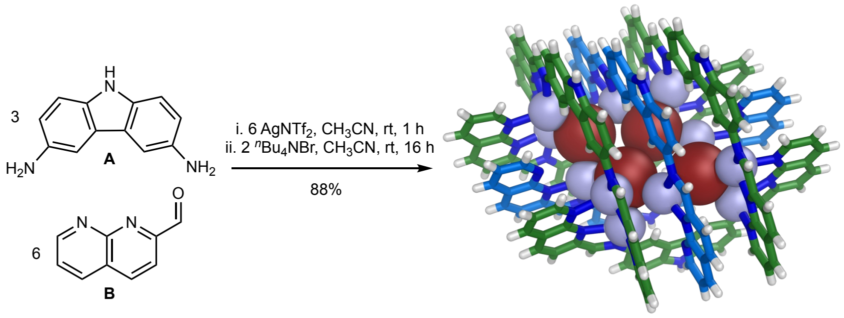

$[Ag^I_{12}Br_4L_6](NTf_2)_8$

3,6-diamino-9*H*-carbazole **A** (3.94 mg, 20.0 µmol, 3.0 eq.), 2-formyl-1,8-naphthyridine **B** (6.32 mg, 40.0 µmol, 6.0 eq.) and $AgNTf_2$ (15.52 mg, 40.0 µmol, 6.0 eq.) were stirred in CH_3CN (1.5 mL) at rt for 1 h. $^nBu_4NBr$ (4.30 mg, 13.3 µmol, 2.0 eq.) in CH_3CN (0.5 mL) was added and the reaction stirred at rt for 16 h. The reaction was concentrated under N_2, washed with Et_2O (3 × 10 mL), dissolved in CH_3CN, filtered and concentrated *in vacuo* yielding a dark red solid **1-Br** (19.7 mg, 2.93 µmol, 88%, 6720.3 $gmol^{-1}$).

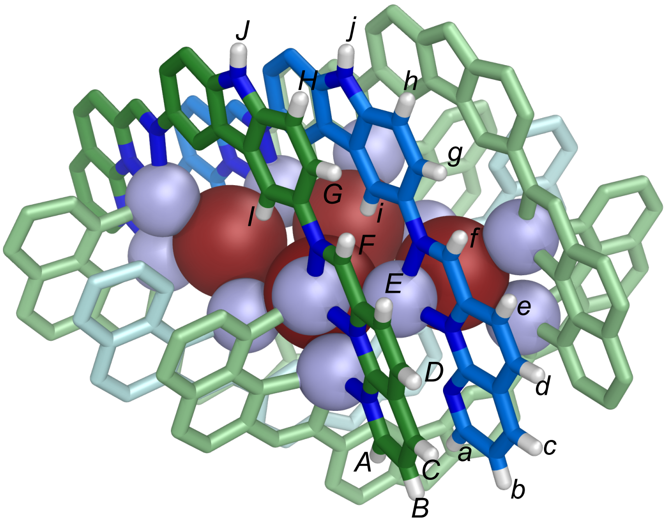

δ_H (CD_3CN, 700 MHz) 10.14 (2H, s, H_J), 9.16 (1H, s, H_j), 8.88 (4H, s, H_F), 8.83 (2H, s, H_i), 8.53 (4H, d, J = 1.5, H_I), 8.15 (2H, dd, J = 4.9, 1.5, H_a), 8.12 (4H, d, J = 8.0, H_D), 7.96 (2H, s, H_f), 7.90 (4H, d, J = 7.9, H_G), 7.79 (4H, d, J = 8.0, H_E), 7.70 (4H, d, J = 8.7, H_H), 7.58 (4H, d, J = 7.5, H_C), 7.35 (4H, d, J = 5.1, H_A), 7.13 (2H, d, J = 7.6, H_d), 7.02-6.97 (6H, m, H_e, H_g, H_h), 6.91 (2H, d, J = 7.4, H_c), 6.53 (2H, dd, J = 7.5, 4.9, H_b), 6.39 (4H, dd, J = 7.5, 5.1, H_B).

δ_C (CD_3CN, 176 MHz) 157.3 (C_A), 156.7 (C_a), 154.1, 153.0 (C_F), 151.6, 151.4, 150.8, 148.7 (C_f), 142.5, 142.0 (C_D), 141.0, 140.5 (C_d), 140.0 (C_C), 138.9, 138.6, 137.4 (C_c), 127.0 (C_E), 125.4, 124.9 (C_e), 124.8 (C_I), 124.7 (C_b), 124.3, 124.2, 124.1 (C_i), 123.9, 123.7 (C_B), 120.9 (q, $^1J_{CF}$ = 320.6), 116.7 (C_G), 115.3 (C_g), 114.1 (C_H), 113.7 (C_h).

δ_F (CD_3CN, 376 MHz) -80.1 ($N(SO_2CF_3)_2$).

δ_{Ag} (CD_3CN, 23 MHz) 683 ($8Ag_{vertex}$), 603 ($4Ag_{edge}$).

DOSY D = 4.85 × 10^{-10} m^2s^{-1}, log(D) = -9.31, r = 13.5 Å.

LR-ESI-MS [POS] $[Ag_{12}Br_4L_6](NTf_2)_8$ *m/z* 3080.0 $[M-2NTf_2]^{2+}$, 1960.0 $[M-3NTf_2]^{3+}$, 1400.0 $[M-4NTf_2]^{4+}$. Fragmentation into $[Ag_6Br_2L_3](NTf_2)_4$ contributes to the peaks at *m/z* 3080.0 $[M-NTf_2]^{1+}$, and 1400.0 $[M-2NTf_2]^{2+}$.

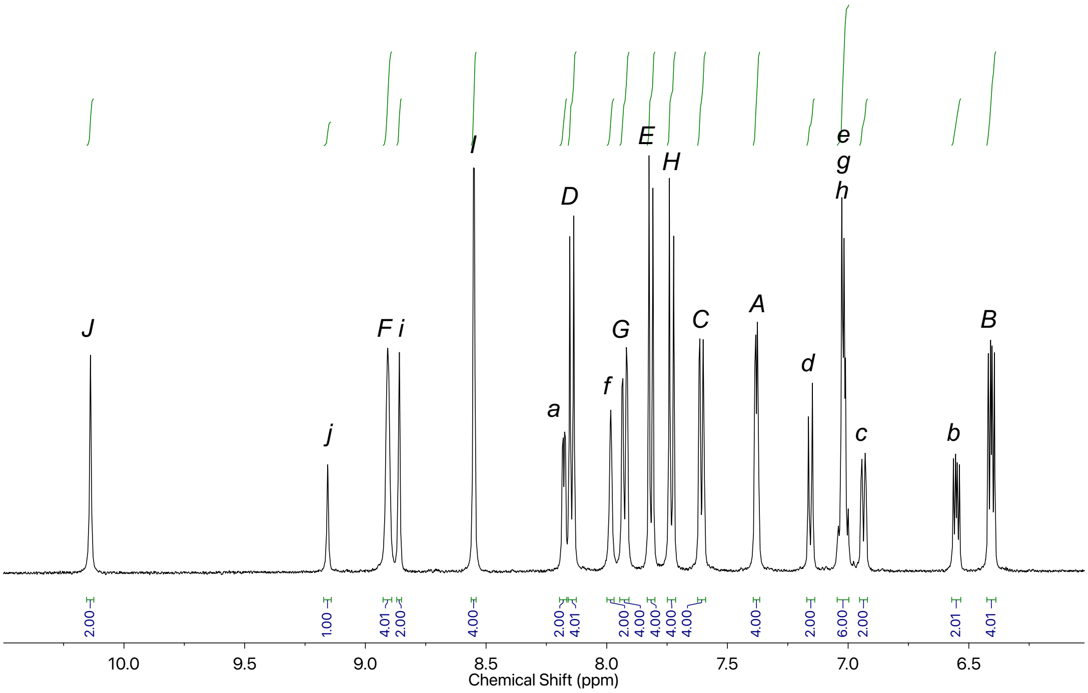

Figure S50. ^{1}H NMR of **1-Br** (CD_3CN, 700 MHz, 298 K).

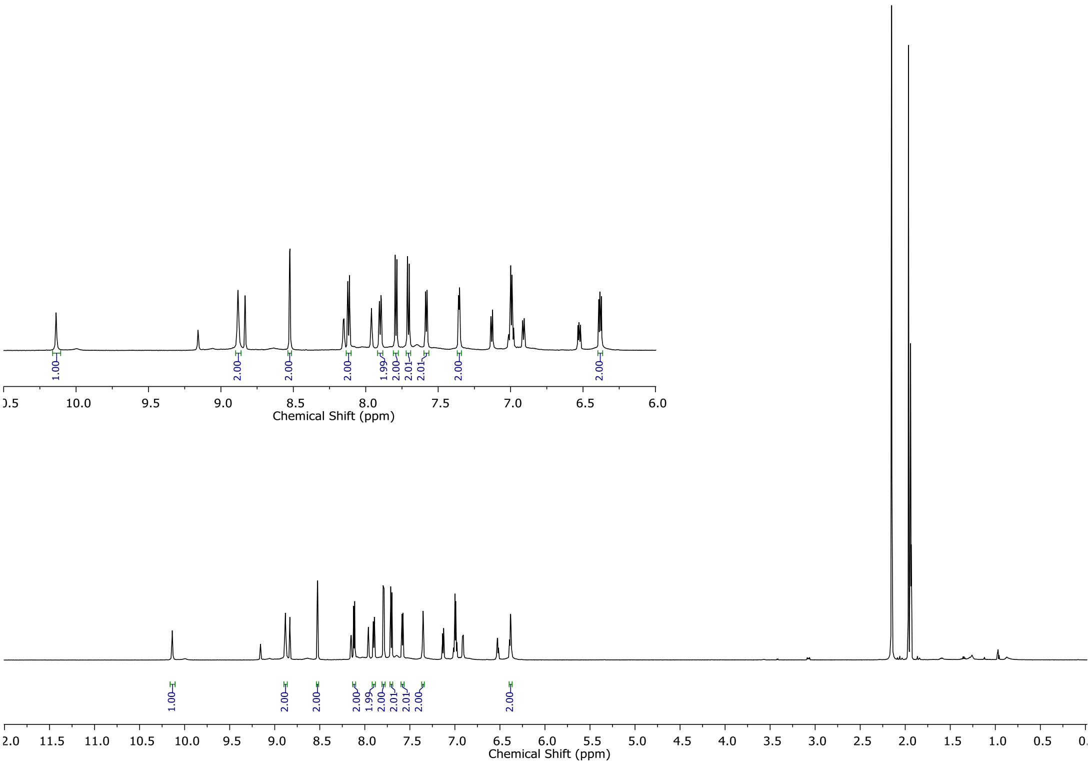

Figure S51. ^{1}H NMR showing the outer arms of **1-Br** (CD_3CN, 700 MHz, 298 K).

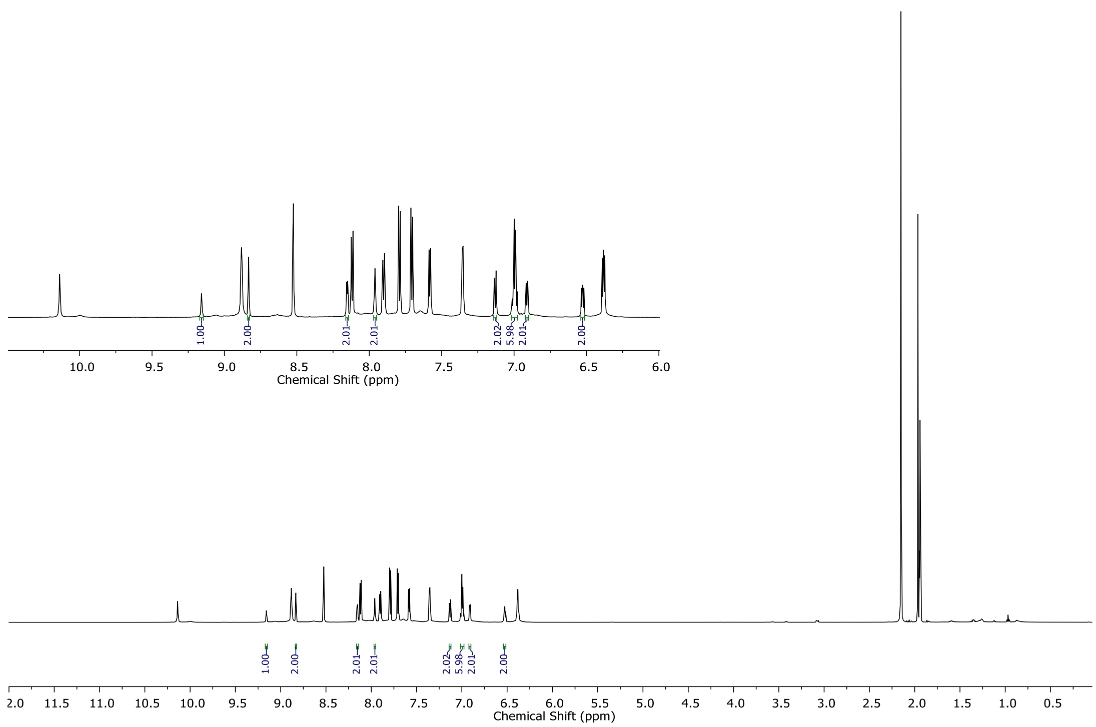

Figure S52. ^{1}H NMR showing the inner arms of **1-Br** (CD_3CN, 700 MHz, 298 K).

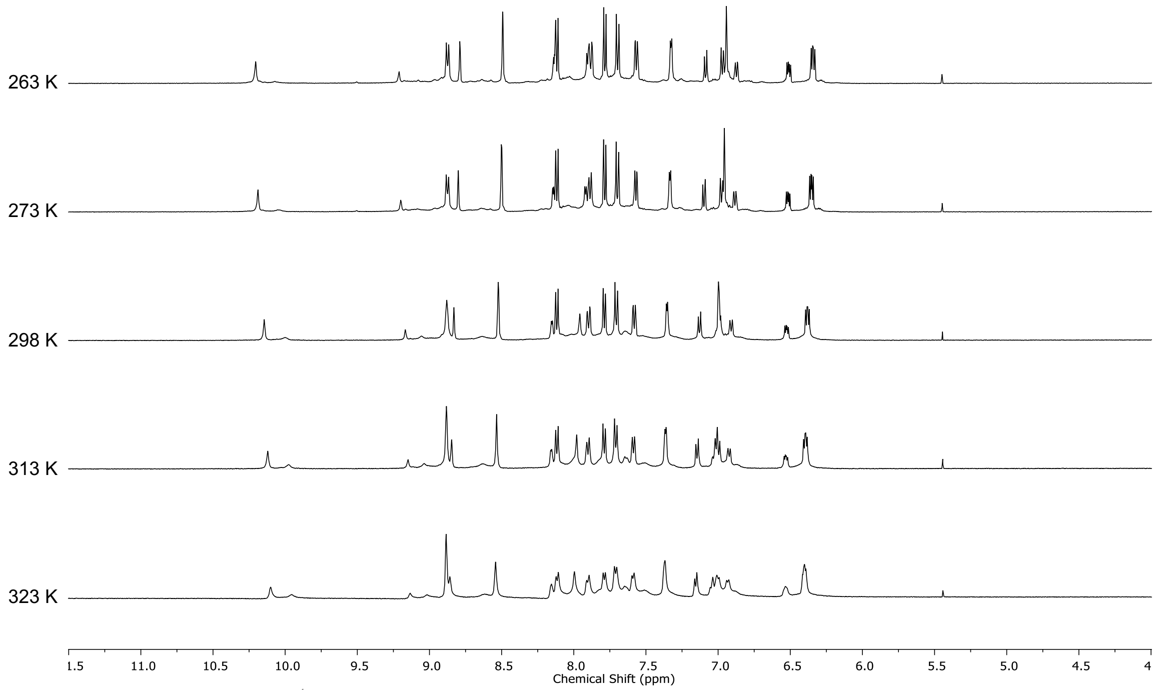

Figure S53. Variable temperature ^{1}H NMR of **1-Br** (CD_3CN, 500 MHz).

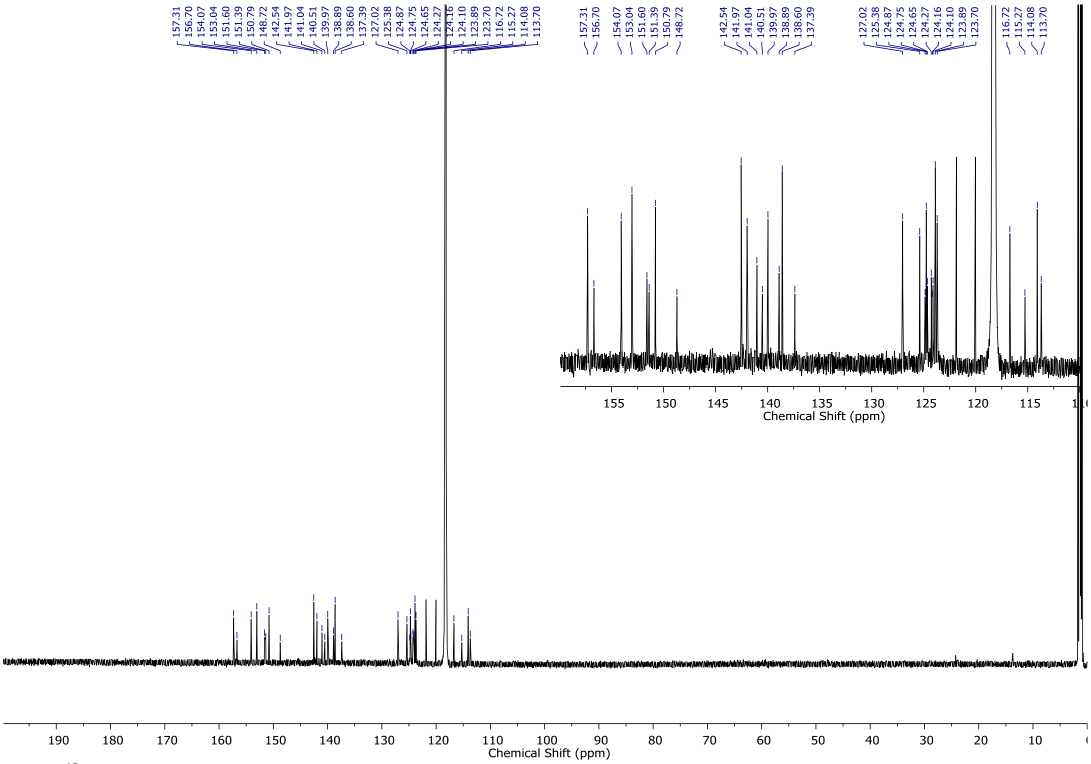

Figure S54. ^{13}C NMR of **1-Br** (CD_3CN, 176 MHz, 298 K).

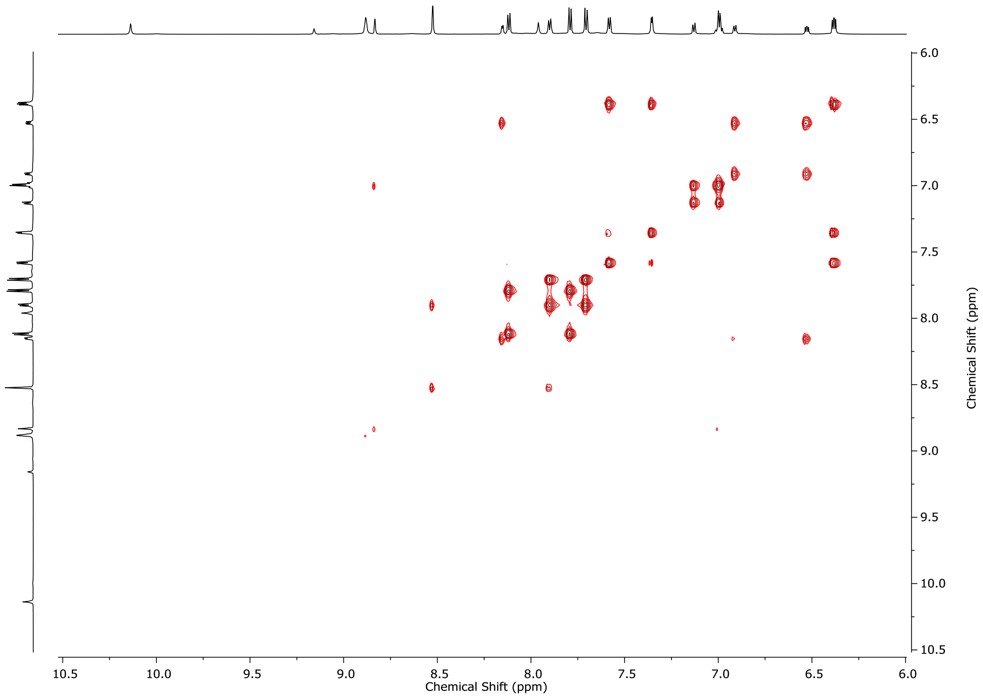

Figure S55. ^{1}H-^{1}H COSY NMR of **1-Br** (CD_3CN, 298 K).

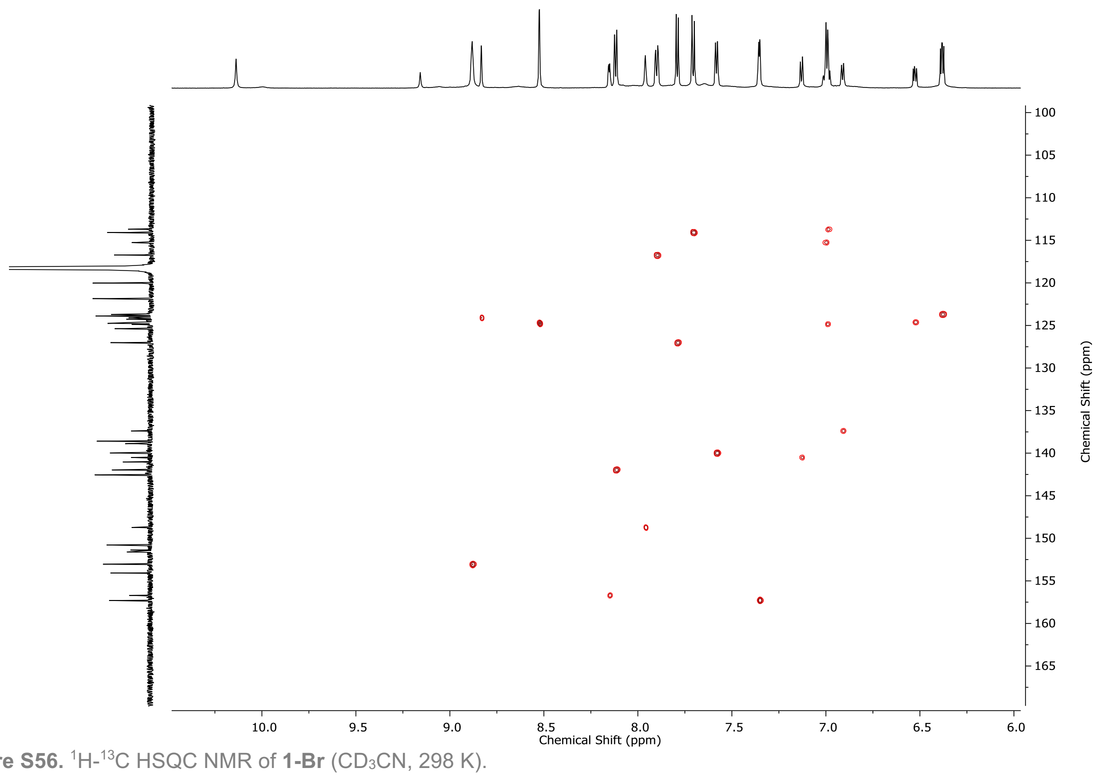

Figure S56. ^{1}H-^{13}C HSQC NMR of **1-Br** (CD_3CN, 298 K).

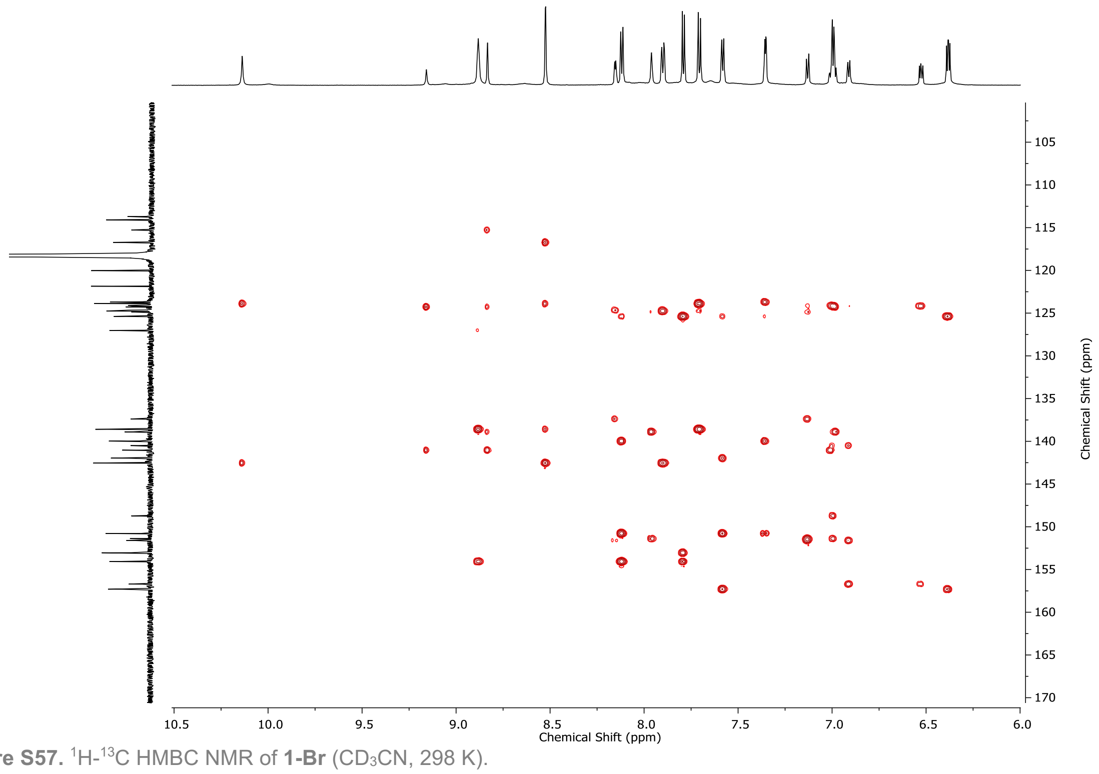

Figure S57. ^{1}H-^{13}C HMBC NMR of **1-Br** (CD_3CN, 298 K).

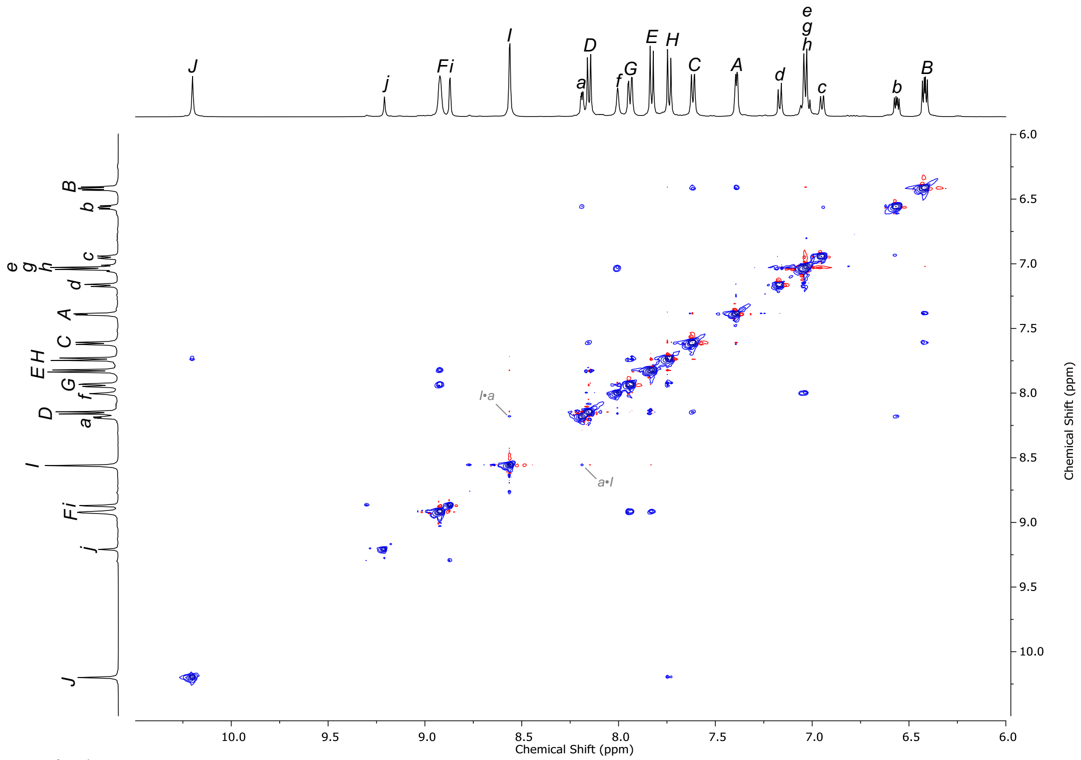

Figure S58. ^{1}H-^{1}H NOESY NMR of **1-Br** with cross-peaks between inner and outer arms annotated (CD_3CN, 298 K).

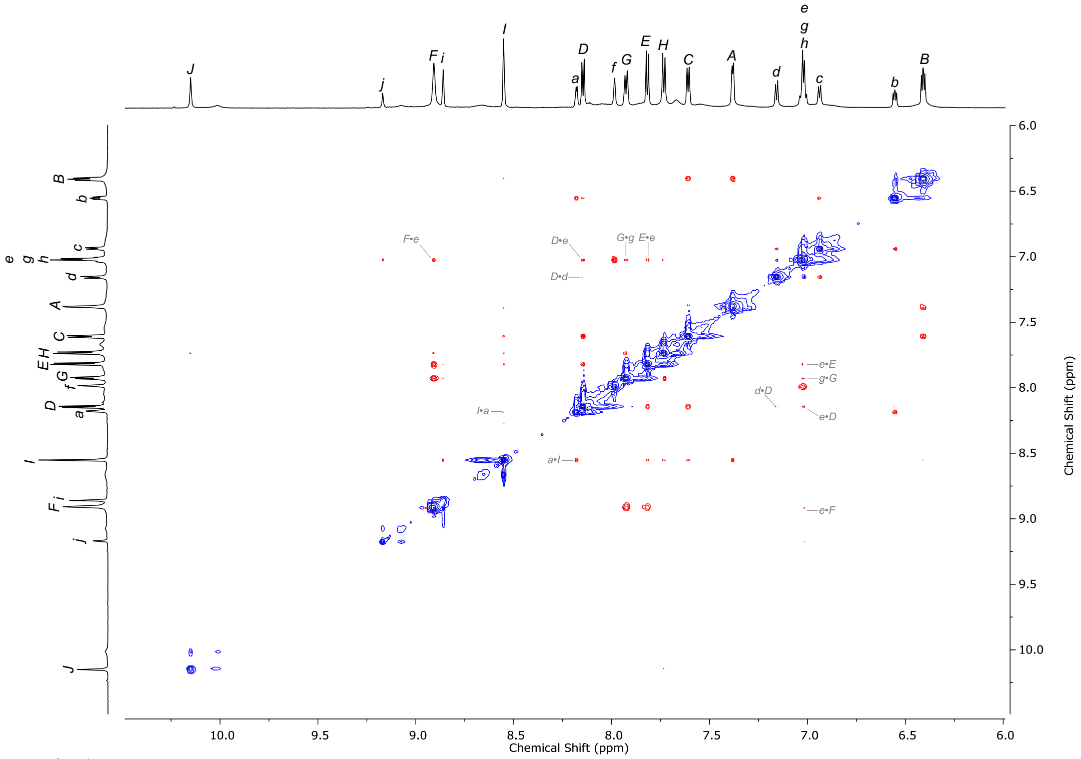

Figure S59. ^{1}H-^{1}H ROESY NMR of **1-Br** with cross-peaks between inner and outer arms annotated (CD_3CN, 298 K).

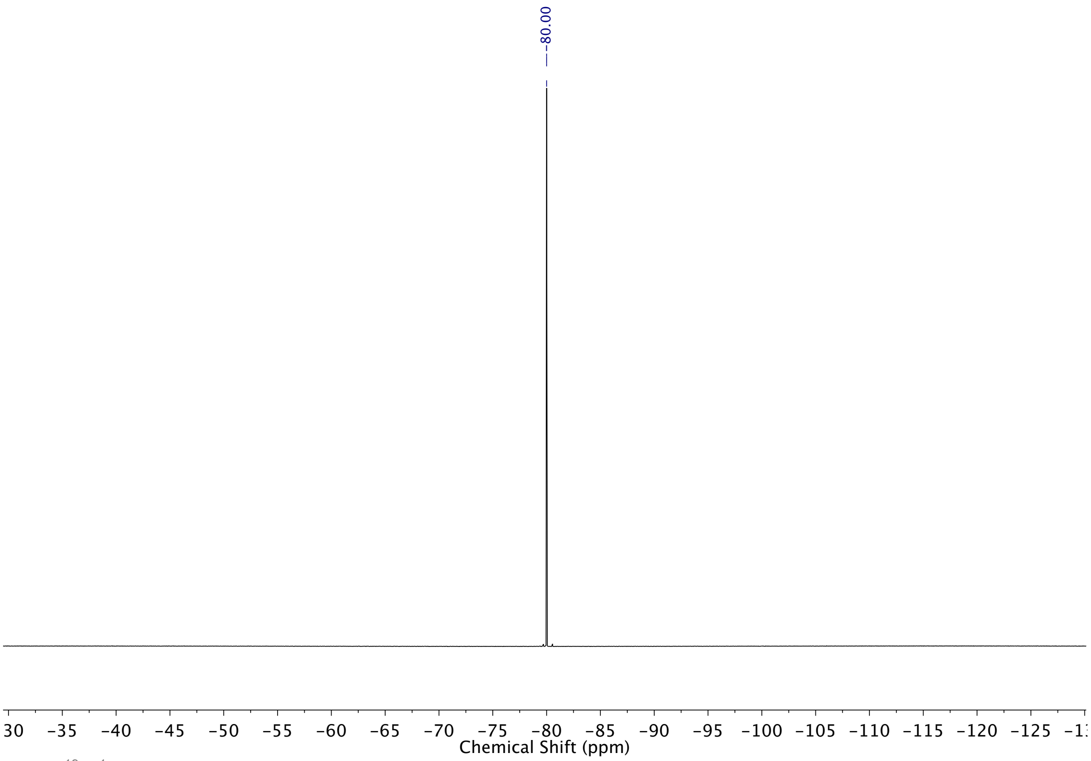

Figure S60. $^{19}F\{^1H\}$ NMR of **1-Br** (CD_3CN, 376 MHz, 298 K).

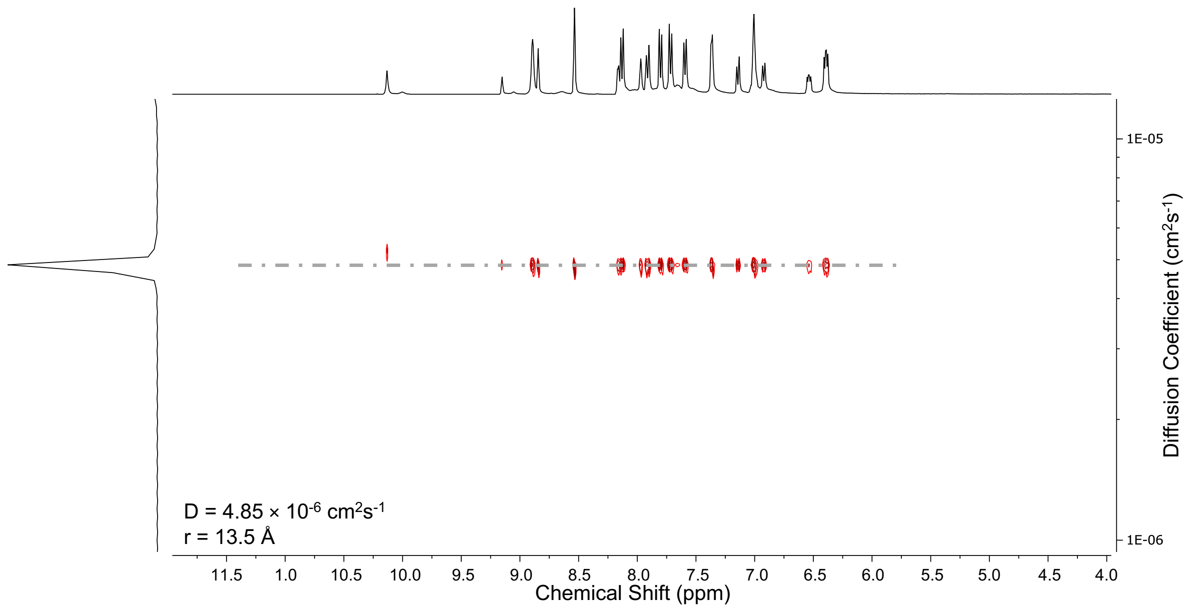

Figure S61. ^1H DOSY NMR of **1-Br** (CD_3CN, 400 MHz, 298 K).

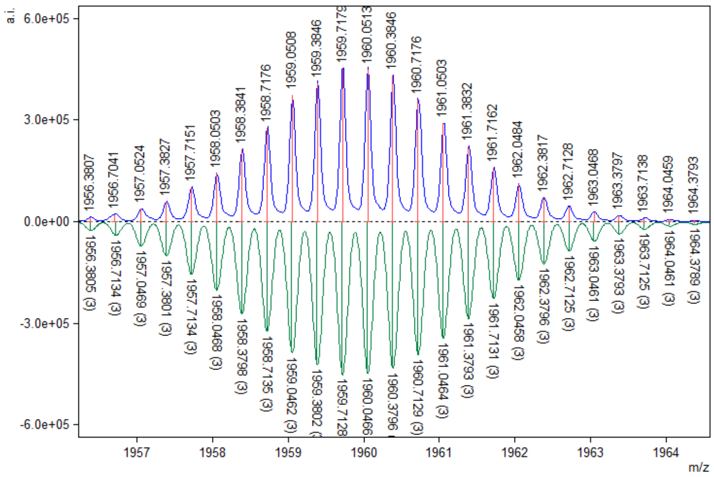

Figure S62. HR-ESI-MS [POS] **1-Br** $[Ag_{12}Br_4L_6](NTf_2)_8$ $[M-3NTf_2]^{3+}$ ($\delta_{expt-calc}$ 2.40 ppm).

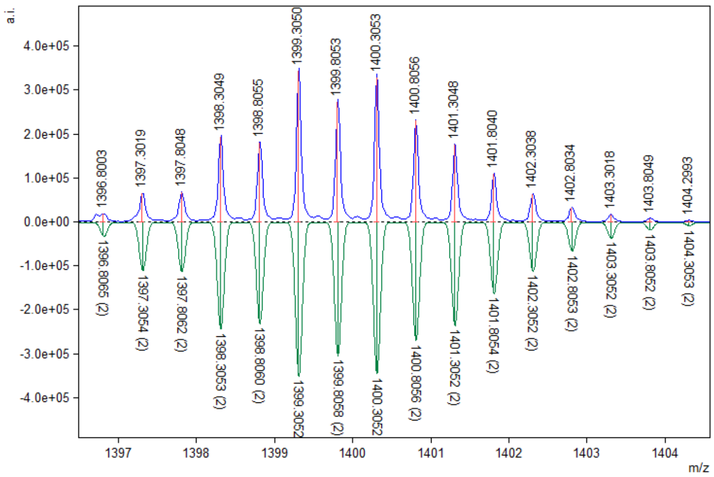

Figure S63. HR-ESI-MS [POS] **1-Br** $[Ag_6Br_2L_3](NTf_2)_4$ $[M-2NTf_2]^{2+}$ ($\delta_{expt-calc}$ 0.36 ppm).

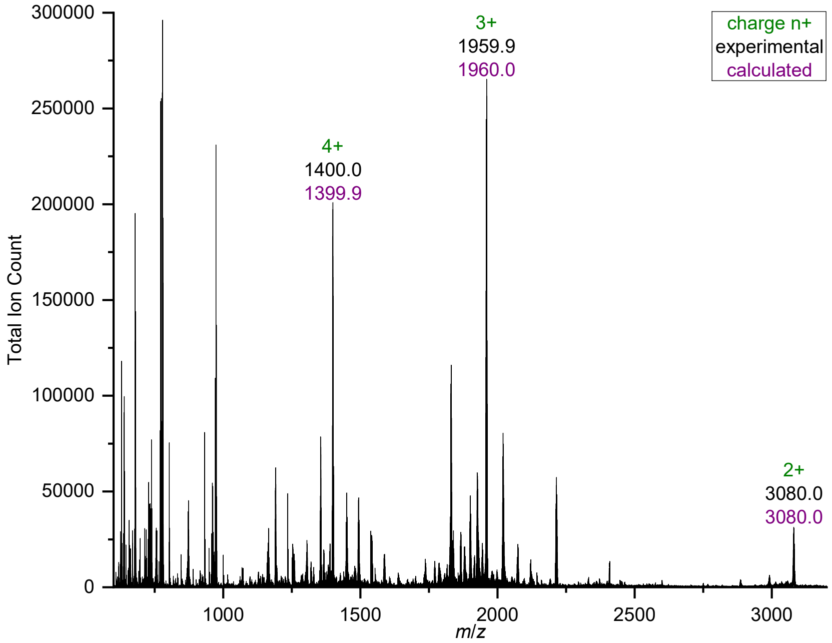

Figure S64. LR-ESI-MS [POS] **1-Br** $[Ag_{12}Br_4L_6](NTf_2)_8$ *m/z* 3080.0 $[M-2NTf_2]^{2+}$, 1960.0 $[M-3NTf_2]^{3+}$, 1400.0 $[M-4NTf_2]^{4+}$. Signals at 3080.0 and 1400.0 have contributions from the $[Ag_6Br_2L_3](NTf_2)_4$ fragment.

3.6 ^{109}Ag NMR characterization of **1-Br**

As with **1-Cl**, at 298 K the 1D ^{109}Ag NMR of **1-Br** (Figure S65) showed two distinct environments. ^{1}H-^{109}Ag HMBC (Figure S66) and selective ^{109}Ag decoupled ^{1}H NMR (Figure S67-Figure S70) confirmed assignment of the signal at 683 ppm to the 8 Ag atoms at the vertex of the grid, and 602 ppm to the 4 Ag atoms along the edges of the grid. Cooling **1-Br** to 232 K showed the same behavior and ^{1}H-^{109}Ag correlations (Figure S71).

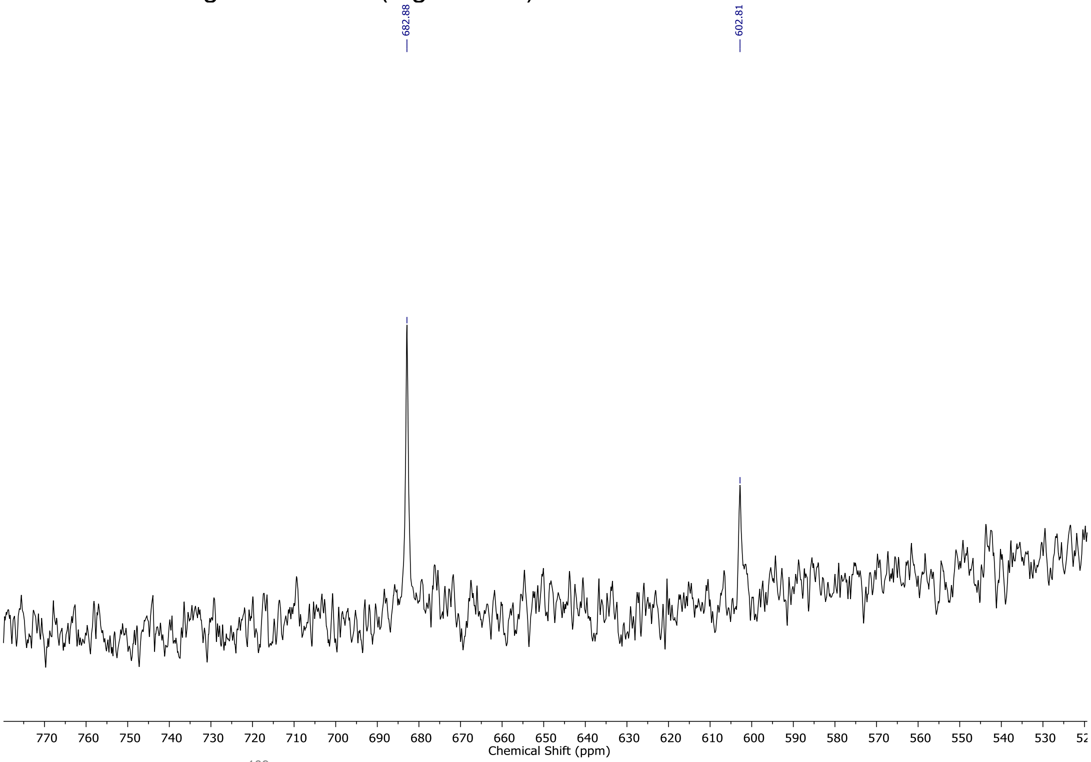

Figure S65. Baseline corrected ^{109}Ag NMR of **1-Br** (CD_3CN, 23 MHz, 298 K).

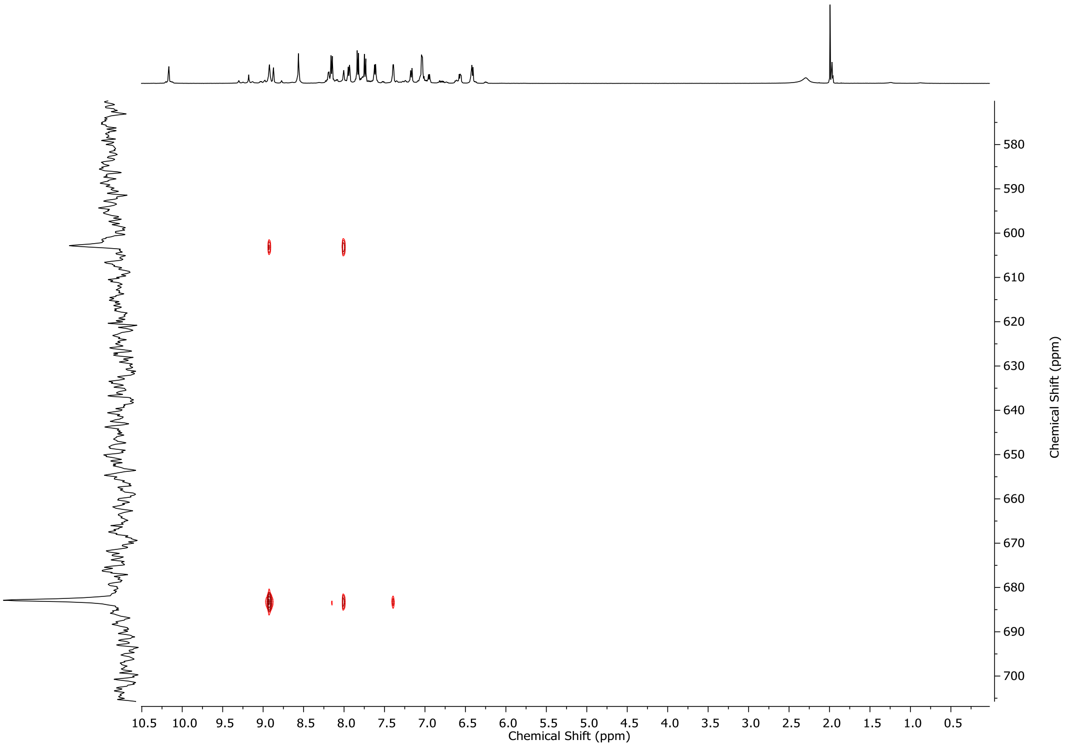

Figure S66. ^{1}H-^{109}Ag HMBC NMR of **1-Br** (CD_3CN, 298 K).

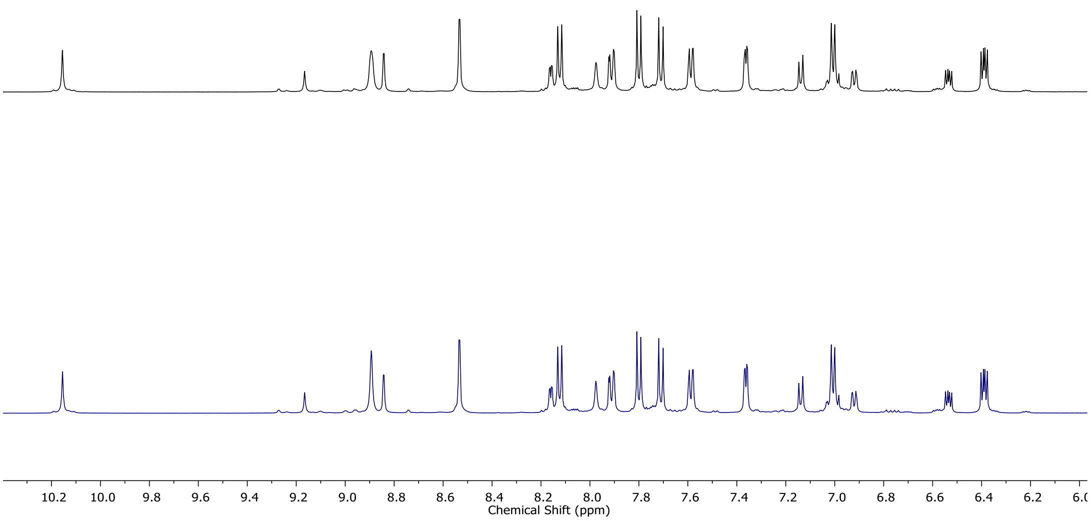

Figure S67. Stackplot of standard ^{1}H NMR (top, black) and ^{1}H NMR with ^{109}Ag decoupling at 683 ppm (bottom, blue) of **1-Br** (CD_3CN, 500 MHz, 298 K).

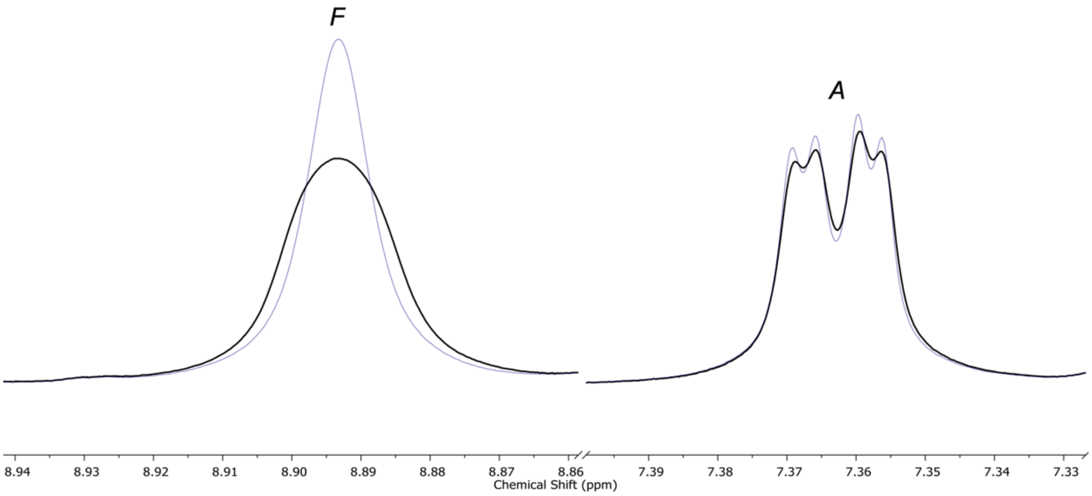

Figure S68. Superposition of standard ^{1}H NMR (black) and ^{1}H NMR with ^{109}Ag decoupling at 683 ppm (blue) of **1-Br** (CD_3CN, 500 MHz, 298 K).

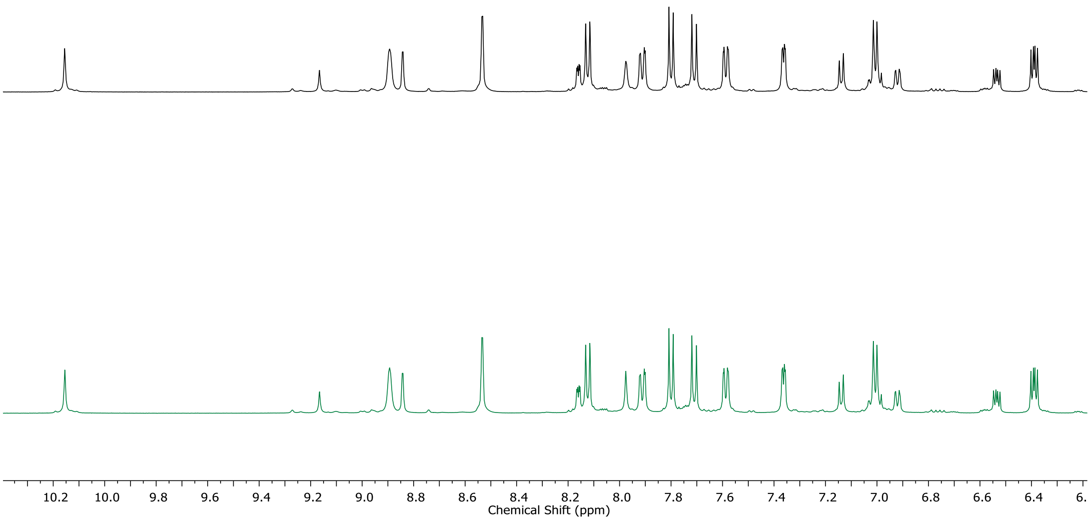

Figure S69. Stackplot of standard ^{1}H NMR (top, black) and ^{1}H NMR with ^{109}Ag decoupling at 601 ppm (bottom, green) of **1-Br** (CD_3CN, 500 MHz, 298 K).

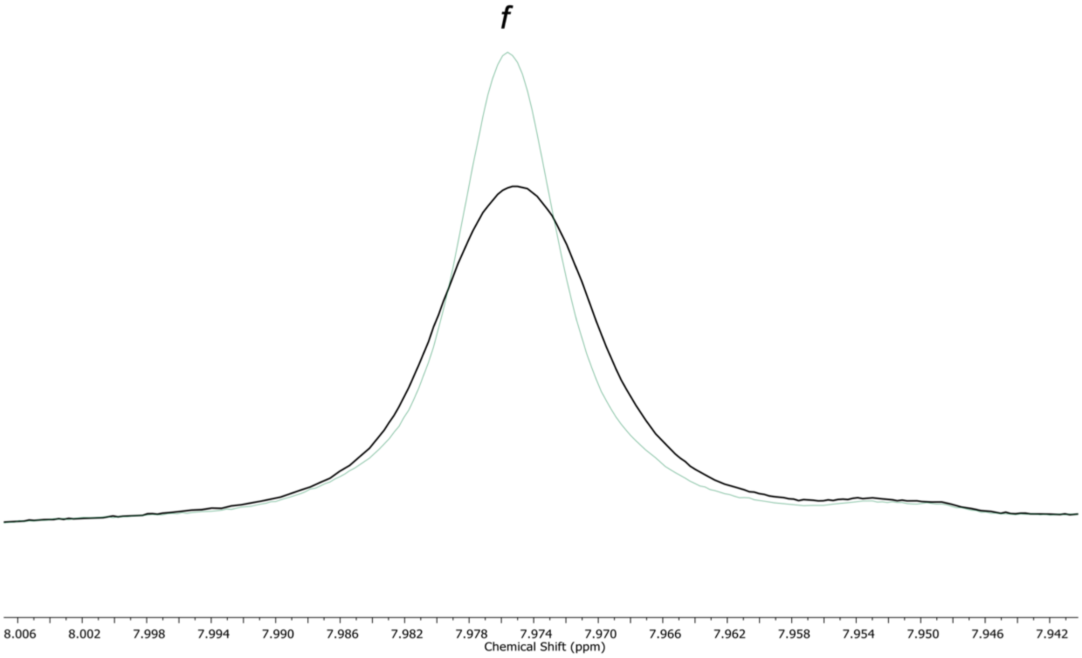

Figure S70. Superposition of standard ^{1}H NMR (black) and ^{1}H NMR with ^{109}Ag decoupling at 601 ppm (green) of **1-Br** (CD_3CN, 500 MHz, 298 K).

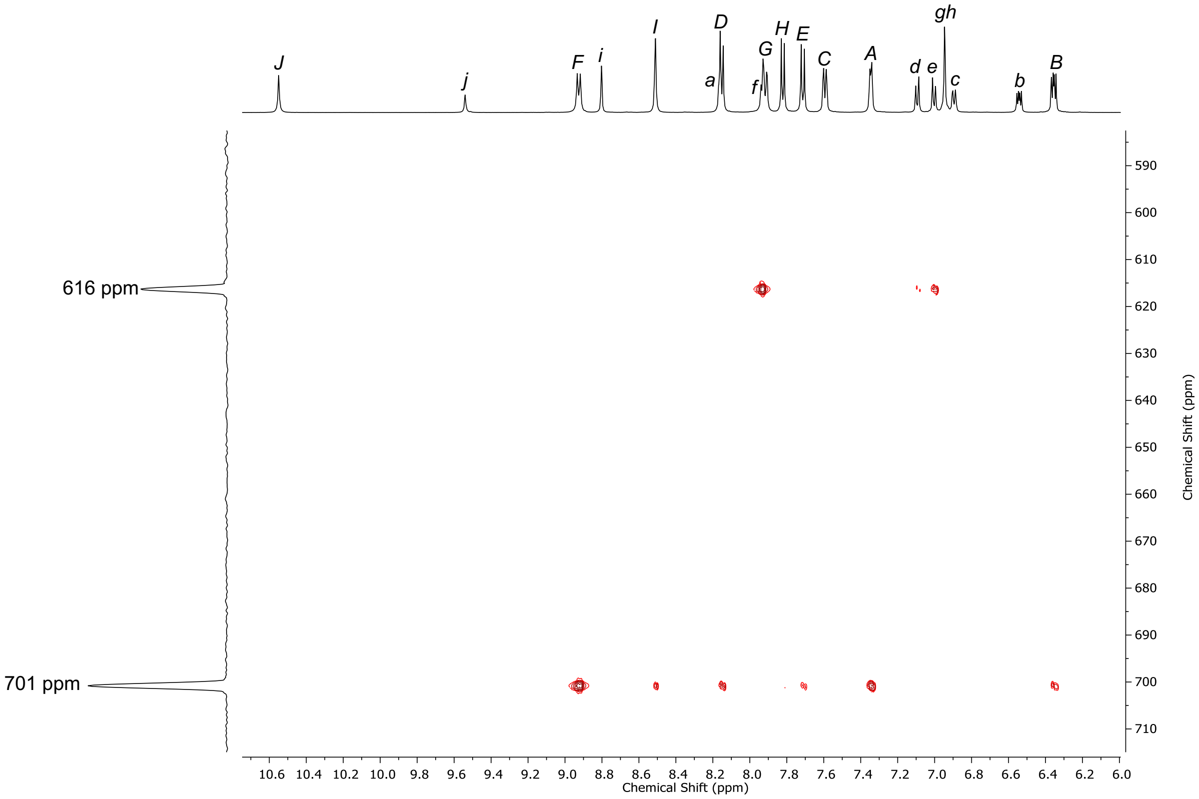

Figure S71. Enlarged ^{1}H-^{109}Ag HMBC NMR of **1-Br** (CD_3CN, 232 K).

4. ^{1}H-^{109}Ag Heteronuclear Single Quantum Coherence-Exchange Spectroscopy

4.1 General Experimental Considerations

Two-dimensional HSQC based longitudinal exchange experiments, adapted from Farrow *et al.*,[1] were acquired as a function of mixing time (τ) for each sample. This experiment incorporates a delay in which ^{109}Ag z-magnetization, enhanced by INEPT transfer from protons, is stored during a variable mixing period, and then transferred back to protons for detection. For each mixing time, the magnetization is stored in alternate scans along the +z and -z axes, and in absence of exchange the magnetization for each silver environment decays exponentially according to its own longitudinal relaxation time constant T_1. At the end of the mixing period the magnetization is transferred back to protons for detection. If there is no exchange the spectrum is as a normal HSQC, with peaks ("auto-peaks") correlating the ^{109}Ag and ^{1}H chemical shifts of each site (I_{AA} and I_{BB}). If exchange between the silver sites is present, the auto-peaks decay, and for each ^{109}Ag frequency "exchange peaks" appear at the proton frequency of the other site (I_{AB} and I_{BA}).

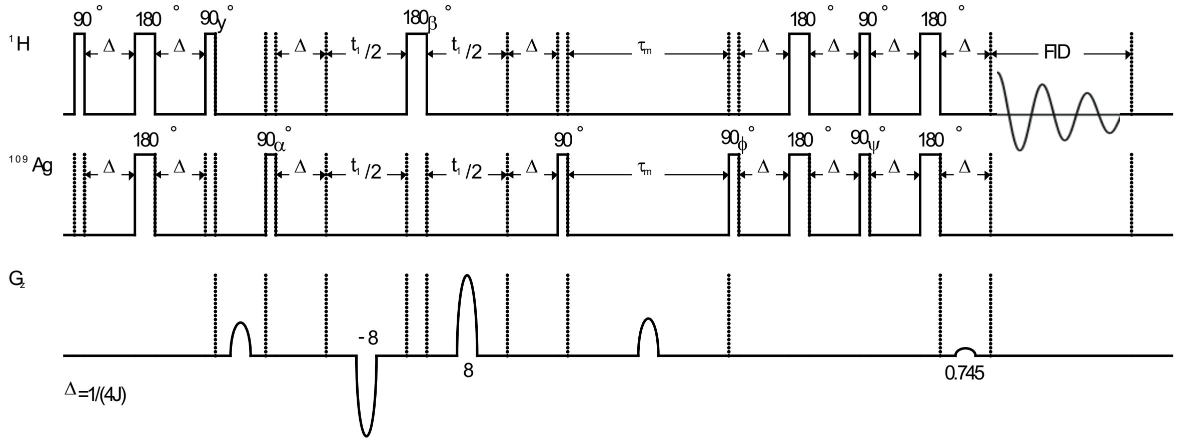

Figure S72. ^{1}H-^{109}Ag HSQC-EX pulse sequence adapted from Farrow *et al.*[1] All pulses have phase x except where indicated, phase cycles α=y, -y; β=x,x,-x,-x; ϕ=y,y,y,y,-y,-y,-y,-y; ψ=x,x,-x,-x; receiver phase= x,-x,-x,x,-x,x,x,-x. During the mixing time τ_m, 180° pulses are applied on the proton channel in the centre of each 5 ms. Quadrature detection in F1 is achieved by the echo-antiecho method with inversion of the signs of the gradients in the t_1 period in alternate FIDs. The ratio of the magnitude of these to the final gradient is given by 0.5× the ratio of the magnetogyric ratios of ^{1}H and ^{109}Ag to achieve coherence selection; actual used settings were -80:80:7.45 in percent of 50 G/cm, with durations 300 μs. Unmarked gradients suppress transverse magnetization; their amplitude is not critical. The delay Δ is nominally 1/4*J*; in this case was set corresponding to *J* = 8.4 Hz.

For **1-Cl**, **1-Br** and **1-I**, spectra were acquired at a temperature where the exchange rate was fast enough to be detectable, but slow enough that reasonable magnetization transfer using INEPT was possible *via* the small coupling constants involved. 2D experiments were acquired as a function of mixing time to allow fitting of the exchange rates. The relaxation delay used for each sample was 3× the longest proton T_1, and the center frequency in the ^{109}Ag dimension was set midway between the two signals.

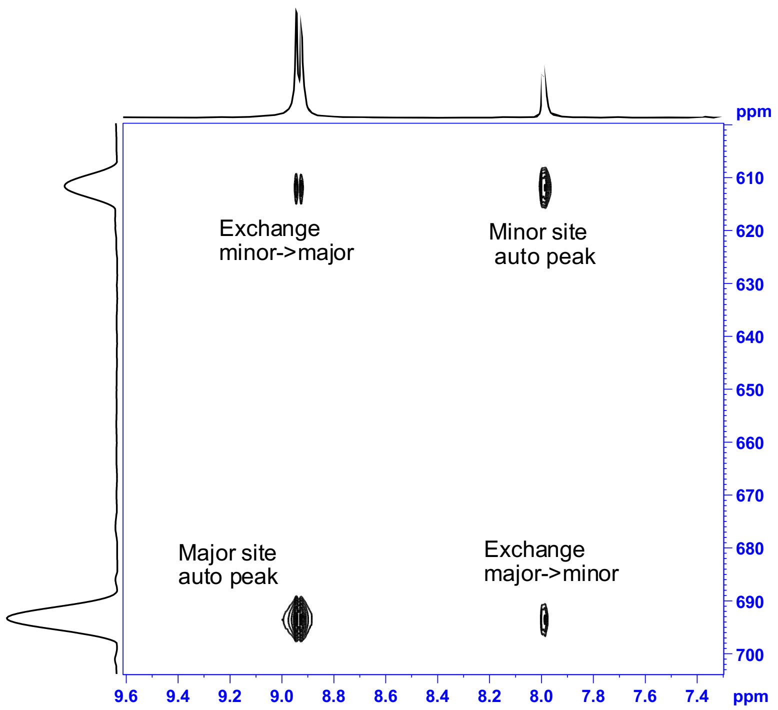

Figure S73. Example ^{109}Ag – ^{1}H HSQC exchange spectrum. The auto-peaks correspond to the normal HSQC spectrum, the exchange peaks show the exchange of silver nuclei between the two sites. Exchange and relaxation rates can be estimated from the variation of the auto-peak and exchange peak volumes as a function of mixing time. I_{AA} is the volume of the major auto-peak. I_{BB} is the volume of the minor auto-peak. I_{AB} is the volume of the major to minor exchange peak. I_{BA} is the volume of the minor to major exchange peak.

Some differences exist between the original[1] and current applications of this experiment. Firstly, in the original experiment the exchange was measured between two conformations; the relative rates of exchange are not constrained in that case. Here the exchange is between fixed sites in a molecule, and the exchange rate constants are constrained to be equal.

Secondly, exchange occurs between the minor and two major sites, so the exchange rate constant for each major site is half the value obtained from the original fitting equations.

Finally, in the original case the experiment can be assumed to be quantitative – the amount of signal transferred in the INEPT step should be the same for all sites, as the coupling constants are essentially identical, and relaxation effects are small. Here, the coupling constants are comparable with the transverse relaxation rate constants and are also different for the two sites in each molecule. The relative volumes of each of the four peaks is affected by the relaxation rates and coupling constants of the sited involved. Rather than attempting to model exactly the effect of the different spin system parameters, a scaling factor was determined for each exchange peak during the fitting process.

4.2 Details of the HSQC-Based Exchange Experiments

The most common NMR method used to probe exchange is one- or two-dimensional EXSY, however, for silver NMR this experiment is challenging due to low sensitivity. Although one-dimensional spectra were obtained (Section 3.2, 3.4 and 3.6), very long acquisition times were required (of the order of 12 hours). For exchange measurements, multiple spectra, each with a lower signal-to-noise ratio for the exchange cross peaks, would be required, leading to impractical experiment durations.

However, exchange was monitored remotely with enhanced sensitivity *via* proton detection in an HSQC based experiment, as has been applied to proteins in a ^{1}H-^{15}N exchange NMR experiment.[1] This method has been adapted to make use of the long range ^{1}H-^{109}Ag coupling present in this grid structure. Here the ^{109}Ag magnetization that undergoes exchange is enhanced by transfer from coupled protons, and after exchange is transferred back to protons for detection with enhanced sensitivity.

The signal-to-noise ratio in NMR experiments for a given number of spins is in principle proportional the product of the magnetogyric ratio (γ) of the excited isotope, and the square of the magnetogyric ratio of the detected isotope. In single pulse experiments the excited and detected nuclei are the same and so the signal to noise is proportional to γ^3.

Thus, aside from the factor of 0.48 due to the natural isotopic abundance of ^{109}Ag, a one-dimensional ^{109}Ag experiment is less sensitive than ^{1}H by a factor $[\gamma_{(1H)}/\gamma_{(109Ag)}]^3$ = 9923. In the H-X HSQC experiment, however, the population difference in the X nucleus is transferred from ^{1}H, and during the after-evolution of the X chemical shift, the magnetization is transferred back to ^{1}H for detection. Thus, in principle the only loss compared to a ^{1}H single pulse experiment is due to the natural abundance of the X nucleus, and a simple ^{1}H-^{109}Ag HSQC experiment should be 9923 times more sensitive than a 1D silver experiment.

In practice this signal enhancement is not completely realized. The noise level in the NMR experiment increases with frequency, often assumed to be proportional to the square root of the NMR frequency,[2] which would result in a factor of 4.6 lower enhancement. Pulsed field gradients can be used to ensure that only ^{1}H magnetization that has been transferred from the X nucleus is detected. This reduces T_1 noise and other artefacts, but in the echo-antiecho approach used here, the signal to noise ratio is further reduced by $\sqrt{2}$. [ref 3]

The transfer of magnetization between the ^{1}H and X nuclei in the HSQC experiment is also generally imperfect. The transfer delay may not be exactly matched to the theoretical optimum value of 1/2*J*, if the H-X coupling constants vary for the signals of interest, as is the case here. There is also loss due to transverse relaxation of ^{1}H if the required delays are not short compared to the ^{1}H T_2, which is likely if the coupling constants are small. There can also be loss due to evolution of ^{1}H-^{1}H couplings, if present.

Finally, the relative sensitivity of ^{1}H and X detected experiments depends on the arrangement of the detector coils in the probe. In the probe used in this study, the inner coil is tuned to X nuclei and the outer one to ^{1}H, which results in a factor of 2 relative signal loss for the HSQC vs 1D ^{109}Ag. However, all these factors together result in a loss of less than two orders of magnitude, meaning the expected gain in signal-to-noise is still of the order of hundreds.

In order for this experiment to be of practical use, three criteria must be met. First, there must be a measurable H-X coupling for all the exchanging X nucleus sites of interest, and these couplings must be similar enough that sufficient signal can be obtained for all sites for some value of the set transfer delay in the HSQC part of the sequence. This requirement may preclude detection of quadrupolar nuclei such as ^{63}Cu, which generally undergo rapid T_1 relaxation. Second, as in all exchange experiments, the X nucleus T_1 must be long enough compared to the inverse of the exchange rate that significant cross peak build up is possible. Third, not all spin-active nuclei are conveniently accessible using standard spectrometers. For example, ^{197}Au is 100% abundant, but its low resonance frequency means it is only accessible using specially built probes.

4.3 Optimization of the Experimental Conditions

To obtain the best sensitivity, some experimental parameters must be optimized.

First, the optimum relaxation delay in the experiment relates to the T_1 of the ^1H nuclei involved; for maximum sensitivity this should be 1.3 times the longest T_1 value. The spin system does not need to be fully relaxed, because the properties of the experiment mean that the absolute amount of X nucleus magnetization present at the start of the mixing period only needs to be the same in all experiments. The T_1 value of the protons of interest can conveniently and rapidly be measured by a standard inversion-recovery experiment.

Second, the transfer delay must be optimized. With the pulse program used here, the delays are calculated from the set value of the nominal coupling constant; this can be set somewhere in the range of the observed heteronuclear couplings and then in principle optimized for maximum signal intensity, with the mixing time set to a small value. This optimization can be carried out in a one-dimensional experiment without evolution of the X nucleus frequency if the signal-to-noise ratio is sufficient.

Third, the center frequency and resolution in the indirect dimension must be set so that the X nucleus signals are within the excitation bandwidth of the pulses and the peaks for the different X nucleus environments are fully resolved. For an X 180° pulse of 55 μs (the longest value used here) inversion is >90% effective for a range of frequencies ± 2000 Hz from the center of the spectrum, and in all cases here the ^{109}Ag signals are within this range. The bandwidth is inversely proportional to the length of the 180° pulse, so should be checked according to the achievable pulse length. In the case of larger chemical shift ranges it may be impossible to satisfy this criterion; in this case in principle multiple separate experiments could be acquired, each centered on one X nucleus site.

Finally, the set of mixing times used must be set. In this implementation each mixing time is recorded as a separate 2D experiment. The mixing time is set by the parameter d7 plus a fixed delay during which a gradient pulse is applied to remove any spurious transverse ^1H magnetization (10 ms in the sequence used here). Test experiments with low signal-to-noise ratios can be acquired for various mixing times to find the approximate signal maximum of the cross peaks, then a larger number of experiments with better signal-to-noise acquired with more closely spaced mixing times for values beyond this point and with longer spacing for mixing times up to the point where minimal signal remains.

4.4 NMR Determination of Sample Concentration

The concentration of the complex in the NMR samples was determined by quantitative NMR using an external reference sample of 0.1% ethylbenzene (the manufacturer's sensitivity standard). Spectra of the reference and of the samples of the complex were acquired under identical conditions and the ratio of integrals was used to determine the concentration of the complex. Because the samples have significant ionic strength the PULCON method was used to correct for the effect of ionic strength on the detected signal intensity.[4] The concentration of the ethylbenzene standard was 9.400 mM. The concentration of **1-Cl** was measured as 19.5 mM, **1-Br** as 15.3 mM, and **1-I** as 15.8 mM. After dilution, the concentration of **1-Cl** was measured again as 3.04 mM for the concentration control studies. Concentrations are reported to the nearest mM below.

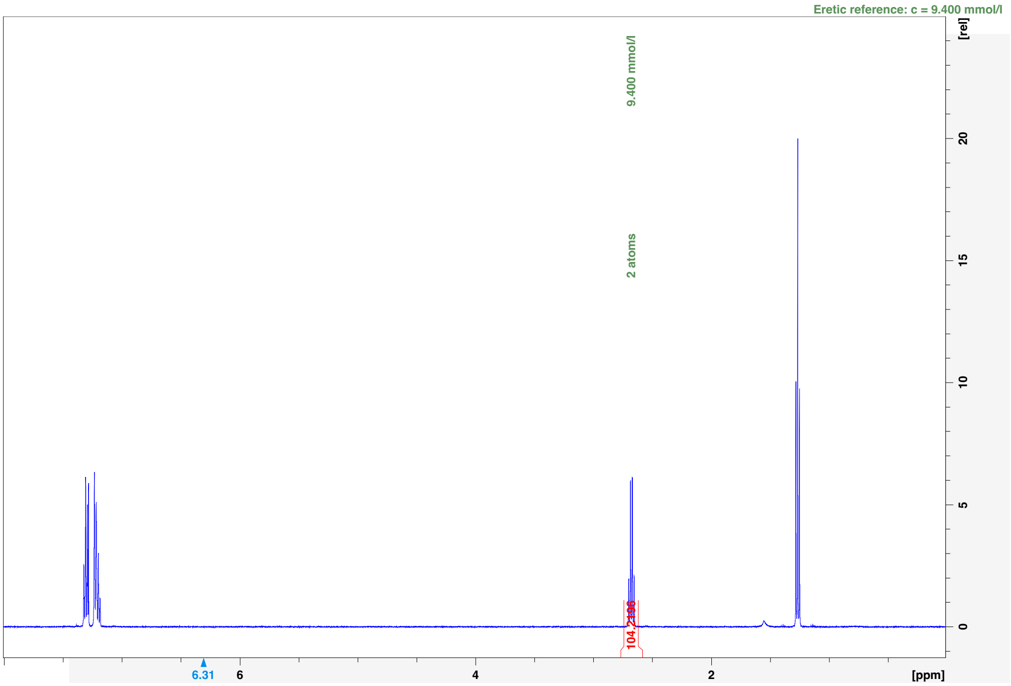

Figure S74. ^{1}H NMR spectrum of ethylbenzene **r**eference at 9.400 mM concentration (CD_3CN, 500 MHz).

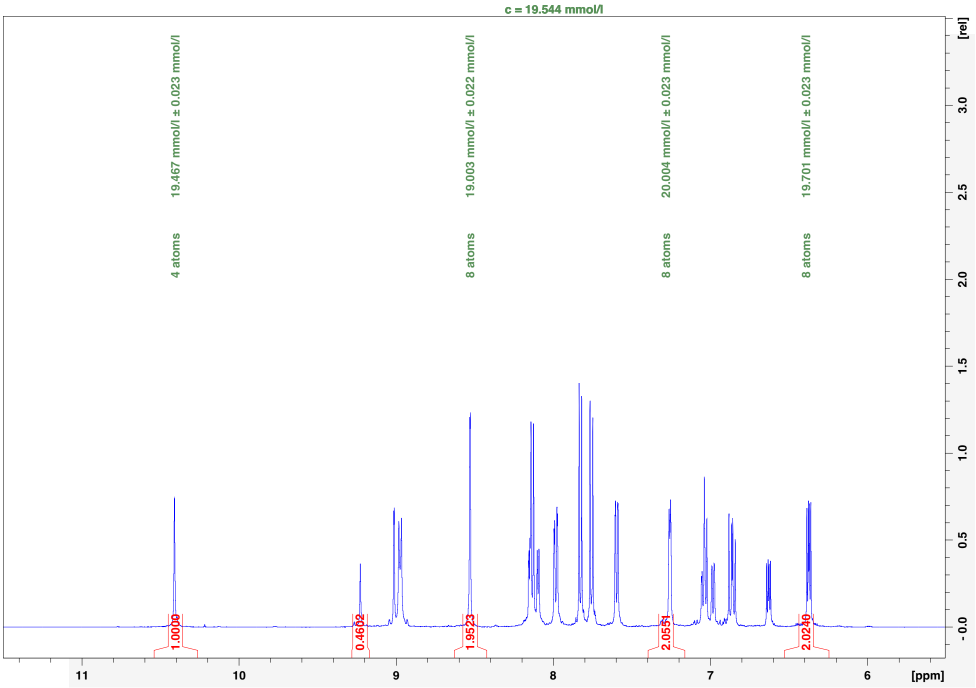

Figure S75. ^{1}H NMR spectrum of **1-Cl** to determine its concentration to be 19.5 mM (CD_3CN, 500 MHz).

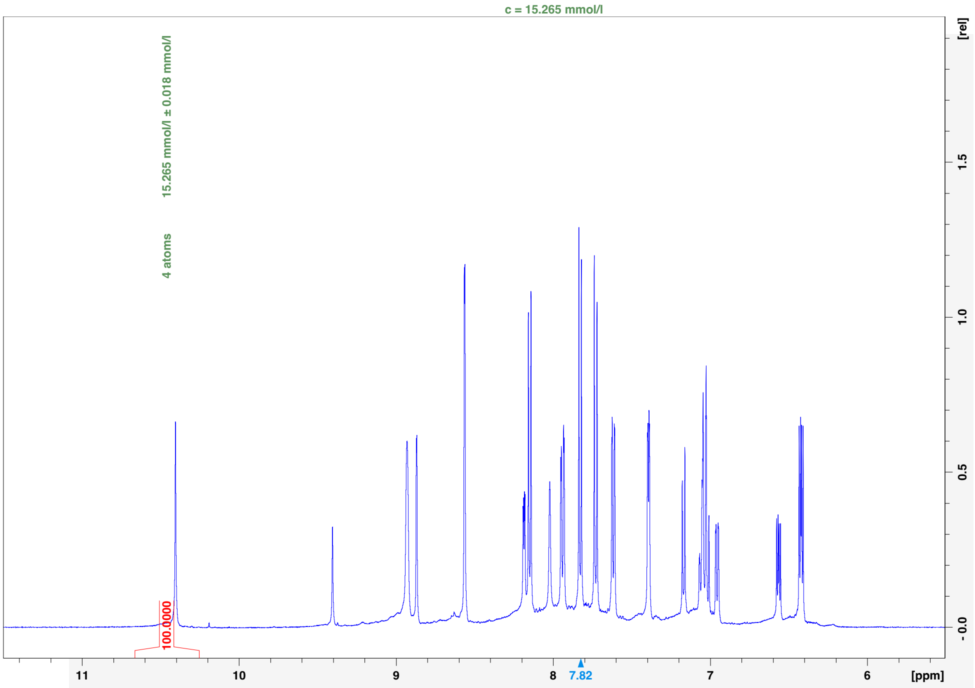

Figure S76. ^{1}H NMR spectrum of **1-Br** to determine its concentration to be 15.3 mM (CD_3CN, 500 MHz).

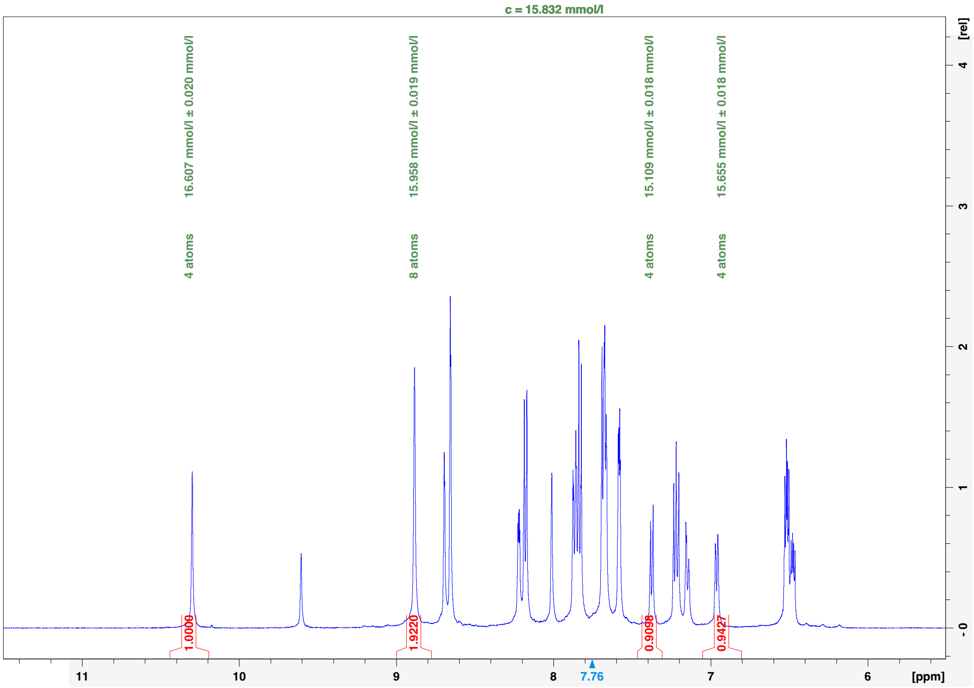

Figure S77. ^{1}H NMR spectrum of **1-I** to determine its concentration to be 15.8 mM (CD_3CN, 500 MHz).

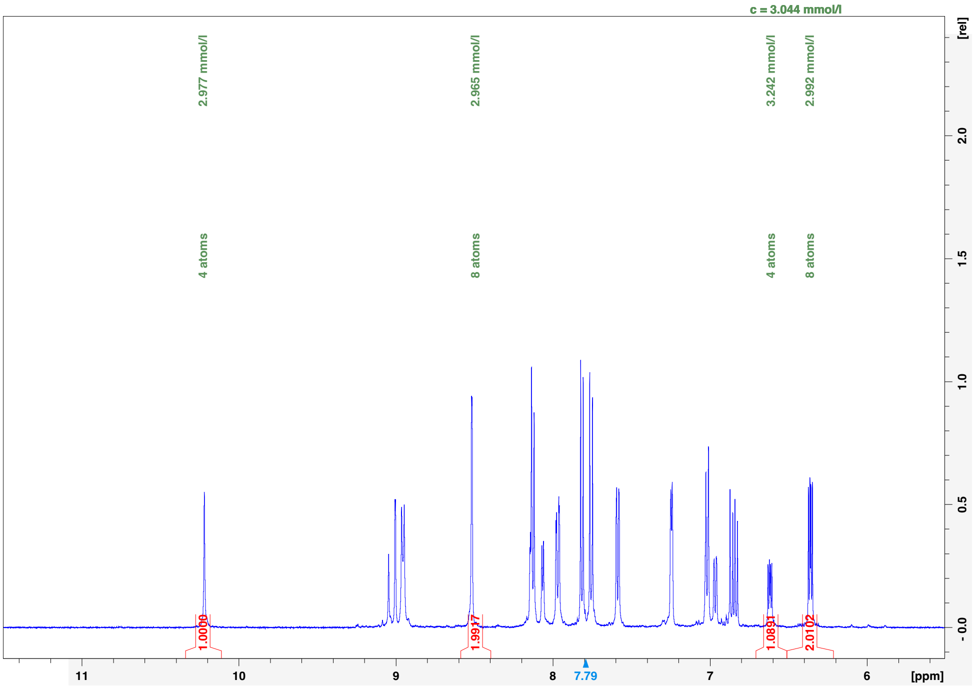

Figure S78. ^{1}H NMR spectrum of **1-Cl** to determine its concentration to be 3.0 mM for the dilution control experiment (CD_3CN, 500 MHz).

4.5 Mathematical Description of Signal Decay

The auto-peak volumes for sites A (major silver site) and B (minor silver site) were fitted to equations:

$$I_{AA}(\tau) = I_{AA}(0)\,(-(\lambda_2 - a_{11})\,e^{-\lambda_1\tau} + (\lambda_1 - a_{11})\,e^{-\lambda_2\tau})/(\lambda_1 - \lambda_2)$$

$$I_{BB}(\tau) = I_{BB}(0)\,(-(\lambda_2 - a_{22})\,e^{-\lambda_1\tau} + (\lambda_1 - a_{22})\,e^{-\lambda_2\tau})/(\lambda_1 - \lambda_2)$$

Where $I_{AA}(0)$ and $I_{BB}(0)$ are the calculated initial peak volumes at 0 ms.

While the volumes for the exchange peaks corresponding to magnetization transfer from silver site A to B ($I_{AB}(\tau)$) and from B to A ($I_{BA}(\tau)$) are given by:

$$I_{AB}(\tau) = C_{AB}\,(a_{21}\,e^{-\lambda_1\tau} - a_{21}\,e^{-\lambda_2\tau})/(\lambda_1 - \lambda_2)$$

And

$$I_{BA}(\tau) = C_{BA}\,(a_{12}\,e^{-\lambda_1\tau} - a_{12}\,e^{-\lambda_2\tau})/(\lambda_1 - \lambda_2)$$

Where:

$$\lambda_1 = \frac{1}{2}\left\{(a_{11} + a_{22}) + [(a_{11} - a_{22})^2 + 4k^2]^{1/2}\right\}$$

$$\lambda_2 = \frac{1}{2}\left\{(a_{11} + a_{22}) - [(a_{11} - a_{22})^2 + 4k^2]^{1/2}\right\}$$

$$a_{11} = R_A + k$$

$$a_{12} = a_{21} = k$$

$$a_{22} = R_B + k$$

R_A and R_B are the longitudinal relaxation rates of ^{109}Ag in sites A and B, and *k* is the total exchange rate for magnetization transferring from site A to B (which is equal to the exchange rate from site B to A), i.e. twice the exchange rate for each individual major site.

The factors C_{AB} and C_{BA} replace $I_{AA}(0)$ and $I_{BB}(0)$ in the original equations and account for the non-quantitative transfer and are fitted to the data.

τ is the mixing time (in seconds).

For each sample, the relaxation rates and exchange rate were obtained by minimizing the sum of squared errors between the experimental data for all four peaks, and the theoretical peak volumes, simultaneously, by varying the rate constants, the initial volumes $I_{AA}(0)$ and $I_{BB}(0)$, and the coefficients C_{AB} and C_{BA}.

In order to allow a gradient pulse in the mixing time, the minimum mixing time is 10 ms; the initial peak volumes are obtained from the fitting. Peak volumes and errors were determined using Dynamic Centre. Matlab was used to fit the decay and calculate errors in the fit parameters (Table S1).

Table S1. Optimized Parameters used for line fitting and rate constant determination of **1-Cl** [20 mM], **1-Cl** [3 mM], **1-Br** [15 mM], and **1-I** [16 mM]. A 95% confidence interval (±2σ) is reported in square brackets for all fitted parameters.

Parameter		**1-Cl [20 mM]**	**1-Cl [3 mM]**	**1-Br [15 mM]**	**1-I [16 mM]**
Temp. / K	Measurement temperature	298.0	298.0	263.0	243.0
$I_{AA}(0)$	Initial volume of major auto-peak	64205406.89 [62434346.70 - 65976467.08]	84096822.38 [81629883.26 - 86563761.51]	61814858.64 [60545706.75 - 63084010.53]	316327274.16 [313187399.58 - 319467148.75]
$I_{BB}(0)$	Initial volume of minor auto-peak	56295047.90 [54359774.70 - 58230321.10]	59922762.94 [57525148.69 - 62320377.20]	37084484.34 [35723861.78 - 38445106.91]	130307372.04 [127356671.92 - 133258072.17]
C_{AB}	Volume after magnetization transfer (major-to-minor)	0.922731 [0.775058 - 1.070404]	0.836780 [0.694706 - 0.978853]	0.652173 [0.578546 - 0.725800]	0.608815 [0.567171 - 0.650460]
C_{BA}	Volume after magnetization transfer (minor-to-major)	1.137748 [0.957859 - 1.317637]	1.139701 [0.945191 - 1.334210]	0.791481 [0.691973 - 0.890989]	1.166802 [1.079233 - 1.254371]
R_A / s^{-1}	^{109}Ag longitudinal relaxation rate in environment A	1.004097 [0.806785 - 1.201410]	1.705357 [1.452514 - 1.958199]	0.433677 [0.333262 - 0.534092]	1.126572 [1.012194 - 1.240950]
R_B / s^{-1}	^{109}Ag longitudinal relaxation rate in environment B	2.485391 [2.228655 - 2.742127]	2.669191 [2.327767 - 3.010616]	2.216375 [2.034160 - 2.398590]	3.889747 [3.655025 - 4.124469]
a_{11} / s^{-1}	$a_{11} = R_A + k$	3.33494	4.596858	2.248639	4.507536
a_{12} / s^{-1}	$a_{12} = a_{21} = - k$	-2.330843	-2.891501	-1.814962	-3.380964
a_{22} / s^{-1}	$a_{22} = R_B + k$	4.816234	5.560692	4.031337	7.270711
λ_1 / s^{-1}	See equations above	6.521274443	8.01000524	5.162014237	9.541479122
λ_2 / s^{-1}	See equations above	1.629899557	2.147399675	1.117961763	2.236767878
k / s^{-1}	Exchange rate constant	2.330843 [1.884874 - 2.776813]	2.891501 [2.299191 - 3.483810]	1.814962 [1.564221 - 2.065703]	3.380964 [3.106509 - 3.655420]

The rate constants for total magnetization exchange can be extracted from the fit parameters.

$k_{\mathbf{1\text{-}Cl}}^{298\text{ K}}$ = 2.33(22) Hz, relative σ of **±9%** (measured at 20 mM concentration)
$k_{\mathbf{1\text{-}Br}}^{263\text{ K}}$ = 1.81(13) Hz, relative σ of **±7%** (measured at 15 mM concentration)
$k_{\mathbf{1\text{-}I}}^{243\text{ K}}$ = 3.38(14) Hz, relative σ of **±4%** (measured at 16 mM concentration)
$k_{\mathbf{1\text{-}Cl}}^{298\text{ K}}$ = 2.89(30) Hz, relative σ of **±10%** (measured at 3 mM concentration)

To determine the effect of concentration and to further rule out silver exchange through dissociation, **1-Cl** was diluted six-fold (to 3 mM), and the ^{1}H-^{109}Ag HSQC-EX NMR experiments were repeated. The lower signal-to-noise at 3 mM (compared to 20 mM) resulted in a slightly poorer fit. At 3 mM, $k_{\mathbf{1\text{-}Cl}}^{298\text{ K}}$ increased slightly, however the value determined at 20 mM is within the 95% confidence window of the 3 mM value. The good agreement between the 3 mM and 20 mM values of $k_{\mathbf{1\text{-}Cl}}^{298\text{ K}}$ confirm that concentration has minimal effect on rate constants, supporting an intramolecular silver exchange mechanism.

4.6 Exchange Measurements on **1-I** at 243 K

At 243 K, the silver exchange behavior in **1-I** was observable. The data were fitted to the mathematical description (Section 4.5), with a total magnetization transfer rate constant ($k_{1\text{-}I}^{243\ K}$ = 3.38(14) Hz). The ^{1}H-^{109}Ag HSQC-EX spectra below show a decrease in auto-peak volume and increase in exchange peak volume as mixing time increases, followed by a decrease in exchange volume at high mixing times due to longitudinal relaxation effects.

Table S2 shows the auto- and exchange peak volumes at each mixing times. Each row of the table corresponds to a ^{1}H-^{109}Ag HSQC-EX NMR spectrum (Figure S79-S91). These data are fitted (Figure S92-S94) to the equations detailed in section 4.5, optimized over the four signals, to extract the signal decay parameters and the a magnetization transfer rate constant $k_{1\text{-}I}^{243\ K}$.

This experiment was performed at 16 mM concentration in CD_3CN at 243 K.

Table S2. Volume of auto-peak and exchange peaks in **1-I** [16 mM] at 243 K.

Mixing Time (τ / s)	Major auto-peak (I_{AA})	Minor auto-peak (I_{BB})	Major to minor exchange peak (I_{AB})	Minor to Major exchange peak (I_{AB})
0.010	308145512 (± 31077)	123465207 (± 31013)	5527466 (± 31077)	3776342 (± 34428)
0.030	276580788 (± 30776)	102954568 (± 30713)	15466420 (± 30776)	10983732 (± 34095)
0.050	250813821 (± 32272)	92885066 (± 32206)	22214193 (± 32272)	18717285 (± 35752)
0.080	227078735 (± 31202)	76041376 (± 31138)	31704767 (± 31202)	24213066 (± 34567)
0.120	205151188 (± 29919)	65554831 (± 29583)	37529141 (± 30297)	29294661 (± 32836)
0.160	174289210 (± 29702)	44238886 (± 29641)	43182514 (± 29702)	34949672 (± 32905)
0.210	149798659 (± 31609)	37051378 (± 31545)	43519706 (± 31609)	35507007 (± 35018)
0.310	114159911 (± 30684)	23325621 (± 30621)	40594279 (± 30684)	31444839 (± 33993)
0.410	88202084 (± 31456)	16528174 (± 31392)	35517649 (± 31456)	27934837 (± 34848)
0.610	56801371 (± 29905)	9208317 (± 29844)	24843677 (± 29905)	18601666 (± 33130)
0.810	35362104 (± 31688)	4535846 (± 31623)	14626741 (± 31688)	12281400 (± 35105)
1.010	23843585 (± 31333)	3359239 (± 31269)	10422748 (± 31333)	7242885 (± 34712)
1.210	14317304 (± 31333)	2762599 (± 31269)	6299807 (± 31333)	4419630 (± 34712)

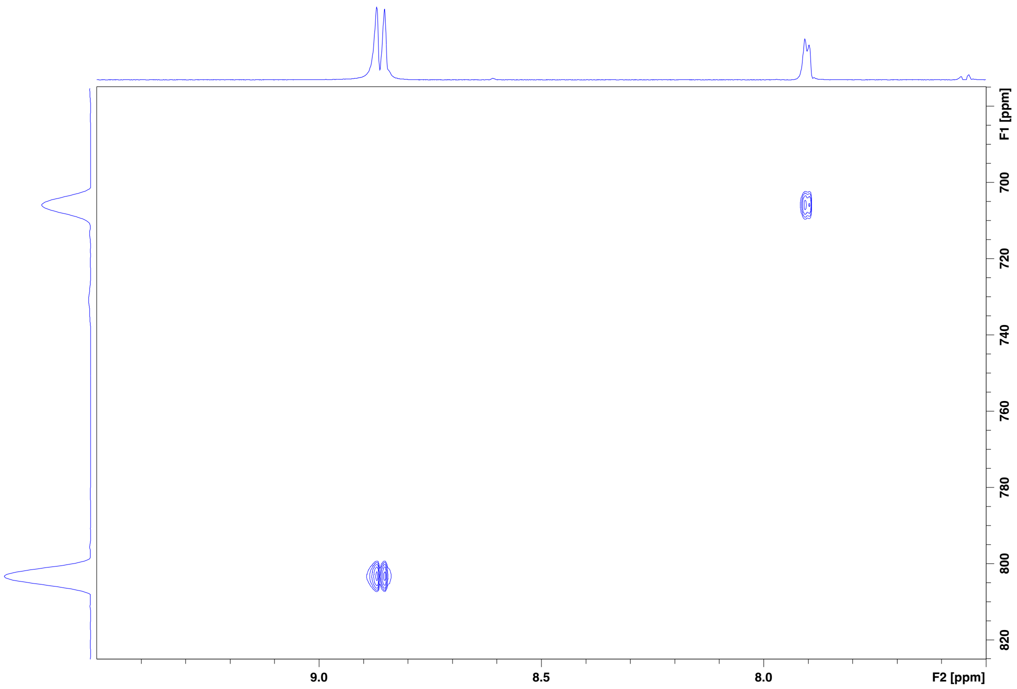

Figure S79. ^{1}H-^{109}Ag HSQC-EX NMR spectrum of **1-I** with mixing delay τ = 10 ms (500 MHz, 16 mM in CD_3CN, 243 K). Auto-peak integrals I_{AA} = 308145512, I_{BB} = 123465207. Exchange peak integrals I_{AB} = 5527466, I_{BA} = 3776342.

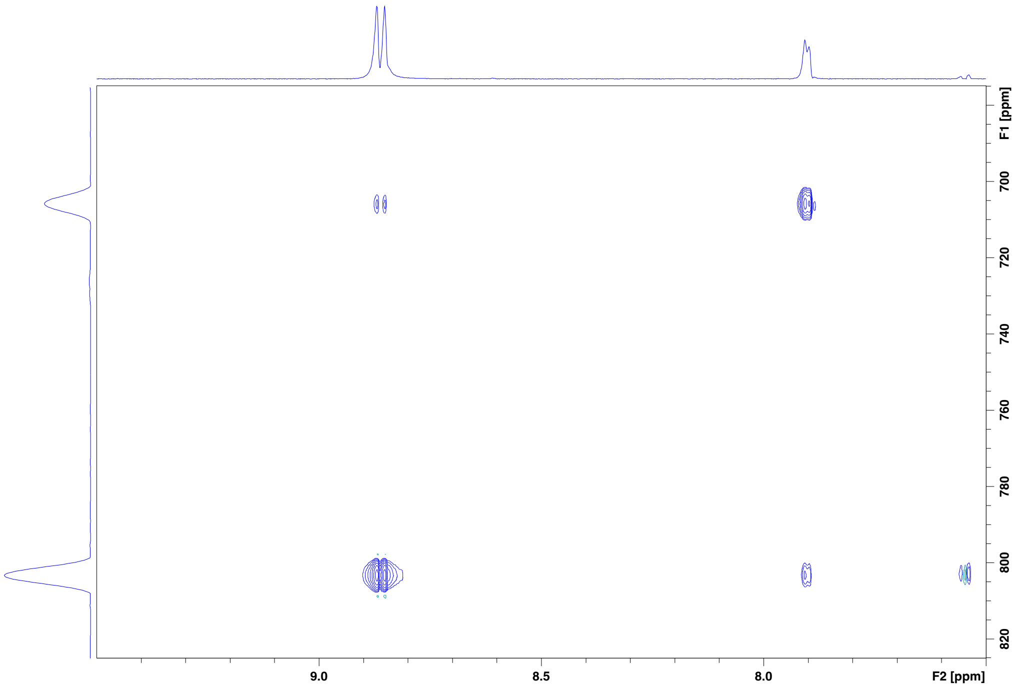

Figure S80. ^{1}H-^{109}Ag HSQC-EX NMR spectrum of **1-I** with mixing delay τ = 30 ms (500 MHz, 16 mM in CD_3CN, 243 K). Auto-peak integrals I_{AA} = 276580788, I_{BB} = 102954568. Exchange peak integrals I_{AB} = 15466420, I_{BA} = 10983732.

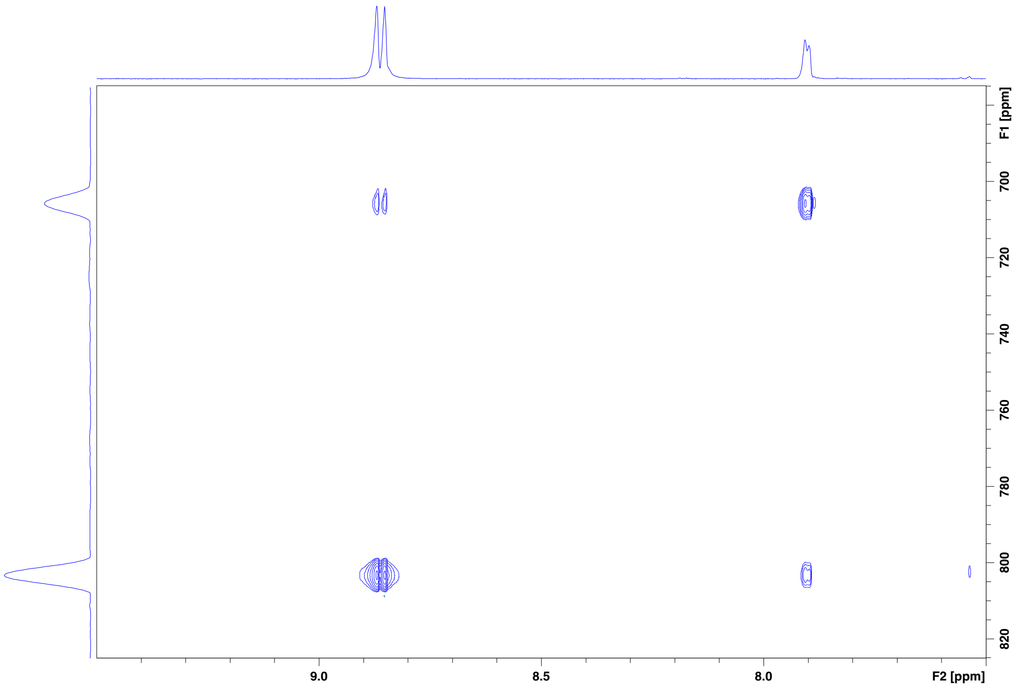

Figure S81. ^{1}H-^{109}Ag HSQC-EX NMR spectrum of **1-I** with mixing delay τ = 50 ms (500 MHz, 16 mM in CD_3CN, 243 K). Auto-peak integrals I_{AA} = 250813821, I_{BB} = 92885066. Exchange peak integrals I_{AB} = 22214193, I_{BA} = 18717285.

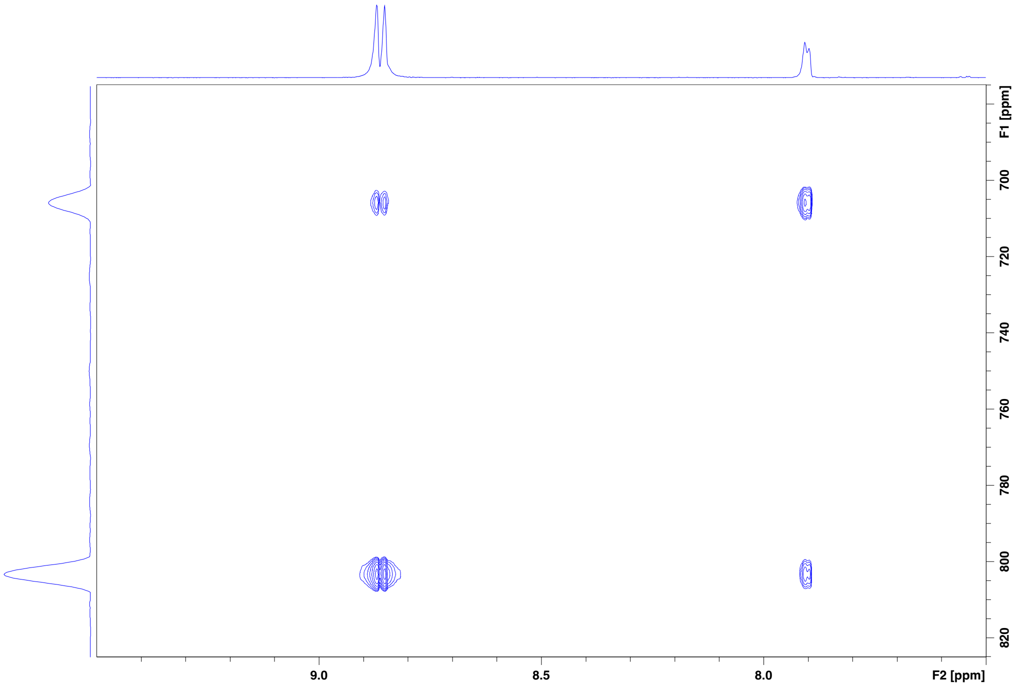

Figure S82. ^{1}H-^{109}Ag HSQC-EX NMR spectrum of **1-I** with mixing delay τ = 80 ms (500 MHz, 16 mM in CD_3CN, 243 K). Auto-peak integrals I_{AA} = 227078735, I_{BB} = 76041376. Exchange peak integrals I_{AB} = 31704767, I_{BA} = 24213066.

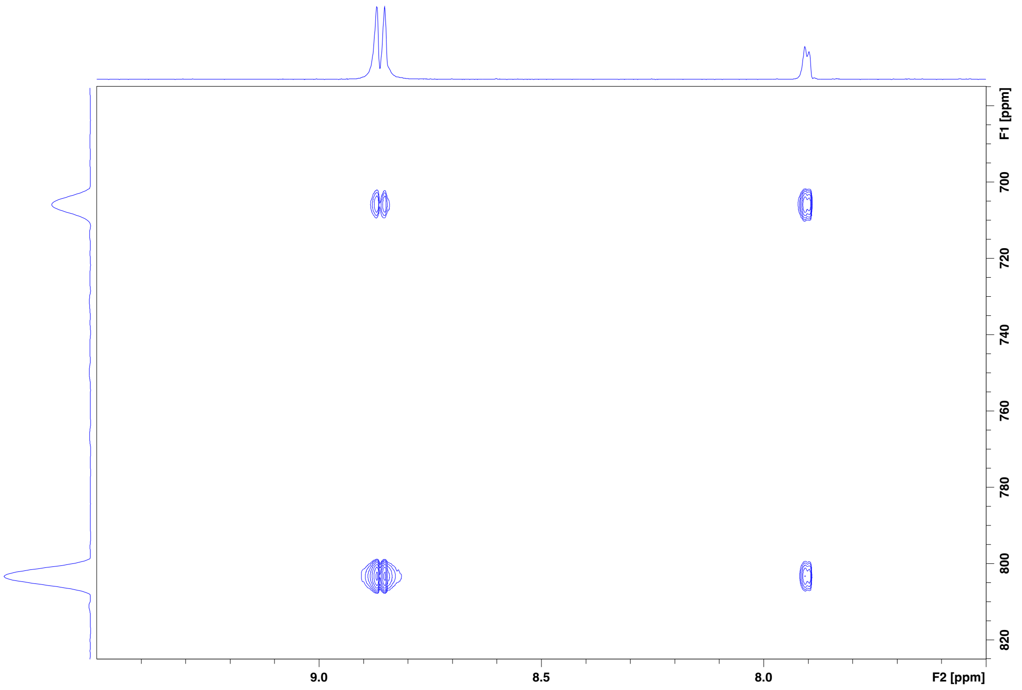

Figure S83. ^{1}H-^{109}Ag HSQC-EX NMR spectrum of **1-I** with mixing delay τ = 110 ms (500 MHz, 16 mM in CD_3CN, 243 K). Auto-peak integrals I_{AA} = 205151187, I_{BB} = 65554831. Exchange peak integrals I_{AB} = 37529141, I_{BA} = 29294661.

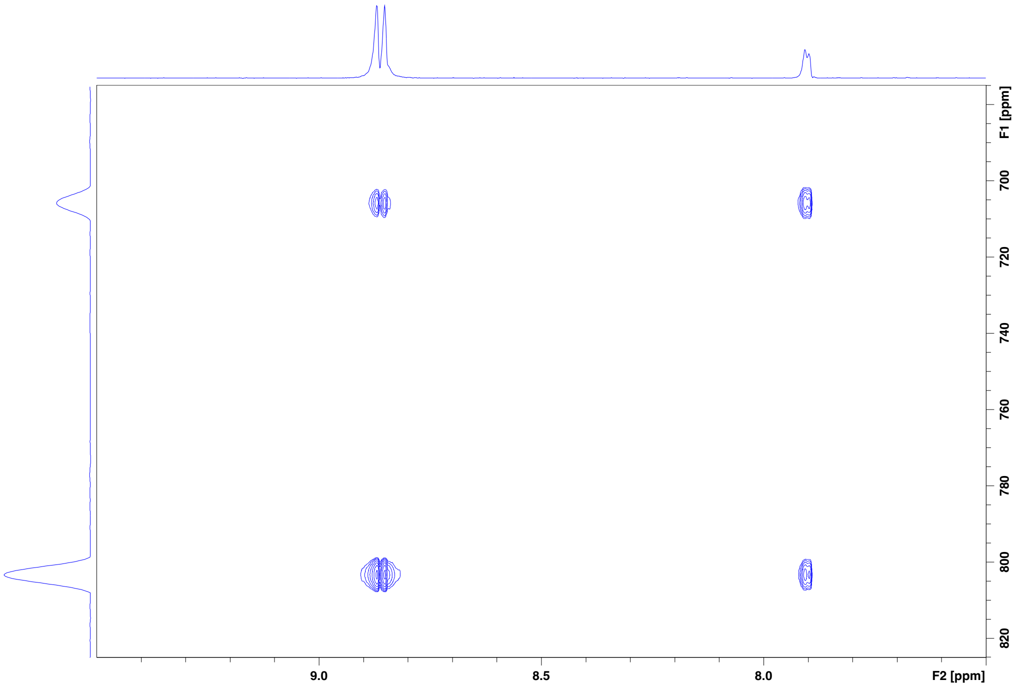

Figure S84. ^{1}H-^{109}Ag HSQC-EX NMR spectrum of **1-I** with mixing delay τ = 160 ms (500 MHz, 16 mM in CD_3CN, 243 K). Auto-peak integrals I_{AA} = 174289210, I_{BB} = 44238886. Exchange peak integrals I_{AB} = 43182514, I_{BA} = 34949672.

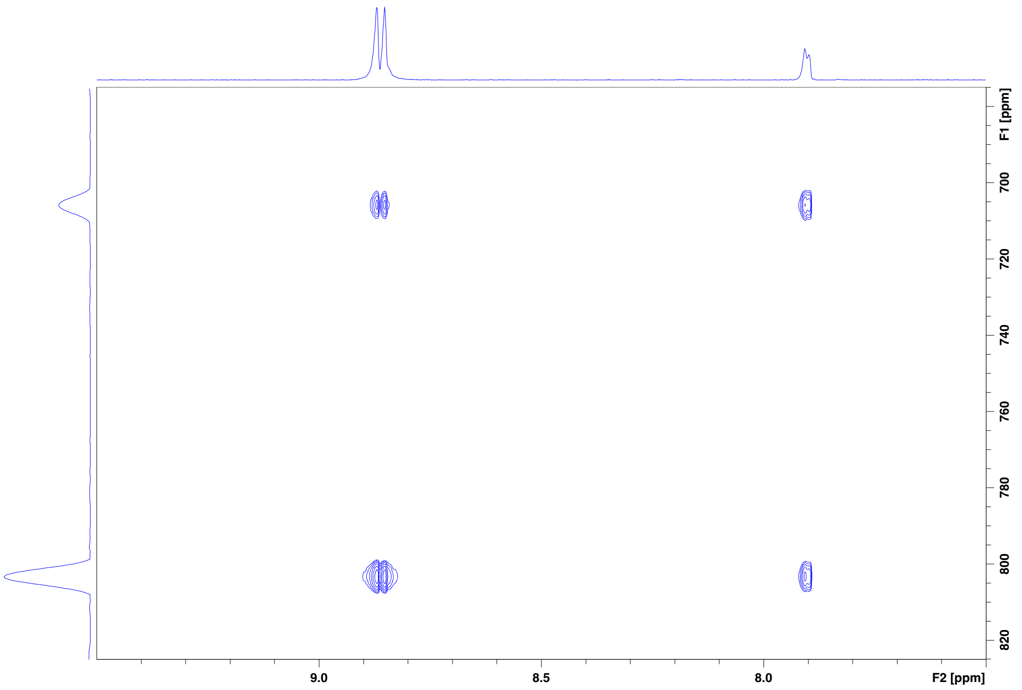

Figure S85. ^{1}H-^{109}Ag HSQC-EX NMR spectrum of **1-I** with mixing delay τ = 210 ms (500 MHz, 16 mM in CD_3CN, 243 K). Auto-peak integrals I_{AA} = 149798659, I_{BB} = 37051378. Exchange peak integrals I_{AB} = 43519706, I_{BA} = 35507007.

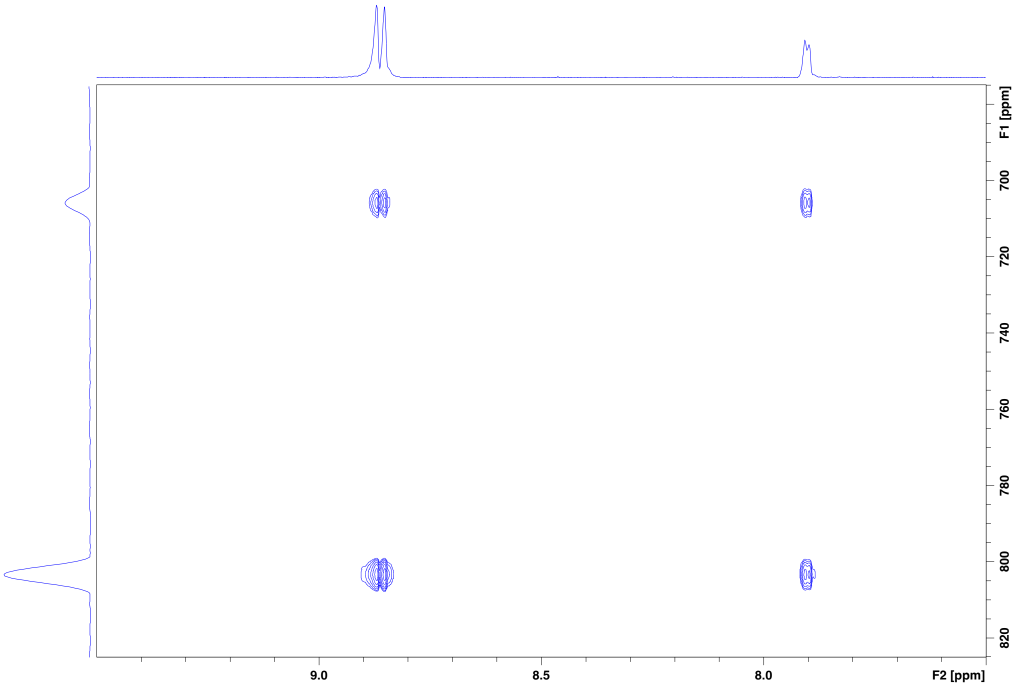

Figure S86. ^{1}H-^{109}Ag HSQC-EX NMR spectrum of **1-I** with mixing delay τ = 310 ms (500 MHz, 16 mM in CD_3CN, 243 K). Auto-peak integrals I_{AA} = 114159910, I_{BB} = 23325621. Exchange peak integrals I_{AB} = 40594279, I_{BA} = 31444839.

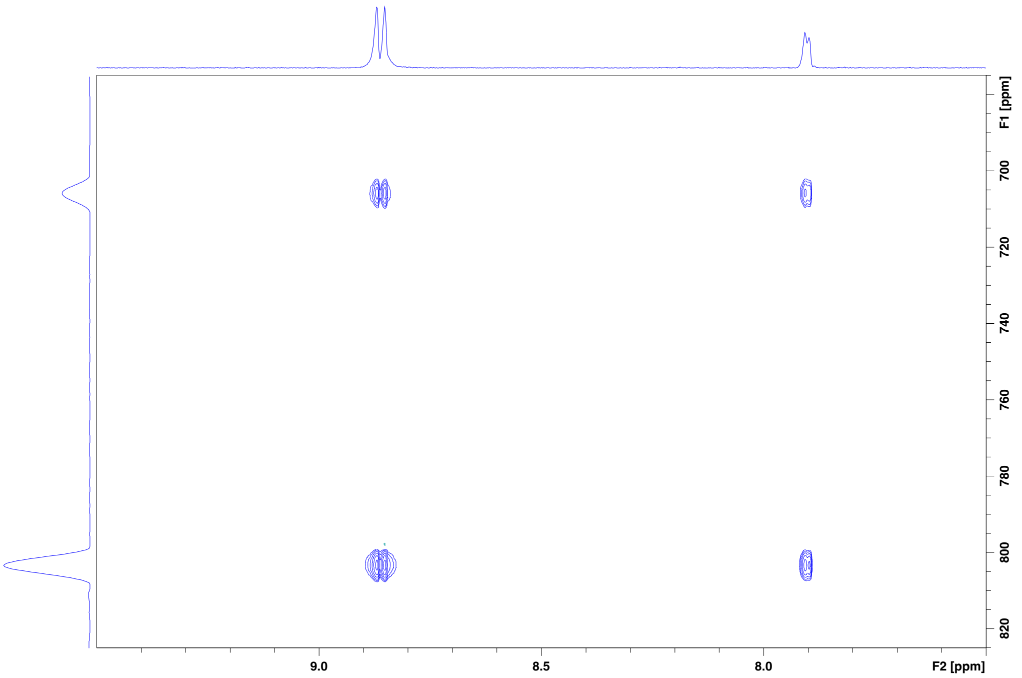

Figure S87. ^{1}H-^{109}Ag HSQC-EX NMR spectrum of **1-I** with mixing delay τ = 410 ms (500 MHz, 16 mM in CD_3CN, 243 K). Auto-peak integrals I_{AA} = 88202084, I_{BB} = 16528174. Exchange peak integrals I_{AB} = 35517649, I_{BA} = 27934837.

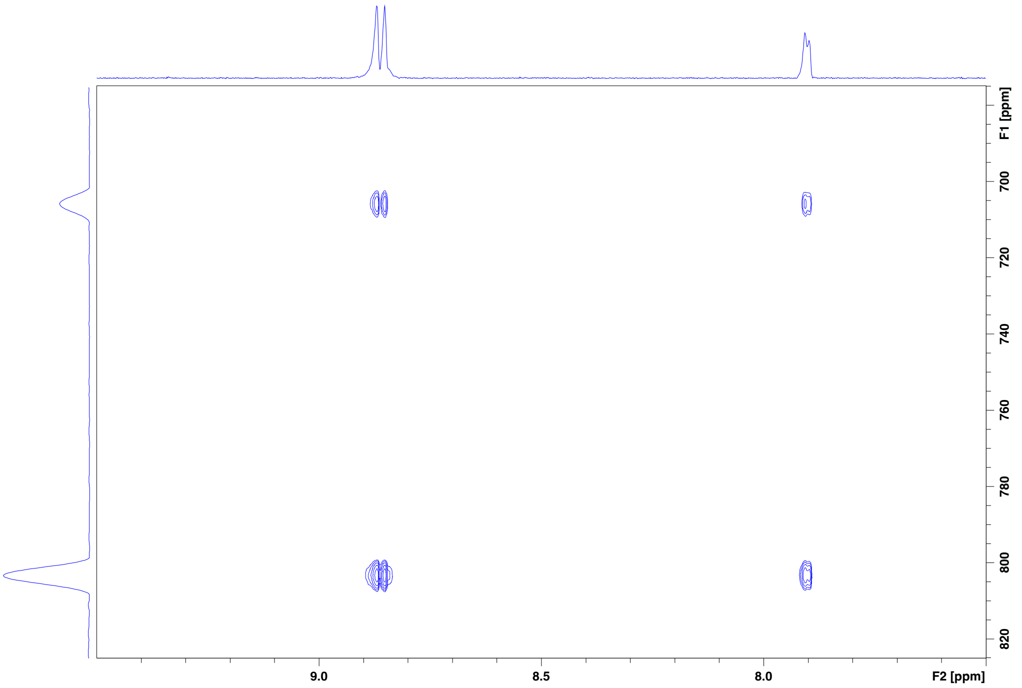

Figure S88. ^{1}H-^{109}Ag HSQC-EX NMR spectrum of **1-I** with mixing delay τ = 610 ms (500 MHz, 16 mM in CD_3CN, 243 K). Auto-peak integrals I_{AA} = 56801371, I_{BB} = 9208317. Exchange peak integrals I_{AB} = 24843677, I_{BA} = 18601666.

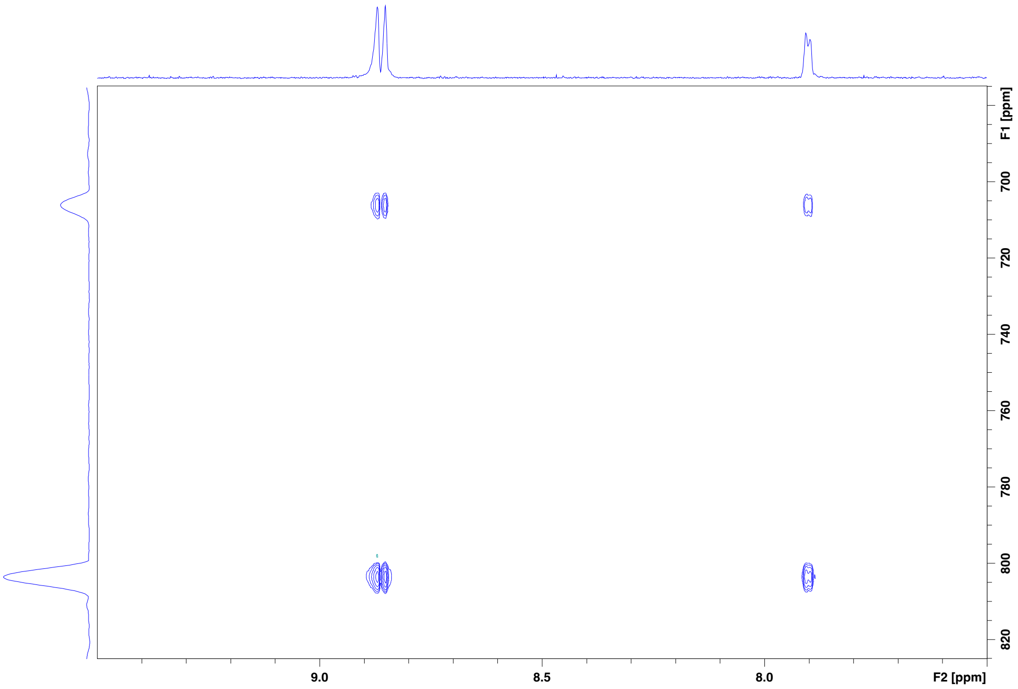

Figure S89. ^{1}H-^{109}Ag HSQC-EX NMR spectrum of **1-I** with mixing delay τ = 810 ms (500 MHz, 16 mM in CD_3CN, 243 K). Auto-peak integrals I_{AA} = 35362104, I_{BB} = 4535846. Exchange peak integrals I_{AB} = 14626741, I_{BA} = 12281400.

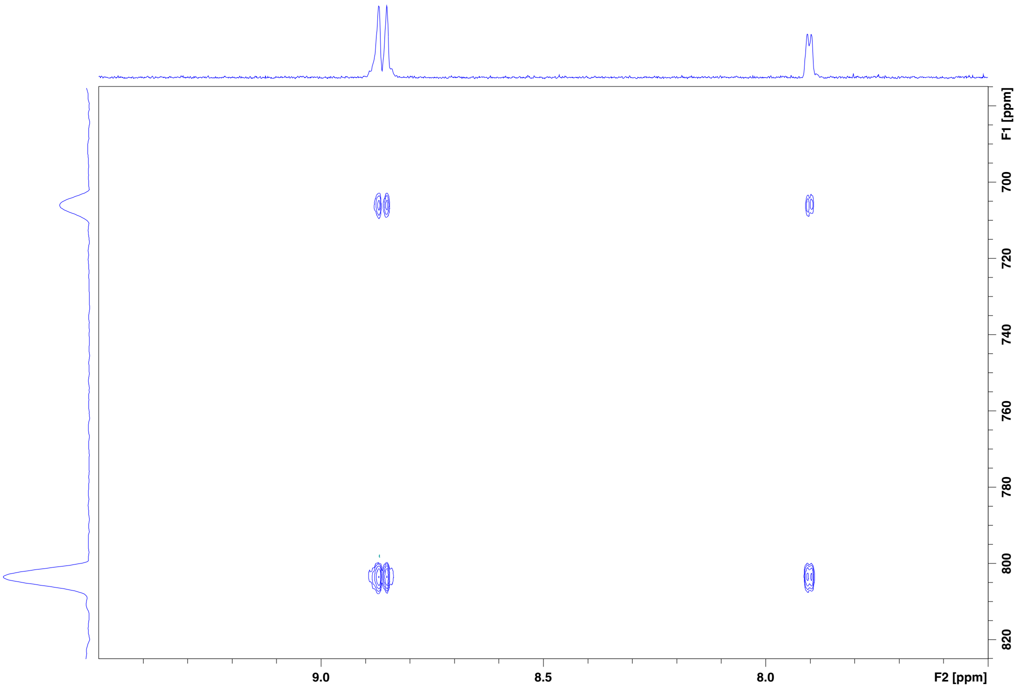

Figure S90. ^{1}H-^{109}Ag HSQC-EX NMR spectrum of **1-I** with mixing delay τ = 1010 ms (500 MHz, 16 mM in CD_3CN, 243 K). Auto-peak integrals I_{AA} = 23843585, I_{BB} = 3359239. Exchange peak integrals I_{AB} = 10422748, I_{BA} = 7242885.

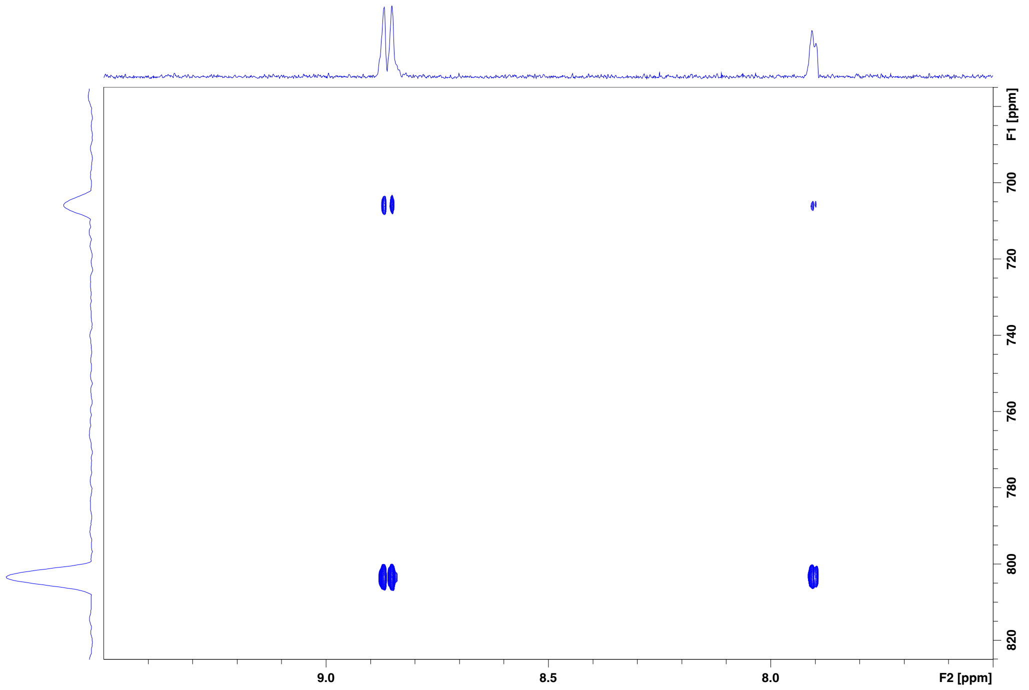

Figure S91. ^{1}H-^{109}Ag HSQC-EX NMR spectrum of **1-I** with mixing delay τ = 1210 ms (500 MHz, 16 mM in CD_3CN, 243 K). Auto-peak integrals I_{AA} = 14317304, I_{BB} = 2762599. Exchange peak integrals I_{AB} = 6299807, I_{BA} = 4419630.

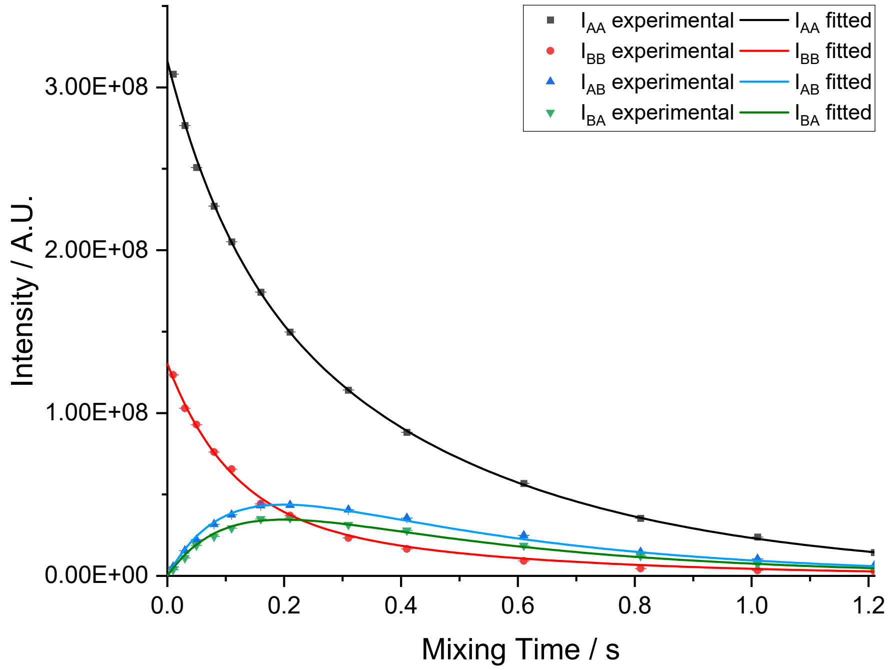

Figure S92. Decay of auto-peak and exchange peak volume of **1-I** at 243 K (16 mM in CD_3CN) with integral error bars.

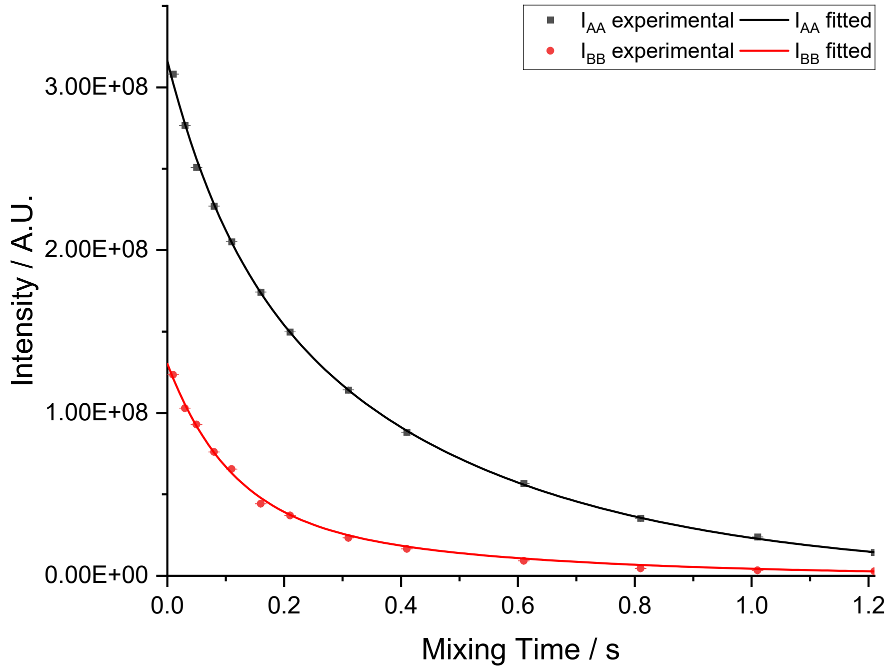

Figure S93. Decay of auto-peak volume of **1-I** at 243 K (16 mM in CD_3CN) with integral error bars.

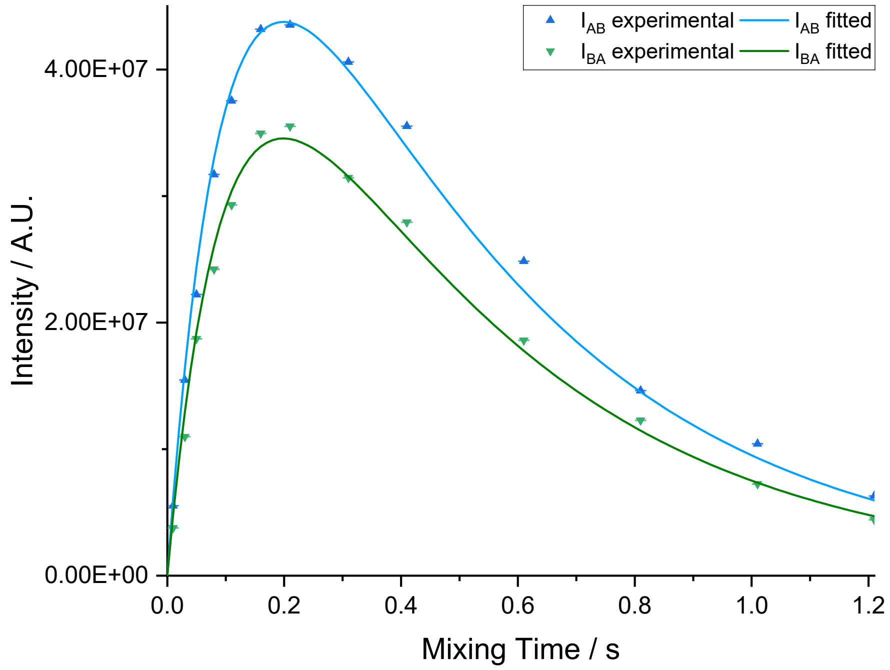

Figure S94. Decay of exchange peak volume of **1-I** at 243 K (16 mM in CD_3CN) with integral error bars.

4.7 Exchange Measurements on **1-Br** at 263 K

At 263 K, the silver exchange behavior in **1-Br** was observable. The data were fitted to the mathematical description (Section 4.5), with a magnetization transfer rate constant ($k_{\mathbf{1\text{-}Br}}^{263\ K}$ = 1.81(13) Hz). The ^{1}H-^{109}Ag HSQC-EX spectra below show a decrease in auto-peak volume and increase in exchange peak volume as mixing time increases, followed by a decrease in exchange volume at high mixing times due to longitudinal relaxation effects.

Table S3 shows the auto- and exchange peak volumes at each mixing times. Each row of the table corresponds to a ^{1}H-^{109}Ag HSQC-EX NMR spectrum (Figure S95-S114). These data are fitted (Figure S115-S117) to the equations detailed in section 4.5, optimized over the four signals, to extract the signal decay parameters and the magnetization transfer rate constant $k_{\mathbf{1\text{-}Br}}^{263\ K}$.

This experiment was performed at 15 mM concentration in CD_3CN at 263 K.

Table S3. Volume of auto-peak and exchange peaks in **1-Br** [15 mM] at 263 K.

Mixing Time (τ / s)	Major auto-peak (I_{AA})	Minor auto-peak (I_{BB})	Major to minor exchange peak (I_{AB})	Minor to Major exchange peak (I_{AB})
0.010	64695340 (± 35943)	36398732 (± 37134)	57776 (± 33352)	871133 (± 38288)
0.060	51850966 (± 33361)	28386467 (± 34467)	2825581 (± 30957)	2367070 (± 35538)
0.110	47248754 (± 35114)	24465332 (± 36277)	5229135 (± 32583)	3981533 (± 37405)
0.160	43202490 (± 34551)	21120818 (± 35696)	6004935 (± 32061)	4670156 (± 36805)
0.210	40162181 (± 33940)	17006964 (± 33725)	7662556 (± 31527)	5509945 (± 37559)
0.260	38999031 (± 33391)	14792118 (± 34497)	7918413 (± 30984)	6142783 (± 35569)
0.310	35160340 (± 34689)	12729802 (± 35838)	9042611 (± 32189)	6822785 (± 36952)
0.360	32361210 (± 33913)	11110703 (± 35037)	9103167 (± 31469)	6964641 (± 36126)
0.410	30463613 (± 30342)	9557496 (± 31348)	9201655 (± 28156)	6556065 (± 32322)
0.510	26747956 (± 35903)	7518680 (± 37092)	9376293 (± 33315)	6779679 (± 38245)
0.610	23820007 (± 33413)	6382232 (± 34520)	9057719 (± 31005)	6313655 (± 35593)
0.710	20938518 (± 34571)	4677216 (± 35716)	8552060 (± 32079)	5692891 (± 36826)
0.810	18329351 (± 35319)	3835884 (± 36489)	7781613 (± 32774)	5250073 (± 37623)
1.010	14621180 (± 35329)	2910597 (± 36500)	6403959 (± 32783)	5066589 (± 37634)
1.260	10951081 (± 30331)	1749743 (± 31337)	4955706 (± 28145)	3030786 (± 32310)
1.510	7862947 (± 35418)	1202068 (± 36592)	3918144 (± 32866)	2610916 (± 37729)
1.760	6141619 (± 33225)	1517653 (± 34326)	2950444 (± 30831)	1895150 (± 35393)
2.010	4445861 (± 33985)	1110079 (± 35111)	1717791 (± 31535)	1259602 (± 36202)
2.510	2701746 (± 34623)	431038 (± 35770)	1083793 (± 32127)	732162 (± 36881)
3.010	911790 (± 35037)	98089 (± 36198)	590887 (± 32512)	293104 (± 37323)

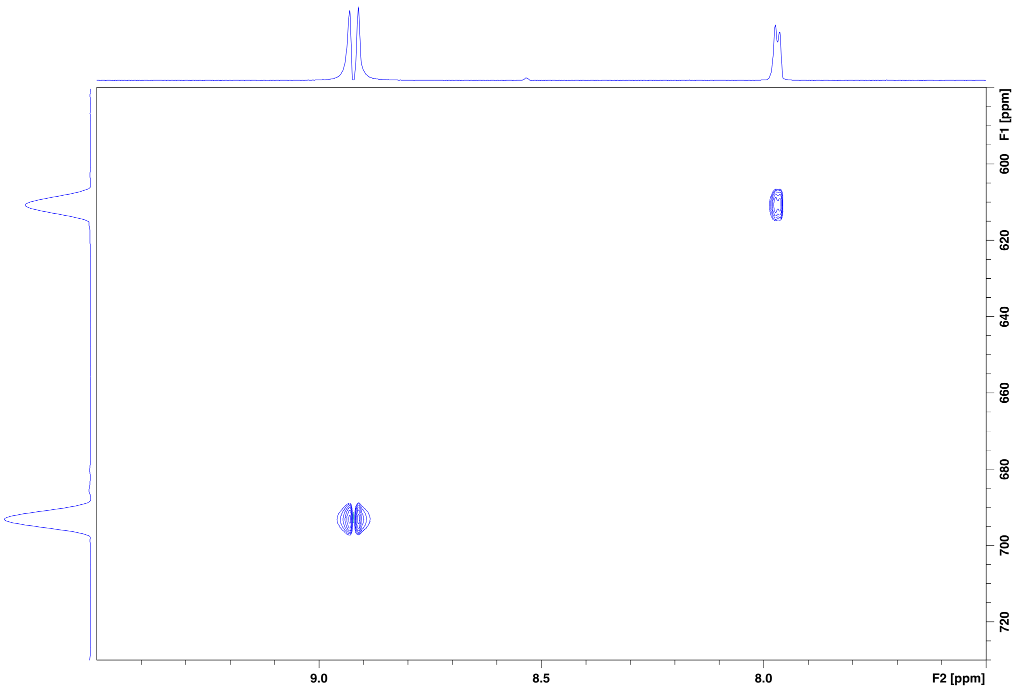

Figure S95. ^{1}H-^{109}Ag HSQC-EX NMR spectrum of **1-Br** with mixing delay τ= 10 ms (500 MHz, 15 mM in CD_3CN, 263 K). Auto-peak integrals I_{AA} = 64695340, I_{BB} = 36398732. Exchange peak integrals I_{AB} = 57776, I_{BA} = 871133.

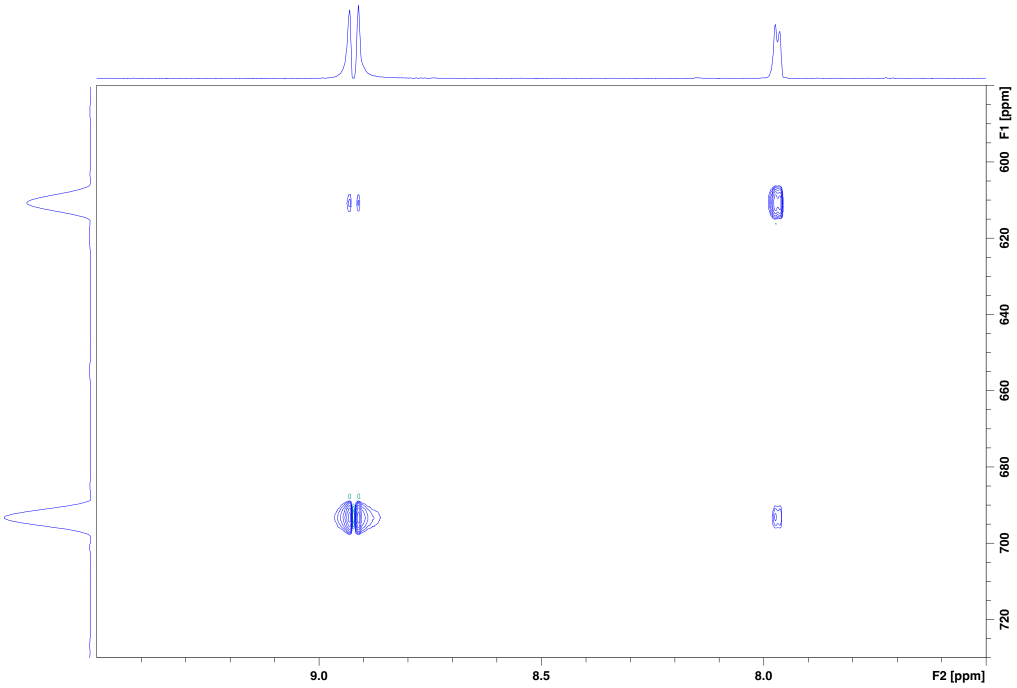

Figure S96. ^{1}H-^{109}Ag HSQC-EX NMR spectrum of **1-Br** with mixing delay τ = 60 ms (500 MHz, 15 mM in CD_3CN, 263 K). Auto-peak integrals I_{AA} = 51850966, I_{BB} = 28386467. Exchange peak integrals I_{AB} = 2825581, I_{BA} = 2367070.

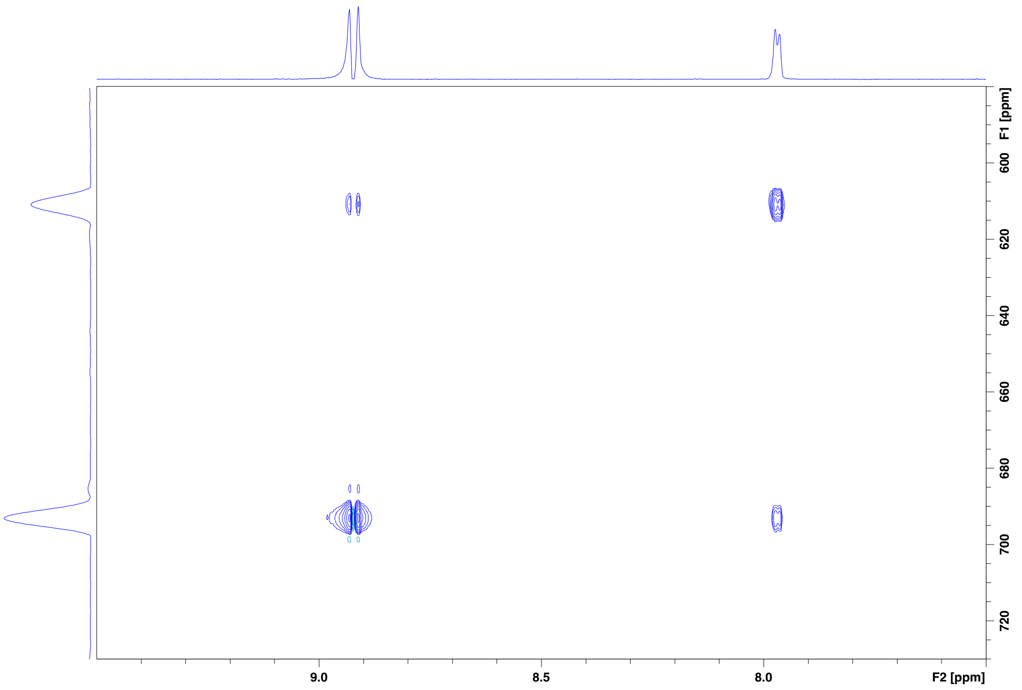

Figure S97. ^{1}H-^{109}Ag HSQC-EX NMR spectrum of **1-Br** with mixing delay τ = 110 ms (500 MHz, 15 mM in CD_3CN, 263 K). Auto-peak integrals I_{AA} = 47248754, I_{BB} = 24465332. Exchange peak integrals I_{AB} = 5229135, I_{BA} = 3981533.

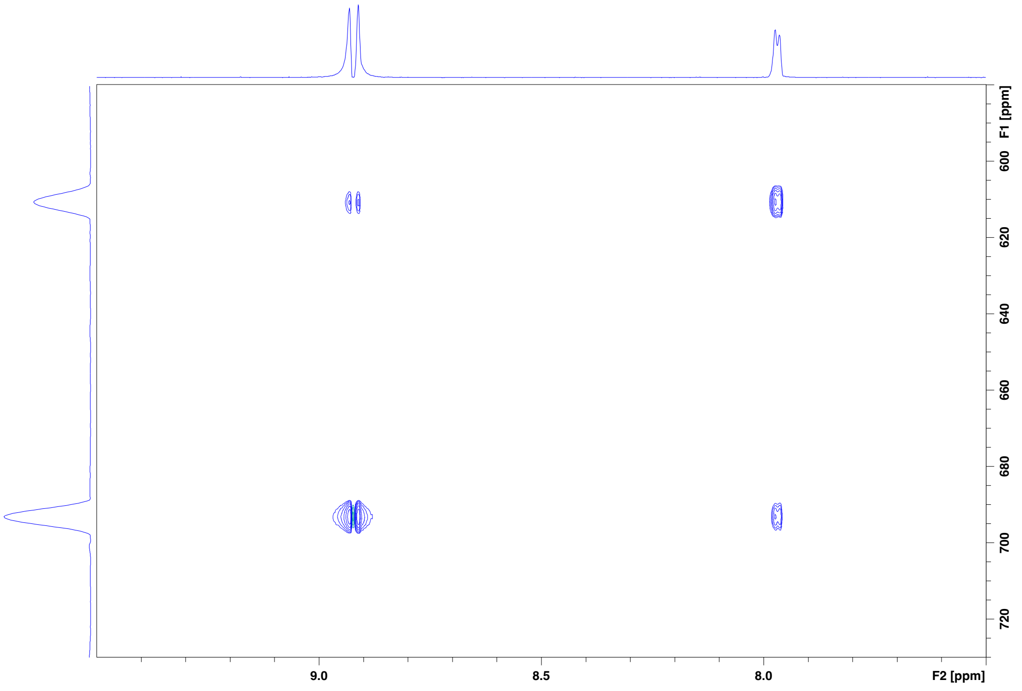

Figure S98. ^{1}H-^{109}Ag HSQC-EX NMR spectrum of **1-Br** with mixing delay τ = 160 ms (500 MHz, 15 mM in CD_3CN, 263 K). Auto-peak integrals I_{AA} = 43202490, I_{BB} = 21120818. Exchange peak integrals I_{AB} = 6004935, I_{BA} = 4670156.

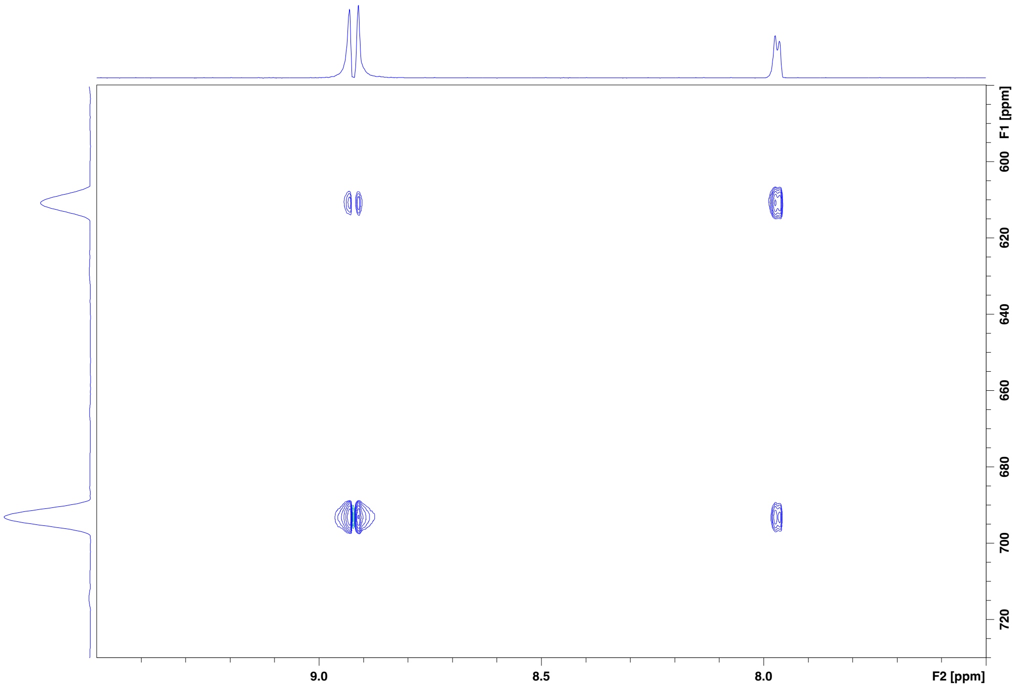

Figure S99. ^{1}H-^{109}Ag HSQC-EX NMR spectrum of **1-Br** with mixing delay τ = 210 ms (500 MHz, 15 mM in CD_3CN, 263 K). Auto-peak integrals I_{AA} = 40162181, I_{BB} = 17006964. Exchange peak integrals I_{AB} = 7662556, I_{BA} = 5509945.

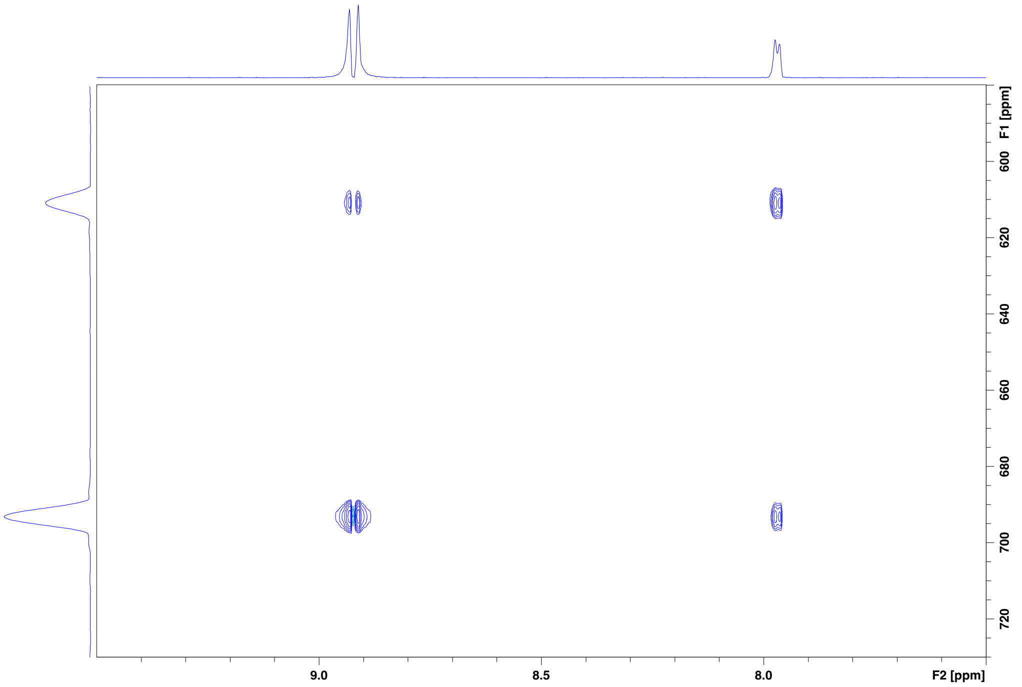

Figure S100. ^{1}H-^{109}Ag HSQC-EX NMR spectrum of **1-Br** with mixing delay τ = 260 ms (500 MHz, 15 mM in CD_3CN, 263 K). Auto-peak integrals I_{AA} = 38999031, I_{BB} = 14792118. Exchange peak integrals I_{AB} = 7918413, I_{BA} = 6142783.

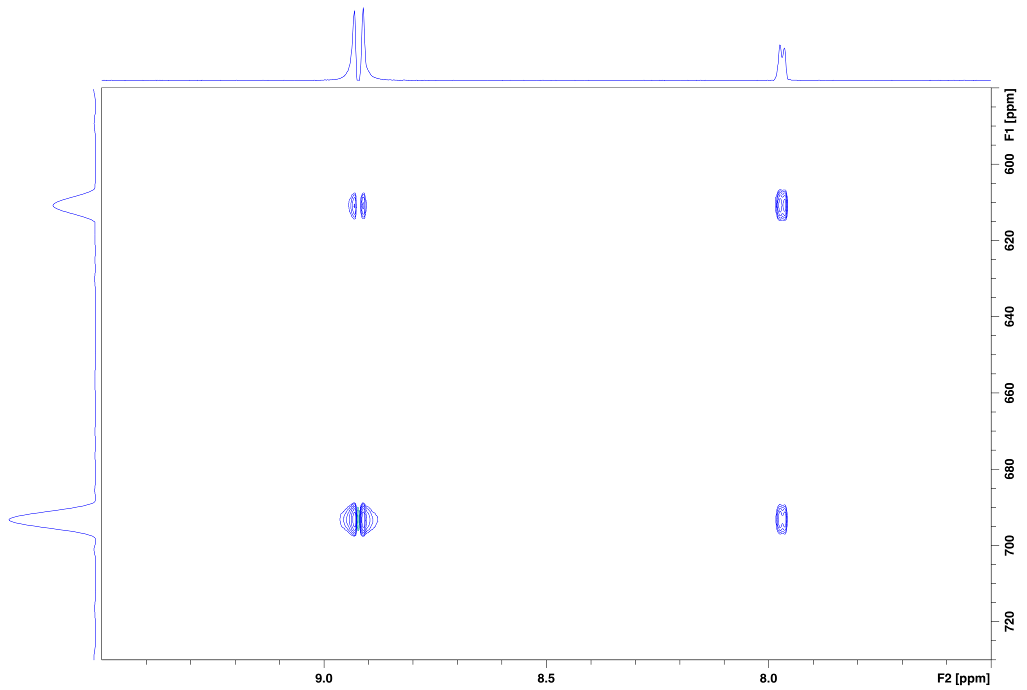

Figure S101. ^{1}H-^{109}Ag HSQC-EX NMR spectrum of **1-Br** with mixing delay τ = 310 ms (500 MHz, 15 mM in CD_3CN, 263 K). Auto-peak integrals I_{AA} = 35160340, I_{BB} = 12729802. Exchange peak integrals I_{AB} = 9042611, I_{BA} = 6822785.

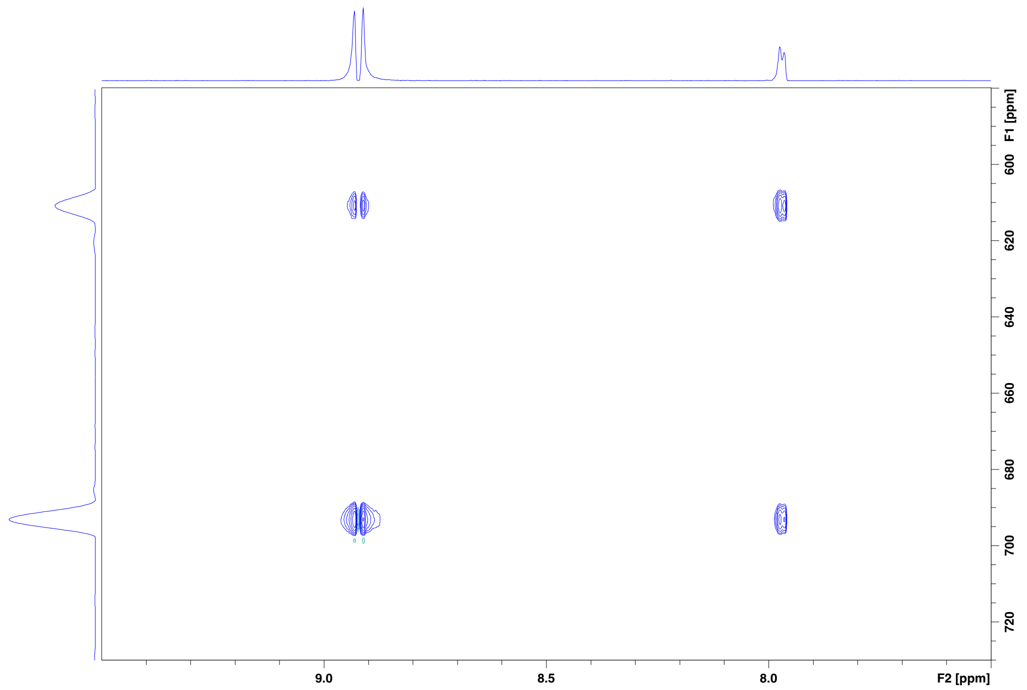

Figure S102. ^{1}H-^{109}Ag HSQC-EX NMR spectrum of **1-Br** with mixing delay τ = 360 ms (500 MHz, 15 mM in CD_3CN, 263 K). Auto-peak integrals I_{AA} = 32361210, I_{BB} = 11110703. Exchange peak integrals I_{AB} = 9103167, I_{BA} = 6964641.

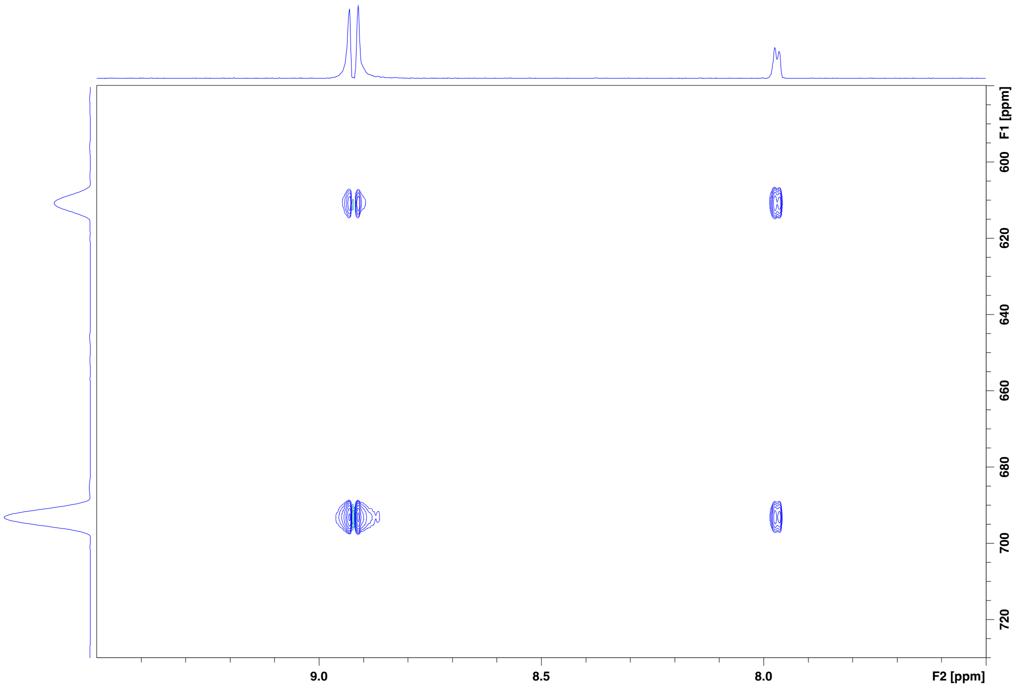

Figure S103. ^{1}H-^{109}Ag HSQC-EX NMR spectrum of **1-Br** with mixing delay τ = 410 ms (500 MHz, 15 mM in CD_3CN, 263 K). Auto-peak integrals I_{AA} = 30463613, I_{BB} = 9557496. Exchange peak integrals I_{AB} = 9201655, I_{BA} = 6556065.

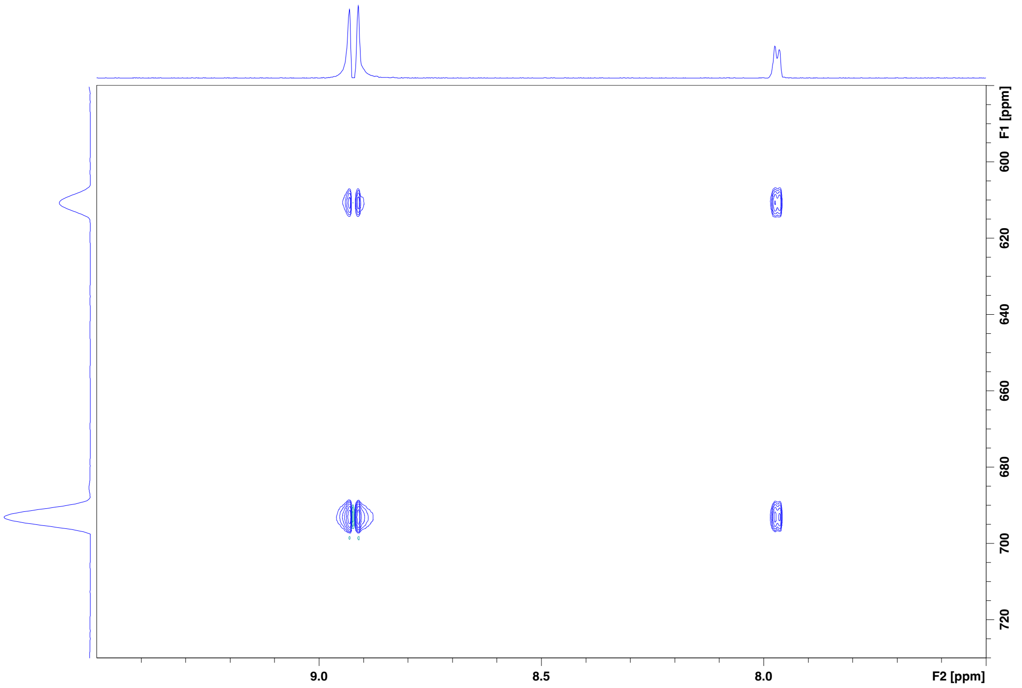

Figure S104. ^{1}H-^{109}Ag HSQC-EX NMR spectrum of **1-Br** with mixing delay τ = 510 ms (500 MHz, 15 mM in CD_3CN, 263 K). Auto-peak integrals I_{AA} = 26747956, I_{BB} = 7518680. Exchange peak integrals I_{AB} = 9376293, I_{BA} = 6779679.

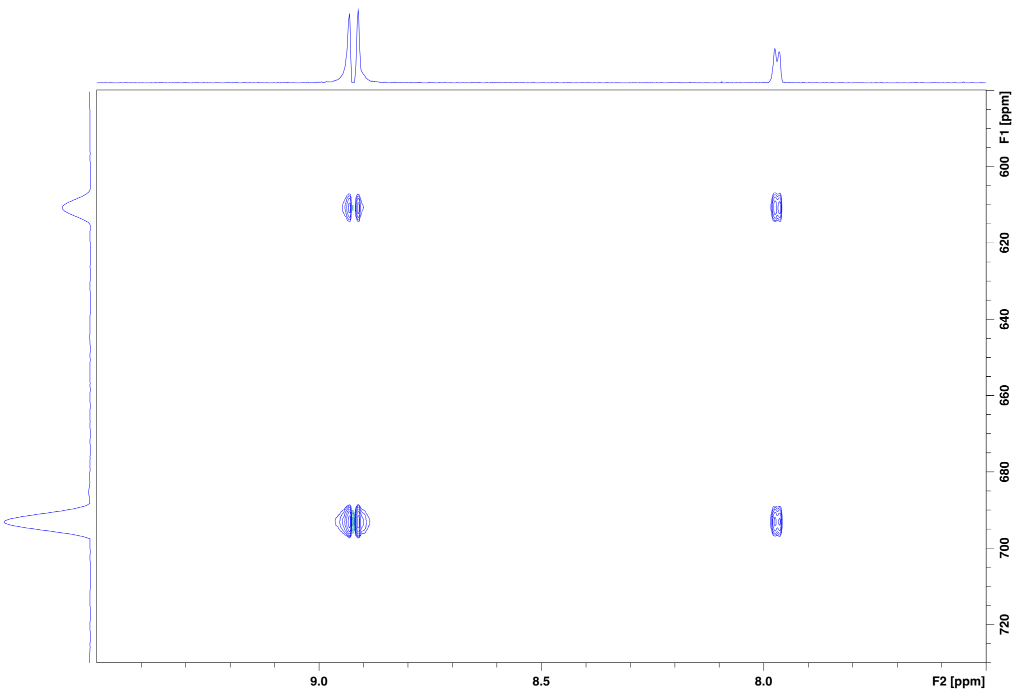

Figure S105. ^{1}H-^{109}Ag HSQC-EX NMR spectrum of **1-Br** with mixing delay τ = 610 ms (500 MHz, 15 mM in CD_3CN, 263 K). Auto-peak integrals I_{AA} = 23820007, I_{BB} = 6382232. Exchange peak integrals I_{AB} = 9057719, I_{BA} = 6313655.

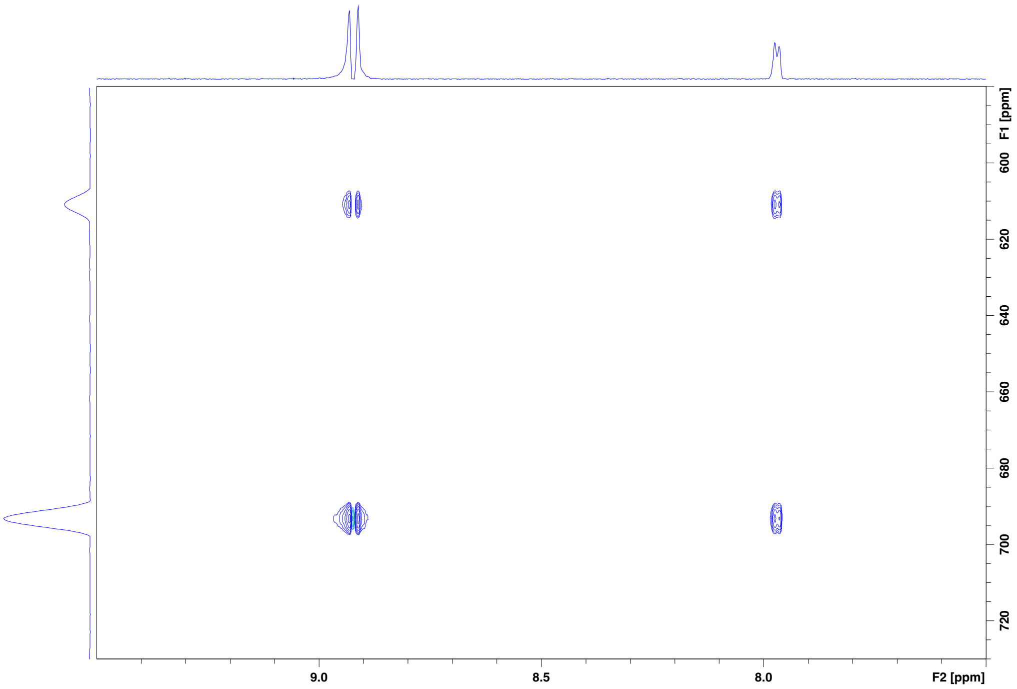

Figure S106. ^{1}H-^{109}Ag HSQC-EX NMR spectrum of **1-Br** with mixing delay τ = 710 ms (500 MHz, 15 mM in CD_3CN, 263 K). Auto-peak integrals I_{AA} = 20938518, I_{BB} = 4677216. Exchange peak integrals I_{AB} = 8552060, I_{BA} = 5692891.

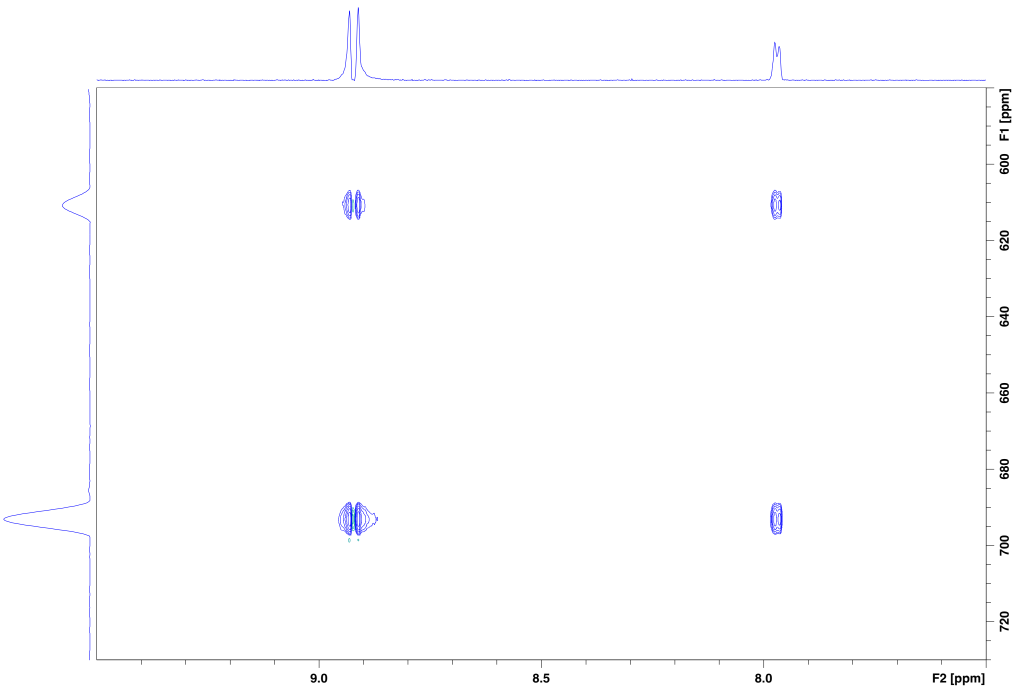

Figure S107. ^{1}H-^{109}Ag HSQC-EX NMR spectrum of **1-Br** with mixing delay τ = 810 ms (500 MHz, 15 mM in CD_3CN, 263 K). Auto-peak integrals I_{AA} = 18329351, I_{BB} = 3835884. Exchange peak integrals I_{AB} = 7781613, I_{BA} = 5250073.

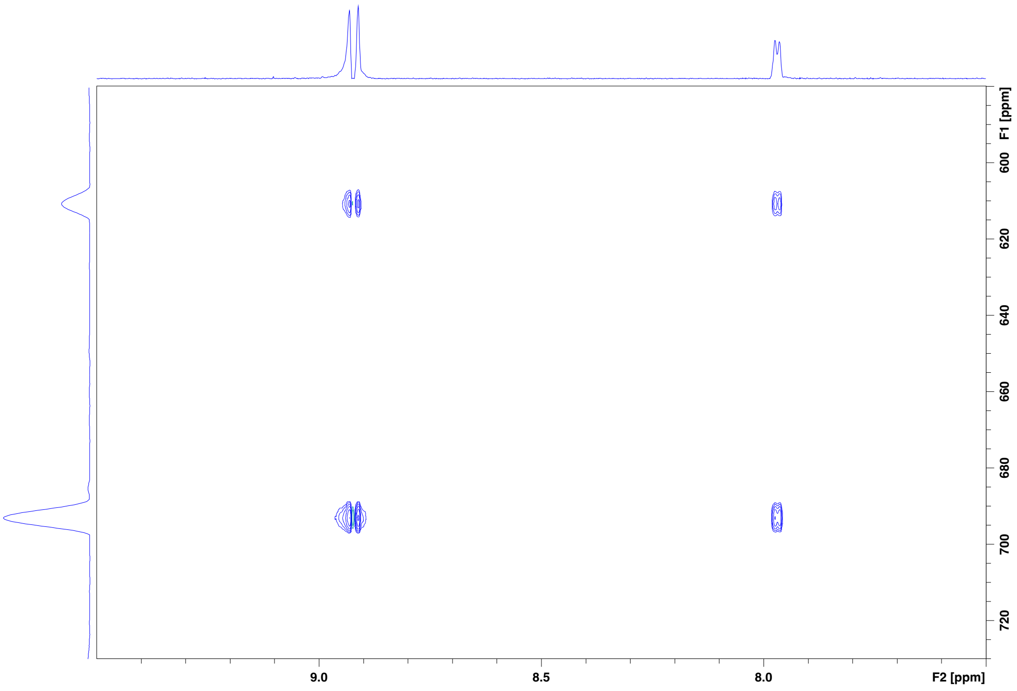

Figure S108. ^{1}H-^{109}Ag HSQC-EX NMR spectrum of **1-Br** with mixing delay τ = 1010 ms (500 MHz, 15 mM in CD_3CN, 263 K). Auto-peak integrals I_{AA} = 14621180, I_{BB} = 2910597. Exchange peak integrals I_{AB} = 6403959, I_{BA} = 5066589.

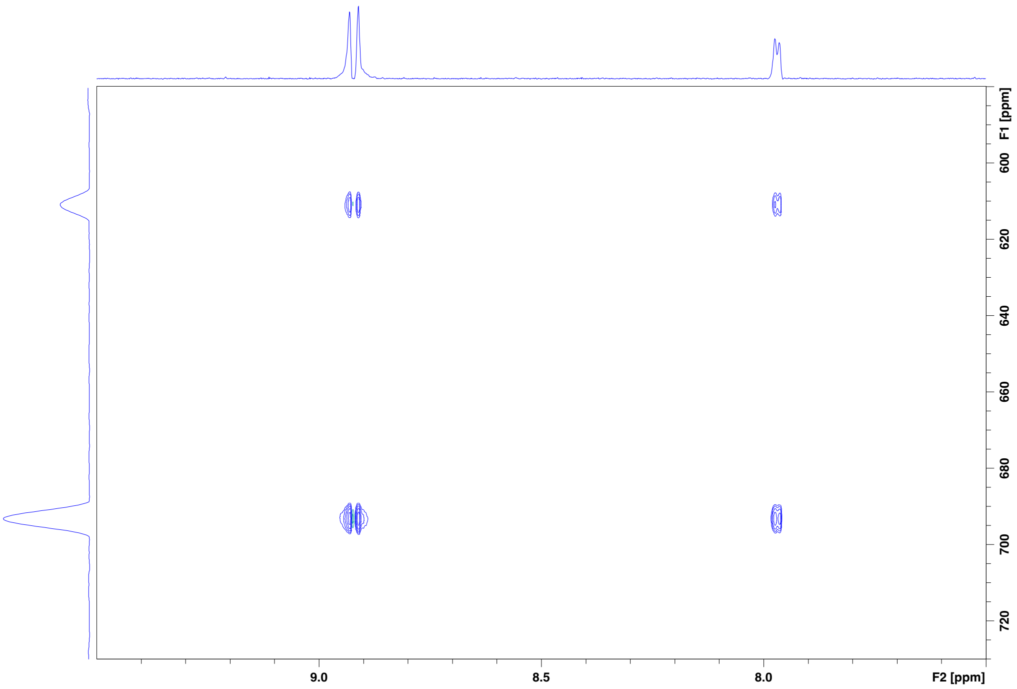

Figure S109. ^{1}H-^{109}Ag HSQC-EX NMR spectrum of **1-Br** with mixing delay τ = 1260 ms (500 MHz, 15 mM in CD_3CN, 263 K). Auto-peak integrals I_{AA} = 10951081, I_{BB} = 1749743. Exchange peak integrals I_{AB} = 4955706, I_{BA} = 3030786.

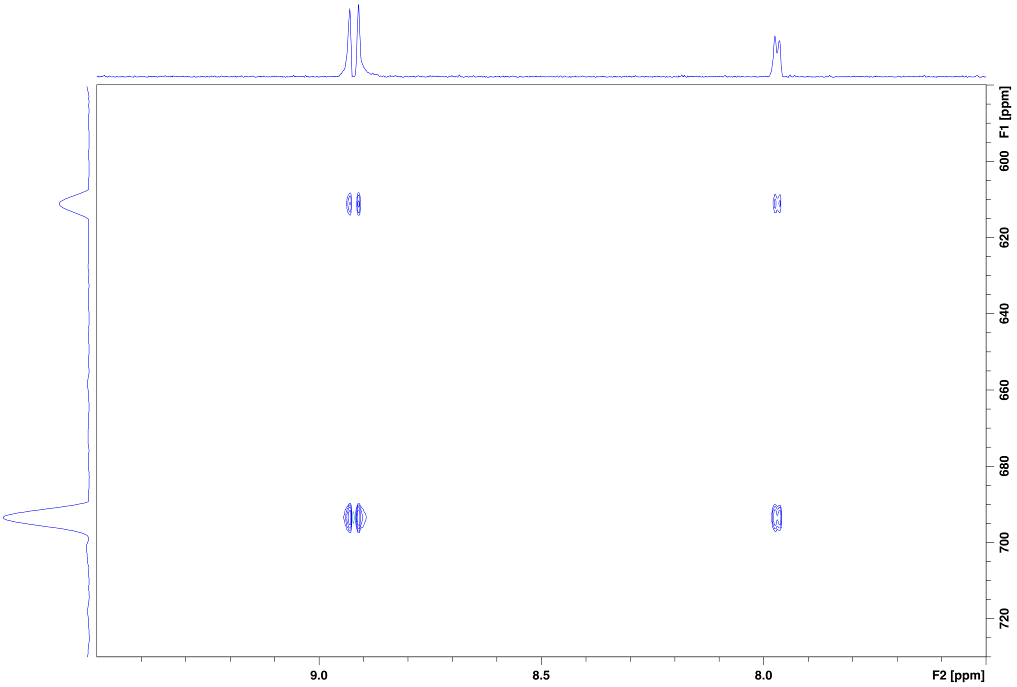

Figure S110. ^{1}H-^{109}Ag HSQC-EX NMR spectrum of **1-Br** with mixing delay τ = 1510 ms (500 MHz, 15 mM in CD_3CN, 263 K). Auto-peak integrals I_{AA} = 7862947, I_{BB} = 1202068. Exchange peak integrals I_{AB} = 3918144, I_{BA} = 2610916.

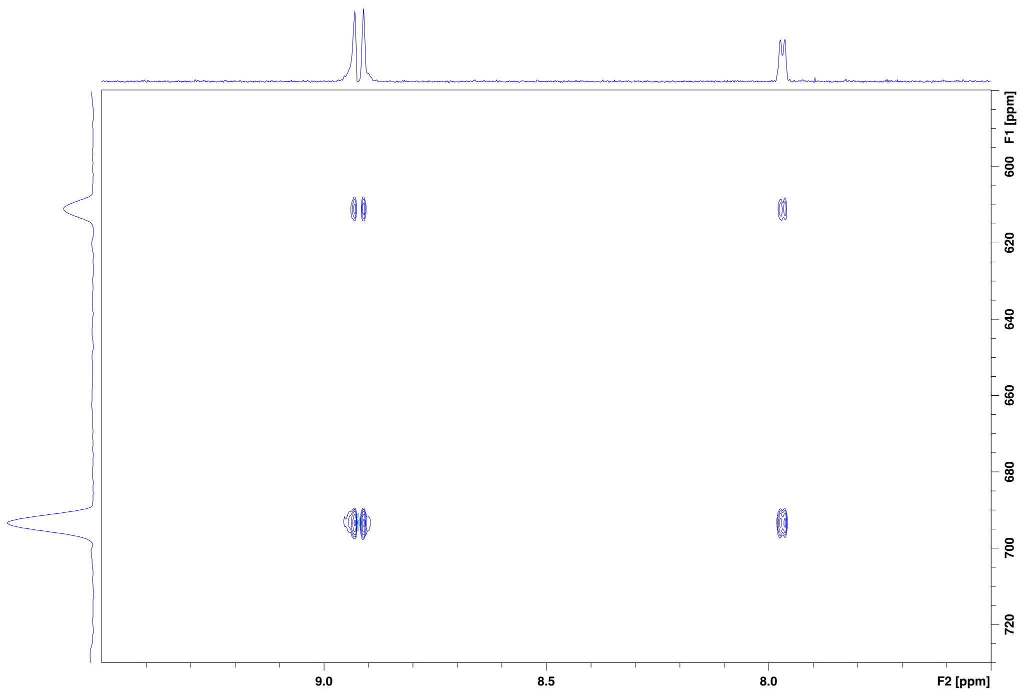

Figure S111. ^{1}H-^{109}Ag HSQC-EX NMR spectrum of **1-Br** with mixing delay τ = 1760 ms (500 MHz, 15 mM in CD_3CN, 263 K). Auto-peak integrals I_{AA} = 6141619, I_{BB} = 1517653. Exchange peak integrals I_{AB} = 2950444, I_{BA} = 1895150.

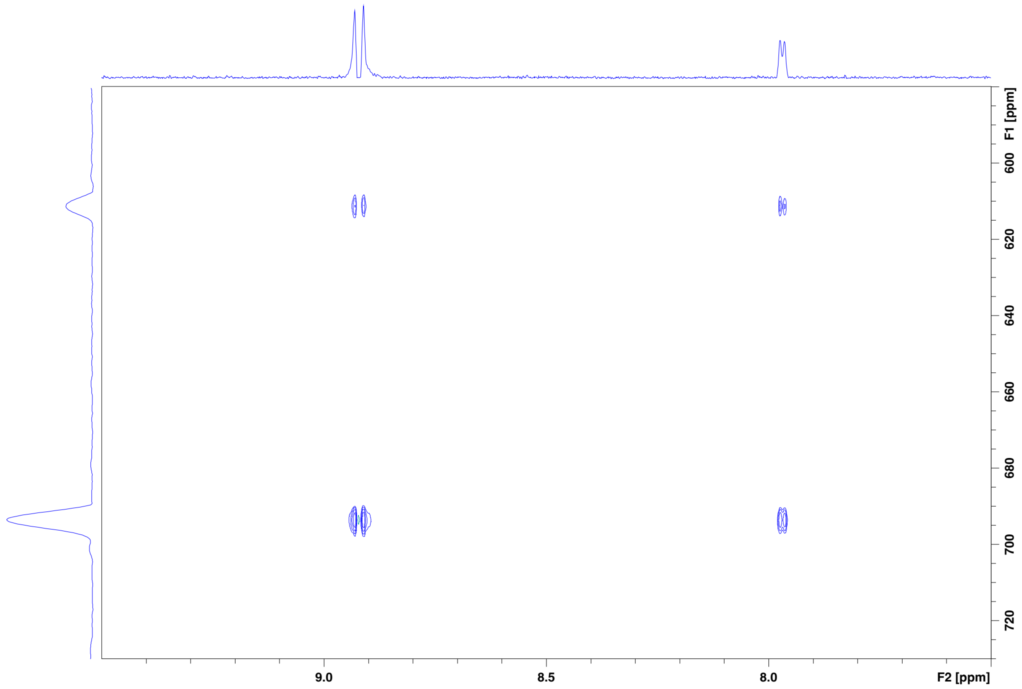

Figure S112. ^{1}H-^{109}Ag HSQC-EX NMR spectrum of **1-Br** with mixing delay τ = 2010 ms (500 MHz, 15 mM in CD_3CN, 263 K). Auto-peak integrals I_{AA} = 4445861, I_{BB} = 1110079. Exchange peak integrals I_{AB} = 1717791, I_{BA} = 1259602.

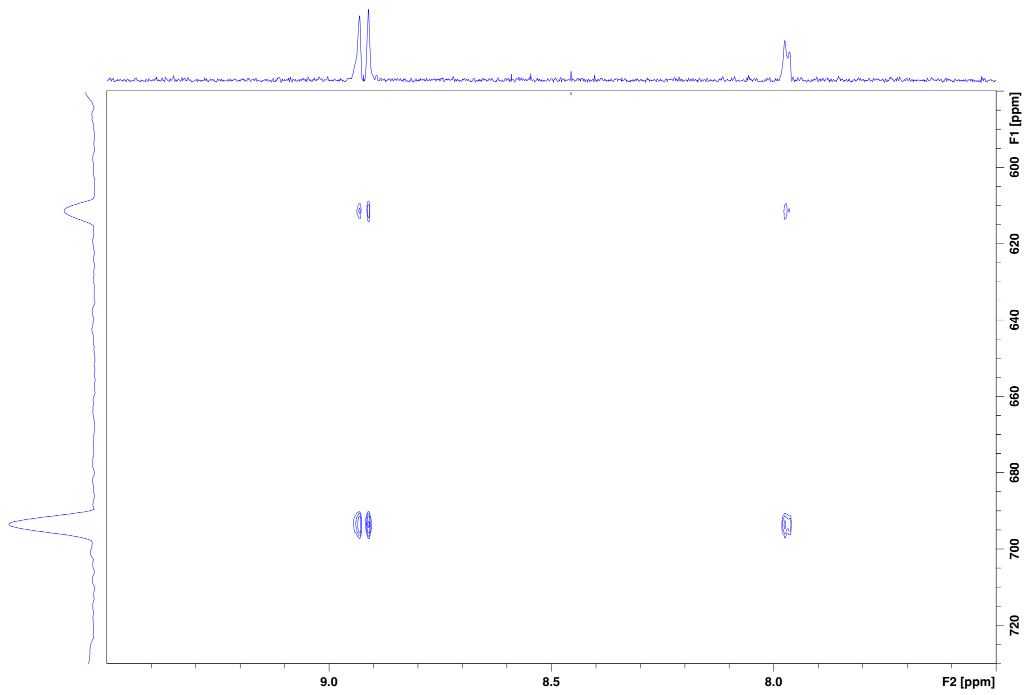

Figure S113. ^{1}H-^{109}Ag HSQC-EX NMR spectrum of **1-Br** with mixing delay τ = 2510 ms (500 MHz, 15 mM in CD_3CN, 263 K). Auto-peak integrals I_{AA} = 2701746, I_{BB} = 431038. Exchange peak integrals I_{AB} = 1083793, I_{BA} = 73216.

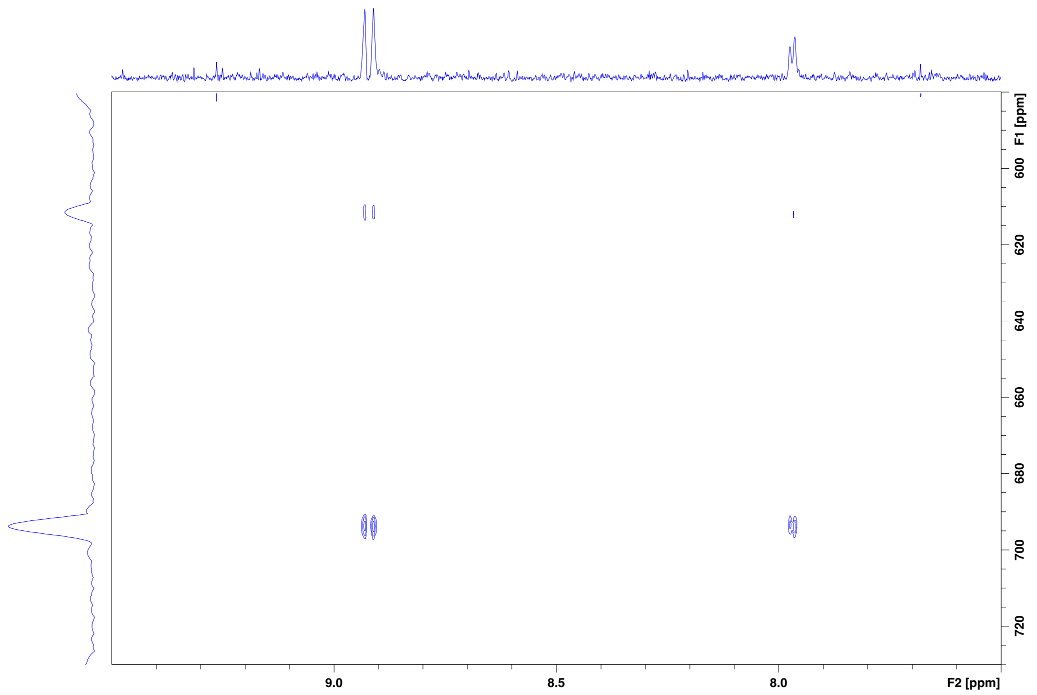

Figure S114. ^{1}H-^{109}Ag HSQC-EX NMR spectrum of **1-Br** with mixing delay τ = 3010 ms (500 MHz, 15 mM in CD_3CN, 263 K). Auto-peak integrals I_{AA} = 911790, I_{BB} = 98089. Exchange peak integrals I_{AB} = 590887, I_{BA} = 293104.

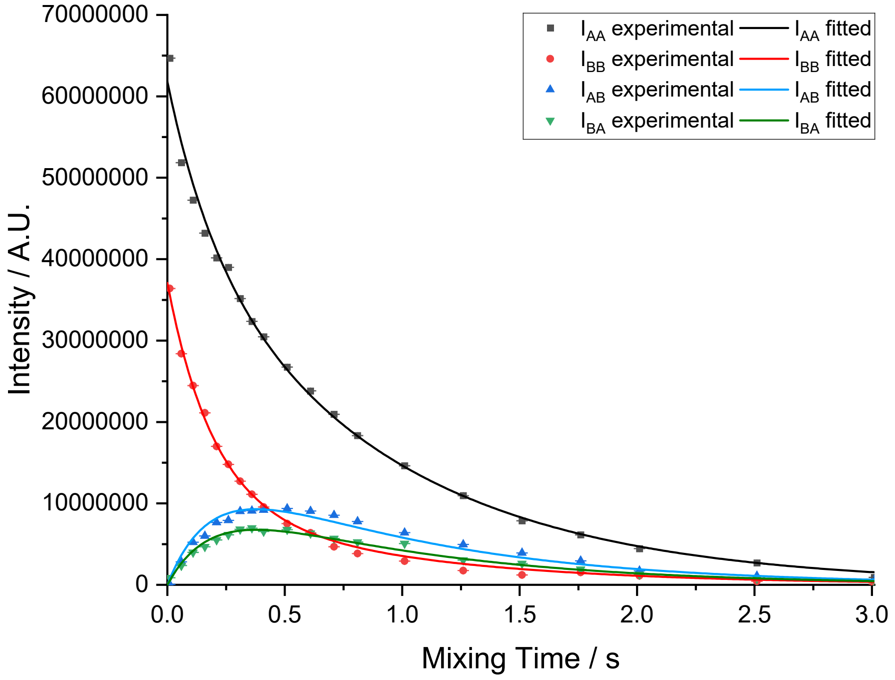

Figure S115. Decay of auto-peak and exchange peak volume of **1-Br** at 263 K (15 mM in CD_3CN) with integral error bars.

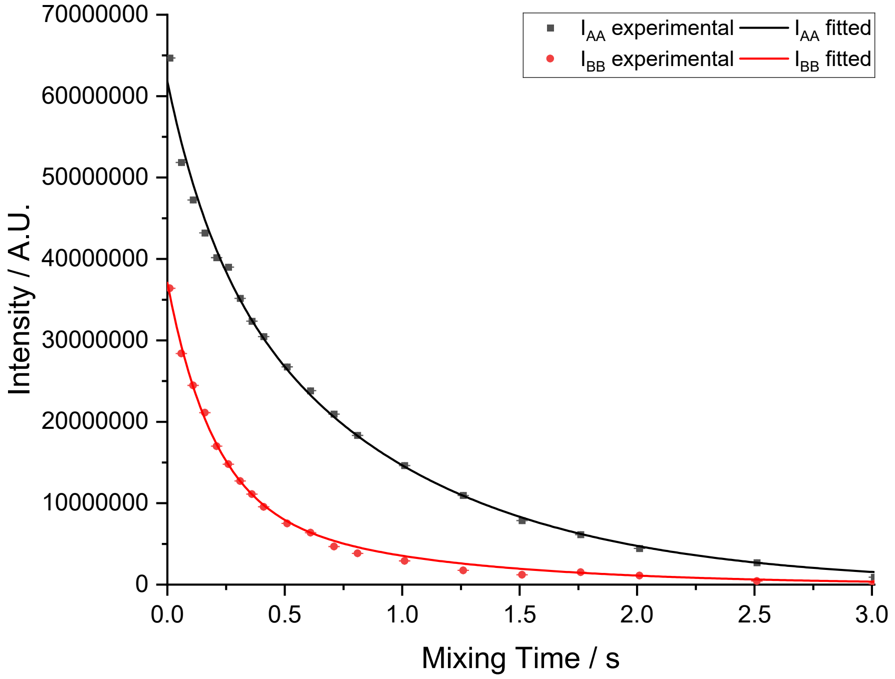

Figure S116. Decay of auto-peak volume of **1-Br** at 263 K (15 mM in CD_3CN) with integral error bars.

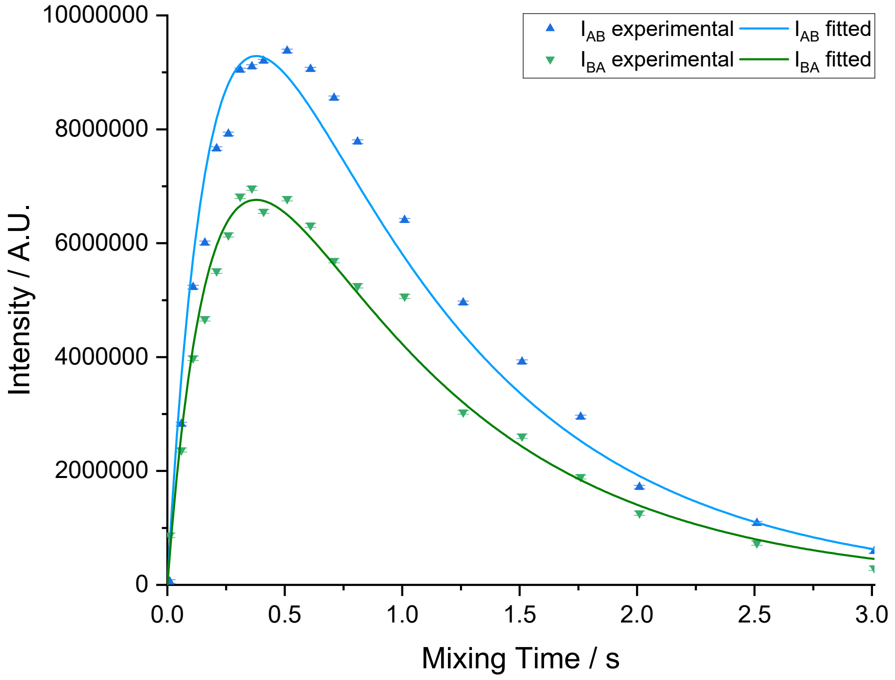

Figure S117. Decay of exchange peak volume of **1-Br** at 263 K (15 mM in CD_3CN) with integral error bars.

4.8 Exchange Measurements on **1-Cl** at 298 K

At 298 K, the silver exchange behavior in **1-Cl** was observable. The data were fitted to the mathematical description (Section 4.5), with a magnetization transfer rate constant ($k_{1\text{-Cl}}^{298\,K}$ = 2.33(22) Hz). The ^{1}H-^{109}Ag HSQC-EX spectra below show a decrease in auto-peak volume and increase in exchange peak volume as mixing time increases, followed by a decrease in exchange volume at high mixing times due to longitudinal relaxation effects.

Table S4 shows the auto- and exchange peak volumes at each mixing times. Each row of the table corresponds to a ^{1}H-^{109}Ag HSQC-EX NMR spectrum (Figure S118-S136). These data are fitted (Figure S137-S139) to the equations detailed in section 4.5, optimized over the four signals, to extract the signal decay parameters and the magnetization transfer rate constant $k_{1\text{-Cl}}^{298\,K}$.

This experiment was performed at 20 mM concentration in CD_3CN at 298 K.

Table S4. Volume of auto-peak and exchange peaks in **1-Cl** [20 mM] at 298 K.

**Mixing Time (τ / s)**	**Major auto-peak (I_{AA})**	**Minor auto-peak (I_{BB})**	**Major to minor exchange peak (I_{AB})**	**Minor to Major exchange peak (I_{AB})**
0.010	68510618 (± 16967)	53204326 (± 20330)	2014767 (± 21541)	1956196 (± 19042)
0.030	58018196 (± 15947)	49067519 (± 19108)	3810065 (± 20246)	4079906 (± 17897)
0.050	50743003 (± 17983)	44821041 (± 21547)	5296132 (± 22831)	5680880 (± 20182)
0.070	48050948 (± 17656)	40312914 (± 19620)	6716822 (± 22163)	7211595 (± 18955)
0.090	46177174 (± 17646)	36960214 (± 21143)	8110126 (± 22403)	8802041 (± 19804)
0.110	44871444 (± 18118)	34544847 (± 21710)	9633591 (± 23003)	9988781 (± 20334)
0.160	40416544 (± 15015)	28059930 (± 17991)	11701781 (± 19062)	12298368 (± 16851)
0.210	35547217 (± 17218)	23524435 (± 20631)	12723913 (± 21860)	13781779 (± 19324)
0.260	32102212 (± 16604)	19747709 (± 19895)	13375386 (± 21081)	14622693 (± 18635)
0.310	29400026 (± 17071)	16951450 (± 20454)	13576259 (± 21673)	14774803 (± 19158)
0.360	26641207 (± 18401)	14744625 (± 22048)	13579412 (± 23361)	14618267 (± 20651)
0.410	24324876 (± 16877)	12887322 (± 20223)	12875318 (± 21427)	14092401 (± 18941)
0.510	20916347 (± 17106)	10376768 (± 20496)	11896803 (± 21717)	13167610 (± 19197)
0.610	17154733 (± 17600)	8223700 (± 21089)	10340746 (± 22345)	11142539 (± 19753)
0.810	11716138 (± 16471)	5621795 (± 19735)	7421094 (± 20911)	8106201 (± 18485)
1.010	6813088 (± 17770)	2973538 (± 21292)	4281700 (± 22560)	4430850 (± 19943)
1.510	1044974 (± 17601)	340585 (± 21090)	158456 (± 22346)	704698 (± 19753)
2.010	-477976 (± 17774)	-311342 (± 21297)	-429869 (± 22565)	-397991 (± 19947)
2.510	72037 (± 18001)	-37681 (± 21569)	-19112 (± 22854)	-53597 (± 20203)

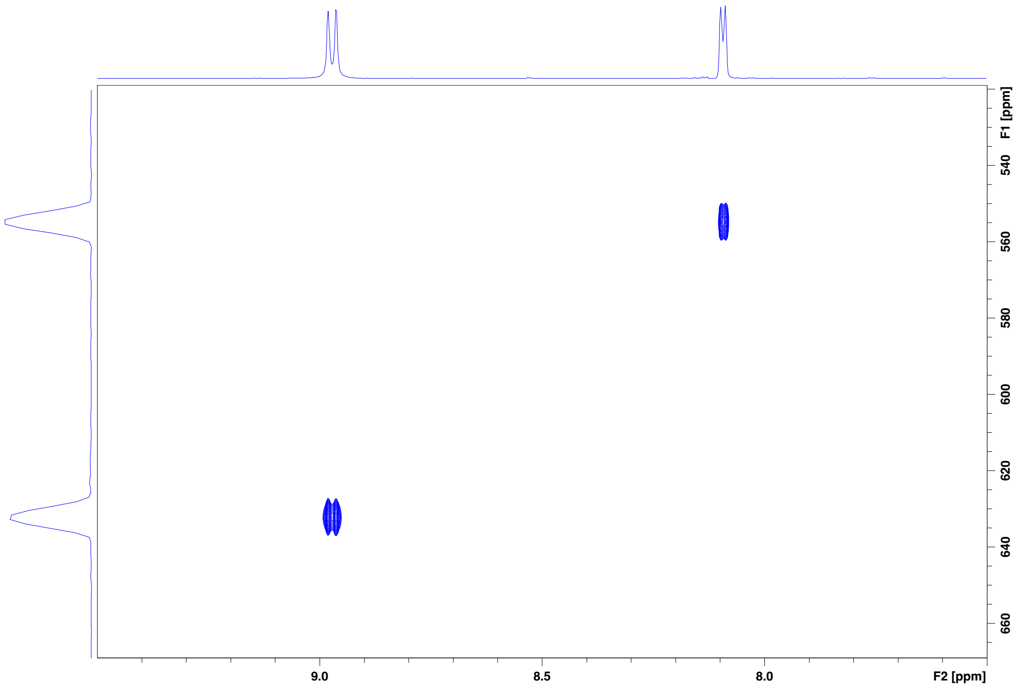

Figure S118. ^{1}H-^{109}Ag HSQC-EX NMR spectrum of **1-Cl** with mixing delay τ = 10 ms (500 MHz, 20 mM in CD_3CN, 298 K). Auto-peak integrals I_{AA} = 68510618, I_{BB} = 53204326. Exchange peak integrals I_{AB} = 2014767, I_{BA} = 1956196.

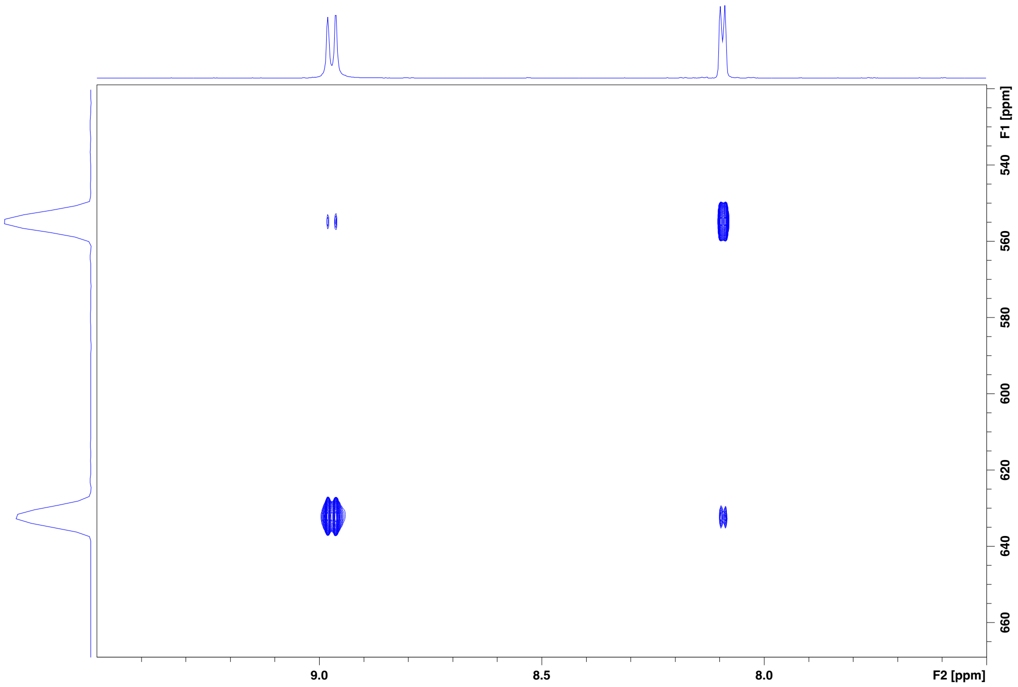

Figure S119. ^{1}H-^{109}Ag HSQC-EX NMR spectrum of **1-Cl** with mixing delay τ = 30 ms (500 MHz, 20 mM in CD_3CN, 298 K). Auto-peak integrals I_{AA} = 58018196, I_{BB} = 49067519. Exchange peak integrals I_{AB} = 3810065, I_{BA} = 4079906.

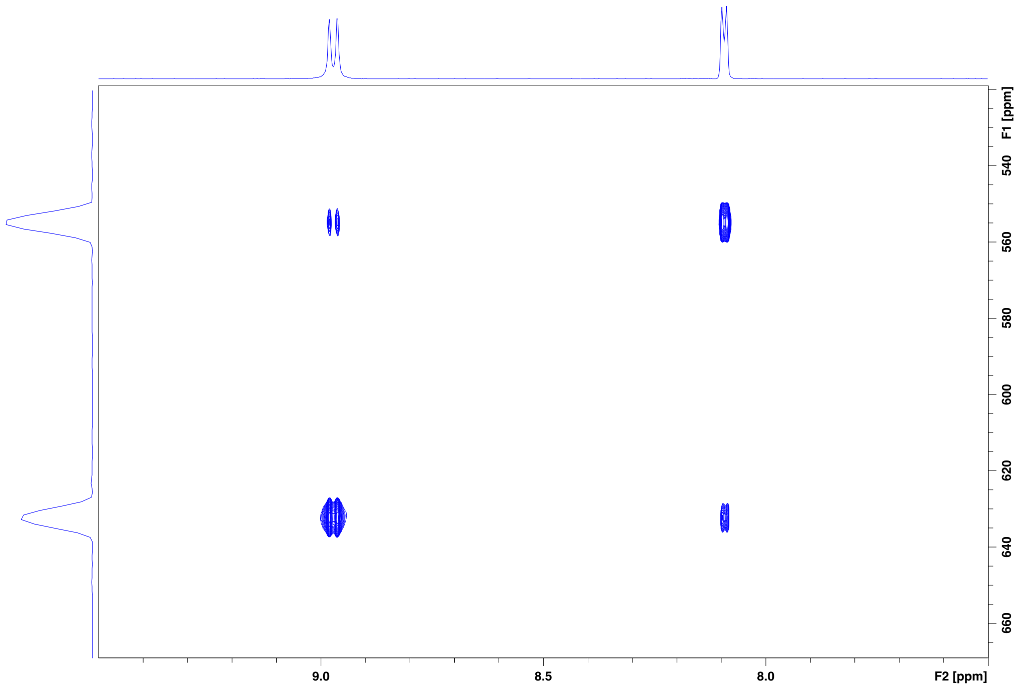

Figure S120. ^{1}H-^{109}Ag HSQC-EX NMR spectrum of **1-Cl** with mixing delay τ = 50 ms (500 MHz, 20 mM in CD_3CN, 298 K). Auto-peak integrals I_{AA} = 50743003, I_{BB} = 44821041. Exchange peak integrals I_{AB} = 5296132, I_{BA} = 5680880.

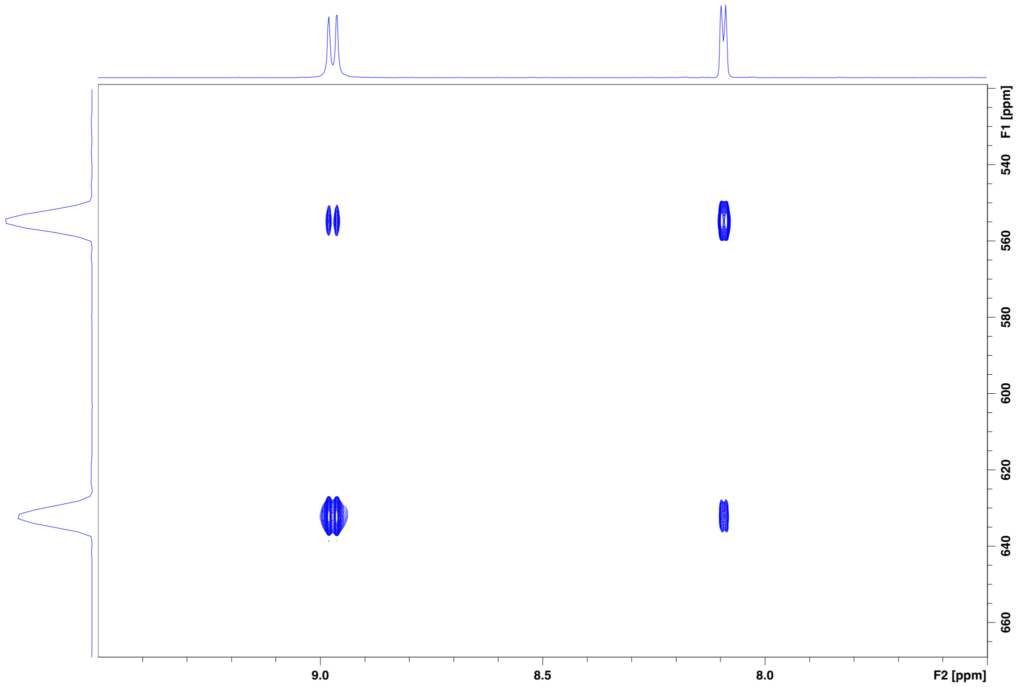

Figure S121. ^{1}H-^{109}Ag HSQC-EX NMR spectrum of **1-Cl** with mixing delay τ = 70 ms (500 MHz, 20 mM in CD_3CN, 298 K). Auto-peak integrals I_{AA} = 48050948, I_{BB} = 40312914. Exchange peak integrals I_{AB} = 6716822, I_{BA} = 7211595.

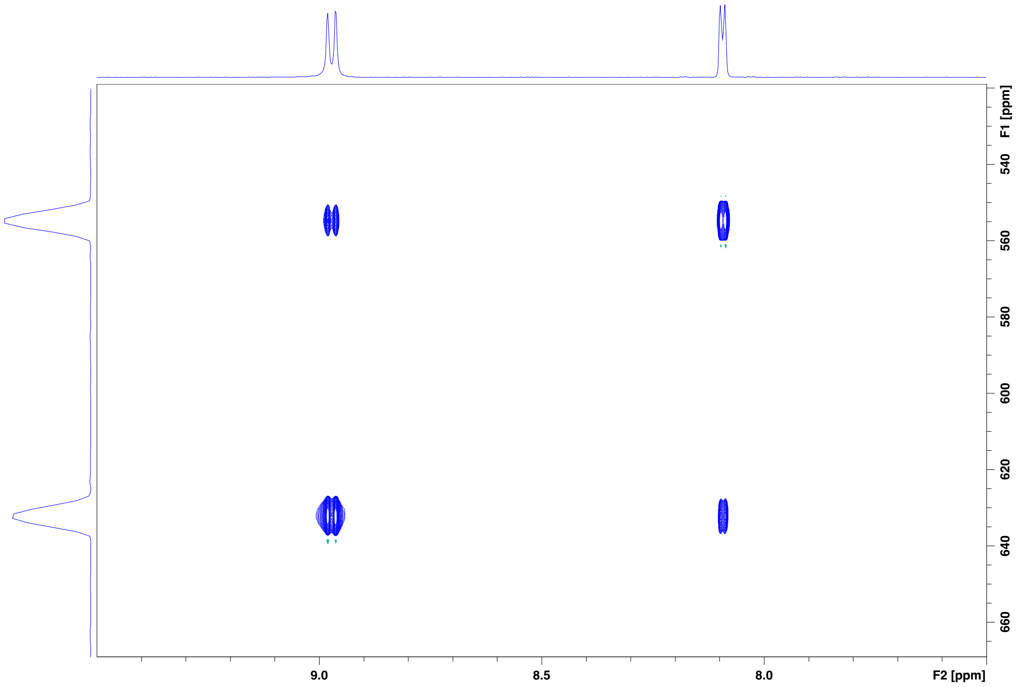

Figure S122. ^{1}H-^{109}Ag HSQC-EX NMR spectrum of **1-Cl** with mixing delay τ = 90 ms (500 MHz, 20 mM in CD_3CN, 298 K). Auto-peak integrals I_{AA} = 46177174, I_{BB} = 36960214. Exchange peak integrals I_{AB} = 8110126, I_{BA} = 8802041.

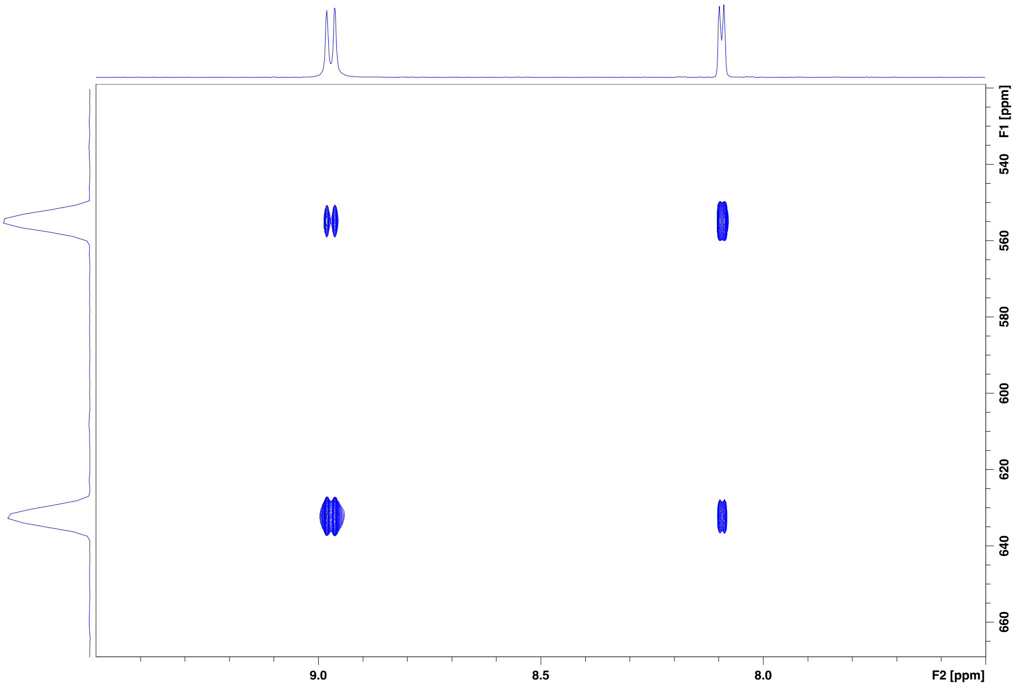

Figure S123. ^{1}H-^{109}Ag HSQC-EX NMR spectrum of **1-Cl** with mixing delay τ = 110 ms (500 MHz, 20 mM in CD_3CN, 298 K). Auto-peak integrals I_{AA} = 44871444, I_{BB} = 34544847. Exchange peak integrals I_{AB} = 9633591, I_{BA} = 9988781.

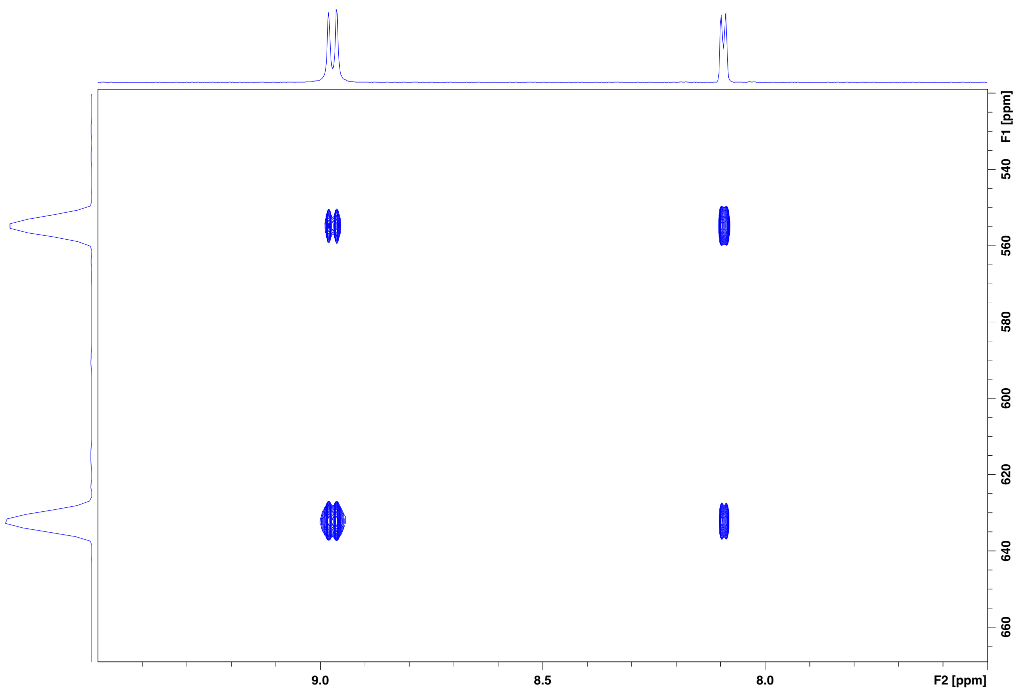

Figure S124. ^{1}H-^{109}Ag HSQC-EX NMR spectrum of **1-Cl** with mixing delay τ = 160 ms (500 MHz, 20 mM in CD_3CN, 298 K). Auto-peak integrals I_{AA} = 40416544, I_{BB} = 28059930. Exchange peak integrals I_{AB} = 11701781, I_{BA} = 12298368.

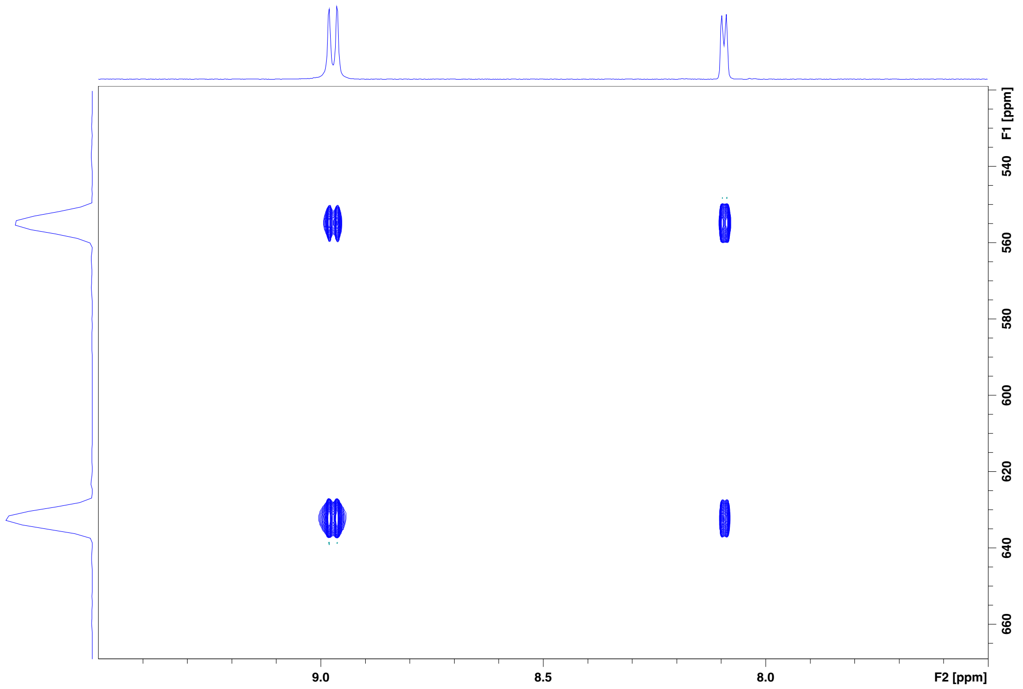

Figure S125. ^{1}H-^{109}Ag HSQC-EX NMR spectrum of **1-Cl** with mixing delay τ = 210 ms (500 MHz, 20 mM in CD_3CN, 298 K). Auto-peak integrals I_{AA} = 35547217, I_{BB} = 23524435. Exchange peak integrals I_{AB} = 12723913, I_{BA} = 13781779.

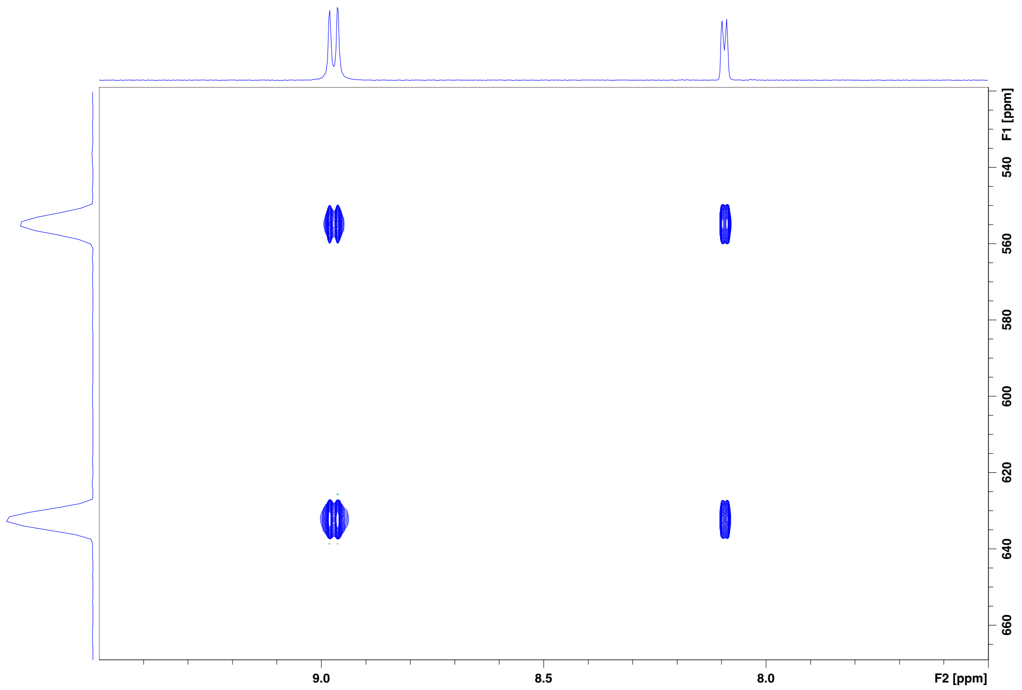

Figure S126. ^{1}H-^{109}Ag HSQC-EX NMR spectrum of **1-Cl** with mixing delay τ = 260 ms (500 MHz, 20 mM in CD_3CN, 298 K). Auto-peak integrals I_{AA} = 32102212, I_{BB} = 19747709. Exchange peak integrals I_{AB} = 13375386, I_{BA} = 14622693.

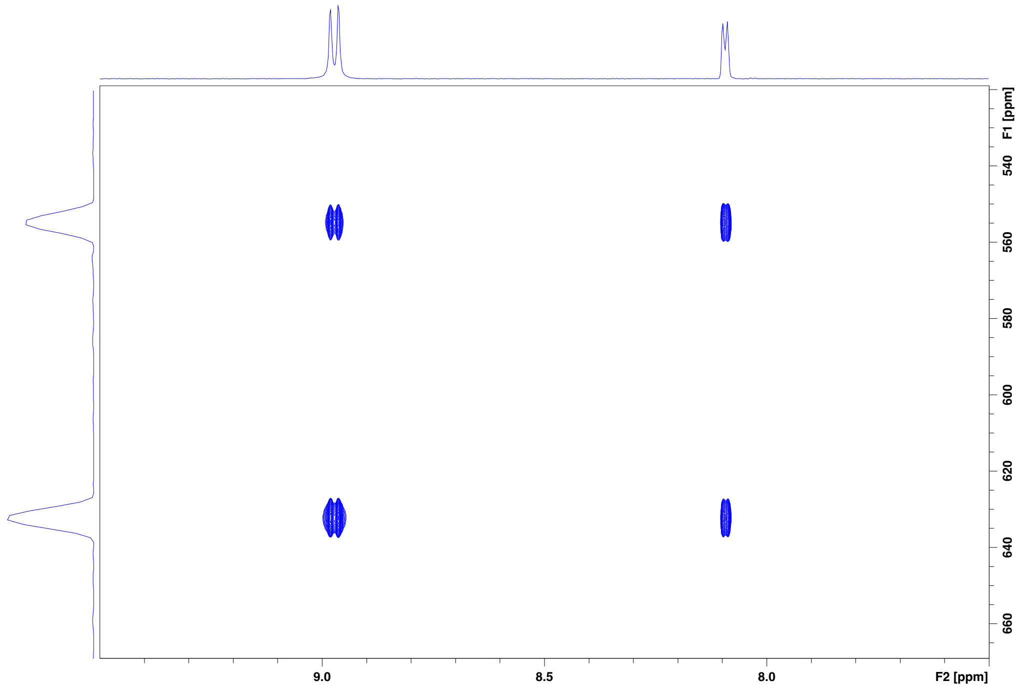

Figure S127. ^{1}H-^{109}Ag HSQC-EX NMR spectrum of **1-Cl** with mixing delay τ = 310 ms (500 MHz, 20 mM in CD_3CN, 298 K). Auto-peak integrals I_{AA} = 29400026, I_{BB} = 16951450. Exchange peak integrals I_{AB} = 13576259, I_{BA} = 14774803.

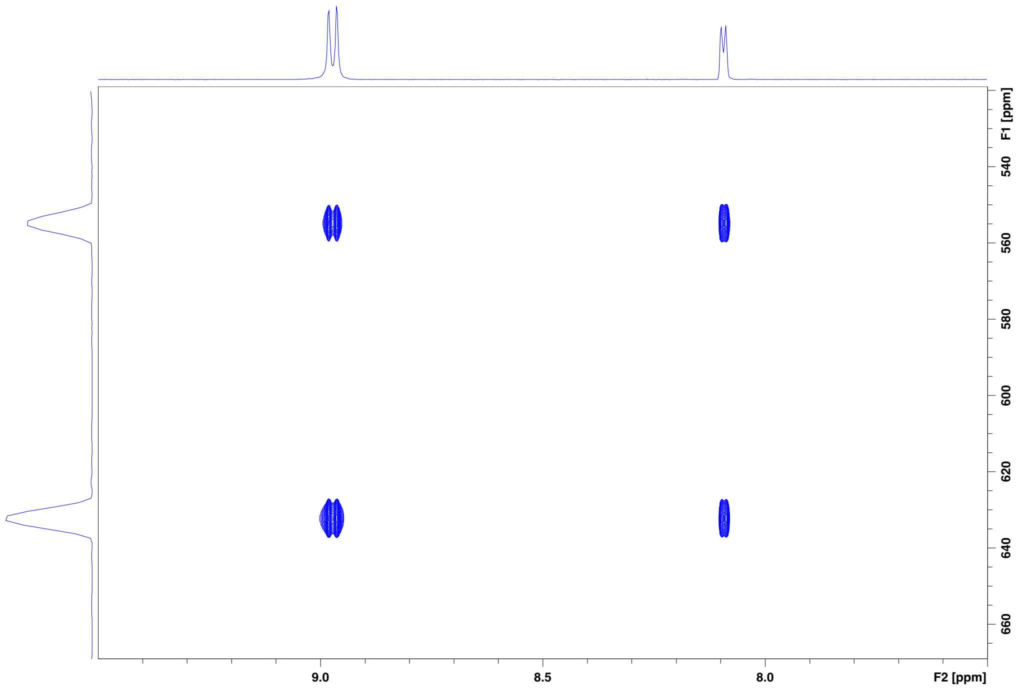

Figure S128. ^{1}H-^{109}Ag HSQC-EX NMR spectrum of **1-Cl** with mixing delay τ = 360 ms (500 MHz, 20 mM in CD_3CN, 298 K). Auto-peak integrals I_{AA} = 26641207, I_{BB} = 14744625. Exchange peak integrals I_{AB} = 13579412, I_{BA} = 14618267.

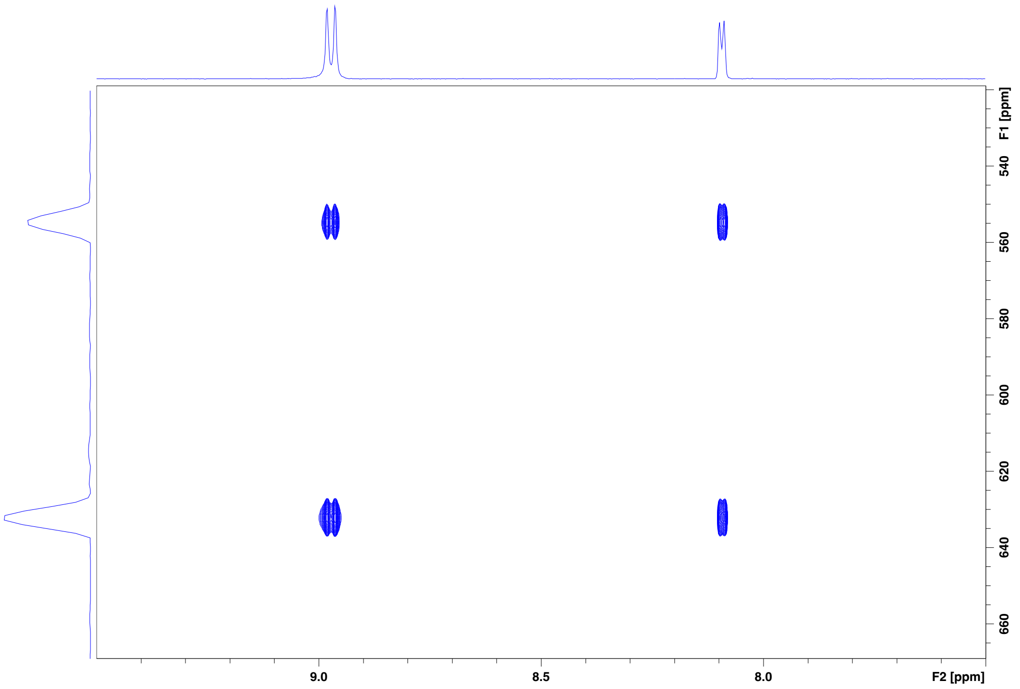

Figure S129. ^{1}H-^{109}Ag HSQC-EX NMR spectrum of **1-Cl** with mixing delay τ = 410 ms (500 MHz, 20 mM in CD_3CN, 298 K). Auto-peak integrals I_{AA} = 24324876, I_{BB} = 12887322. Exchange peak integrals I_{AB} = 12875318, I_{BA} = 14092401.

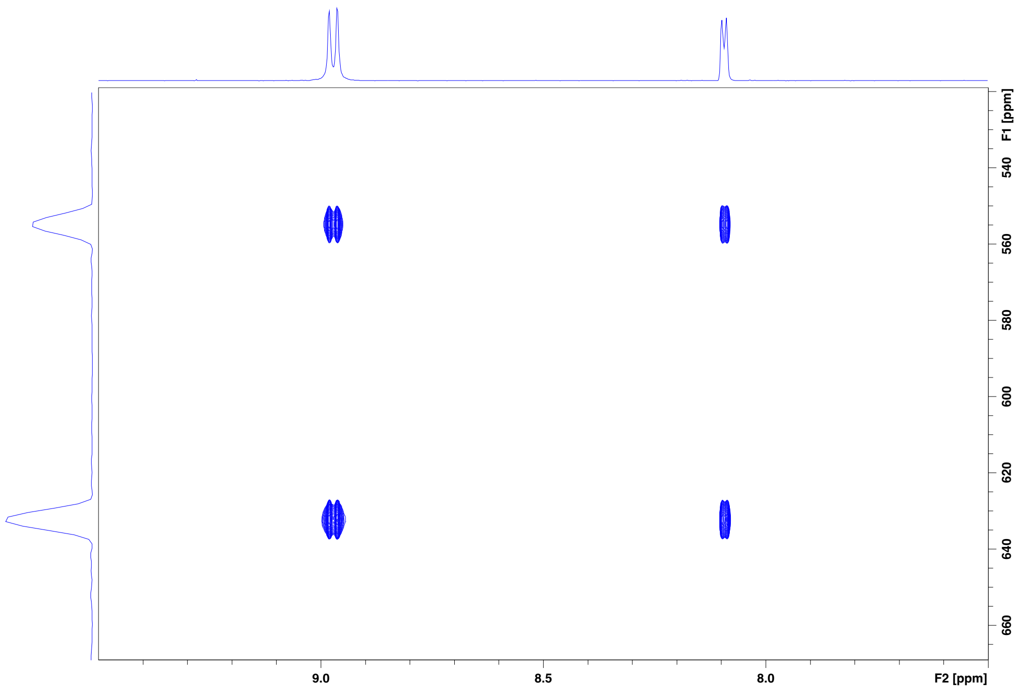

Figure S130. ^{1}H-^{109}Ag HSQC-EX NMR spectrum of **1-Cl** with mixing delay τ = 510 ms (500 MHz, 20 mM in CD_3CN, 298 K). Auto-peak integrals I_{AA} = 20916347, I_{BB} = 10376768. Exchange peak integrals I_{AB} = 11896803, I_{BA} = 13167610.

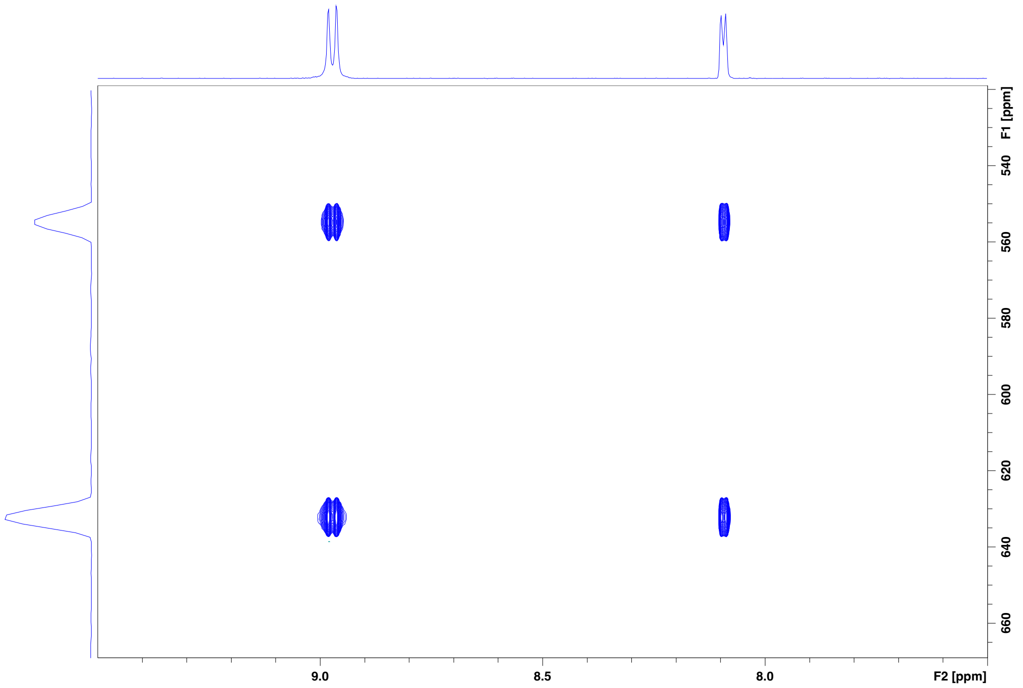

Figure S131. ^{1}H-^{109}Ag HSQC-EX NMR spectrum of **1-Cl** with mixing delay τ = 610 ms (500 MHz, 20 mM in CD_3CN, 298 K). Auto-peak integrals I_{AA} = 17154733, I_{BB} = 8223700. Exchange peak integrals I_{AB} = 10340746, I_{BA} = 11142539.

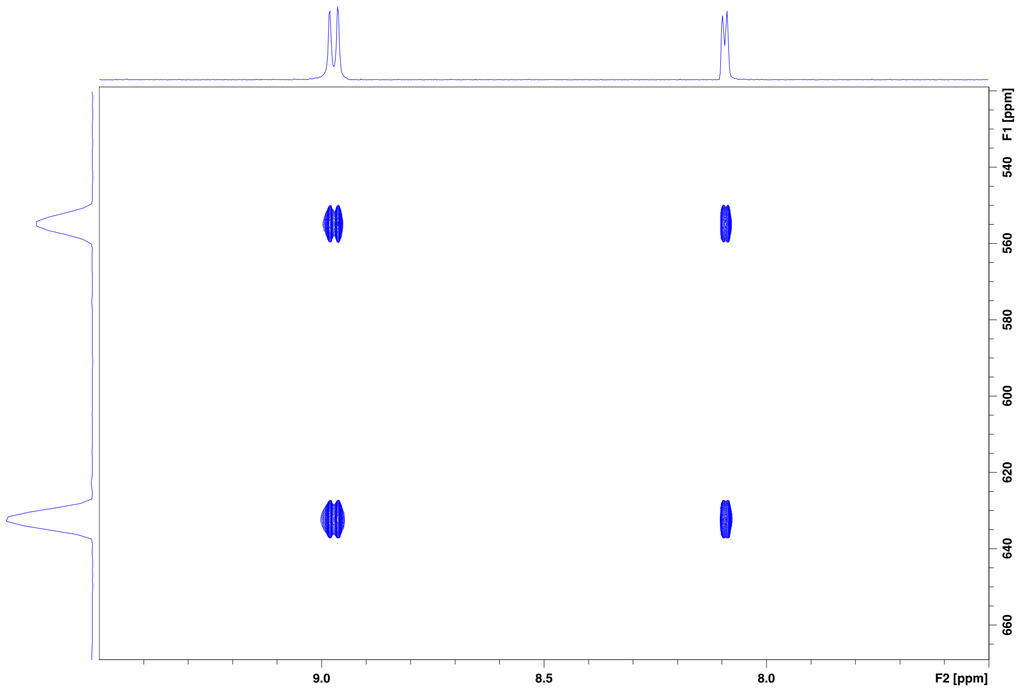

Figure S132. ^{1}H-^{109}Ag HSQC-EX NMR spectrum of **1-Cl** with mixing delay τ = 810 ms (500 MHz, 20 mM in CD_3CN, 298 K). Auto-peak integrals I_{AA} = 11716138, I_{BB} = 5621795. Exchange peak integrals I_{AB} = 7421094, I_{BA} = 8106201.

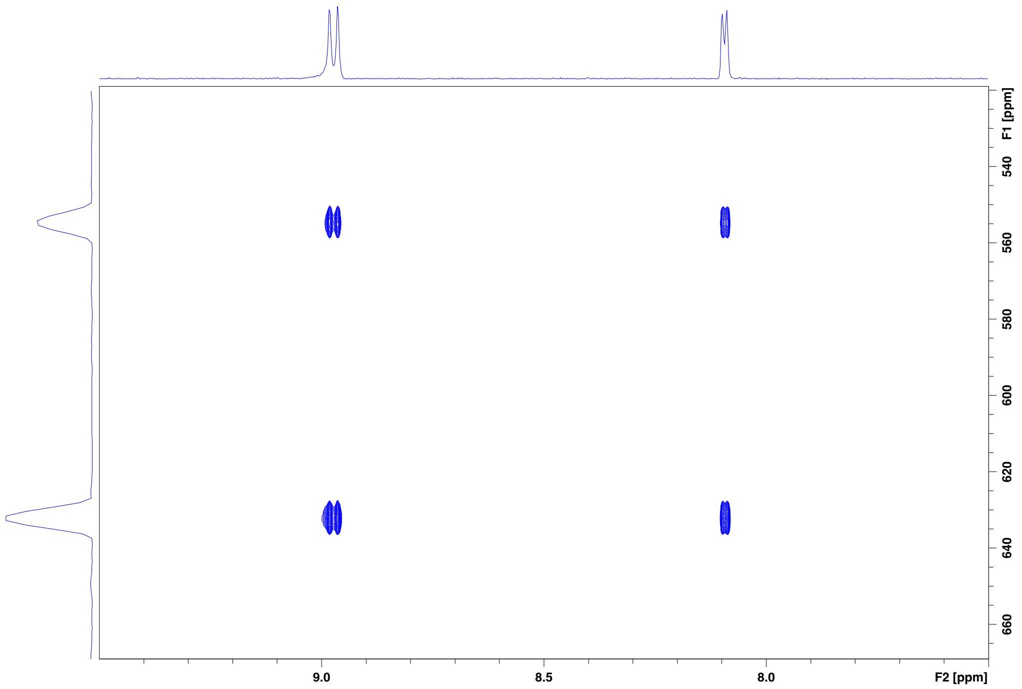

Figure S133. ^{1}H-^{109}Ag HSQC-EX NMR spectrum of **1-Cl** with mixing delay τ = 1010 ms (500 MHz, 20 mM in CD_3CN, 298 K). Auto-peak integrals I_{AA} = 6813088, I_{BB} = 2973538. Exchange peak integrals I_{AB} = 4281700, I_{BA} = 4430850.

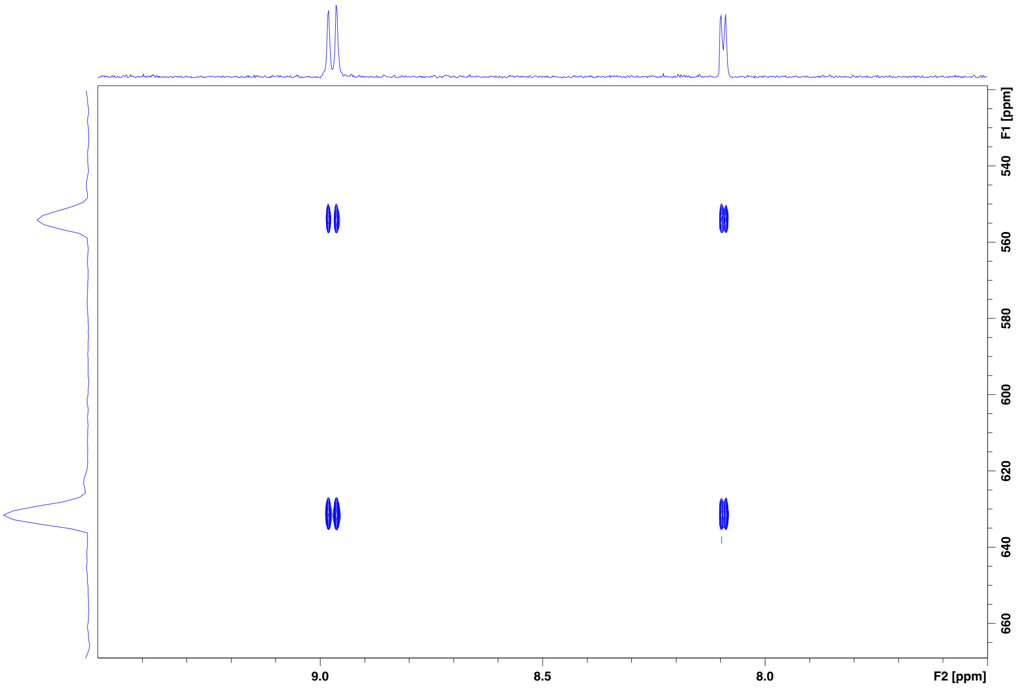

Figure S134. ^{1}H-^{109}Ag HSQC-EX NMR spectrum of **1-Cl** with mixing delay τ = 1510 ms (500 MHz, 20 mM in CD_3CN, 298 K). Auto-peak integrals I_{AA} = 1044974, I_{BB} = 340585. Exchange peak integrals I_{AB} = 158456, I_{BA} = 704698.

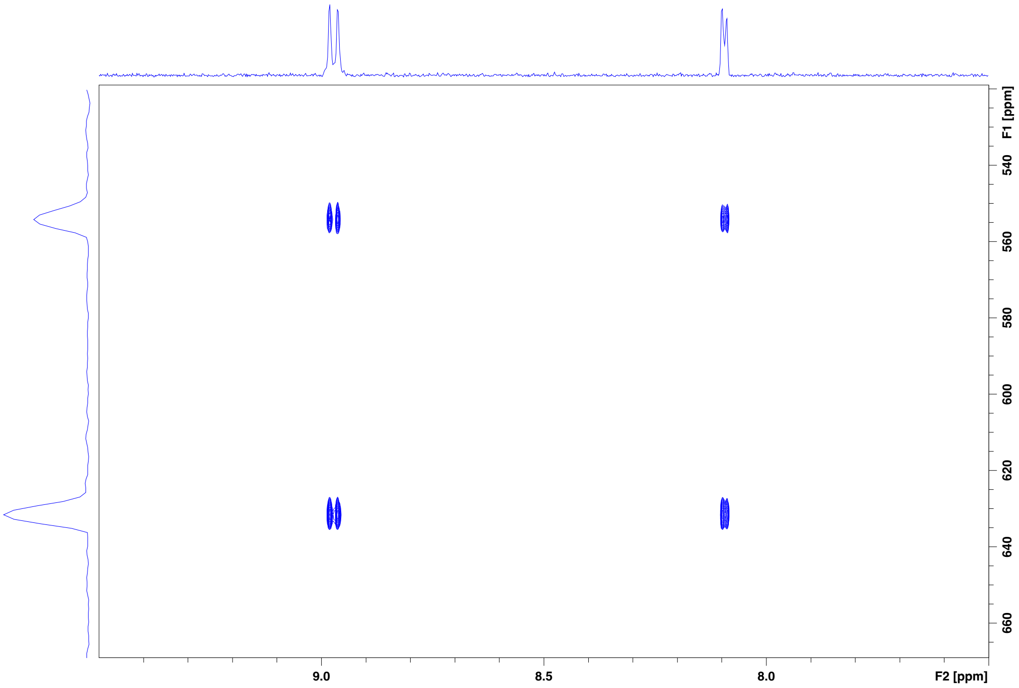

Figure S135. ^{1}H-^{109}Ag HSQC-EX NMR spectrum of **1-Cl** with mixing delay τ = 2010 ms (500 MHz, 20 mM in CD_3CN, 298 K). Auto-peak integrals I_{AA} = -477976, I_{BB} = -311342. Exchange peak integrals I_{AB} = -429869, I_{BA} = -397991.

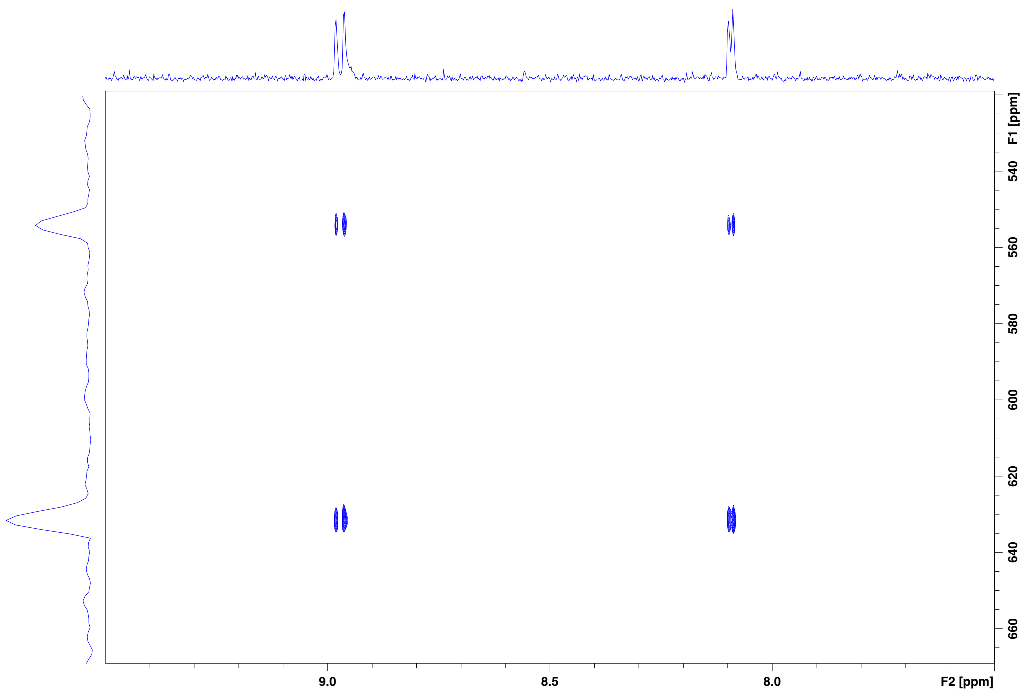

Figure S136. ^{1}H-^{109}Ag HSQC-EX NMR spectrum of **1-Cl** with mixing delay τ = 2510 ms (500 MHz, 20 mM in CD_3CN, 298 K). Auto-peak integrals I_{AA} = 72037, I_{BB} = -37681. Exchange peak integrals I_{AB} = -19112, I_{BA} = -53597.

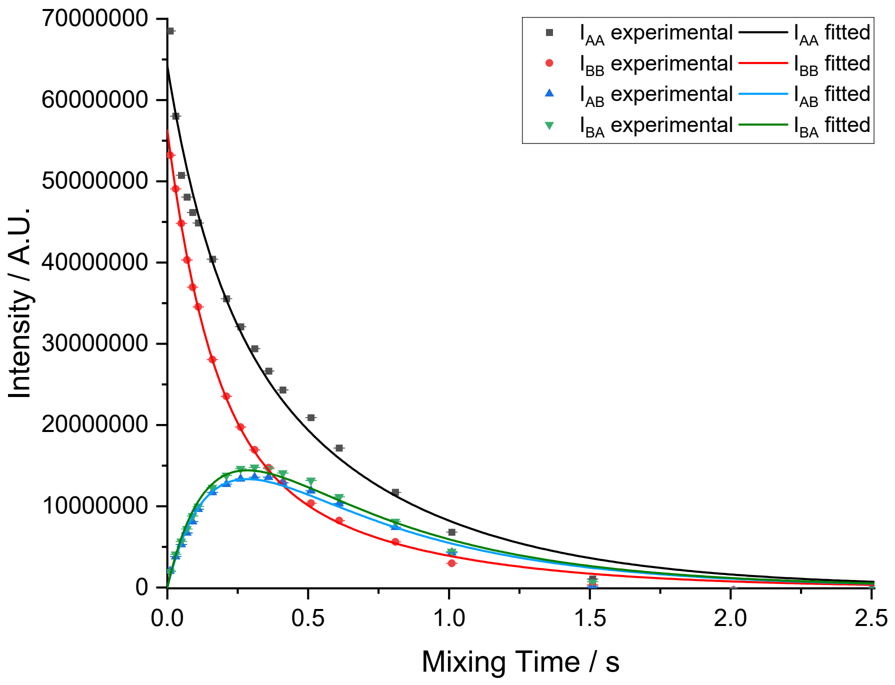

Figure S137. Decay of auto-peak and exchange peak volume of **1-Cl** at 298 K (20 mM in CD_3CN) with integral error bars.

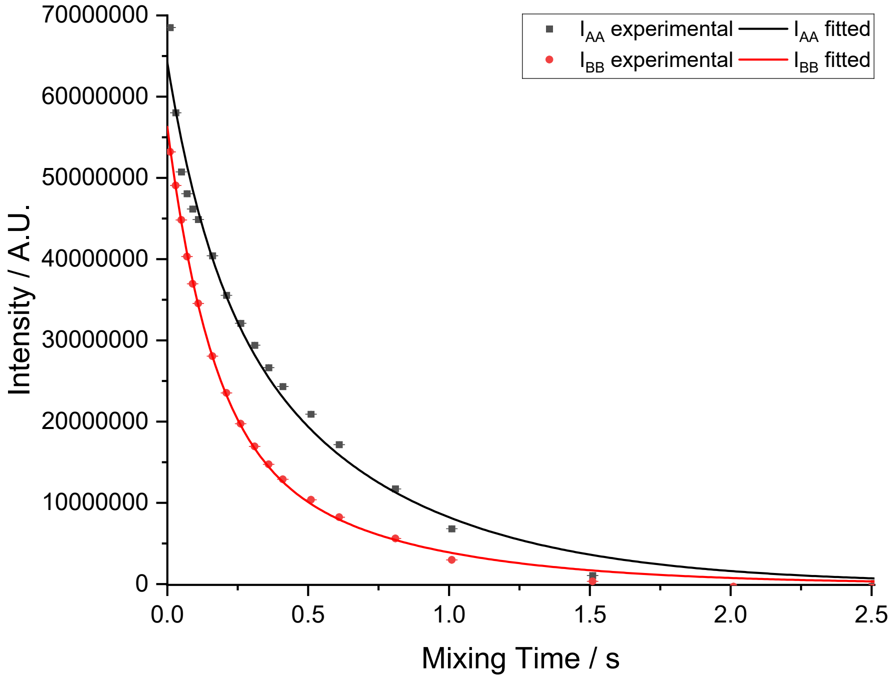

Figure S138. Decay of auto-peak volume of **1-Cl** at 298 K (20 mM in CD_3CN) with integral error bars.

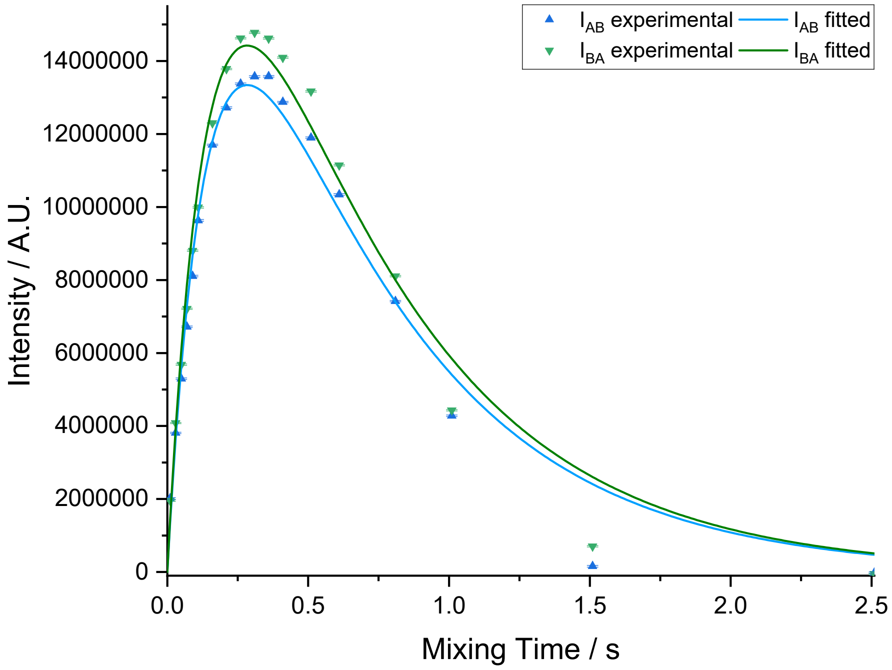

Figure S139. Decay of exchange peak volume of **1-Cl** at 298 K (20 mM in CD_3CN) with integral error bars.

4.9 Dilution Control Experiment for **1-Cl** at 298 K

In addition to the ^{1}H-^{1}H ROESY control experiments, the ^{1}H-^{109}Ag HSQC-EX experiment on **1-Cl** was repeated under six-fold dilution (3 mM).

The data were fitted to the mathematical description (Section 4.5), with a magnetization transfer rate constant ($k_{1\text{-}Cl}^{298\ K}$ = 2.89(30) Hz). The signal-to-noise ratio at 3 mM was lower, resulting in increased error in the fit. Despite this, the rate constant determined at 20 mM was within the 95% confidence window of the value determined at 3 mM. The good agreement between values implies there is a minimal effect of concentration on the rate of silver exchange, and further supports an intramolecular exchange mechanism.

Table S5 shows the auto- and exchange peak volumes at each mixing times. Each row of the table corresponds to a ^{1}H-^{109}Ag HSQC-EX NMR spectrum (Figure S140-S154). These data are fitted (Figure S155-S157) to the equations detailed in section 4.5, optimized over the four signals, to extract the signal decay parameters and the magnetization transfer rate constant $k_{1\text{-}Cl}^{298\ K}$.

This experiment was performed at 3 mM concentration in CD_3CN at 298 K.

Table S5. Volume of auto-peak and exchange peaks in **1-Cl** [3 mM] at 298 K.

Mixing Time (τ / s)	Major auto-peak (I_{AA})	Minor auto-peak (I_{BB})	Major to minor exchange peak (I_{AB})	Minor to Major exchange peak (I_{AB})
0.010	83706803	58185383	2561947	1198811
0.030	73033232	50243593	5204774	3899362
0.050	66268188	44547808	8129527	5908173
0.070	62655626	41300101	9819204	9438297
0.090	55043048	37920805	10848776	11780941
0.110	51655610	34762790	13185405	11907753
0.160	43800161	28518907	12839382	12464097
0.210	37103242	21380622	15485985	16802218
0.260	31621681	16691324	14073696	14907889
0.310	29437192	16332821	16472141	13840689
0.360	26154406	15550745	13865961	16430692
0.410	25249239	11767351	16943157	14345864
0.510	16187007	8181199	11516099	10512651
0.610	14651492	5407536	9698430	8635652
0.810	4498767	4040090	3420009	4449297

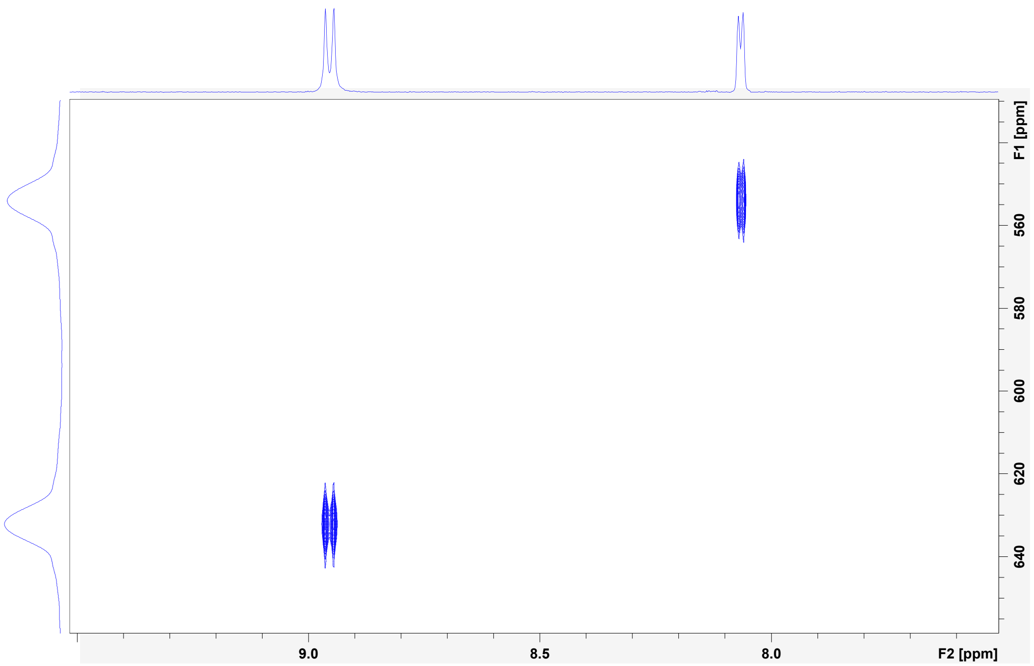

Figure S140. ^{1}H-^{109}Ag HSQC-EX NMR spectrum of **1-Cl** with mixing delay τ = 10 ms (500 MHz, 3 mM in CD_3CN, 298 K). Auto-peak integrals I_{AA} = 83706803, I_{BB} = 58185383. Exchange peak integrals I_{AB} = 2561947, I_{BA} = 1198811.

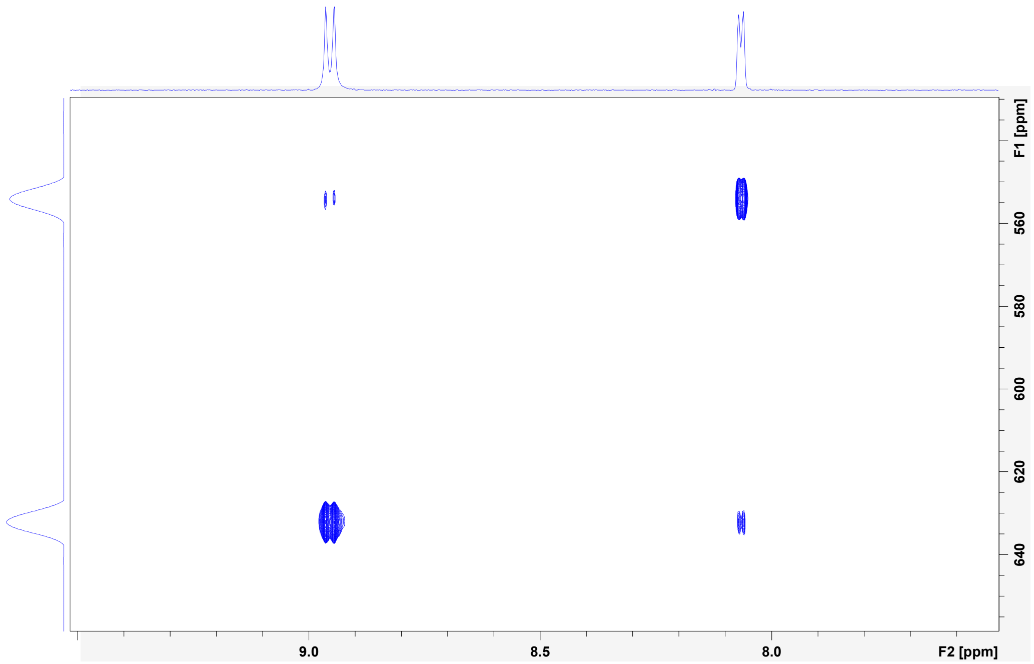

Figure S141. ^{1}H-^{109}Ag HSQC-EX NMR spectrum of **1-Cl** with mixing delay τ = 30 ms (500 MHz, 3 mM in CD_3CN, 298 K). Auto-peak integrals I_{AA} = 73033232, I_{BB} = 50243593. Exchange peak integrals I_{AB} = 5204774, I_{BA} = 3899362.

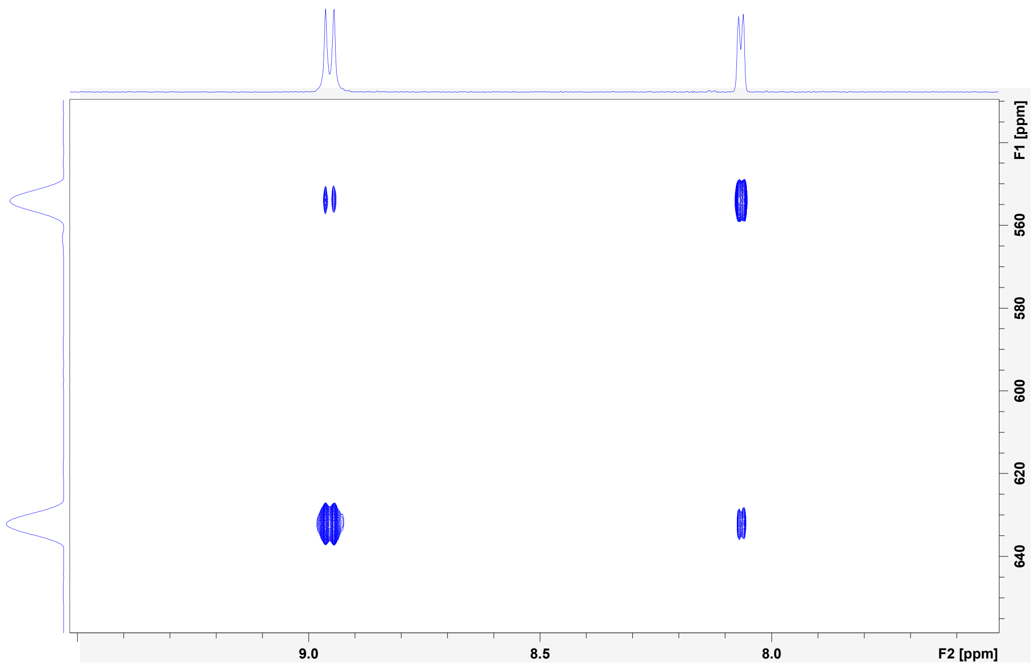

Figure S142. ^{1}H-^{109}Ag HSQC-EX NMR spectrum of **1-Cl** with mixing delay τ = 50 ms (500 MHz, 3 mM in CD_3CN, 298 K). Auto-peak integrals I_{AA} = 66268188, I_{BB} = 44547808. Exchange peak integrals I_{AB} = 8129527, I_{BA} = 5908173.

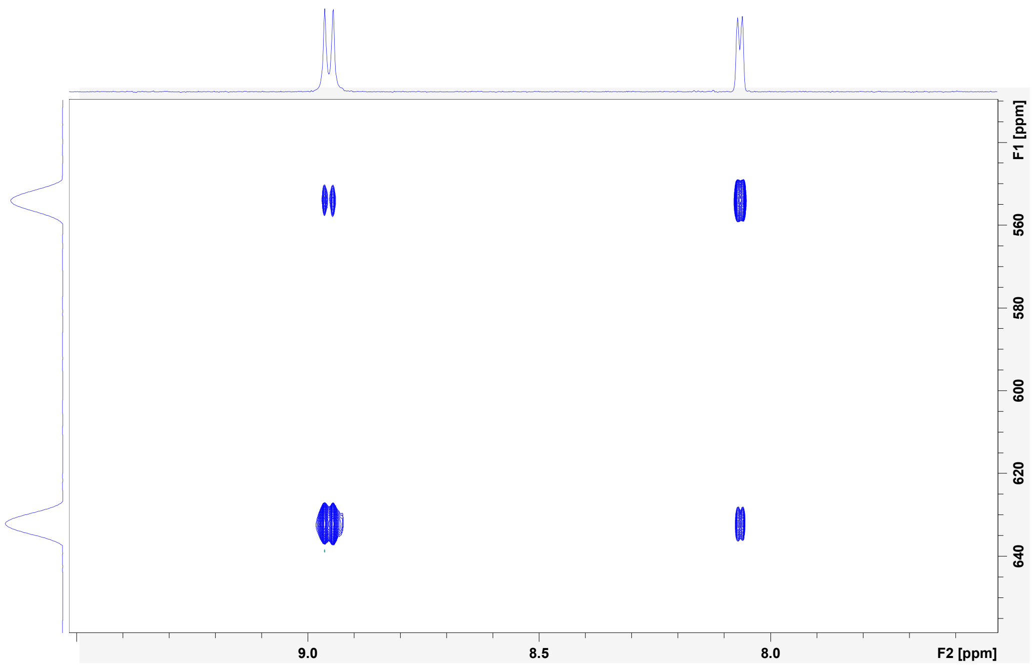

Figure S143. ^{1}H-^{109}Ag HSQC-EX NMR spectrum of **1-Cl** with mixing delay τ = 70 ms (500 MHz, 3 mM in CD_3CN, 298 K). Auto Auto-peak integrals I_{AA} = 62655626, I_{BB} = 41300101. Exchange peak integrals I_{AB} = 9819204, I_{BA} = 9438297.

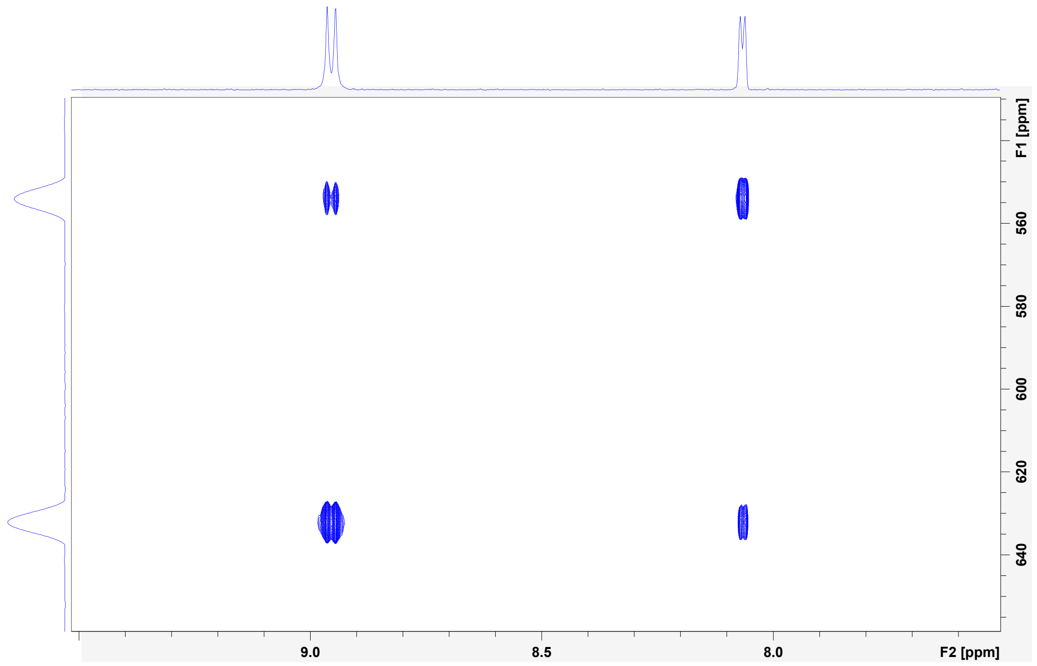

Figure S144. ^{1}H-^{109}Ag HSQC-EX NMR spectrum of **1-Cl** with mixing delay τ = 90 ms (500 MHz, 3 mM in CD_3CN, 298 K). Auto-peak integrals I_{AA} = 55043048, I_{BB} = 37920805. Exchange peak integrals I_{AB} = 10848776, I_{BA} = 11780941.

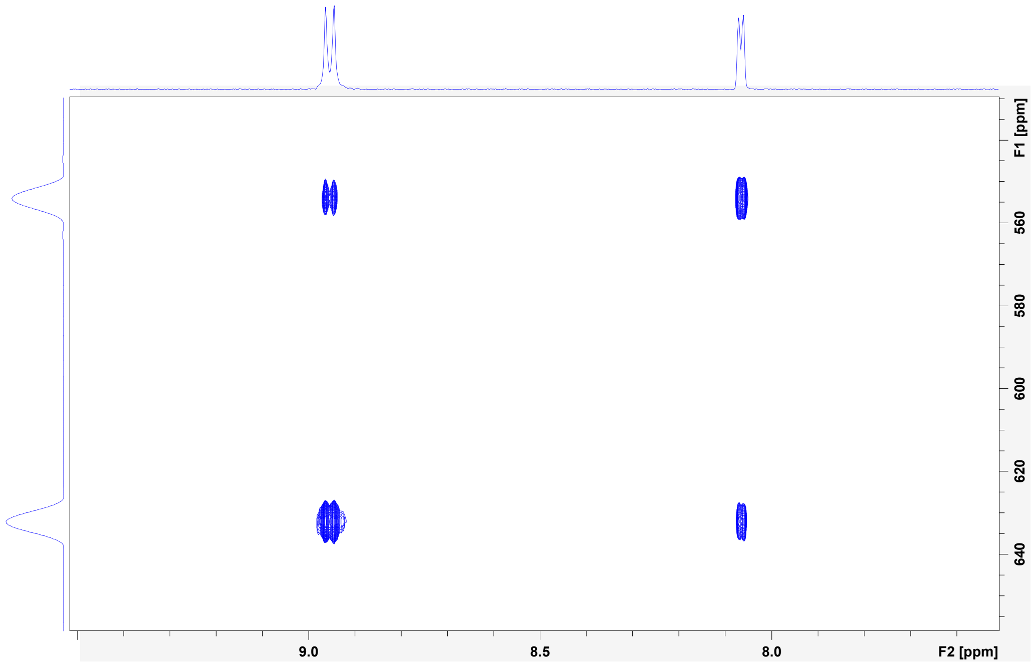

Figure S145. ^{1}H-^{109}Ag HSQC-EX NMR spectrum of **1-Cl** with mixing delay τ = 110 ms (500 MHz, 3 mM in CD_3CN, 298 K). Auto-peak integrals I_{AA} = 51655610, I_{BB} = 34762790. Exchange peak integrals I_{AB} = 13185405, I_{BA} = 11907753.

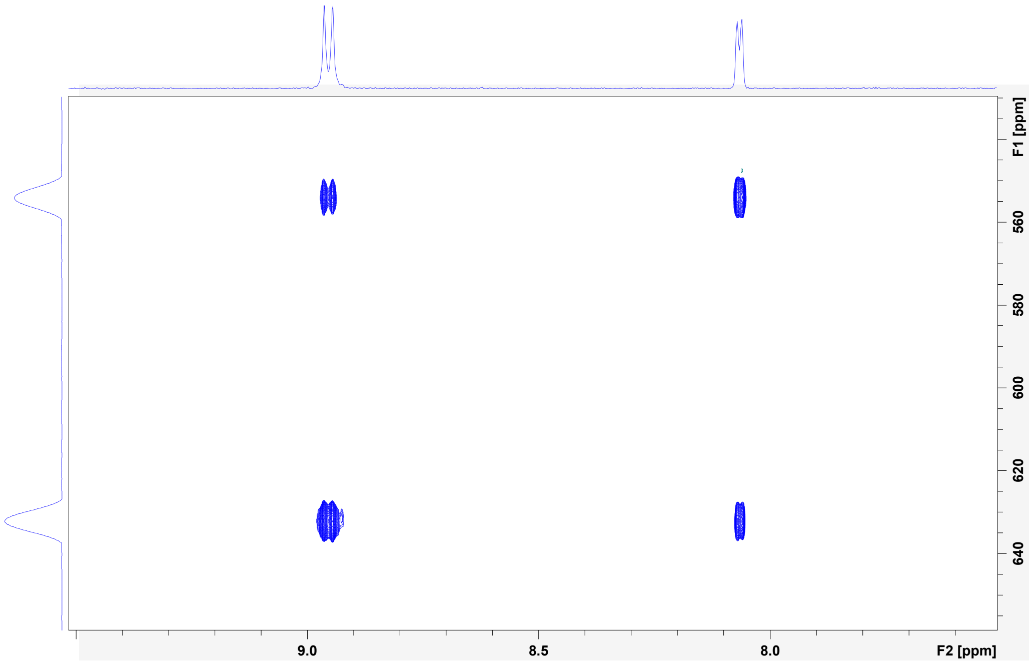

Figure S146. ^{1}H-^{109}Ag HSQC-EX NMR spectrum of **1-Cl** with mixing delay τ = 160 ms (500 MHz, 3 mM in CD_3CN, 298 K). Auto-peak integrals I_{AA} = 43800161, I_{BB} = 28518907. Exchange peak integrals I_{AB} = 12839382, I_{BA} = 12464097.

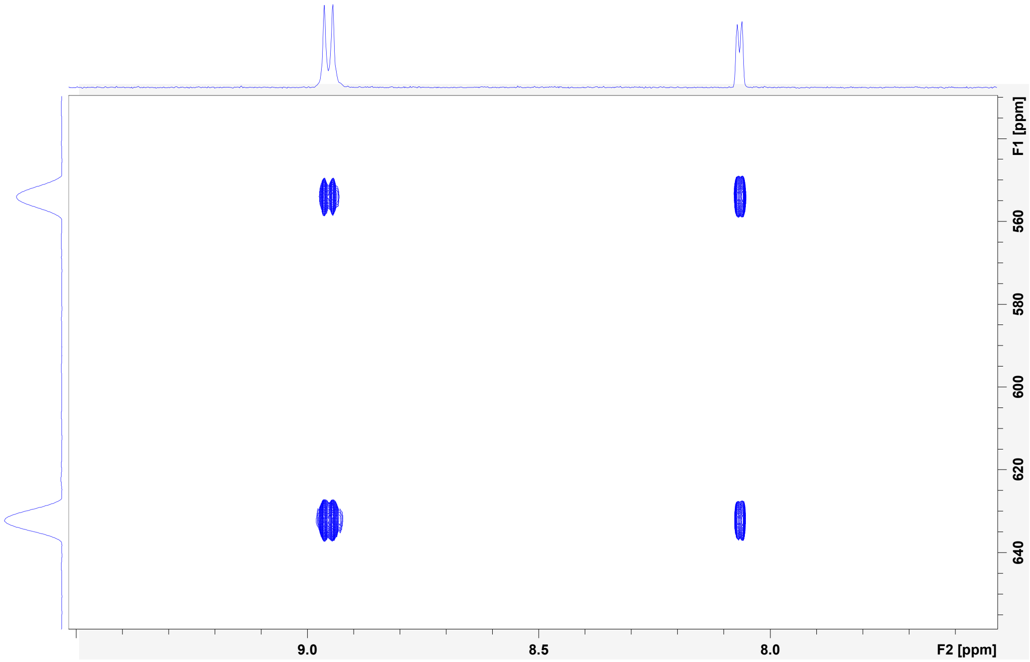

Figure S147. ^{1}H-^{109}Ag HSQC-EX NMR spectrum of **1-Cl** with mixing delay τ = 210 ms (500 MHz, 3 mM in CD_3CN, 298 K). Auto-peak integrals I_{AA} = 37103242, I_{BB} = 21380622. Exchange peak integrals I_{AB} = 15485985, I_{BA} = 16802218.

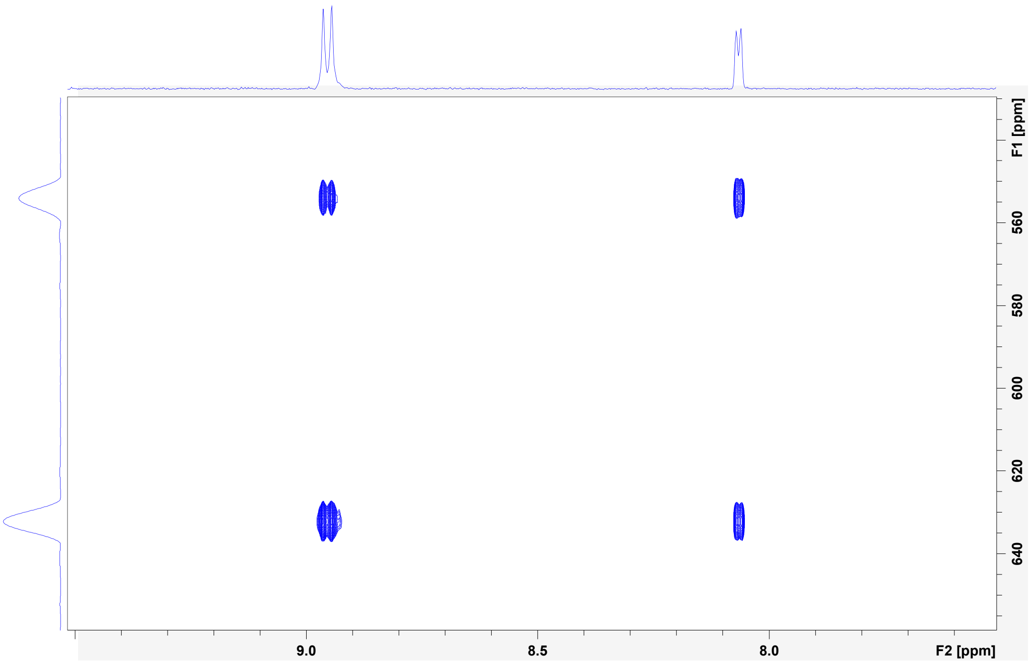

Figure S148. ^{1}H-^{109}Ag HSQC-EX NMR spectrum of **1-Cl** with mixing delay τ = 260 ms (500 MHz, 3 mM in CD_3CN, 298 K). Auto-peak integrals I_{AA} = 31621681, I_{BB} = 16691324. Exchange peak integrals I_{AB} = 14073696, I_{BA} = 14907889.

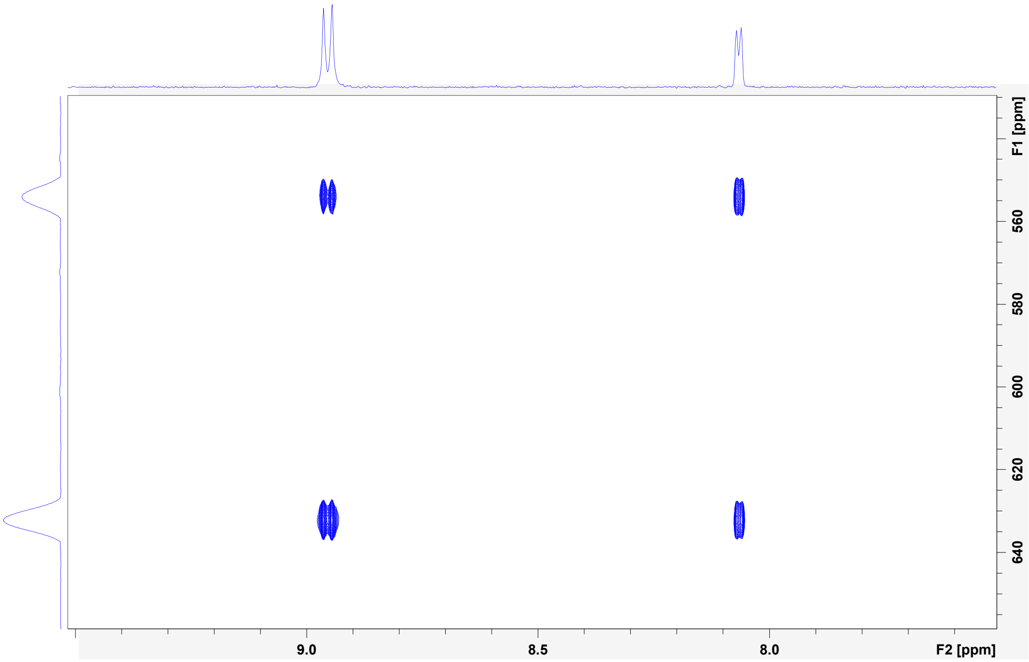

Figure S149. ^{1}H-^{109}Ag HSQC-EX NMR spectrum of **1-Cl** with mixing delay τ = 310 ms (500 MHz, 3 mM in CD_3CN, 298 K). Auto-peak integrals I_{AA} = 29437192, I_{BB} = 16332821. Exchange peak integrals I_{AB} = 16472141, I_{BA} = 13840689.

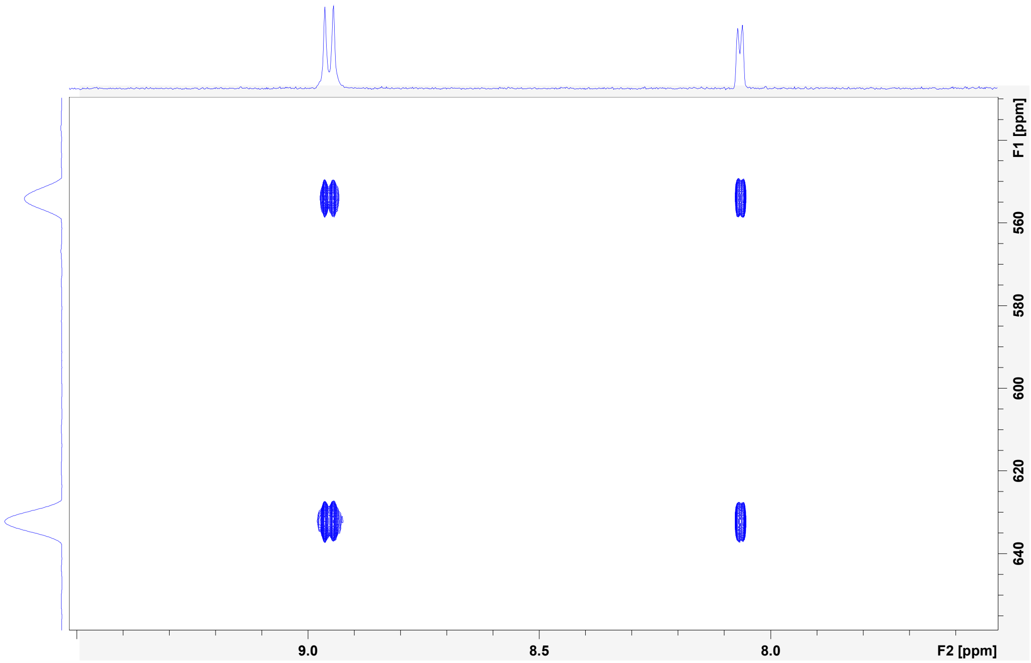

Figure S150. ^{1}H-^{109}Ag HSQC-EX NMR spectrum of **1-Cl** with mixing delay τ = 360 ms (500 MHz, 3 mM in CD_3CN, 298 K). Auto-peak integrals I_{AA} = 26154406, I_{BB} = 15550745. Exchange peak integrals I_{AB} = 13865961, I_{BA} = 16430692.

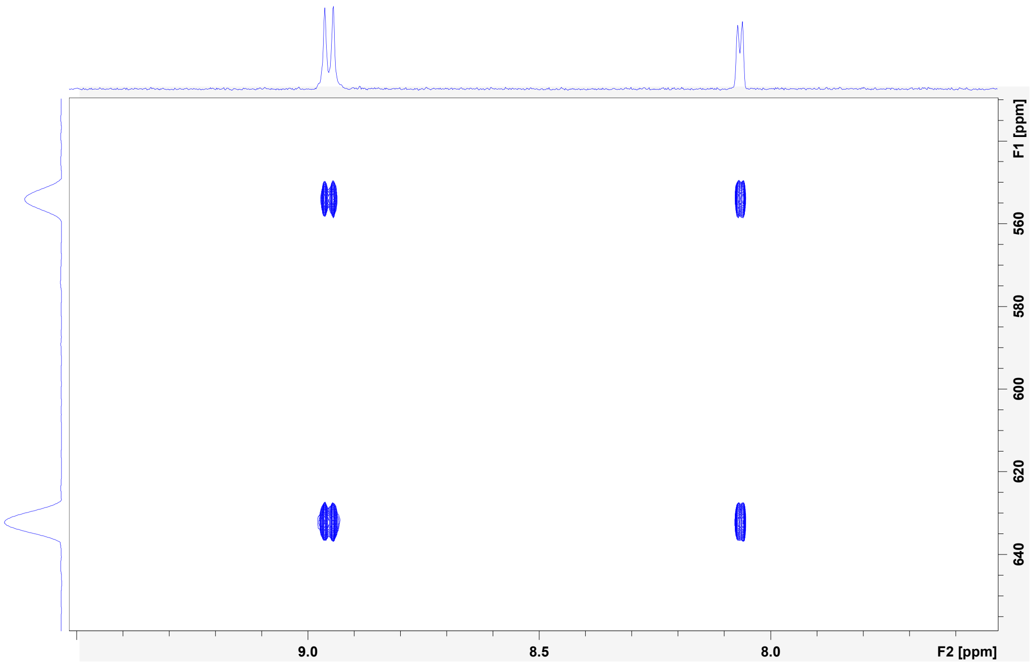

Figure S151. ^{1}H-^{109}Ag HSQC-EX NMR spectrum of **1-Cl** with mixing delay τ = 410 ms (500 MHz, 3 mM in CD_3CN, 298 K). Auto-peak integrals I_{AA} = 25249239, I_{BB} = 11767351. Exchange peak integrals I_{AB} = 16943157, I_{BA} = 14345864.

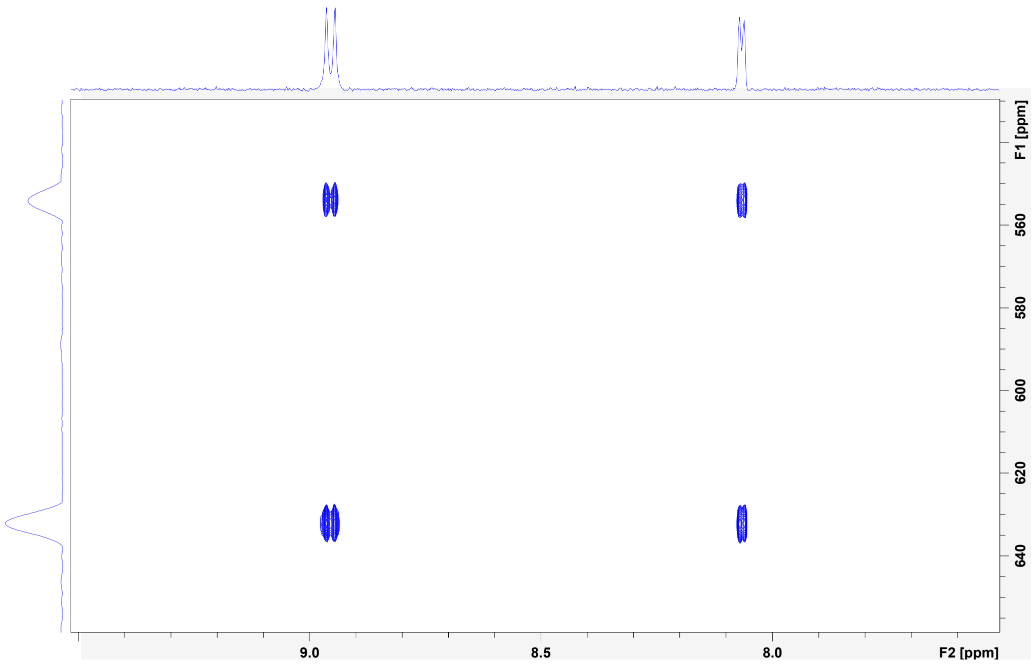

Figure S152. ^{1}H-^{109}Ag HSQC-EX NMR spectrum of **1-Cl** with mixing delay τ = 510 ms (500 MHz, 3 mM in CD_3CN, 298 K). Auto-peak integrals I_{AA} = 16187007, I_{BB} = 8181199. Exchange peak integrals I_{AB} = 11516099, I_{BA} = 10512651.

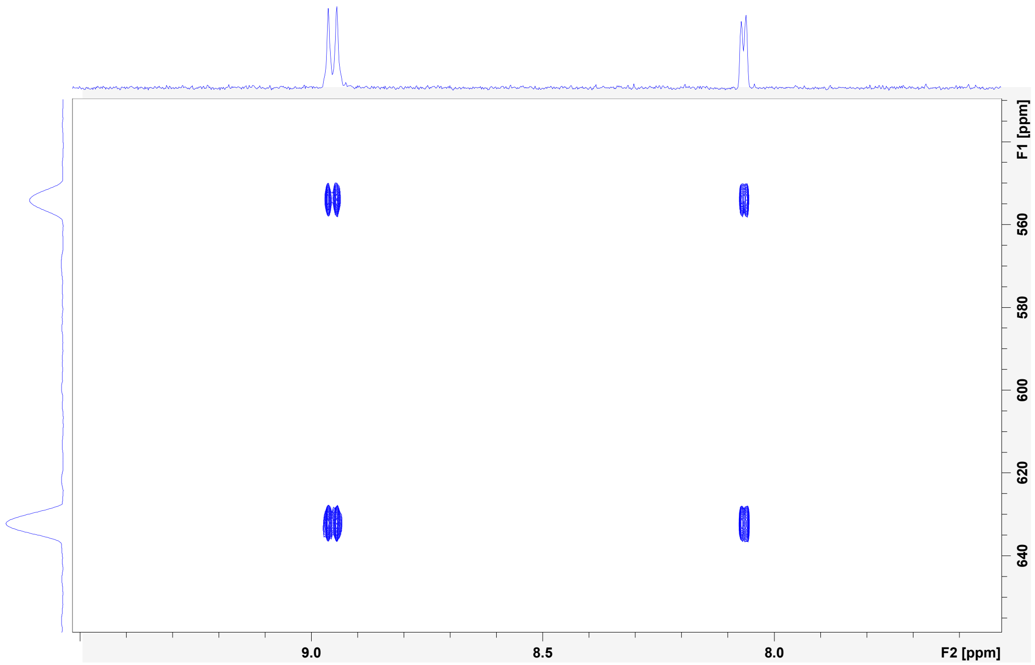

Figure S153. ^{1}H-^{109}Ag HSQC-EX NMR spectrum of **1-Cl** with mixing delay τ = 610 ms (500 MHz, 3 mM in CD_3CN, 298 K). Auto-peak integrals I_{AA} = 14651492, I_{BB} = 5407536. Exchange peak integrals I_{AB} = 9698430, I_{BA} = 8635652.

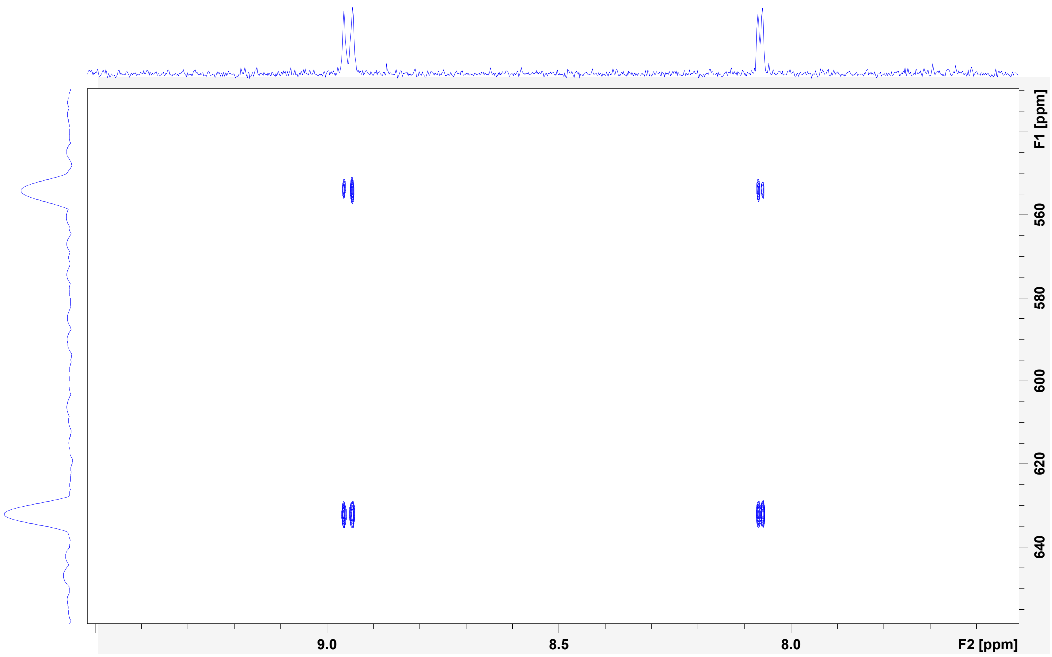

Figure S154. ^{1}H-^{109}Ag HSQC-EX NMR spectrum of **1-Cl** with mixing delay τ = 810 ms (500 MHz, 3 mM in CD_3CN, 298 K). Auto-peak integrals I_{AA} = 4498767, I_{BB} = 4040090. Exchange peak integrals I_{AB} = 3420009, I_{BA} = 4449297.

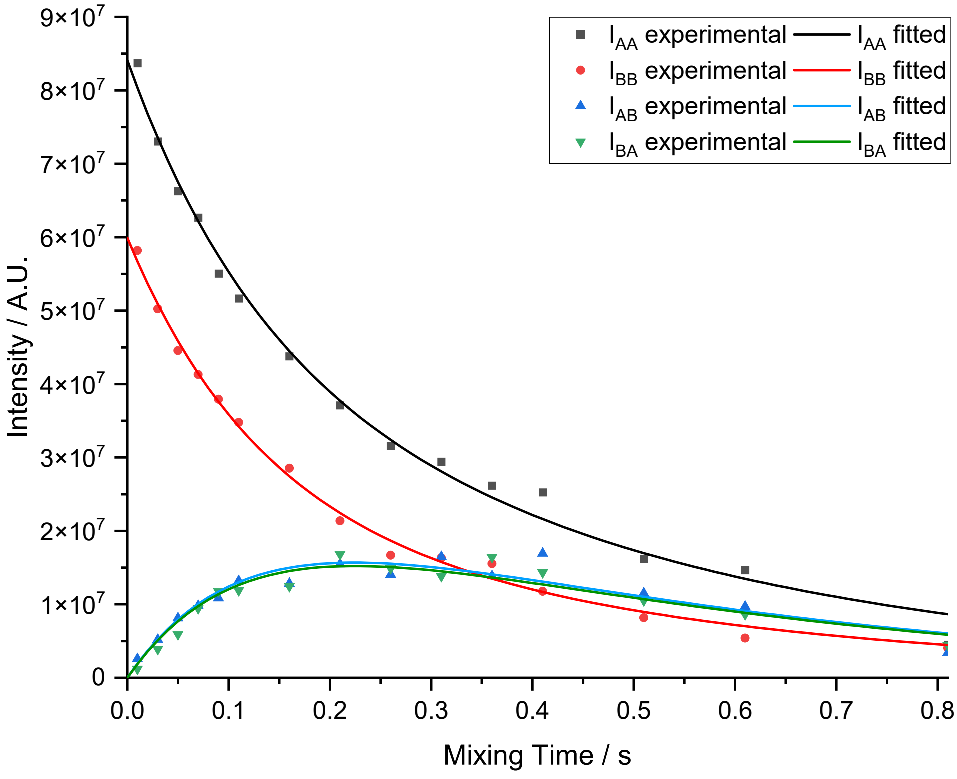

Figure S155. Decay of auto-peak and exchange peak volume of **1-Cl** at 298 K (3 mM in CD_3CN).

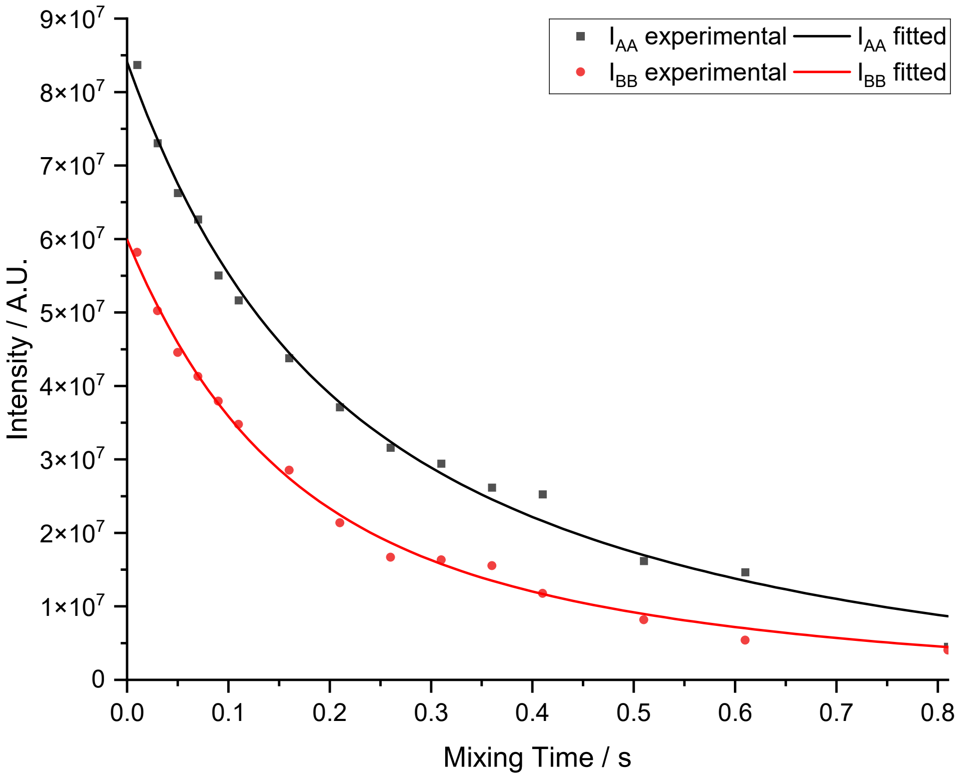

Figure S156. Decay of auto-peak volume of **1-Cl** at 298 K (3 mM in CD_3CN).

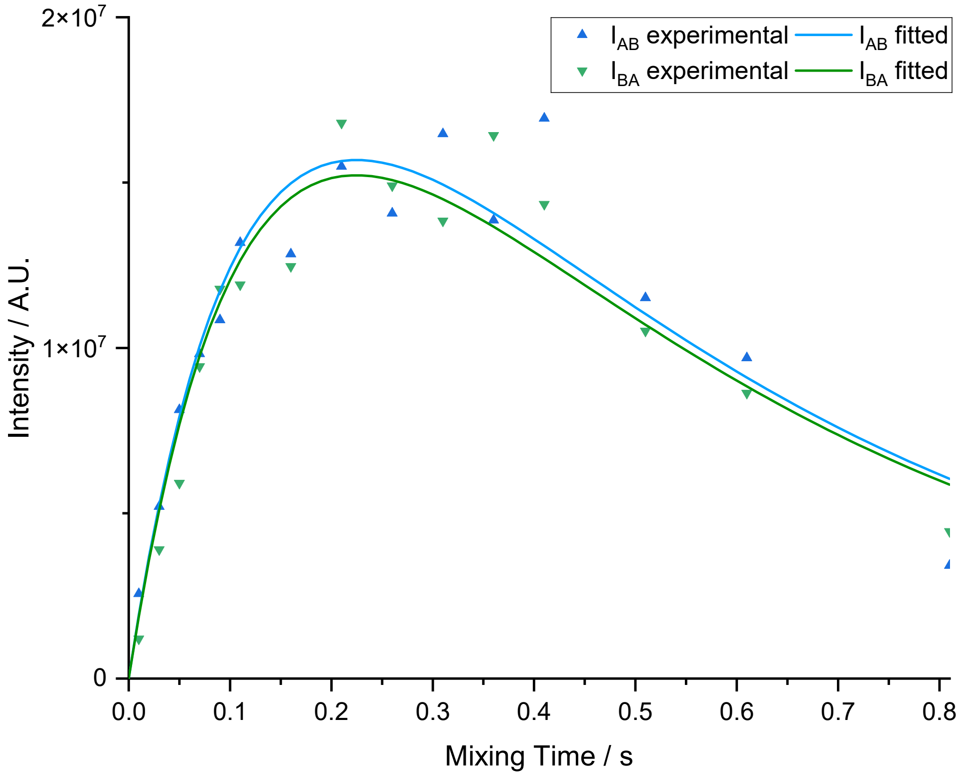

Figure S157. Decay of exchange peak volume of **1-Cl** at 298 K (3 mM in CD_3CN).

4.10 EXSY Control Experiments

^{1}H-^{1}H NOESY and EASY-ROESY[11] experiments were completed to ensure that the exchange between Ag environments was due to an intramolecular rearrangement, as opposed to a disassembly-reassembly process which would show exchange peaks between ^{1}H environments appearing in the ROESY spectra.

If ^{1}H exchange signals were observed between the two sets of ligand ^{1}H environments on the same timeframe as the Ag-exchange, a disassembly-reassembly process is occurring. However, if the ligands are not in exchange on the same timeframe, the Ag-exchange must be occurring *via* an intramolecular rearrangement with the ligands in their initial location.

The EASY-ROESY spectra for **1-Cl**, **1-Br**, and **1-I** show only NOE through space correlations, and no exchange correlation peaks between the same ^{1}H of two ligands are observed. At 400 ms mixing time at 298 K, significant Ag-exchange is occurring, but no ^{1}H exchange. It can be concluded that the Ag exchange is occurring intramolecularly, and not through a disassembly-reassembly mechanism.

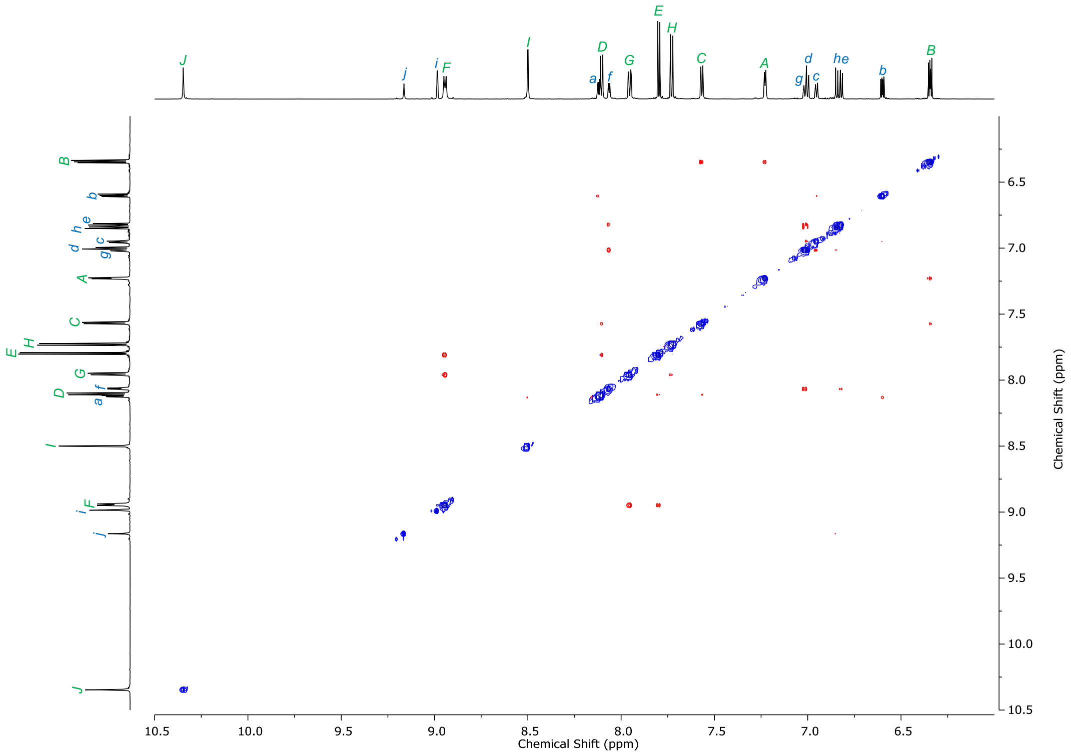

Figure S158. ^{1}H-^{1}H EASY-ROESY spectrum of **1-Cl** with mixing delay τ = 400 ms (700 MHz, 20 mM in CD_3CN, 298 K).

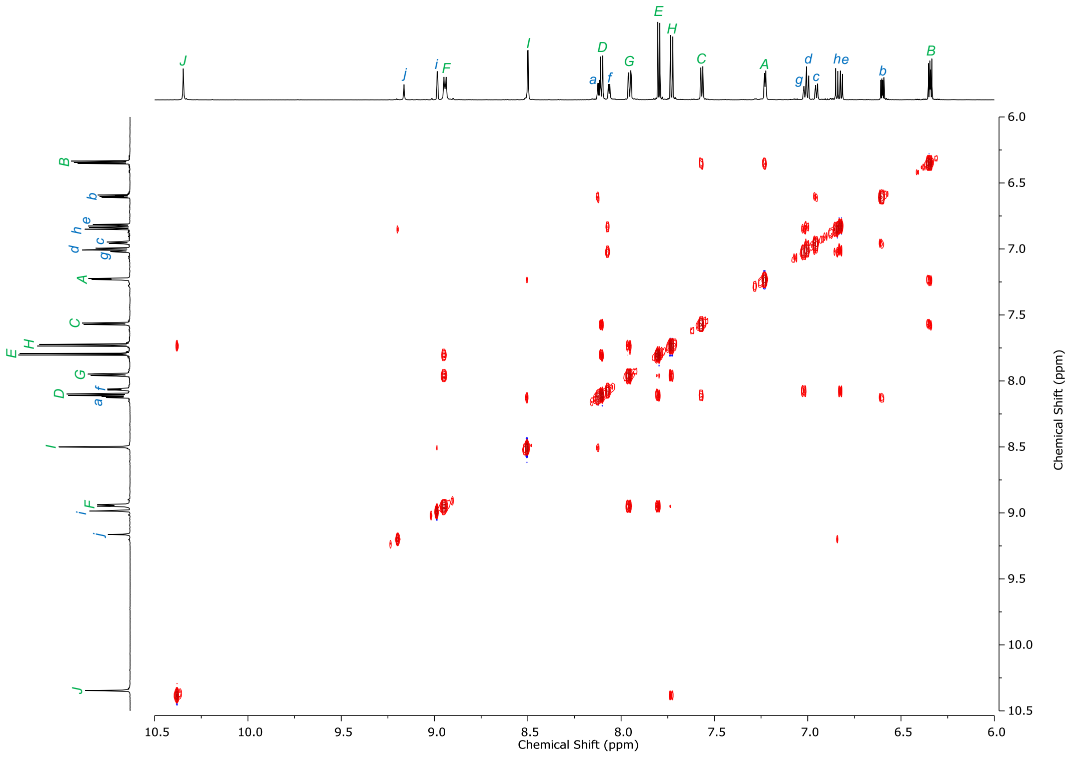

Figure S159. ^{1}H-^{1}H NOESY spectrum of **1-Cl** with mixing delay τ = 1000 ms (700 MHz, 20 mM in CD_3CN, 298 K).

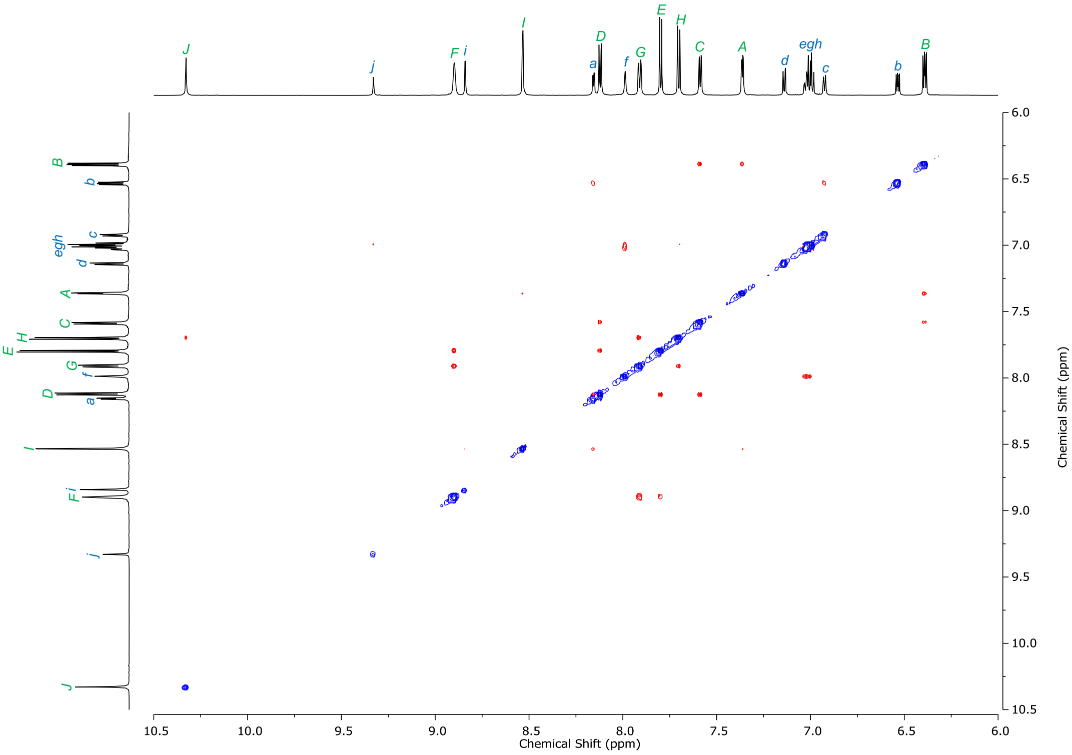

Figure S160. ^{1}H-^{1}H EASY-ROESY spectrum of **1-Br** with mixing delay τ = 400 ms (700 MHz, 15 mM in CD_3CN, 298 K).

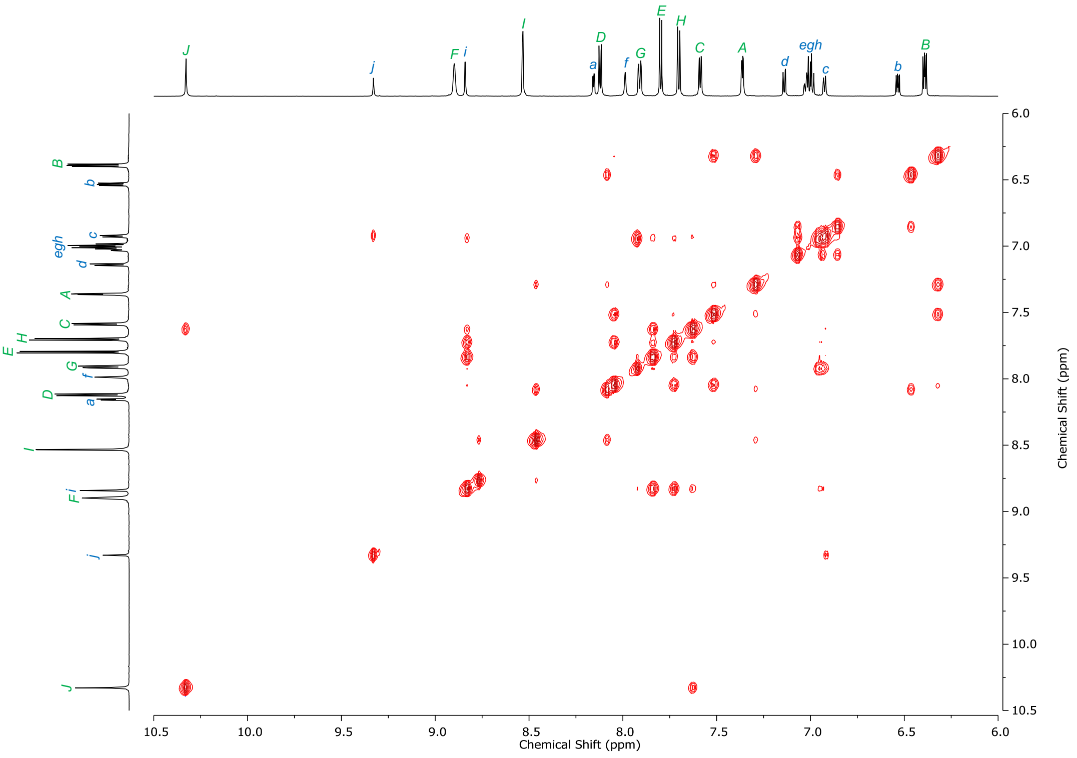

Figure S161. ^{1}H-^{1}H NOESY spectrum of **1-Br** with mixing delay τ = 1000 ms (700 MHz, 15 mM in CD_3CN, 298 K).

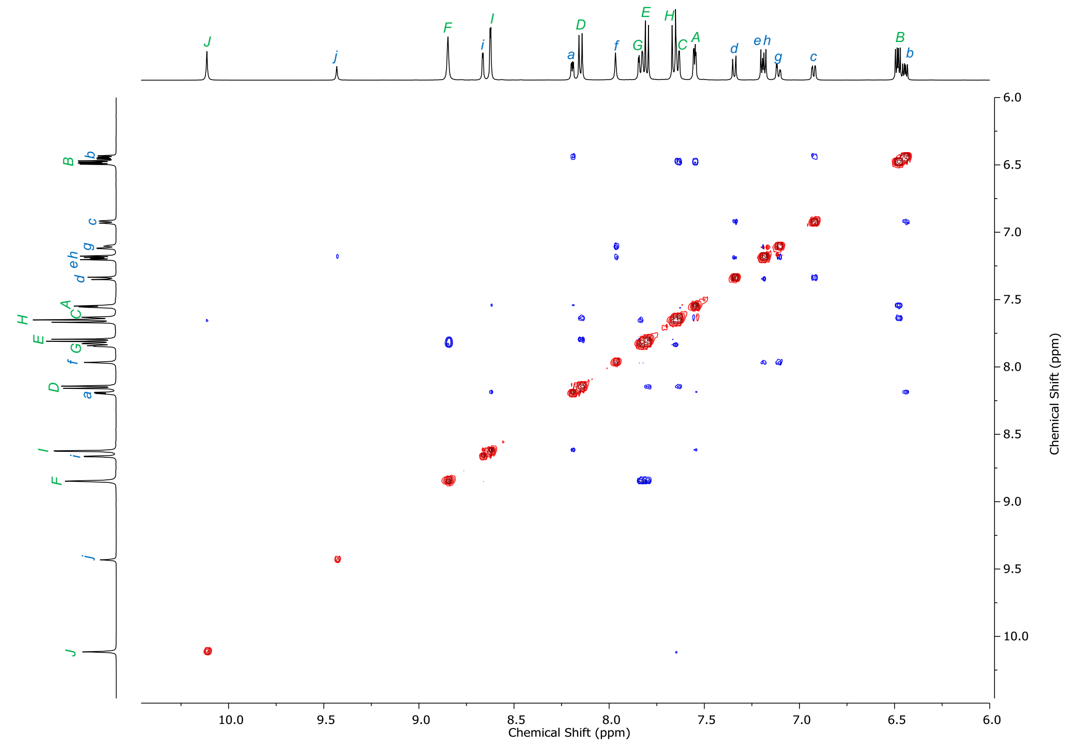

Figure S162. ^{1}H-^{1}H EASY-ROESY spectrum of **1-I** with mixing delay τ = 400 ms (700 MHz, 16 mM in CD_3CN, 298 K).

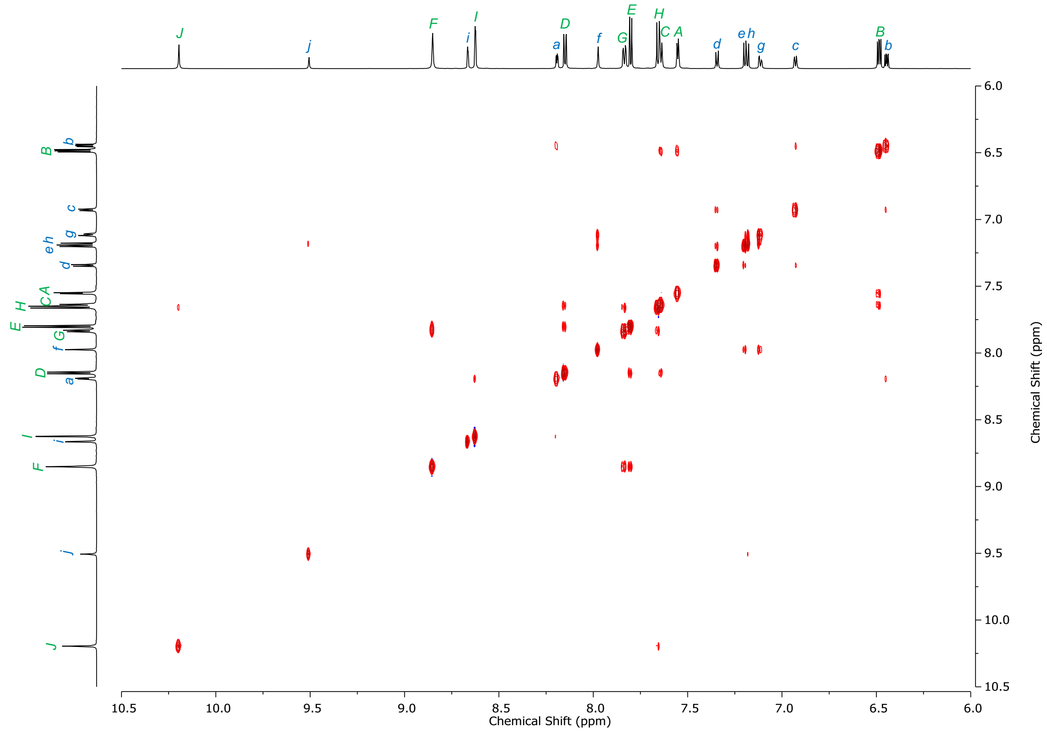

Figure S163. ^{1}H-^{1}H NOESY spectrum of **1-I** with mixing delay τ = 1000 ms (700 MHz, 16 mM in CD_3CN, 298 K).

4.11 Summary and Analysis of ^{1}H-^{109}Ag HSQC-EX Experiments

The concerted rotation of each $Ag^{I}_{3}X$ cluster is observed by ^{1}H-^{109}Ag HSQC-EX NMR spectroscopy, and the total magnetization transfer rate constant $k_{\mathbf{1\text{-}x}}^{T}$ is extracted from the fitted data (section 4.5). The process is an equilibrium between two degenerate $Ag^{I}_{12}X_{4}L_{6}$ complexes (K_{eq}=1, ΔG=0). The rotation of the four vertex $Ag^{I}_{3}X$ clusters is assumed independent of each other, and the concerted rotation of one $Ag^{I}_{3}X$ cluster has no effect on the total $Ag^{I}_{12}X_{4}$ ions.

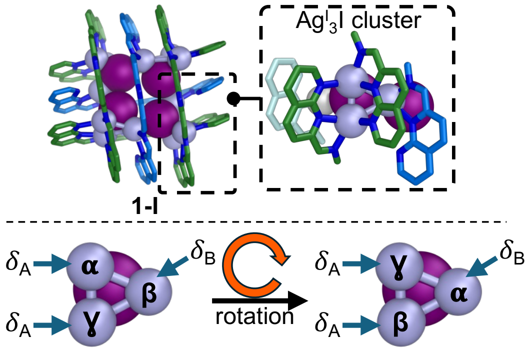

Figure S164. (Top) Schematic isolating the $Ag^{I}_{3}I$ cluster from the full **1-I** structure. (Bottom) Schematic showing the movement of silver ions and the detectable silver(I) NMR signals after one rotation.

In the 3-fold rotation process shown above, one of the silver atoms occupying position A (labeled γ) is transformed into the degenerate position A, that is γ (δ_{A1}) ↔ γ (δ_{A2}) and is therefore unobservable *via* NMR spectroscopy. This same rotational process transforms the silver atom at δ_B into one at δ_A (β (δ_B) ↔ β (δ_A)), as well as the silver atom at δ_A into δ_B, (α (δ_A) ↔ α (δ_B)). The experimentally observed magnetization transfer rate constant $k_{\mathbf{1\text{-}x}}^{T}$ is the sum of forward and reverse rate constants. Thus, the rate constant for the motion of an individual Ag^{I} ion is the value of $k_{A\to B}$ = $k_{B\to A}$ = (½)$k_{\mathbf{1\text{-}x}}^{T}$.

$k_{\mathbf{1\text{-}Cl}}^{298\ K}$ = 2.33(22) Hz, relative σ of ± 9% [20 mM]
$k_{\mathbf{1\text{-}Cl}}^{298\ K}$ = 2.89(30) Hz, relative σ of ±10% [3 mM]
$k_{\mathbf{1\text{-}Br}}^{263\ K}$ = 1.81(13) Hz, relative σ of ± 7% [15 mM]
$k_{\mathbf{1\text{-}I}}^{243\ K}$ = 3.38(14) Hz, relative σ of ± 4% [16 mM]

Exchange rate constants for **1-Cl**, **1-Br** and **1-I** have been calculated from the auto-peak and exchange peak volumes from the ^{1}H-^{109}Ag HSQC-EX data. Using the Eyring equation, Gibbs energy of activation ($\Delta^{‡}G_{\mathbf{1\text{-}x}}$) can be calculated for each system.

$$k_{A\to B} = \frac{1}{2}k_{\mathbf{1-x}}^{\mathrm{T}} = \frac{\kappa k_B T}{h}e^{\frac{-\Delta^{‡}G_{\mathbf{1-x}}}{RT}}$$

$$\Delta^{‡}G_{\mathbf{1-x}} = -RTln(\frac{k_{A\to B}h}{\kappa k_B T})$$

Where $k_{A\to B}$ is the rate constant, κ is the transmission coefficient (assumed to be equal to 1, i.e. every time the system achieves the transition state geometry, it moves on to product and never goes back to reactants), T is temperature, h is Planck constant (6.62607×10^{-34} J·s^{-1}), R is the molar gas constant (8.31446 J·K^{-1}·mol^{-1}), and k_B is the Boltzmann constant (1.380649×10^{-23} J·K^{-1}). h and k_B are assumed to be known to infinite precision.

Table S6. Rate constants and activation barriers for silver exchange in **1-Cl**, **1-Br**, and **1-I**.

Sample	Concentration / mM	Temperature / K	Total exchange rate $k_{1\text{-}x}^{T}$ / s^{-1}	Exchange rate constant $k_{A\to B}$ / s^{-1}	$\Delta^{‡}G_{1\text{-}x}$ / kJ·mol^{-1}	$\Delta^{‡}G_{1\text{-}x}$ / eV
**1-Cl**	20	298.0	2.33(22)	1.17(11)	72.60(23)	0.752(2)
**1-Cl**	3	298.0	2.89(30)	1.45(15)	72.07(26)	0.747(2)
**1-Br**	16	263.0	1.81(13)	0.91(07)	64.35(8)	0.6669(8)
**1-I**	15	243.0	3.38(14)	1.69(07)	58.04(5)	0.6015(5)

The uncertainties in k and in T propagated into $\Delta^{\ddagger}G_{1\text{-}X}$ according to:

$$\Delta^{\ddagger}G^2 = \left(\frac{\partial\Delta^{\ddagger}G}{\partial k_{A\rightarrow B}}\right)_T^2 dk_{A\rightarrow B}{}^2 + \left(\frac{\partial\Delta^{\ddagger}G}{\partial T}\right)_{k_{A\rightarrow B}}^2 dT^2$$

T is known accurately to ± 0.1 K, giving an uncertainty of 0.04% (at 243 K), dT has a negligible contribution to the uncertainty in $\Delta^{\ddagger}G_{1\text{-}X}$. Therefore, uncertainty in $k_{A\rightarrow B}$, $dk_{A\rightarrow B}$, is propagated into $\Delta^{\ddagger}G_{1\text{-}X}$ *via*:

$$d\Delta^{\ddagger}G = \frac{RT}{k} dk_{A\rightarrow B}$$

The Gibbs free energy of activation can be calculated for all four data sets ~~three metal-organic grids~~.

1-Cl [20 mM] : $\Delta^{\ddagger}G_{1\text{-}Cl}$ = 72.6(2) kJ·mol^{-1}, relative σ of ± 0.3%

1-Cl [3 mM] : $\Delta^{\ddagger}G_{1\text{-}Cl}$ = 72.1(3) kJ·mol^{-1}, relative σ of ± 0.4%

1-Br [16 mM] : $\Delta^{\ddagger}G_{1\text{-}Br}$ = 64.35(8) kJ·mol^{-1}, relative σ of ± 0.1%

1-I [15 mM] : $\Delta^{\ddagger}G_{1\text{-}I}$ = 58.04(5) kJ·mol^{-1}, relative σ of ± 0.1%

It can be concluded that the exchange **1-Cl** to **1-Br** lowers $\Delta^{\ddagger}G_{1\text{-}X}$ by 8.3(3) kJ·mol^{-1}; **1-Cl** to **1-I** lowers $\Delta^{\ddagger}G_{1\text{-}X}$ by 14.6(3) kJ·mol^{-1}; and **1-Br** to **1-I** lowers $\Delta^{\ddagger}G_{1\text{-}X}$ by 6.31(13) kJ·mol^{-1}.

Assuming $\Delta^{\ddagger}G_{1\text{-}X}$ has minimal change due to temperature. Utilising $\Delta^{\ddagger}G_{1\text{-}Br}$, $k_{1\text{-}Br}^{298\text{ K}}$ can be calculated as 65.0 Hz, 28-fold accelerated relative to $k_{1\text{-}Cl}^{298\text{ K}}$. Utilising $\Delta^{\ddagger}G_{1\text{-}I}$, $k_{1\text{-}I}^{298\text{ K}}$ can be calculated as 832 Hz, 360-fold accelerated relative to $k_{1\text{-}Cl}^{298\text{ K}}$, and 13-fold accelerated relative to $k_{1\text{-}Br}^{298\text{ K}}$.

Comparisons of the 72.1(3) kJ·mol^{-1} barrier determined at 3 mM, and the 72.6(2) kJ·mol^{-1} barrier determined at 20 mM concentration of **1-Cl** demonstrated that concentration has negligible effect on the barrier to silver flux. The lower signal-to-noise ratios for the 3 mM sample resulted in marginally higher errors.

5. X-ray crystallography of 1

5.1 X-ray crystallography of grid **1-I**

Specific refinement details:

Crystals of **1-I** were grown by vapor diffusion of diethyl ether into an acetonitrile solution of **1-I** with excess tetra-*n*-butylammonium hexafluorophosphate, yielding red crystal blocks of $[Ag_{12}I_4L_6](PF_6)_8$•$(Et_2O)_1$•$(CH_3CN)_{12.5}$. These crystals diffracted synchrotron radiation strongly and were collected on 1% transmission. One complete ligand, portions of three other ligands and three silver ions were modeled as disordered over two locations. Bond lengths and angles within the chemically identical disordered ligands were restrained to be similar to each other using SAME. The hexafluorophosphate anions within the structure show evidence of substantial disorder. The eight anions were modeled as disordered over twelve lattice sites, most of which displayed further disorder over up to three locations. Geometric and thermal parameter restraints were applied to all disordered anions. SADI restraints were used to achieve a reasonable geometry for the better resolved sites while DFIX restraints were used for some low occupancy sites. While many lattice sites were overall full occupancy the equivalent of around one anion was highly disordered across numerous lower occupancy sites. The occupancies of these sites were first allowed to freely refine to ensure that all anions had been located before the sum of their free variables was fixed. In order to obtain a geometrically reasonable model for the highly disordered acetonitrile and diethyl ether molecules DFIX and DANG restraints were used. Some lower occupancy disordered anions and solvents were modeled with isotropic thermal parameters and thermal parameter restraints were applied to facilitate realistic modelling of all disordered solvents. The hydrogen atoms of some disordered acetonitrile molecules could not be located in the electron density map and were not included in the model. Eleven reflections, mostly at low angle, have been omitted from the final refinement. These reflections are likely affected by beam damage or absorption or possibly a small amount of twinning that could not be resolved during data processing. CheckCIF gives 1B alert. This crystal structure is deposited in the CCDC with deposition number 2377078.

Table S7. Crystal data and structure refinement for 1-I_awhv023c.

Identification code	awhv23c
Empirical formula	C_{209} $H_{161.50}$ Ag_{12} F_{48} I_4 $N_{54.50}$ O P_8
Formula weight	6414.22
Temperature	100(2) K
Wavelength	0.6889 Å
Crystal system	Tetragonal
Space group	I 41/a
Unit cell dimensions	a = 36.87840(10) Å
	b = 36.87840(10) Å
	c = 71.0220(2) Å
Volume	96591.1(6) Å^3
Z	16
Density (calculated)	1.764 Mg/m^3
Absorption coefficient	1.463 mm^{-1}
F(000)	50128
Crystal size	0.100 × 0.050 × 0.050 mm^3
Theta range for data collection	0.939 to 25.503°.
Index ranges	-44<=h<=46, -45<=k<=45, -88<=l<=86
Reflections collected	365374
Independent reflections	49286 [R(int) = 0.0454]
Completeness to theta = 24.415°	99.8 %
Absorption correction	Empirical
Max. and min. transmission	1.0 and 0.7128307640696863
Refinement method	Full-matrix least-squares on F^2
Data / restraints / parameters	49286 / 9444 / 3882
Goodness-of-fit on F^2	1.100
Final R indices [I>2sigma(I)]	R1 = 0.0590, wR2 = 0.1897
R indices (all data)	R1 = 0.0755, wR2 = 0.1981
Extinction coefficient	n/a
Largest diff. peak and hole	1.894 and -1.631 e.Å^{-3}

1-I contains three $Ag^{I}_{3}I$ clusters with an average Ag·Ag distance of 3.14 Å (Figure S165e, f, g), within the 3.44 Å sum of Van der Waals radii implying argentophilic interactions. The average, out of Van der Waals distance to the neighboring Ag ion is 4.79 Å. The other cluster environment of **1-I** (Figure S165d) contains an $Ag^{I}_{2}I$ cluster with an argentophilic Ag·Ag distance of 2.81 Å and average distances to the neighboring Ag ions of 3.82 Å and 4.65 Å.

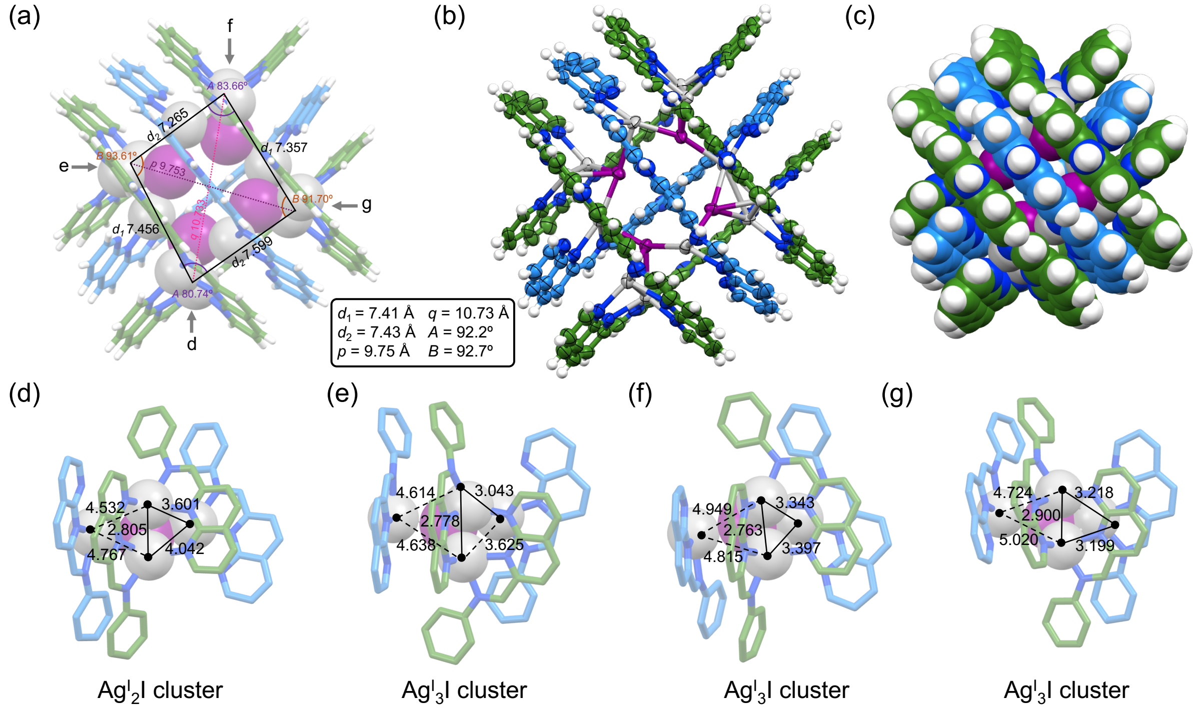

Figure S165. X-ray crystal structure of cationic component of **1-I** in (a) capped stick, (b) ellipsoids with 50% probability, and (c) spacefill representations. Disorder, anions and solvent molecules are omitted for clarity. (d) $Ag_{2}I$ cluster with intersilver dimensions labeled. (e, f, g) Intersilver dimensions in $Ag^{I}_{3}I$ clusters.

5.2 X-ray crystallography of grid **1-Cl**

Specific refinement details:

Crystals of **1-Cl** were grown by vapor diffusion of diethyl ether into acetonitrile solution of **1-Cl**, yielding red crystal blocks of **1-Cl** $[Ag_{12}Cl_4L_6](NTf_2)_8{\bullet}(CH_3CN)_4{\bullet}(H_2O)_{0.75}$. These crystals diffracted synchrotron radiation strongly and were collected on 1% transmission. The bis(trifluoromethanesulfonyl)imide anions within the structure show evidence of substantial disorder. Five of the eight of the anions were modeled as disordered over up to four locations. Some lower occupancy disordered sites were modeled with isotropic thermal parameters and thermal parameter restraints were applied to facilitate realistic modelling of all disordered anions. In order to obtain a geometrically reasonable model for the disordered anions similarity restraints (SAME) were used for higher occupancy sites and the anions that were modeled as disordered over only two parts while extensive DFIX and DANG restraints were used for the more highly disordered anions. Disordered acetonitrile molecules were modeled with similarity restraints (SAME). The occupancies of these solvent molecules were first refined and then fixed close to the obtained values. The hydrogen atoms of some low occupancy acetonitrile molecules and a partial occupancy water molecule could not be located in the electron density map and were not included in the model. Five reflections have been omitted from the final refinement. Four low angle reflections are likely affected by beam damage or absorption. A reflection (-14, 4, 0) at 1.18 Å resolution appears to be an artefact from the synchrotron data collection. CheckCIF gives a single B alert for residual density close to Ag8 likely arising from absorption effects, possibly beam damage during synchrotron data collection or minor unresolved disorder. This crystal structure is deposited in the CCDC with deposition number 2378429.

Table S8. Crystal data and structure refinement for 1-Cl_awhv85f2.

Identification code	**1-Cl**_cut07
Empirical formula	$C_{205.50}$ $H_{129.75}$ Ag_{12} Cl_4 F_{48} $N_{54.75}$ $O_{32.75}$ S_{16}
Formula weight	6751.08
Temperature	100(2) K
Wavelength	0.6889 Å
Crystal system	Triclinic
Space group	P -1
Unit cell dimensions	a = 17.26920(10) Å
	b = 20.33090(10) Å
	c = 35.8111(2) Å
Volume	11761.36(11) $Å^3$
Z	2
Density (calculated)	1.906 Mg/m^3
Absorption coefficient	1.186 mm^{-1}
F(000)	6656
Crystal size	0.100 × 0.100 × 0.050 mm^3
Theta range for data collection	0.589 to 27.741°.
Index ranges	-23<=h<=23, -27<=k<=27, -48<=l<=48
Reflections collected	186547
Independent reflections	60099 [R(int) = 0.0482]
Completeness to theta = 24.415°	99.2 %
Absorption correction	Empirical
Max. and min. transmission	1.0 and 0.8419538745664206
Refinement method	Full-matrix least-squares on F^2
Data / restraints / parameters	60099 / 3246 / 3860
Goodness-of-fit on F^2	1.015
Final R indices [I>2sigma(I)]	R1 = 0.0597, wR2 = 0.1751
R indices (all data)	R1 = 0.0744, wR2 = 0.1801
Extinction coefficient	n/a
Largest diff. peak and hole	1.841 and -1.886 e.$Å^{-3}$

The obtuse angles in **1-Cl** contain $Ag^{I}_{4}Cl$ clusters with average Ag·Ag distances of 2.81 Å and 3.33 Å (Figure S166d-e), within the 3.44 Å sum of Van der Waals radii implying argentophilic interactions. The acute angles of **1-Cl** contain $Ag^{I}_{2}Cl$ clusters with average Ag·Ag distances of 2.80 Å (Figure S166f-g), the average distance to the next closest Ag ion is 4.31 Å, longer than sum of Van der Waals radii.

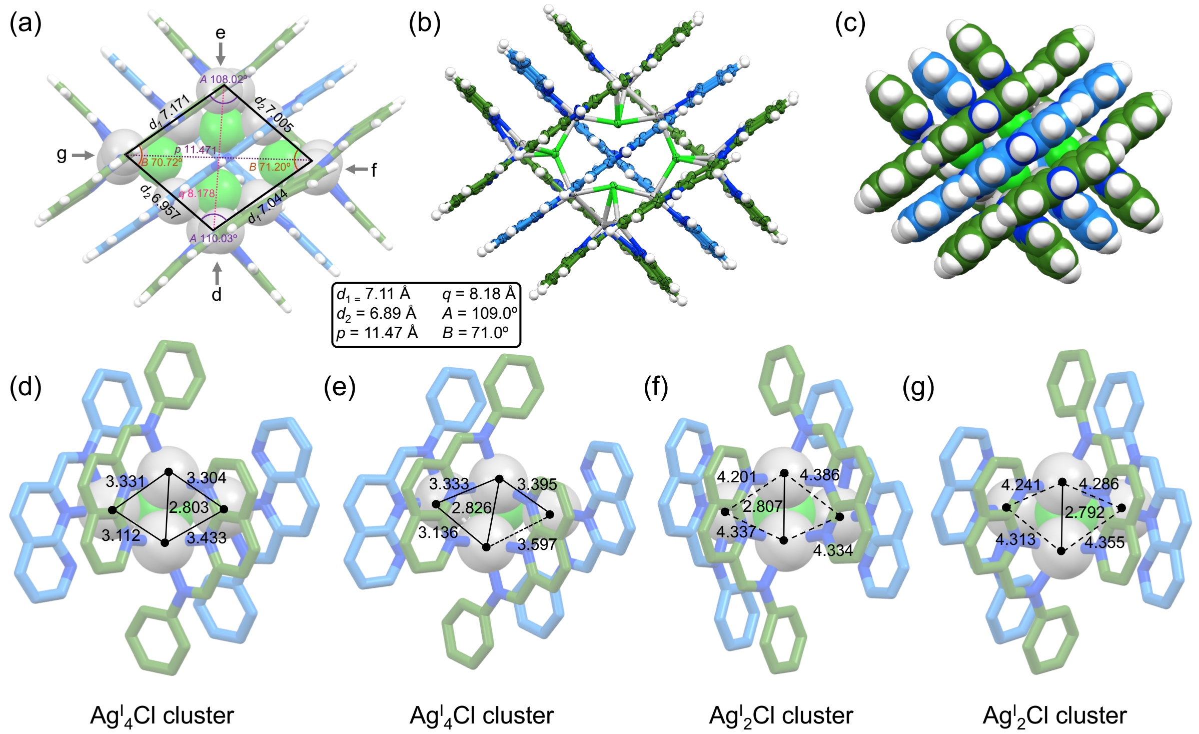

Figure S166. X-ray crystal structure of cationic component of **1-Cl** in (a) capped stick, (b) ellipsoids with 50% probability, and (c) spacefill representations. Disorder, anions and solvent molecules are omitted for clarity. (d, e) Intersilver dimensions in $Ag^{I}_{4}Cl$ clusters in the obtuse angle *A*. (f,g) Intersilver dimensions in $Ag^{I}_{2}Cl$ clusters in the acute angles *B*.

5.3 X-ray crystallography of grid **1-Br**

Specific refinement details:

Crystals of **1-Br** were grown by vapor diffusion of diisopropyl ether into an acetonitrile solution of **1-Br** with excess tetra-*n*-butylammonium tetrafluoroborate, yielding red crystal blocks of $[Ag_{12}Br_4L_6](BF_4)_8{\bullet}(CH_3CN)_{11.5}{\bullet}(H_2O)_{1.25}$. These crystals diffracted synchrotron radiation strongly and were collected on 1% transmission. Two of the naphthyridine-imine arms and their coordinated silver ions were modeled as disordered over two locations. The geometries of the two parts were restrained to be similar to each other using SAME and thermal parameter restraints were applied in order to refine the major occupancy part anisotropically. The tetrafluoroborate anions within the structure show evidence of substantial disorder. Six of the eight anions were modeled as disordered over up to three locations. Disordered tetrafluoroborate anions were restrained to be approximately tetrahedral with a single free variable refined for their average bond length. The acetonitrile solvent molecules were also extensively disordered. The occupancies of these solvent molecules were first refined and then fixed close to the obtained values when a lattice site was partially occupied or refined with free variables when the overall refined occupancy was close to one. In order to obtain a geometrically reasonable model for the disordered acetonitrile molecules similarity restraints (SAME) were used for higher occupancy sites and DFIX restraints were used for the lower occupancy sites. Some lower occupancy disordered anions and solvents were modeled with isotropic thermal parameters and thermal parameter restraints were applied to facilitate realistic modelling of all disordered species. The hydrogen atoms of some low occupancy acetonitrile molecules and partial occupancy water molecules could not be located in the electron density map and were not included in the model. Sixteen reflections, mostly at low angle, have been omitted from the final refinement. These reflections are likely affected by beam damage or absorption or possibly a small amount of twinning that could not be resolved during data processing. CheckCIF gives a single B alert for negative residual density close to Ag6 likely arising from absorption effects, beam damage during synchrotron data collection or minor unresolved disorder. This crystal structure is deposited in the CCDC with deposition number 2377075.

Table S9. Crystal data and structure refinement for 1-Br_awhv023b.

Identification code	awhv023b
Empirical formula	$C_{203}\ H_{151}\ Ag_{12}\ B_8\ Br_4\ F_{32}\ N_{53.50}\ O_{1.25}$
Formula weight	5668.32
Temperature	100(2) K
Wavelength	0.6889 Å
Crystal system	Triclinic
Space group	P -1
Unit cell dimensions	a = 19.29970(9) Å
	b = 20.38770(10) Å
	c = 27.70230(13) Å
Volume	10154.46(9) Å^3
Z	2
Density (calculated)	1.854 Mg/m^3
Absorption coefficient	1.860 mm^{-1}
F(000)	5571
Crystal size	0.050 × 0.050 × 0.010 mm^3
Theta range for data collection	0.752 to 29.477°.
Index ranges	-27<=h<=27, -29<=k<=29, -39<=l<=39
Reflections collected	178103
Independent reflections	61227 [R(int) = 0.0404]
Completeness to theta = 24.415°	99.3 %
Absorption correction	Empirical
Max. and min. transmission	1.0 and 0.7798325017426402
Refinement method	Full-matrix least-squares on F^2
Data / restraints / parameters	61227 / 2261 / 3243
Goodness-of-fit on F^2	0.960
Final R indices [I>2sigma(I)]	R1 = 0.0375, wR2 = 0.1028
R indices (all data)	R1 = 0.0473, wR2 = 0.1046
Extinction coefficient	n/a
Largest diff. peak and hole	0.997 and -1.385 e.Å^{-3}

The obtuse angles in **1-Br** contain one $Ag^{I}_{4}Br$ cluster with Ag·Ag distances of 2.78 Å and 3.30 Å (Figure S167d), within the 3.44 Å sum of Van der Waals radii implying argentophilic interactions. The other obtuse angle in **1-Br** contains an $Ag^{I}_{3}Br$ cluster with an average Ag·Ag distance of 2.97 Å, with average distance to the neighboring Ag ion at 3.93 Å (Figure S167e). The acute angles of **1-Br** contain $Ag^{I}_{2}Br$ clusters with average Ag·Ag distances of 2.79 Å (Figure S167f-g), the average distance to the next closest Ag ion is 4.46 Å, longer than sum of Van der Waals radii.

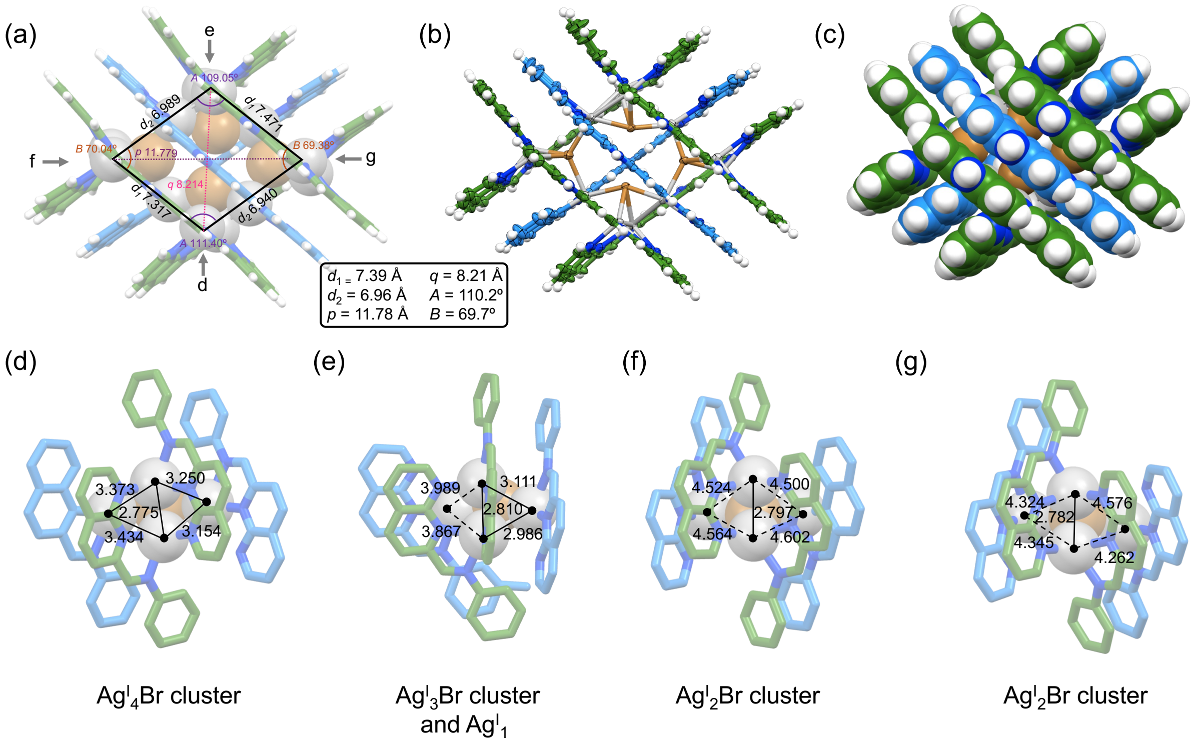

Figure S167. X-ray crystal structure of cationic component of **1-Br** in (a) capped stick, (b) ellipsoids with 50% probability, and (c) spacefill representations. Disorder, anions and solvent molecules are omitted for clarity. (d) Intersilver dimensions in $Ag^{I}_{4}Br$ cluster. (e) Intersilver dimensions in $Ag^{I}_{3}Br$ cluster. (f,g) Intersilver dimensions in $Ag^{I}_{2}Br$ clusters in the acute angles *B*.

5.4 Analysis of crystal structures **1-Cl**, **1-Br** and **1-I**

Rhombic dimensions and inter-silver dimensions are measured from the crystal structures, between the centroids of the Ag_2 clusters at the vertices of the rhomboid. Dimension d_1 refers to the average distance between the centroids of Ag_2 clusters between the longer edges of the rhomboid. Dimension d_2 refers to the average distance between the centroids of Ag_2 clusters between the shorter edges of the rhomboid. Dimensions p (bisecting the acute angles) and q (bisecting the obtuse angles) refer to the longer and shorter diagonal lengths respectively. Angles A and B refer to the average obtuse and average acute angles respectively.

The larger iodide anions favor Ag^{I}_3 clusters, causing the grid to rearrange with angles A and B changing from approximately 109º and 71º to closer to 90º. The C_{aryl}-N_{imine} bond rotation mediates the formation of an Ag^{I}_3 cluster, moving the edge Ag ion closer to the vertex cluster environment.

1-Cl has two Ag^{I}_4Cl environments in the obtuse angles, and two Ag^{I}_2Cl environments in the acute angles. **1-Br** has similar Ag^{I} environments to **1-Cl**, however one of the obtuse angles has an extended Ag·Ag distance, resulting in an Ag^{I}_3Br cluster. **1-I** has three similar environments with Ag^{I}_3I clusters, however one environment has elongated Ag·Ag distances resulting in one formal Ag^{I}_2I environment.

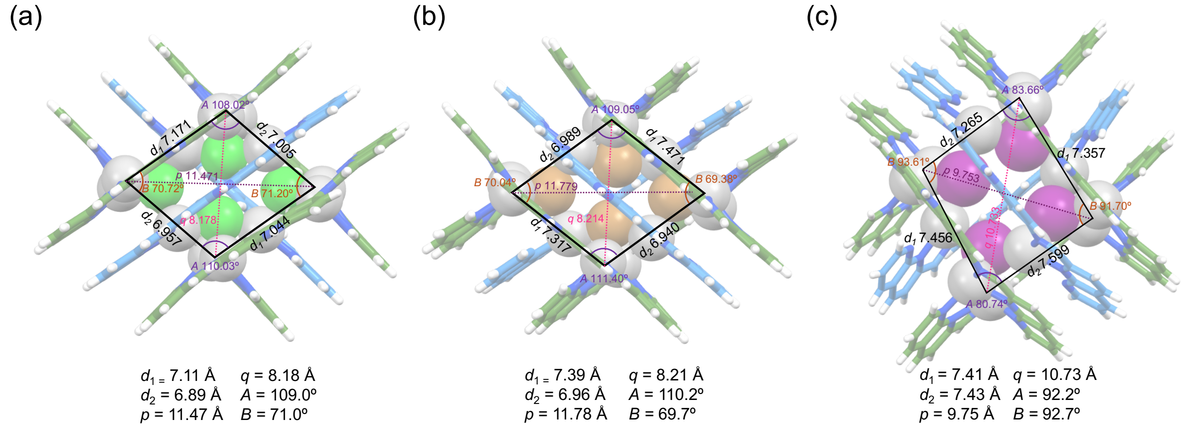

Figure S168. Rhomboid dimensions in (a) **1-Cl**, (b) **1-Br**, and (c) **1-I**.

Table S10. Summary of distances and angles from crystal structures of **1-Cl**, **1-Br** and **1-I**.

	**1-Cl**	**1-Br**	**1-I**
d_1 / Å	7.11	7.31	7.41
d_2 / Å	6.89	6.96	7.43
p / Å	11.47	11.78	9.75
q / Å	8.18	8.21	10.73
A	109.0º	110.2º	92.2º
B	71.0º	69.7º	92.7º

6. Halide Exchange Experiments of 1

In previous publications we have demonstrated complete exchange of halides on addition of a softer halide, and no exchange of halides upon addition of harder halides.[12] Initially it was hypothesized that the softer halides would stabilize the Ag^{I} clusters more. However, whilst the isotope pattern in the high resolution mass spectrum of **1-Cl** showed both the $[Ag_{12}Cl_4L_6](NTf_2)_4^{4+}$ parent ion and the $[Ag_6Cl_2L_3](NTf_2)_2^{2+}$ fragment ion, the isotope patterns of **1-Br** and **1-I** showed only the fragment ion, $[Ag_6X_2L_3](NTf_2)_2^{2+}$, and none of the parent ion, $[Ag_{12}X_4L_6](NTf_2)_4^{4+}$. The differing fragmentation efficiencies implied that steric strain from larger halides may destabilize the $[Ag_{12}X_4L_6](NTf_2)_8$ structure, in opposition to the initial hypothesis based on favorable soft-soft interactions. To further probe the relative stabilities of each Ag^{I} cluster in this structure, we performed halide exchange experiments.

Aliquots of tetra-*n*-butyl ammonium halide ($^{n}Bu_4NX$) in CD_3CN (30 mM) were added to $[Ag_{12}X_4L_6](NTf_2)_8$ (**1-Cl**, 1.4 µmol; **1-Br**, 2.0 µmol; **1-I**, 1.6 µmol) in CD_3CN (0.5 mL) with an internal standard (5 µL of 0.72 M 1,2-dichloroethane in CD_3CN) with conversion monitored by ^1H NMR. The decreasing concentration and precipitation induced by anion exchange caused broadening of the ^1H NMR.

The titrations confirmed that this structure follows the previously observed preference for softer halides exchanging the harder halides. **1-Cl** showed halide exchange with Br^- (Figure S170) and I^- (Figure S171). **1-Br** showed halide exchange with softer I^- (Figure S173) however, no conversion was observed on addition of harder Cl^- (Figure S172). **1-I** showed no exchange with harder Cl^- (Figure S174) or Br^- (Figure S175).

Upon addition of 0.5-2.0 equivalents of a softer halide to **1-Cl** or **1-Br** (Figure S170, Figure S171, Figure S173), intermediate species are observed which converge upon addition of 4.0 equivalents.

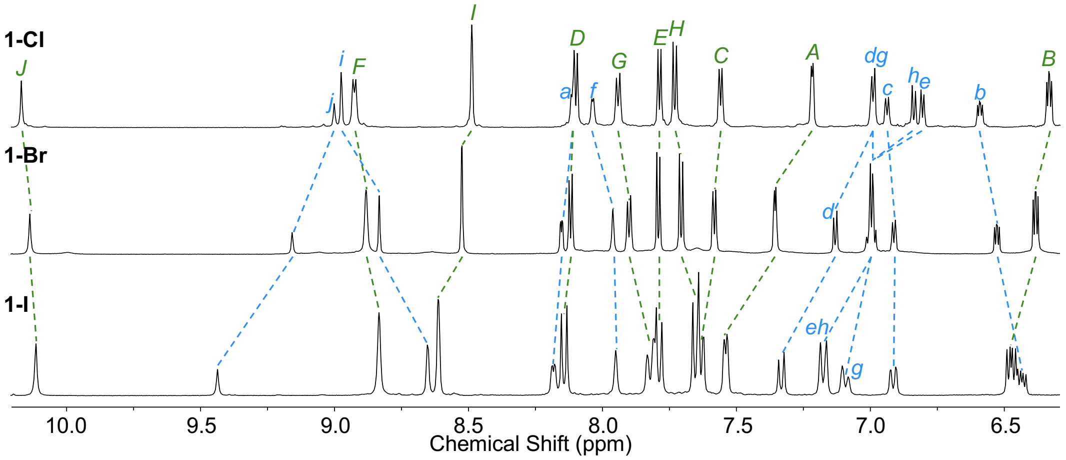

Figure S169. ^1H NMR stack plot showing **1-Cl** template (top), **1-Br** template (middle) and **1-I** template (bottom) (CD_3CN, 500 MHz, 298 K).

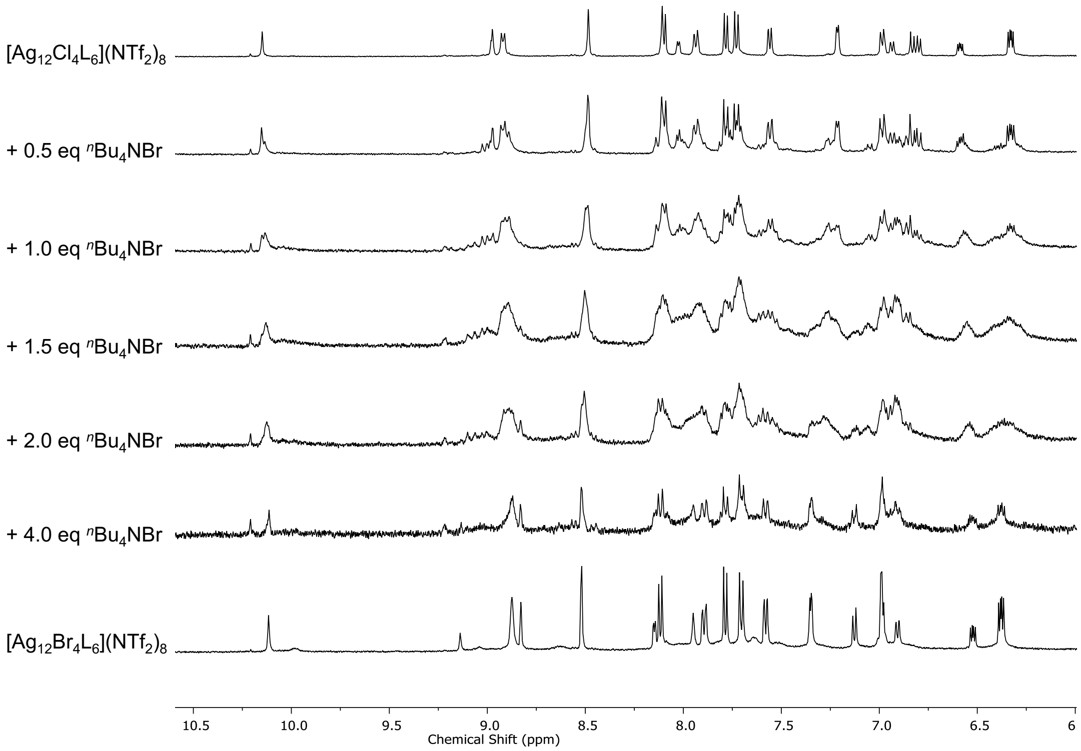

Figure S170. Titration of $^{n}Bu_4NBr$ into **1-Cl** (CD_3CN, 400 MHz, 298 K).

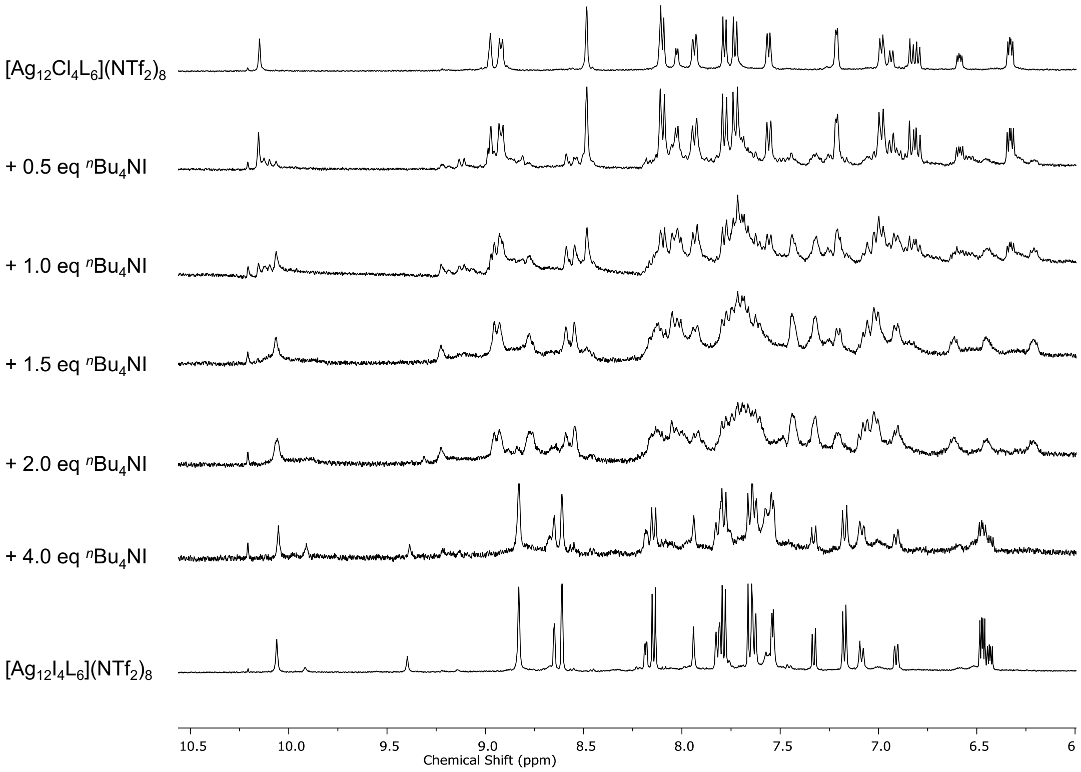

Figure S171. Titration of $^{n}Bu_4NI$ into **1-Cl** (CD_3CN, 400 MHz, 298 K).

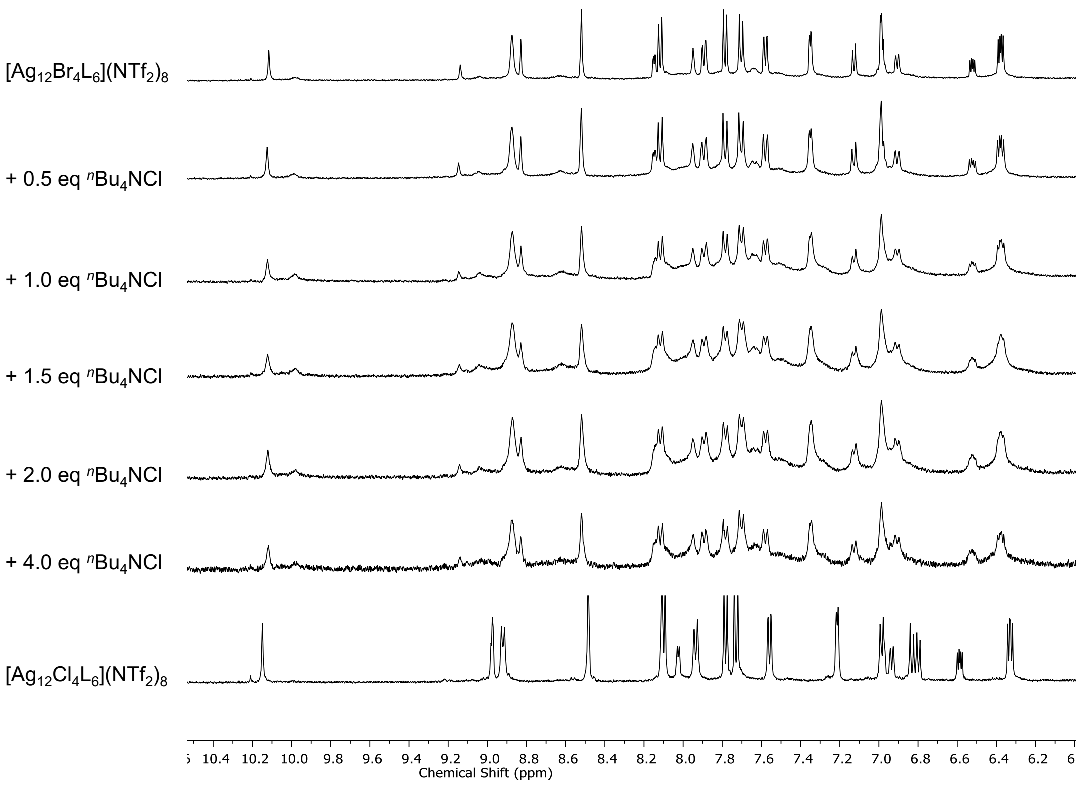

Figure S172. Titration of ^{n}Bu$_4$NCl into **1-Br** (CD_3CN, 400 MHz, 298 K).

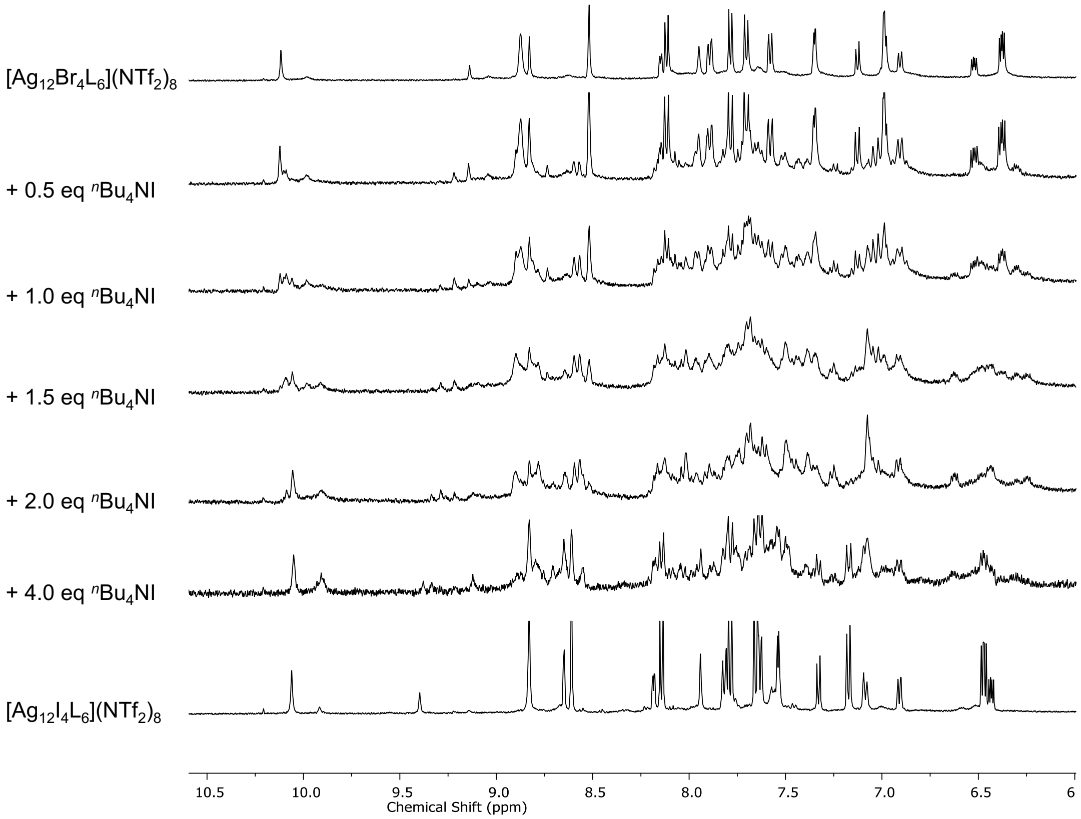

Figure S173. Titration of ^{n}Bu$_4$NI into **1-Br** (CD_3CN, 400 MHz, 298 K).

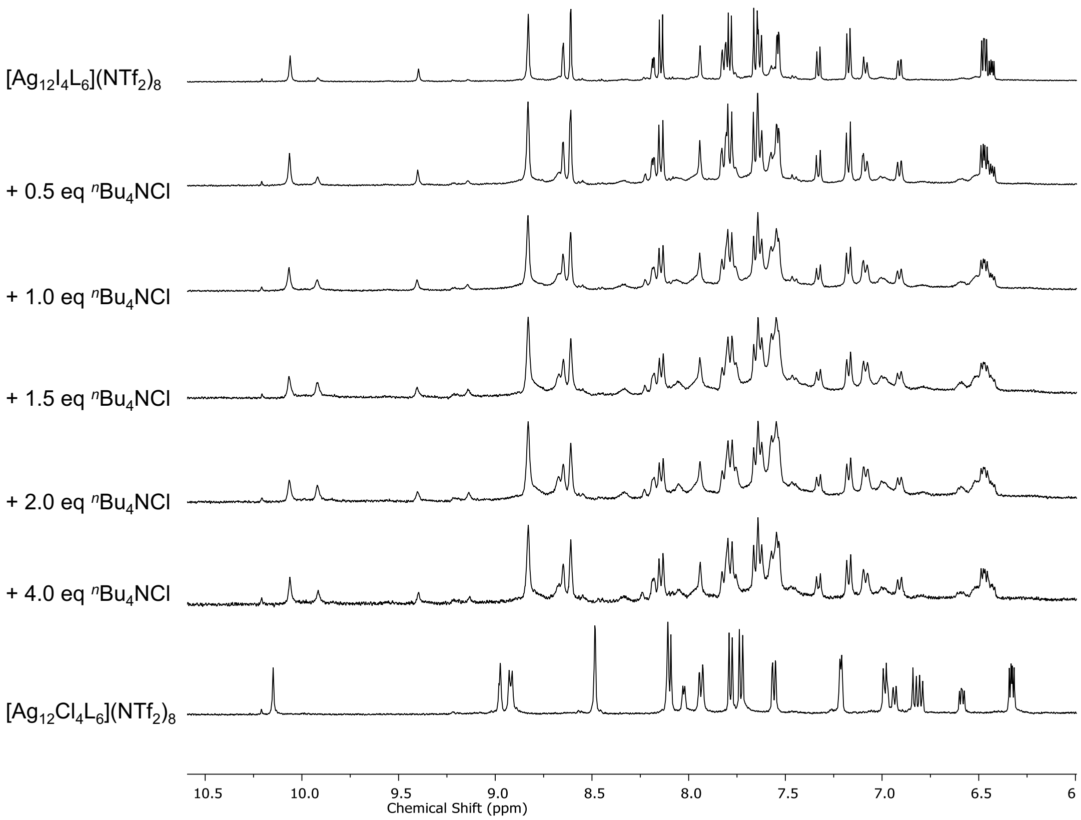

Figure S174. Titration of ^{n}Bu$_4$NCl into **1-I** (CD_3CN, 400 MHz, 298 K).

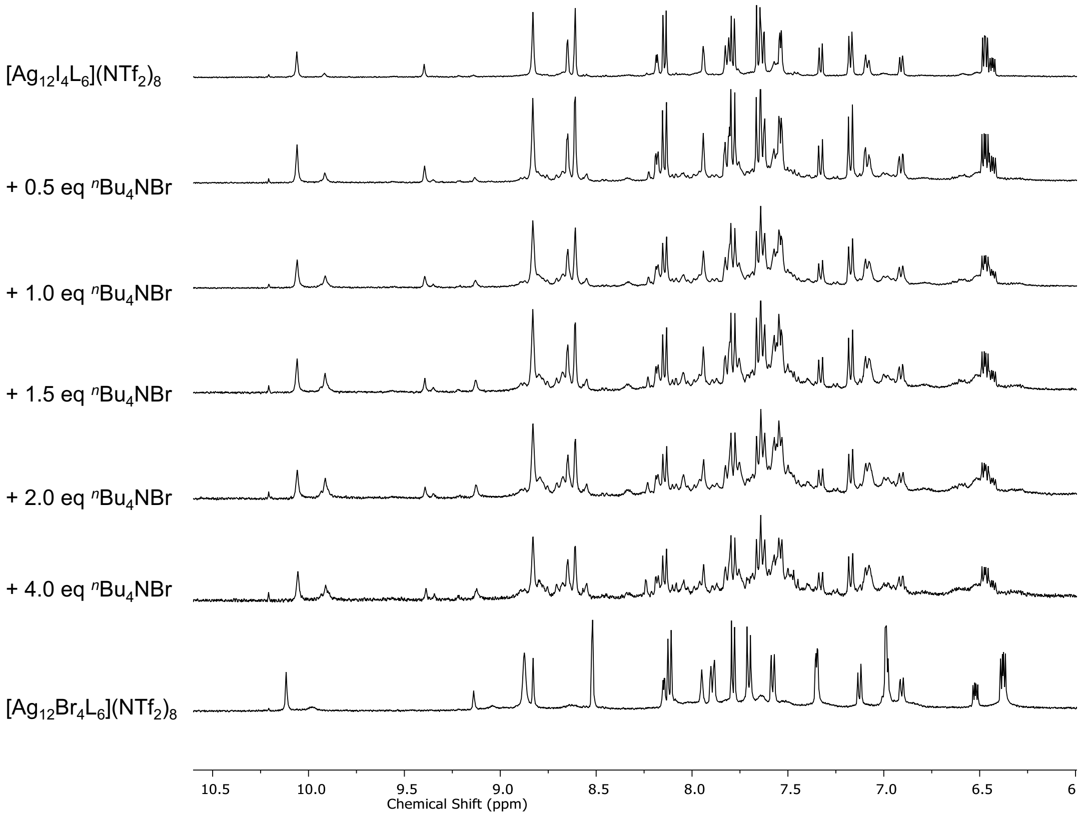

Figure S175. Titration of ^{n}Bu$_4$NBr into **1-I** (CD_3CN, 400 MHz, 298 K).

7. Raman analysis of grids 1-Cl, 1-Br, and 1-I

7.1 Thin-flim Raman Spectroscopy

Visible-light Raman spectra were recorded using an Invia Renishaw spectrometer (50) equipped with 785 nm lasers. Compounds **1-Cl**, **1-Br** and **1-I** were dissolved in acetonitrile and two drops were deposited on a quartz slide and left to dry at room temperature, sub-component **B** was put on the slide as powder. Spectra were acquired on at least five areas of the sample and representative spectra are shown.

Force constants and bond lengths of Ag·Ag interactions were determined from the Herschbach-Laurie equations (equations 1 and 2).[13]

(1) $$F_{Ag_2} = \mu\left[2\pi c v_{Ag_2}\right]^2$$

(2) $$r_{Ag_2} = -0.284 \cdot \ln\left(F_{Ag_2}\right) + 2.53$$

Where F_{Ag2} is the force constant, mdyn·$Å^{-1}$; μ is reduced mass, kg; *c* is the speed of light, ms^{-1}; ν_{Ag2} is the stretching wavenumber, cm^{-1}; r_{Ag2} is the bond length, Å.

Solid state Raman spectroscopy of thin films of **1-Cl**, **1-Br** and **1-I** was used to further investigate the Ag^{I}_{n} cluster environments. The region 1100-1650 cm^{-1} (Figure 176) showed several sharp signals corresponding to surface-enhanced Raman scattering (SERS) of aromatic nuclei and Ag atoms.[14] A diagnostic signal at 1372 cm^{-1} corresponded to a symmetric 1,8-naphthyridyl vibrational mode.[15] The region of 1500-1600 cm^{-1} corresponds to stretching frequencies of the C=N bonds of metal-coordinated imines,[16] shifted from the typical C=N region of 1630-1650 cm^{-1}.[17] Sub-component **B** showed no bands in the imine region, instead showing a strong peak attributed to C=O stretching frequency at 1702 cm^{-1}.[18] This C=O signal was absent in the spectra of the grids **1-Cl**, **1-Br** and **1-I** confirming the absence of imine-hydrolysis and the stability of the assemblies under thin film Raman conditions.

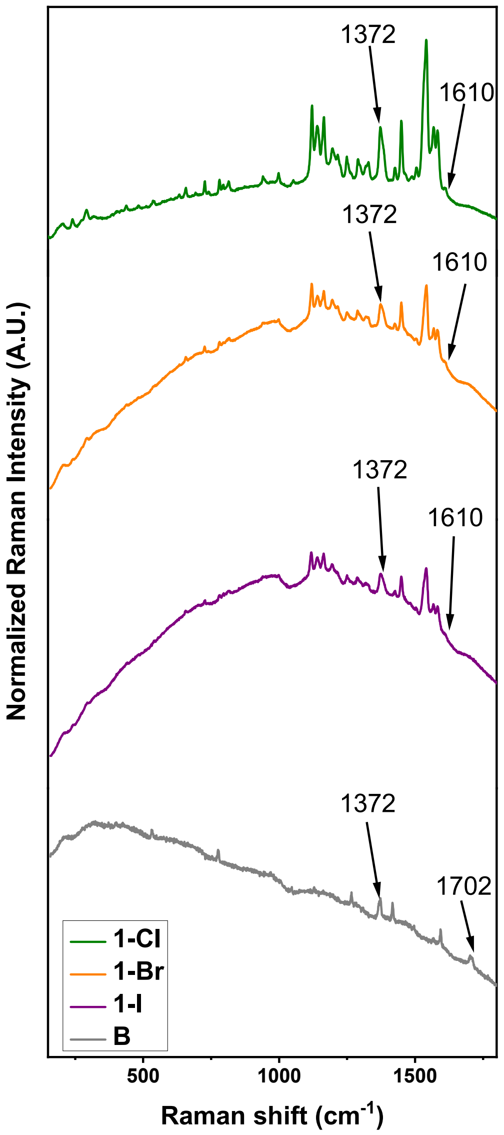

Figure S176. Raman spectra for **1-Cl**, **1-Br** and **1-I**, with **B** as a reference to confirm the absence of cage hydrolysis, acquired on a thin-film sample irradiating at 785 nm. Average of 20 accumulations.

Structures **1-Cl**, **1-Br** and **1-I** all had thin-film Raman bands at 240-242 cm^{-1} (Figure S177), corresponding to the Ag–N bending frequency.[19] The Ag·Ag stretching region shows multiple stretching frequencies for **1-Cl**, matching Ag·Ag interactions of different strengths and interatomic separations. **1-Cl**, **1-Br** and **1-I** have signals at 205-207, 321-323, 437-429 cm^{-1} corresponding to the Ag·Ag stretching overtone ($\Delta\nu$ = 116 cm^{-1}) with an interatomic distance of 2.8 Å (F = 0.43 mdyn·$Å^{-1}$), similar to the shortest Ag·Ag distances measured from the X-ray crystal structures of **1-Cl** (section 5.2), **1-Br** (section 5.3) and **1-I** (section 5.1).

In structure **1-Cl**, additional Ag·Ag signals (482, 536, 590 cm^{-1}) occur in progression at the recurrent distance of 54 cm^{-1}, which could be compatible with stretching overtones of a weaker Ag·Ag interaction at interatomic distance of 3.2 Å (F = 0.094 mdyn·$Å^{-1}$), similar to the average distance of 3.34 Å measured from the X-ray crystal structure of **1-Cl** (section 5.2).

These thin film Raman spectra corroborate the observed Ag^{I}_2 and Ag^{I}_4 clusters in solid state for **1-Cl**. Structures **1-Br** and **1-I** show fewer, broader Ag·Ag stretching frequencies corresponding to more similar Ag·Ag distances observed in the Ag^{I}_3 clusters, further matching solid state observations from the X-ray crystal structures. We inferred that in the case of soft halides, such as Br^- and I^-, the stronger Ag-X bonding results in the preservation of only the strongest Ag-Ag bonding

in the film state. Conversely, the weaker interaction between Ag and hard Cl^- enables the preservation of weaker Ag-Ag interactions that are found in the XRD structure of **1-Br** and **1-I**.

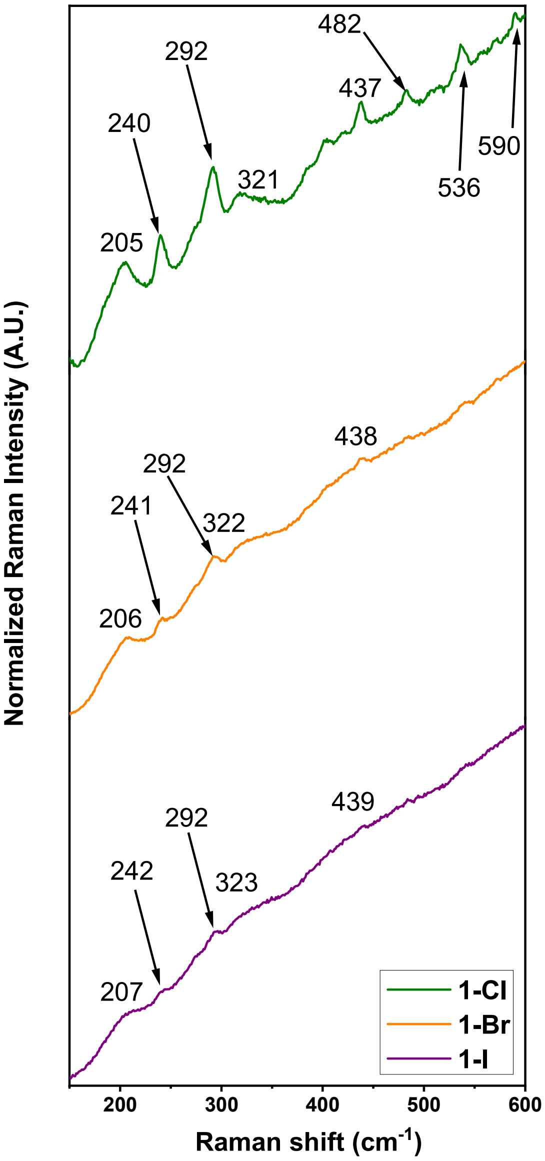

Figure S177. Raman spectra for grids in the range between 150-600 cm^{-1}, acquired on a thin-film sample irradiating at 785 nm. Average of 20 accumulations. Ag-N bending at 240-242 and 292 cm^{-1}; Ag···Ag overtones at 205-207, 321-323 and 437-439 cm^{-1}; additional Ag···Ag overtones at 482, 536, 590 cm^{-1} for **1-Cl**.

7.2 UV Resonance Raman Spectroscopy

UV Resonance Raman (UVRR) spectroscopy was used to assess the integrity of **1-Cl**, **1-Br**, and **1-I** in the solution state. Excitation at 266 nm enabled the amplification of aromatic signals in the fingerprint region between 1500-1700 cm^{-1} and suppression of those arising from other functional groups.

The Raman signals associated to the solvent are clearly distinguishable in the UVRR spectra of **A**, **B**, **1-Cl**, **1-Br** and **1-I** and were easily subtracted from the spectra. Moreover, using this excitation wavelength, the interfering fluorescence background superimposed to the Raman signal in the visible spectra can be completely suppressed.

The Raman signals of aromatic moieties dominate the UVRR spectra around 1500-1600 cm^{-1}, the stretching of the aldehyde group of **A** at 1720 cm^{-1} was not present in the spectra of **1-Cl**, **1-Br**, and **1-I**, confirming the retention of the imine C=N bond. The aromatic region displayed the rising of complex signature that was not simply the sum of the aromatic contribution of the sub-components **A** and **B**. This is evident in the broad distribution between about 1545 and 1652 cm^{-1} in the UVRR spectra of the grid, both for **1-Cl** and **1-Br**. We inferred that these new spectral features are the result of interactions between the aromatic panels in the grid, in agreement with the π-π stacking observed by single-crystal XRD.

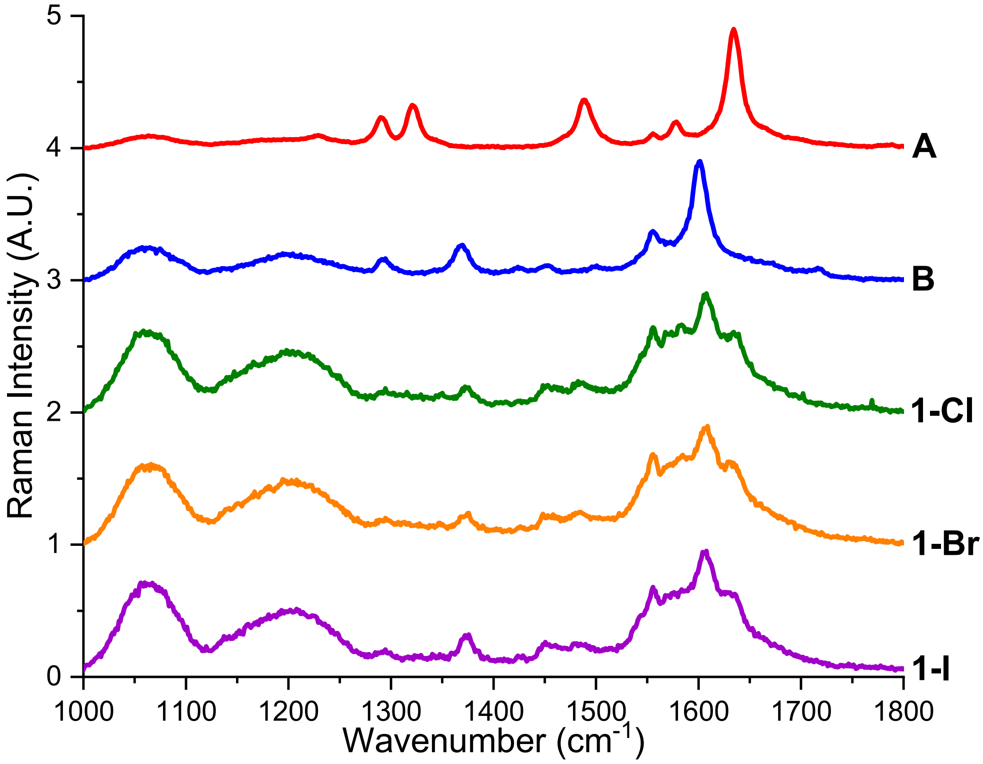

Figure S178. UV resonance-raman spectra of **A** (red), **B** (blue), **1-Cl** (green), **1-Br** (orange), and **1-I** (purple).

8. Computational dynamics

8.1 Quantum Mechanics/Molecular Mechanics Molecular Dynamics Simulations

The QM/MM molecular dynamics simulations were performed using CP2K[20] to explore the short-timescale dynamics of the **1-I** system. For simplicity, the compound was modeled as $[Ag^{I}_{12}I_4L_6](NO_3)_8$ due to the reduced complexity of nitrate anions compared to bis(trifluoromethanesulfonyl)imide. The simulation system was divided into a QM region, consisting of the metal-organic complex (including Ag and I ions and surrounding organic components), and an MM region, which modeled the solvent (acetonitrile and nitrate ions). The QM region was treated with density functional theory (DFT), while the MM region was represented using a classical force field derived from the AMBER parameter set.[21]

The QM region calculations employed the GPW (Gaussian and Plane Waves) method with the hybrid PBE0 functional.[22] To account for dispersion interactions, the DFT-D3 correction with BJ damping was applied.[23] Basis sets were carefully chosen to balance accuracy and computational cost, with TZV2P-MOLOPT-GTH basis sets used for all atoms in the QM region and GTH pseudopotentials were applied. A truncated Coulomb potential with a cutoff radius of 6 Å was used for Hartree-Fock exchange, and the Poisson equation was solved under periodic boundary conditions in all three dimensions (XYZ). To ensure high accuracy in the electronic density calculations, the grid cutoff was set to 900 Ry, with a relative cutoff of 100 Ry.

The MM region was modeled using standard AMBER force field model.[24] Non-bonded interactions in the MM region were calculated using the Ewald summation method with a smooth particle-mesh Ewald (SPME) scheme,[25] an alpha parameter of 0.35, and a cutoff radius of 10 Å for real-space interactions. The coupling between the QM and MM regions was handled using a Gaussian embedding scheme, where the QM region's electrostatic potential influenced the MM region's dynamics. The QM region was dynamically centered within the simulation box at every MD step to ensure proper boundary conditions, with polarization effects accounted for using atomic radii provided for boundary atoms.

The molecular dynamics simulations were conducted in the NVT ensemble with temperature controlled using a Canonical Sampling through Velocity Rescaling (CSVR) thermostat.[26] The target temperature was set to 330 K with a thermostat time constant of 10 fs to allow an accelerated exploration of the conformational space accessible at lower temperatures. The timestep for the integration was 0.5 fs, and the simulation was run for a total of 8,000 steps, corresponding to 4 ps of simulation time.

The auxiliary density matrix method (ADMM),[27] reducing the computational overhead of hybrid functional calculations by approximating the exact exchange term. This setup enabled a detailed examination of the interactions within **1-I** and its interaction with the solvent, providing insights into the structural and electronic dynamics on the picosecond timescale.

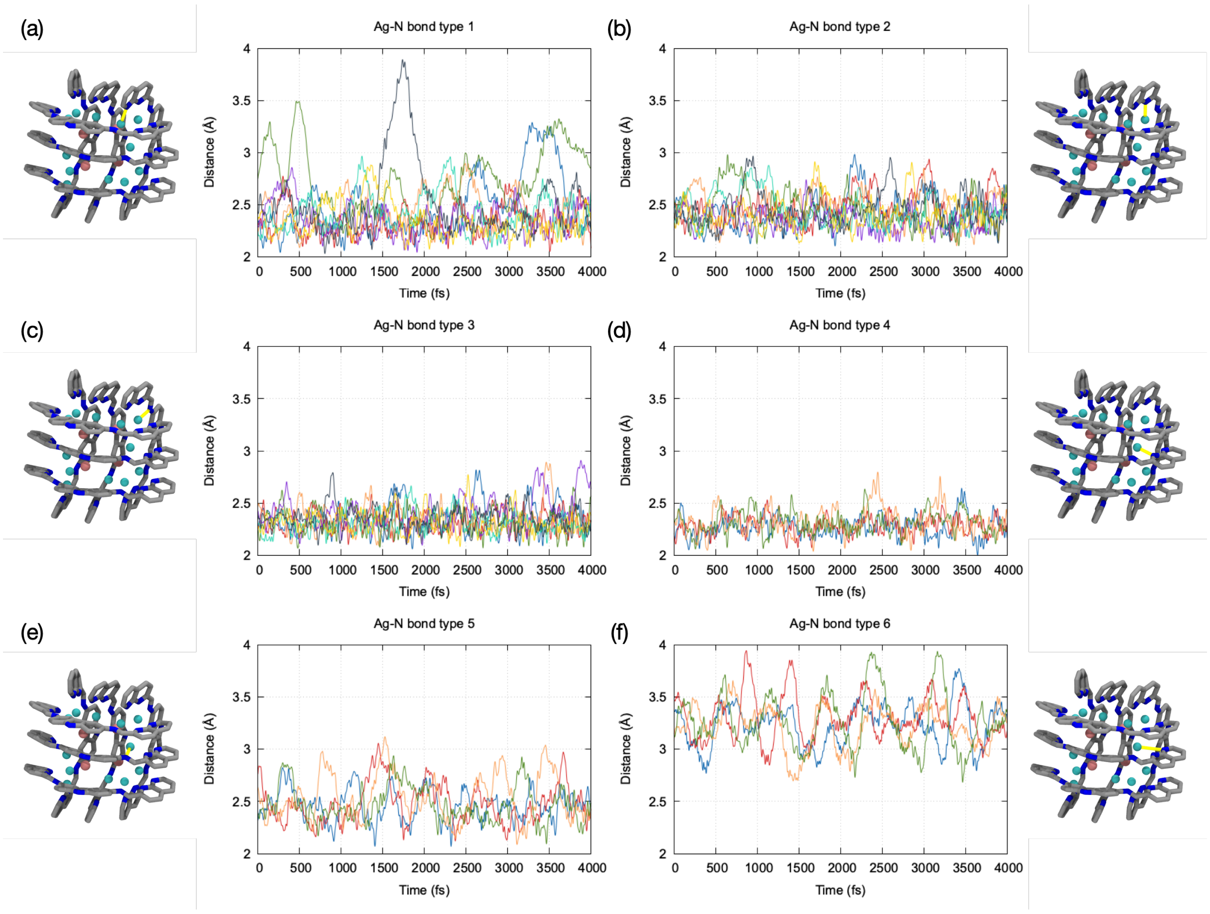

Figure S179. Time evolution of the N-Ag distance. The time evolution of the N–Ag bond distance is plotted for each topologically similar bond (bond type). For each bond type (1–6), an example is highlighted in yellow in the neighboring cartoon representation. Panels (a–c) represent interactions between Ag ions and external organic molecules, with each bond type comprising 8 equivalent distances. Panels (d–f) represent interactions between Ag^{I} ions and the central organic components, with each bond type comprising 4 equivalent distances. Though stable, large fluctuations can be observed for bond type 1 (a). Bond type 6 (f) competes with bond type 5 (e) but is weaker, displaying significant fluctuations. This, however, stabilizes bond type 4 (d), which shows less variability. These observations highlight the dynamic interplay between different bond types and their roles in stabilizing the overall coordination environment. See Figure 3 for the distribution of each bond types.

8.2 Classical Molecular Dynamics Simulations

To investigate the structural dynamics of **1-I** on 1 μs timescales, classical molecular dynamics (MD) simulations were performed using GROMACS.[28]

Gaussian 16[29] along with Antchamber[30] softwares were emplyed for the parametrization of the atomic charges. The geometry was first optimized using PM7 starting from the crystal structure.[31] The electrostatic potential (ESP) map was estimated from this structure at the B3LYP level of theory with the 6-31G(d) basis set for light atoms (C, H, N) and the DGDZVP basis set for heavy atoms (Ag, I),[32] within the Merz-Kollman (MK).[33] The resulting ESP data was used to fit atomic charges via the RESP method using antchamber,[30] capturing the electronic environment of the complex for use in classical molecular dynamics simulations.

The organic components were parametrized using the General Amber Force Field (GAFF),[34] which provides reliable parameters for small organic molecules. To model the N–Ag–I interactions, bond and angles were tuned following Seminario's method, applied to the PM7 Hessian calculation. Additional tests showed that modifying the parametrization procedure for these interactions within the 3×3 grid (e.g., Boltzmann inversion with respect to QM/MM MD) did not alter the overall picture provided by the models. This indicates that, as long as metal ion dynamics can be observed within the time scales accessible by MD simulations, they remain consistently associated with and coupled to the dynamics of both the grid and its inner cavity. The advantage of this approach lies in the reduced interatomic strain provided by the parametrization of Van der Waals interactions within GAFF.

The acetonitrile solvent, was parameterized according to what described in the literature,[24] which provides parameters optimized for simulating acetonitrile's dynamics and interactions with solutes. The simulated system consisted of the **1-I** complex surrounded by 1235 acetonitrile molecules in a periodic cubic box of 4.9 nm long side. Eight nitrite molecules were used as counterions.

The simulation system was equilibrated through a multi-step process, starting with energy minimization to relax any steric clashes in the initial configuration. This was followed by a short simulation to stabilize the system under constant temperature and pressure conditions. Temperature coupling was achieved using the v-rescale thermostat, maintaining a stable temperature of 300 K.[26] The pressure was controlled using the C-rescale barostat,[35] which applied isotropic pressure coupling to maintain the system at 1 atm. These equilibration steps ensured that the system reached a stable and thermodynamically relevant configuration before initiating the production run.

The production MD simulation was conducted for 1 μs with a timestep of 2 fs, allowing for a detailed observation of the system's dynamics. Long-range electrostatic interactions were treated using the particle-mesh Ewald (PME) method.[25] A cutoff of 1.0 nm was applied to both Van der Waals and Coulomb interactions. To maintain the integrity of hydrogen bonds, bond constraints were applied using the LINCS algorithm, with a high order to ensure robust treatment of bonded interactions.[36]

Throughout the simulation, the metal-organic complex remained structurally stable, with dynamic rocking and torsional motions observed in the organic framework. As observed during the dynamics (Supporting Movie 3) and as indicated by the principal component analysis (PCA),[37] the second and third modes (Suppplementary Movie 4-5 respectively) of the structure are related to the displacements of the central ligands.

The results of these simulations provide critical insights into the interplay between silver ions and the organic framework, highlighting the role of torsional flexibility and collective motions in maintaining structural integrity and enabling potential migration pathways.

9. References

(1) Farrow, N. A.; Zhang, O.; Forman-Kay, J. D.; Kay, L. E. A heteronuclear correlation experiment for simultaneous determination of ^{15}N longitudinal decay and chemical exchange rates of systems in slow equilibrium. *J. Biomol. NMR* **1994**, *4* (5), 727-734. DOI: 10.1007/BF00404280
(2) Allan, D.; Nowell, H.; Barnett, S.; Warren, M.; Wilcox, A.; Christensen, J.; Saunders, L.; Peach, A.; Hooper, M.; Zaja, L.; et al. A Novel Dual Air-Bearing Fixed-χ Diffractometer for Small-Molecule Single-Crystal X-ray Diffraction on Beamline I19 at Diamond Light Source. *Crystals* **2017**, *7* (11). DOI: 10.3390/cryst7110336
(3) Winter, G.; Waterman, D. G.; Parkhurst, J. M.; Brewster, A. S.; Gildea, R. J.; Gerstel, M.; Fuentes-Montero, L.; Vollmar, M.; Michels-Clark, T.; Young, I. D.; et al. DIALS: implementation and evaluation of a new integration package. *Acta Cryst. D* **2018**, *74* (Pt 2), 85-97. DOI: 10.1107/S2059798317017235 . Winter, G. xia2: an expert system for macromolecular crystallography data reduction. *J. Appl. Cryst.* **2009**, *43* (1), 186-190. DOI: 10.1107/s0021889809045701 . Evans, P. Scaling and assessment of data quality. *Acta Cryst. D* **2006**, *62* (Pt 1), 72-82. DOI: 10.1107/S0907444905036693
(4) Evans, P. R.; Murshudov, G. N. How good are my data and what is the resolution? *Acta Cryst. D* **2013**, *69* (Pt 7), 1204-1214. DOI: 10.1107/S0907444913000061
(5) Winn, M. D.; Ballard, C. C.; Cowtan, K. D.; Dodson, E. J.; Emsley, P.; Evans, P. R.; Keegan, R. M.; Krissinel, E. B.; Leslie, A. G.; McCoy, A.; et al. Overview of the CCP4 suite and current developments. *Acta Cryst. D* **2011**, *67* (Pt 4), 235-242. DOI: 10.1107/S0907444910045749
(6) Sheldrick, G. M. SHELXT - integrated space-group and crystal-structure determination. *Acta Cryst. A* **2015**, *71* (Pt 1), 3-8. DOI: 10.1107/S2053273314026370
(7) Sheldrick, G. M. Crystal structure refinement with SHELXL. *Acta Cryst. C* **2015**, *71* (Pt 1), 3-8. DOI: 10.1107/S2053229614024218
(8) Zhao, J.; Peng, L.; Zhu, Y.-L.; Song, Y.-J.; Wang, L.-J.; Shen, Y.-Z. Synthesis and memory characteristics of novel soluble polyimides based on asymmetrical diamines containing carbazole. *Polymer* **2016**, *91*, 118-127. DOI: 10.1016/j.polymer.2016.03.067
(9) Mousavi, B.; Chauvin, A. S.; Moriggi, L.; Helm, L. Carbazole as Linker for Dinuclear Gadolinium-Based MRI Contrast Agents. *Eur. J. Inorg. Chem.* **2017**, *2017* (45), 5403-5412. DOI: 10.1002/ejic.201700847
(10) Liu, G.; Liu, C.; Han, F.; Wang, Z.; Wang, J. Highly active palladium catalysts containing a 1,10-phenanthroline analogue N-heterocyclic carbene for room temperature Suzuki-Miyaura coupling reactions of aryl chlorides with arylboronic acids in aqueous media. *Tet. Lett.* **2017**, *58* (8), 726-731. DOI: 10.1016/j.tetlet.2016.12.071
(11) Thiele, C. M.; Petzold, K.; Schleucher, J. EASY ROESY: reliable cross-peak integration in adiabatic symmetrized ROESY. *Chem. Eur. J.* **2009**, *15* (3), 585-588. DOI: 10.1002/chem.200802027 . Schleucher, J.; Quant, J.; Glaser, S. J.; Griesinger, C. A Theorem Relating Cross-Relaxation and Hartmann-Hahn Transfer in Multiple-Pulse Sequences. Optimal Suppression of TOCSY Transfer in ROESY. *J. Magn. Reson. A* **1995**, *112* (2), 144-151. DOI: 10.1006/jmra.1995.1025
(12) Clark, S. E.; Heard, A. W.; McTernan, C. T.; Ronson, T. K.; Rossi, B.; Rozhin, P.; Marchesan, S.; Nitschke, J. R. A Double-Walled Tetrahedron with Ag^{I}_{4} Vertices Binds Different Guests in Distinct Sites. *Angew. Chem. Int. Ed.* **2023**, *62* (16), e202301612. DOI: 10.1002/anie.202301612
(13) Harvey, P. Reparameterized Herschbach-Laurie empirical relationships between metal-metal distances and force constants applied to homonuclear bi- and polynuclear complexes (M = Cr, Mo, Rh, Pd, Ag, W, Re, Ir, Pt, Au, Hg). *Coord. Chem. Rev.* **1996**, *153*, 175-198. DOI: 10.1016/0010-8545(95)01225-7 . Herschbach, D. R.; Laurie, V. W. Anharmonic Potential Constants and Their Dependence upon Bond Length. *J. Chem. Phys.* **1961**, *35* (2), 458-464. DOI: 10.1063/1.1731952
(14) Chen, L.; Gao, Y.; Xu, H.; Wang, Z.; Li, Z.; Zhang, R. Q. The mechanism of N-Ag bonding determined tunability of surface-enhanced Raman scattering of pyridine on MAg (M = Cu, Ag, Au) diatomic clusters. *Phys. Chem. Chem. Phys.* **2014**, *16* (38), 20665-20671. DOI: 10.1039/c4cp03205e
(15) Carrano, J. T.; Wait, S. C. Vibrational spectrum and assignments for 1,6- and 1,8-naphthyridine. *J. Mol. Spect.* **1973**, *46* (3), 401-418. DOI: 10.1016/0022-2852(73)90053-2
(16) Sánchez-González, R.; Imbarack, E.; Suazo, C.; Soto, J. P.; Leyton, P.; Sánchez-Cortés, S.; Campos-Vallette, M. Synthesis, characterization and surface enhanced Raman spectroscopy

study of a new family of different substituted cruciform molecular systems deposited on gold nanoparticles. *J. Raman Spect.* **2021**, *52* (5), 959-970. DOI: 10.1002/jrs.6082
(17) Socrates, G. *Infrared and Raman Characteristic Group Frequencies: Tables and Charts*; John Wiley & Sons, 2004.
(18) Visser, T.; van der Maas, J. H. Systematic interpretation of Raman spectra of organic compounds. III—carbonyl compounds. *J. Raman Spect.* **2005**, *7* (3), 125-129. DOI: 10.1002/jrs.1250070304
(19) Krasser, W.; Kettler, U.; Bechthold, P. S. Luminescence and enhanced raman spectra of pure and pyridine-bonded small silver clusters. *Chem. Phys. Let.* **1982**, *86* (3), 223-227. DOI: 10.1016/0009-2614(82)80195-4
(20) Kuhne, T. D.; Iannuzzi, M.; Del Ben, M.; Rybkin, V. V.; Seewald, P.; Stein, F.; Laino, T.; Khaliullin, R. Z.; Schutt, O.; Schiffmann, F.; et al. CP2K: An electronic structure and molecular dynamics software package - Quickstep: Efficient and accurate electronic structure calculations. *J. Chem. Phys.* **2020**, *152* (19), 194103. DOI: 10.1063/5.0007045
(21) He, X.; Man, V. H.; Yang, W.; Lee, T. S.; Wang, J. A fast and high-quality charge model for the next generation general AMBER force field. *J. Chem. Phys.* **2020**, *153* (11), 114502. DOI: 10.1063/5.0019056
(22) Adamo, C.; Cossi, M.; Barone, V. An accurate density functional method for the study of magnetic properties: the PBE0 model. *Journal of Molecular Structure: THEOCHEM* **1999**, *493* (1-3), 145-157. DOI: 10.1016/s0166-1280(99)00235-3
(23) Grimme, S.; Ehrlich, S.; Goerigk, L. Effect of the damping function in dispersion corrected density functional theory. *J. Comput. Chem.* **2011**, *32* (7), 1456-1465. DOI: 10.1002/jcc.21759
(24) Nikitin, A. M.; Lyubartsev, A. P. New six-site acetonitrile model for simulations of liquid acetonitrile and its aqueous mixtures. *J. Comput. Chem.* **2007**, *28* (12), 2020-2026. DOI: 10.1002/jcc.20721
(25) Essmann, U.; Perera, L.; Berkowitz, M. L.; Darden, T.; Lee, H.; Pedersen, L. G. A smooth particle mesh Ewald method. *J. Chem. Phys.* **1995**, *103* (19), 8577-8593. DOI: 10.1063/1.470117
(26) Bussi, G.; Donadio, D.; Parrinello, M. Canonical sampling through velocity rescaling. *J. Chem. Phys.* **2007**, *126* (1), 014101. DOI: 10.1063/1.2408420
(27) Guidon, M.; Hutter, J.; VandeVondele, J. Auxiliary Density Matrix Methods for Hartree-Fock Exchange Calculations. *J. Chem. Theory Comput.* **2010**, *6* (8), 2348-2364. DOI: 10.1021/ct1002225
(28) Berendsen, H. J. C.; van der Spoel, D.; van Drunen, R. GROMACS: A message-passing parallel molecular dynamics implementation. *Comput. Phys. Commun.* **1995**, *91* (1-3), 43-56. DOI: 10.1016/0010-4655(95)00042-e
(29) Frisch, M. F.; Trucks, G. W.; Schlegel, H. B.; Scuseria, G. E.; Robb, M. A.; Cheeseman, J. R.; Scalmani, G.; Barone, V.; Petersson, G. A.; Nakatsuji, H.; et al. Gaussian 16, Revision C.01. **2016**.
(30) Wang, J.; Wang, W.; Kollman, P. A.; Case, D. A. Automatic atom type and bond type perception in molecular mechanical calculations. *J Mol Graph Model* **2006**, *25* (2), 247-260. DOI: 10.1016/j.jmgm.2005.12.005
(31) Stewart, J. J. Optimization of parameters for semiempirical methods VI: more modifications to the NDDO approximations and re-optimization of parameters. *J. Mol. Model.* **2013**, *19* (1), 1-32. DOI: 10.1007/s00894-012-1667-x
(32) Becke, A. D. Density-functional thermochemistry. III. The role of exact exchange. *J. Chem. Phys.* **1993**, *98* (7), 5648-5652. DOI: 10.1063/1.464913
(33) Singh, U. C.; Kollman, P. A. An approach to computing electrostatic charges for molecules. *J. Comput. Chem.* **2004**, *5* (2), 129-145. DOI: 10.1002/jcc.540050204
(34) Wang, J.; Wolf, R. M.; Caldwell, J. W.; Kollman, P. A.; Case, D. A. Development and testing of a general amber force field. *J. Comput. Chem.* **2004**, *25* (9), 1157-1174. DOI: 10.1002/jcc.20035
(35) Bernetti, M.; Bussi, G. Pressure control using stochastic cell rescaling. *J. Chem. Phys.* **2020**, *153* (11), 114107. DOI: 10.1063/5.0020514
(36) Hess, B.; Bekker, H.; Berendsen, H. J. C.; Fraaije, J. G. E. M. LINCS: A linear constraint solver for molecular simulations. *J. Comput. Chem.* **1997**, *18* (12), 1463-1472. DOI: 10.1002/(sici)1096-987x(199709)18:12<1463::Aid-jcc4>3.0.Co;2-h
(37) Bakan, A.; Meireles, L. M.; Bahar, I. ProDy: protein dynamics inferred from theory and experiments. *Bioinformatics* **2011**, *27* (11), 1575-1577. DOI: 10.1093/bioinformatics/btr168
